# Sequence-Derived Markers of Drug Targets and Potentially Druggable Human Proteins

**DOI:** 10.3389/fgene.2019.01075

**Published:** 2019-11-15

**Authors:** Sina Ghadermarzi, Xingyi Li, Min Li, Lukasz Kurgan

**Affiliations:** ^1^Department of Computer Science, Virginia Commonwealth University, Richmond, VA, United States; ^2^School of Computer Science and Engineering, Central South University, Changsha, China

**Keywords:** drug targets, druggability, druggable human proteome, drug-protein interactions, protein-protein interactions, intrinsic disorder

## Abstract

Recent research shows that majority of the druggable human proteome is yet to be annotated and explored. Accurate identification of these unexplored druggable proteins would facilitate development, screening, repurposing, and repositioning of drugs, as well as prediction of new drug–protein interactions. We contrast the current drug targets against the datasets of non-druggable and possibly druggable proteins to formulate markers that could be used to identify druggable proteins. We focus on the markers that can be extracted from protein sequences or names/identifiers to ensure that they can be applied across the entire human proteome. These markers quantify key features covered in the past works (topological features of PPIs, cellular functions, and subcellular locations) and several novel factors (intrinsic disorder, residue-level conservation, alternative splicing isoforms, domains, and sequence-derived solvent accessibility). We find that the possibly druggable proteins have significantly higher abundance of alternative splicing isoforms, relatively large number of domains, higher degree of centrality in the protein-protein interaction networks, and lower numbers of conserved and surface residues, when compared with the non-druggable proteins. We show that the current drug targets and possibly druggable proteins share involvement in the catalytic and signaling functions. However, unlike the drug targets, the possibly druggable proteins participate in the metabolic and biosynthesis processes, are enriched in the intrinsic disorder, interact with proteins and nucleic acids, and are localized across the cell. To sum up, we formulate several markers that can help with finding novel druggable human proteins and provide interesting insights into the cellular functions and subcellular locations of the current drug targets and potentially druggable proteins.

## Introduction

Knowledge of the drug-target interactions is essential for numerous applications including screening of drug candidates (Schneider, 2010; Núñez et al., 2012; Dalkas et al., 2013; Tseng and Tuszynski, 2015), drug repositioning and repurposing (Chong and Sullivan, 2007; Haupt and Schroeder, 2011; Oprea and Mestres, 2012; Hu and Bajorath, 2013; Li et al., 2016), characterization and mitigation of side-effects of drugs (Lounkine et al., 2012; Wang et al., 2012b; Kuhn et al., 2013; Tarcsay and Keserű, 2013; Hu et al., 2014), and prediction of novel protein-drug interactions (Wang et al., 2016a; Lotfi Shahreza et al., 2017; Ezzat et al., 2018; Hao et al., 2019; Wang and Kurgan, 2019; Wang and Kurgan, 2018; Wang et al., 2019). Recent analysis reveals that over 95% of the currently known drug targets are proteins and that these proteins facilitate about 93% of known drug-target interactions (Santos et al., 2017). Thus, we focus on the drug-protein interactions and we use the term “drug target” as a synonym for the protein drug target. While earlier works report about 400 drug targets (Hopkins and Groom, 2002; Russ and Lampel, 2005), subsequent studies annotate as many as over 600 drug targets in human (Santos et al., 2017). Furthermore, the druggable human proteome, defined as the full complement of the human drug targets (Hopkins and Groom, 2002; Russ and Lampel, 2005; Rask-Andersen et al., 2014; Cimermancic et al., 2016; Hu et al., 2016), is expected to be much larger. Early estimates place the number of human drug targets at around 3,000 (Hopkins and Groom, 2002; Russ and Lampel, 2005). A more recent analysis approximates this number at 4.5 thousand (Finan et al., 2017), which corresponds to about 22% of the human genome. While the historically typical drug targets include G-protein coupled receptors, nuclear receptors, ion channels, and some of the enzymes (Overington et al., 2006; Imming et al., 2007), recent works suggest that many of the non-enzymes (e.g., scaffolding, regulatory, and structural proteins) and proteins involved in specific protein-protein interactions (PPIs) should be targeted by drugs (Makley and Gestwicki, 2013; Ozdemir et al., 2019), effectively expanding the list of potential drug targets. These observations point to the fact that many of the drug targets remain to be discovered and characterized. The search for these proteins relies on the concept of druggability, which was originally defined based on the presence of structure that favors interactions with drug-like compounds where the corresponding interactions provide desired therapeutic effects (Hopkins and Groom, 2002; Russ and Lampel, 2005; Keller et al., 2006). In a purely structural context, druggability is related to binding of a compound to a given protein target with high affinity (< 1 µM) (Sheridan et al., 2010; Radusky et al., 2014). We focus on the former definition where both the interactions and the therapeutic effects are considered.

One of the key elements in the quest to find druggable proteins is to identify functional and structural characteristics that differentiate drug targets from the non-drug targets (Zheng et al., 2006; Lauss et al., 2007; Bakheet and Doig, 2009; Zhu et al., 2009b; Zhu et al., 2009c; Bull and Doig, Mitsopoulos et al., 2015; 2015; Feng et al., 2017; Kim et al., 2017). In one of the earliest works, Chen *et al.* concentrated on the analysis of structural fold types, target family representation and similarity, pathway associations, tissue distribution, and chromosome location for the drug targets (Zheng et al., 2006). A similar analysis that considered cellular functions, pathway associations, tissue distribution, and subcellular and chromosome location of the drug targets was published soon after by Lauss and colleagues (Lauss et al., 2007). More recent studies have shifted the focus towards characteristic features of the target protein sequence and structure. Bakheet and Doig used a relatively small set of 148 targets to analyze several sequence properties (chain length, hydrophobicity, charge, and isoelectric point), putative secondary structure and transmembrane regions, inclusion of signal peptides, selected set of post-translational modifications (PTMs), as well as the previously studied subcellular location and functions (Bakheet and Doig, 2009). Subsequently, Bull and Doig investigated a similar set of characteristics using a much larger set of 1324 drug targets (Bull and Doig, 2015). They considered a similar set of sequence properties, native secondary structure and signal peptides, selected PTMs, and a few new properties: the number of germline variants, expression levels, and the number of PPIs (Bull and Doig, 2015). The most recent study by Park, Lee, and colleagues expanded the above list of characteristics by inclusion of gene essentiality and tissue specificity (Kim et al., 2017). Moreover, several articles narrowly focused on characteristics that quantify topological features of the underlying PPI networks (Zhu et al., 2009b; Zhu et al., 2009c; Mitsopoulos et al., 2015; Feng et al., 2017). While these studies have considered a broad range of functional and structural features of drug targets, they identified the drug target-specific characteristics by comparing the drug targets against the other human proteins (non-drug targets). However, many of these non-drug targets could be in fact druggable, i.e., as many as 22% according to (Finan et al., 2017). Using the non-drug targets to represent the non-druggable proteins in order to define characteristic features of the druggable targets ultimately creates a bias toward describing the currently known drug targets. Consequently, this reduces our ability to use these characteristics to identify a complete set of druggable proteins.

We address the abovementioned shortcoming of the prior works by comparing sequence-derived characteristics of the drug targets, possibly druggable proteins, and non-druggable proteins using a large and well-curated dataset of human proteins. Our study is novel in four ways. First, we contrast the drug targets (D dataset) not only against all non-drug targets (N dataset), which was also done in prior studies, but also against non-druggable non-drug targets (Nn dataset; the non-drug targets that exclude disease associated proteins) and against possibly druggable non-drug targets (Nd dataset; the non-drug targets that are associated with multiple diseases). The association of the non-drug targets with diseases is necessary for the druggable proteins to exert therapeutic effects. Second, we further compare the D, N, Nd, and Nd proteins against highly promiscuous drug targets that interact with many drugs (Dh dataset) and drug targets that interact with low number of drugs (Dl dataset). This full-spectrum analysis allows us to pinpoint characteristics that differentiate between drug targets, possibly druggable proteins and non-druggable proteins, as well as features that are specific to promiscuous *vs*. non-promiscuous drug targets. Third, we focus on the characteristics that can be quantified directly from the protein sequence or protein name/identifier. This facilitates their use as potential markers for druggability across the entire human proteome. This is in contrast to several related studies that are limited to a relatively small subset of human proteins with solved structures (Hambly et al., 2006; Bull and Doig, 2015; Hu et al., 2016; Wang et al., 2016a; Wang et al., 2019). Fourth, we include several important sequence/protein-derived characteristic that were missed in the past studies including putative intrinsic disorder, residue-level conservation, presence and number of alternative splicing isoforms, inclusion of domains, and solvent accessibility (surface area). Moreover, we cover some of the key characteristics from the prior works, such as the topological features of PPIs, cellular functions, and subcellular locations.

## Materials and Methods

### Datasets

#### Datasets of Drug Targets (D Dataset), Highly Promiscuous Drug Targets (Dh Dataset), and Low-Interaction Drug Targets (Dl Dataset)

We collect a comprehensive set of drug targets by combining interaction information extracted from several large bioactive compounds-protein interaction databases. We filter these bioactive compounds to include only approved and experimental drugs. Furthermore, we focus on human proteins by excluding protein fragments and proteins from other organisms. We maximize the coverage by first collecting an inclusive set of interactions (including all bioactive compounds and protein chains) and then applying the two filters to obtain a high quality and large set of drugs and proteins.

The data collection protocol follows the work in (Wang and Kurgan, 2019; Wang and Kurgan, 2018). We extract the source data from three large repositories: Drug2gene (Roider et al., 2014), TTD (Zhu et al., 2009a), and GtP (Harding et al., 2017). Drug2gene is one of the most inclusive repositories that aggregates 19 source databases including TTD and GtP and several other major databases like ChEMBL (Gaulton et al., 2016) and DrugBank (Wishart et al., 2017). However, Drug2gene includes older and substantially smaller version of the TTD and GtP resources. Therefore, we integrated the latest versions of these two databases into our dataset. These databases provide a list of drug-protein pairs that use different identifiers and which include other information that could be useful to identify these molecules (like drug structure). The arguably most popular way to identify drugs and proteins are the PubChem CIDs and UniProt accession numbers, respectively. We use these identifiers to map data between the resources. We also merged the drugs with different PubChem CID but identical *simplified molecular-input line-entry system* (SMILES) structures. First, we remove the data collected from TTD and GTP that lacks PubChem CID or UniProt identifiers. Next, we map the proteins in Drug2gene that are represented by Entrez Gene ID into the corresponding UniProt accession numbers. After mapping and combining these datasets and removing duplicates, we obtain 2,490,057 interactions for 591,684 bioactive compounds and 4,128 proteins. Next, we filter this list of compounds using the list of drugs obtained from the DrugBank and ChEMBL. We remove the compounds that do not have the same CID or SMILES structure when compared to the list of DrugBank and ChEMBL drugs. Finally, we remove non-human proteins using a reference human proteome from UniProt. At the end, the set of drug targets (D dataset) includes 33,104 interactions between 4,405 drugs (PubChem CID) and 1,638 protein (UniProt identifiers). We provide the complete D dataset in the **Supplementary Material**. Moreover, we generate an expanded set of human and human-like drug targets that includes proteins in the D dataset plus proteins from other organisms that share high sequence similarity to the human proteins (D+ dataset). More specifically, following recent works (Hu et al., 2014; Wang et al., 2016a; Wang et al., 2019), human proteins that share at least 90% sequence identity quantified using BLAST with default parameters (Altschul et al., 1997) to any of the drug targets were added into the D+ dataset. Consequently, the D+ dataset has 1,762 proteins including 124 proteins that were included based on the high similarity; we list these proteins in the **Supplementary Material**. The number of drug targets in our dataset is slightly higher than the sizes of the datasets used in related studies (in the inverse chronological order): 1604 in (Feng et al., 2017), 1578 in (Kim et al., 2017), 1324 in (Bull and Doig, 2015), and 1,030 in (Rask-Andersen et al., 2014). Compared to popular databases, such as KEGG DRUG and DrugBank, our dataset features a more complete set of interactions (33,104 *vs*. 14,222 and 23,380, respectively (Wang and Kurgan, 2019) while focusing on a smaller and relevant set drugs that specifically target human proteins [4,405 *vs*. 5,045 and 10,562, respectively (Wang and Kurgan, 2019).

Drug targets in our dataset interact with as few as 1 drug and as many as 443 drugs. We investigate whether sequence-derived and functional characteristics of highly promiscuous drug targets are different from the drug targets that interact with a few proteins. To do that we extracted two subsets of the drug targets, the highly promiscuous targets (Dh dataset) that correspond to the top quartile of the targets with the highest interaction counts, and the low-interaction drug targets (Dl dataset) that include the bottom quartile of the drug targets with the lowest numbers of interactions.

#### Dataset of Non-Drug Targets (N Dataset)

We contrast the sequence-derived and functional characteristics of the proteins in the D, D+, Dh, and Dl datasets against the proteins that are not current drug targets. We collect these non-drug targets (N dataset) by selecting proteins from the UniProt’s human proteome that are not in the D dataset. The selection process follows two rules. First, we match the size of the N dataset to the size of the D dataset to ensure robust statistical comparisons between different datasets. Second, when down-sampling the human proteins we ensure that the selected proteins have similar size as the proteins in the D dataset. More specifically, for each protein in the D dataset we pick a human non-drug target at random (without replacement) that has a matching sequence length (with 10% tolerance). We introduce the latter rule since the amount of intrinsic disorder in proteins is dependent on proteins length (HOWELL et al., 2012). The same selection process was used in several related studies (Meng et al., 2015b; Na et al., 2016; Meng et al., 2018) to eliminate protein size bias when studying intrinsic disorder. We provide the list of the 1,638 size-matched proteins that constitute the N dataset in the **Supplementary Material**. Moreover, Section “Non-druggable and possibly druggable proteins” describes how the N dataset is used to derive the dataset of Non-druggable non-drug targets (Nn dataset; the non-drug targets that exclude disease associated proteins) and the dataset of possibly druggable non-drug targets (Nd dataset; the non-drug targets that are associated with multiple diseases).

### Characterization of Protein Properties

We characterize a broad collection of characteristics of human proteins that include their disease associations, structural properties derived from the sequence (putative intrinsic disorder and surface), sequence properties (domain annotations, alternative splicing, and residue-level conservation), topological properties of the corresponding PPI network (centrality measures and hubs), and functional properties (GO annotations and predicted protein-binding regions). We extract these characteristics directly from the protein sequence or protein names/identifiers. This means that they could be used as potential markers for druggability that cover the entire human proteome.

#### Disease Associations

The protein-disease association data were collected from DisGeNET (Gutiérrez-Sacristán et al., 2016). DisGeNET integrates several curated databases and offers arguably one of the most complete levels of coverage for human diseases. This database provides association between disease MeSH IDs and Entrez Gene IDs and also provides a mapping between Entrez Gene IDs and UniProt identifiers. We mapped these annotations to our dataset using the UniProt identifiers.

#### Sequence-Derived Structural Properties

We annotate two relevant structural properties that we can accurately derive from the protein sequences: intrinsic disorder and solvent accessibility. We are unable to directly collect structural data since significant majority of the proteins in the D, D+, and N datasets do not have solved structures.

Intrinsically disordered proteins and protein regions lack a stable tertiary structure in isolation (Dunker et al., 2013; Habchi et al., 2014; Uversky, 2014a). Proteins with disordered regions are crucial for many key cellular functions including molecular recognition and assembly, cell cycle and cell death regulation, signal transduction, transcription, translation, and viral cycle (Dyson and Wright, 2005; Uversky et al., 2005; Liu et al., 2006; Xie et al., 2007; Peng et al., 2012; Xue et al., 2012; Peng et al., 2013; Uversky et al., 2013; Fan et al., 2014; Fuxreiter et al., 2014; Peng et al., 2014b; Xue and Uversky, 2014; Dolan et al., 2015; Meng et al., 2015a; Meng et al., 2015b; Varadi et al., 2015; Babu, 2016; Na et al., 2016; Yan et al., 2016; Wang et al., 2016b; Kjaergaard and Kragelund, 2017). They are also the main contributors to the dark proteome (Hu et al., 2018; Kulkarni and Uversky, 2018). Intrinsic disorder is abundant in the human proteins. Computational studies estimate that about 19% amino acids in eukaryotic proteins are intrinsically disordered (Peng et al., 2015) and over 40% human proteins have at least one long disordered region with 30 or more consecutive residues (Oates et al., 2013). These proteins are particularly relevant to this study since they are associated with several human diseases (Uversky et al., 2008; Babu, 2016; Uversky et al., 2014; Uversky, 2014b) and since they attract recent interest as potent drug targets (Cheng et al., 2006; Uversky, 2012; Dunker and Uversky, 2010; Ambadipudi and Zweckstetter, 2016; Tantos et al., 2015). Intrinsic disorder can be predicted accurately from protein sequence using computational methods (Peng and Kurgan, 2012; Walsh et al., 2015; Lieutaud et al., 2016; Meng et al., 2017a; Meng et al., 2017b). We use one of the leading disorder predictors, IUPred (Dosztányi et al., 2005; Dosztanyi, 2018). This selection is motivated by the fact that IUPred is computationally efficient (i.e., it can be used to process large datasets of proteins, such as the D and N datasets) and since it provides accurate predictions (Peng and Kurgan, 2012; Walsh et al., 2015). We use the IUPred’s results to compute the disorder content (fraction of disordered residues in a given protein) and the length of the putative disordered regions.

Solvent accessibility provides a crucial context for the analysis of the residue-level conservation since it allows us to separate conserved residues that are localized on the surface (which include residues that are instrumental for the drug-protein interaction) from those located in the protein core (which are likely responsible for structural stability of the protein). We predict the relative accessible surface area using the ASAquick method (Faraggi et al., 2014). This method predicts relative solvent accessibility from a single sequence (without alignment), and thus it much faster than the other predictors that require calculation of multiple sequence alignment. It also provides accurate prediction, which is why it was recently used in related studies (Zhang et al., 2017; Amirkhani et al., 2018; Meng and Kurgan, 2018). We convert the numeric relative solvent accessibility of residues into a binary annotation (solvent exposed *vs*. buried) using a threshold of 0.15. This value adequately splits the bimodal distribution of solvent accessibility values for the residues in the combined D and N datasets (**Figure S2** in the **Supplementary Material**). We use these results to quantify the fraction of the putative surface residues in a given protein.

We assess quality of these predictions by comparing values of the fraction of the native surface residues that are computed using a limited set of proteins that have structures against the fraction of the predicted surface residues for the same set of proteins. We utilize mapping generated with the SIFTS resource (Velankar et al., 2013) that is available in UniProt to identify structures of the human proteins from the D and N datasets in the PDB database (Berman et al., 2000). We consider structures that cover at least 90% of the corresponding full protein sequences collected from UniProt to ensure that they correspond to a similar set of residues that are covered by the predictions which rely on the full protein chains. We compute the native solvent accessibility from these structures in three steps. First, we remove other molecules (including other protein chains) from the PDB structures. Second, we use DSSP (Kabsch and Sander, 1983; Joosten et al., 2010) to compute solvent accessibility values. Third, we convert the solvent accessibility into the relative solvent accessibility values using the normalization procedure that is described in the ASAquick article (Faraggi et al., 2014). We were able to collect the native solvent accessibility values for 373 drug targets (including 343 proteins from the D dataset, 55 from the Dh dataset, and 103 from the Dl dataset) and 73 proteins non-drug targets (including 39 from the Nd dataset and 12 from the Nn dataset). This corresponds to (373 + 73)/(1762 + 1,638) = 13% structural coverage of the human proteins in our datasets. **Figure S3** compares the distributions of the fractions of the surface residues computed from the protein structures against the fractions that are based on the predicted solvent accessibility for the seven considered datasets. The distributions that rely on the native *vs*. putative solvent accessibility for each of the seven dataset are very similar. The differences are not statistically significant (*p*-values range between 0.17 for the N dataset and 0.88 for the Nd dataset). This results suggests that the solvent accessibility predicted with ASAquick provides an accurate approximation of the native fraction of the surface residues.

#### Protein Sequence Properties

We use the proteins sequences to annotate the domains, alternative splicing isoforms, and sequence conservation. We collect the domain annotations from Pfam (Calderone et al., 2013) using UniProt identifiers, and we use these annotations to compute the domain boundaries (fraction of the domain-assigned residues) and the number of domains per protein. We obtain the number of alternative splicing isoforms from the UniProt database (UniProt: the universal protein knowledgebase, 2016). We calculate residue-level conservation scores using the relative entropy measure (Wang and Samudrala, 2006) from the PSSMs generated with PSI-BLAST (Altschul et al., 1997). We use a threshold to convert the numeric conservation scores to binary, i.e., a given residue is either conserved (if its conservation score > threshold) or non-conserved (otherwise). We selected the threshold that corresponds to the 80^th^ percentile of the distribution of the conservation scores for the residues in the combined D and N datasets (**Figure S1** in the **Supplementary Material**). The corresponding threshold value of 0.63 corresponds to an inflection point in the distribution tail where the conserved residues should be located. Using these annotations, we quantify the rate of the conserved residues in the protein sequence and among the residues located on the putative protein surface, given that this is where the drug-protein interaction occurs.

#### Topological Properties of the Protein-Protein Interaction Network

Motivated by work in (Zhu et al., 2009b; Zhu et al., 2009c; Mitsopoulos et al., 2015; Feng et al., 2017), we quantify the topological characteristics of drug targets and non-drug targets in the human PPI network. We collected the interaction network from the MENTHA resource (Calderone et al., 2013) and directly mapped it to our datasets using UniProt identifiers. MENTHA integrates data coming from several popular databases of PPIs, such as IntAct (Orchard et al., 2014), MINT (Licata et al., 2012), DIP (Salwinski et al., 2004), BioGRID (Oughtred et al., 2019), and MatrixDB (Launay et al., 2015), providing arguably one of the most comprehensive coverage levels. Several different centrality measures can be used to define topological characteristics of proteins in PPI networks (Wang et al., 2013a). We considered a comprehensive set of measures including betweenness centrality (Freeman, 1977), eigenvector centrality (Bonacich, 1987), closeness centrality (Bavelas, 1950), information centrality (Stephenson and Zelen, 1989), degree centrality (Jeong et al., 2001), subgraph centrality (Estrada and Rodriguez-Velazquez, 2005), network centrality (Wang et al., 2012a), and local average connectivity (Li et al., 2011). We reduced this set by removing measures that are redundant (highly correlated). The corresponding subset of four measures (eigenvector, closeness, betweenness and information centrality) has relatively low mutual correlations (< 0.6) while being highly correlated (> 0.8) with at least one of the removed measures. We give the corresponding correlations between these measures on our datasets in **Table S1** in the **Supplementary Material**. The eigenvector centrality is an extension of the node degree in which connections to more important nodes have more impact on the score. The nodes that are connected to many highly connected nodes end up having higher score than nodes which are connected to the same number of less-connected nodes (Bonacich, 1987). The closeness centrality measures the average length of the shortest path from the node to other nodes. The nodes with higher closeness centrality on average have smaller distance to the other nodes (Bavelas, 1950). The betweenness centrality quantifies the frequency with which a given node appears in the shortest paths between nodes in the network. Thus, removal of nodes with high betweenness centrality has big impact on the shortest paths between nodes (Freeman, 1977). Finally, information centrality is based on information along the paths from a given node to the other nodes (Stephenson and Zelen, 1989).

Besides quantifying several different topological features, we also annotate hub proteins, defined as proteins that interact with many proteins (Jeong et al., 2001). While early works on hub proteins defined them using a fixed minimal number of (Jeong et al., 2001), more recent studies use a floating threshold defined as a certain percentage of the most connected nodes in a given interactome (Han et al., 2004; Batada et al., 2006; Dosztányi et al., 2006). This results in different cut-offs that define hubs for different interactomes (different organisms) and emphasizes the fact that hubs are a property of the whole interactome system rather than a property of individual proteins. We used the latter definition using the cut-off that corresponds to the 90^th^ percentile of the interaction counts in the complete human PPI network, which is consistent with several recent studies (Han et al., 2004; Batada et al., 2006; Dosztányi et al., 2006). Therefore, we annotate hub proteins as those that have the number of PPIs in the complete interactome collected from MENTHA that is higher than this threshold (i.e., ≥ 77 interactions).

Hub proteins have increased levels of intrinsic disorder (Meng et al., 2015b; Patil et al., 2010) and the disordered regions are often employed to carry out PPIs (Mohan et al., 2006; Vacic et al., 2007; Yan et al., 2016). The disordered protein-binding regions are also linked to certain human diseases (Uversky, 2018). Thus, we also annotate putative disordered protein binding regions. We use ANCHOR (Dosztányi et al., 2009) to predict the disordered protein-binding residues and we aggregate this information to compute the content of disordered protein binding residues for the proteins in our datasets. The selection of this method is motivated by the fact that it is accurate and popular, and provides fast predictions (i.e., is capable of processing our large datasets) (Meng et al., 2017; Katuwawala et al., 2019).

#### Functional Properties

We annotate cellular functions and subcellular locations of the drug targets and the non-drug targets using the Gene Ontology (GO) terms (Consortium, 2004), which we collect using the PANTHER system (Muruganujan et al., 2018). We annotate and separately analyze the molecular functions, biological processes, and cellular components, where the latter define the subcellular locations.

### Statistical and Similarity Analyses

We compare the sequence-derived and functional characteristics between the drug targets, non-drug targets, and possibly druggable proteins using statistical tests of significance of differences. We quantify the significance of the differences using the *t*-test if the underlying measure of the sequence-derived/functional property has normal distribution, and Wilcoxon rank-sum test otherwise. We used the Anderson-Darling test with the *p*-value cutoff of 0.05 to test normality. We use the Fisher’s exact test when comparing binary characteristics, including disease associations and presence of hubs.

We annotate the cellular functions and subcellular locations associated with a particular set of proteins using enrichment analysis offered by the PANTHER system (Muruganujan et al., 2018). This system generates a list of annotations that are statistically over-represented when compared with the annotations present in the whole human proteome. PANTHER quantifies the ratios of enrichment and the corresponding *p*-values for each GO term when compared with the reference human proteome. We focus on the GO terms that occur at least 10 times in our datasets (to ensure robustness of statistical analysis), and we annotate a given term as associated with a particular set of proteins if its ratio > 2 (at least two fold increase) and the associated *p*-value (quantified using the False Discovery Rate correction) is < 0.05.

We measure similarity between two sets of proteins by comparing the cellular function and subcellular location GO terms associated with these two protein sets. We calculate this similarity using the GOSemSim package (Li et al., 2010) with default parameters [Wang et al. measure (Wang et al., 2007)] and the reference set to human.

## Results and Discussion

### Non-Druggable and Possibly Druggable Proteins

**Figure 1:**
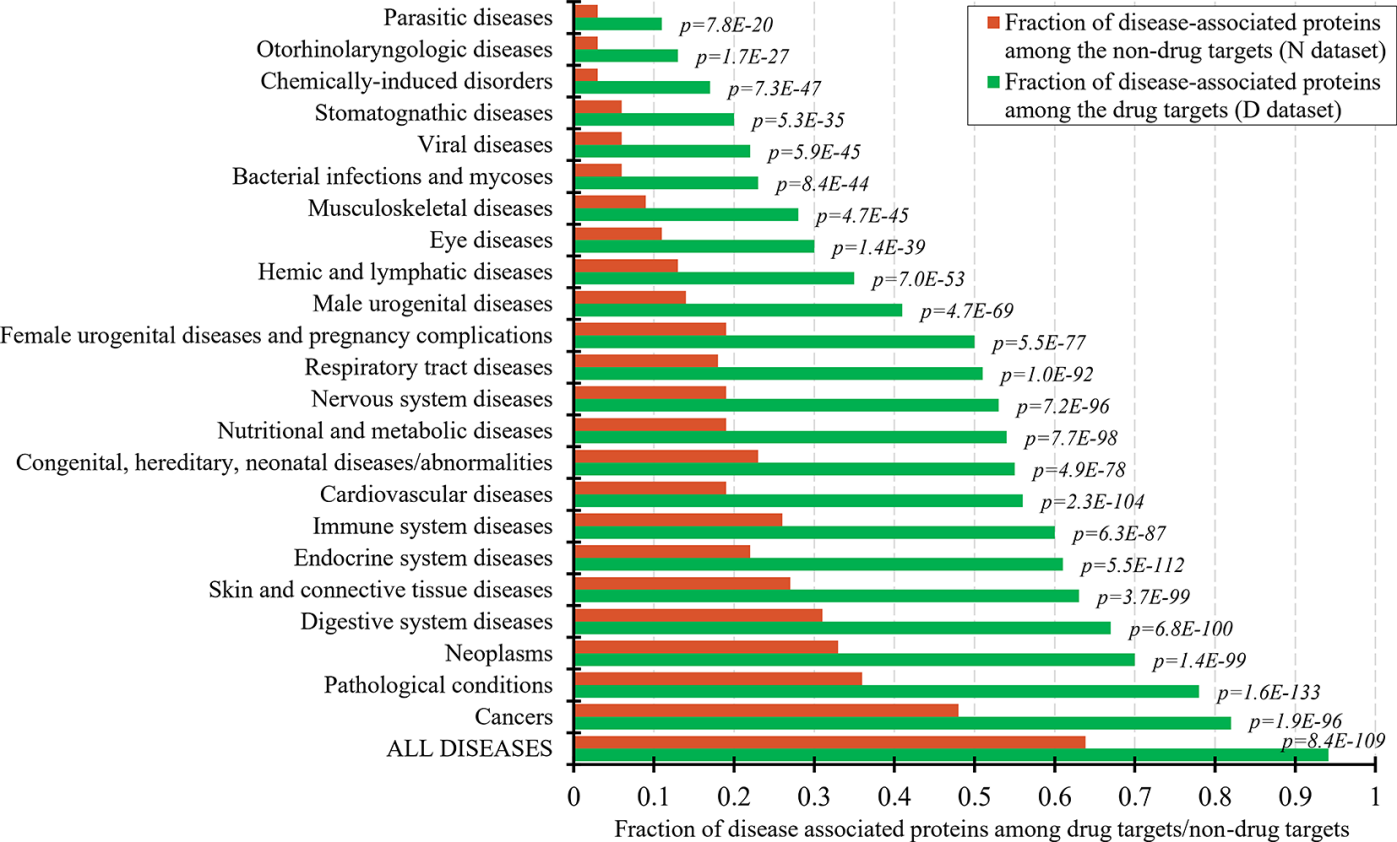
Fraction of drug targets and non-drug targets associated with different classes of diseases. The green and red bars show the fraction of disease associated proteins among the drug targets and non-drug targets for each disease class. The *p*-values quantify the significance of the differences between the two fractions using the Fisher’s exact test. The disease classes are sorted by the value of the fraction of the drug targets.

The set of the non-drug targets likely includes a relatively large number of druggable proteins. The ability to characterize properties that differentiate the drug targets and druggable proteins from the non-drug targets hinges on the annotation of the non-druggable and possibly druggable proteins in the set of these non-drug targets. Druggability of proteins requires that they interact with a drug-like compound and that this interaction provides a desired therapeutic effects (Hopkins and Groom, 2002; Russ and Lampel, 2005; Keller et al., 2006). Thus, one way to annotate possibly druggable and non-druggable proteins is to analyze protein-disease associations. **Figure 1** shows the fractions of the proteins associated with different classes of diseases among the drug targets and the non-drug targets. As expected, the number of the disease associated proteins is significantly higher among the drug targets compared to the non-drug targets. This difference is statistically significant for each of the 23 diseases classes (*p*-values < 0.0001). About 94% of the drug targets are associated with at least one disease, attesting to the relatively high coverage of these annotations and supporting the fact that the drug targets exert therapeutic effects. The largest fraction of the drug targets (82%) is associated with cancers. To compare, only about 64% of the non-drug targets are disease-associated. The latter suggests that the non-drug targets include both non-druggable proteins (those that lack association with any of the diseases) and possibly druggable proteins (those that are associated with diseases). We note that the use of the diseases associations provides a partial support for their druggability since it does not address the ability of the possibly druggable proteins to interact with drug-like molecules.

**Figure 2:**
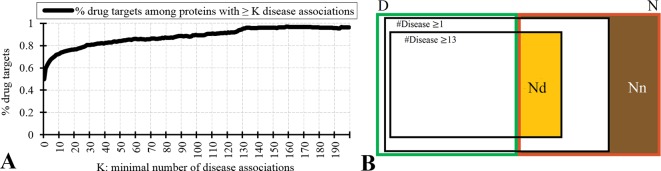
Relation between drug targets, non-drug targets and diseases associations. Panel **A** shows the fraction of the drug targets among proteins associated with a given minimal number of diseases K. Panel **B** is a Venn diagram that visualizes overlap between the disease associated proteins (with K = 1 and K = 13), the drug targets (dataset D; green border), and the non-drug targets (dataset N; red border). Among the non-drug targets we define the Nn dataset of non-druggable proteins (brown area), i.e., the non-drug targets that are not associated with any disease, and the Nd dataset of possibly druggable proteins (orange area), i.e., the non-drug targets that are associated with 13 or more diseases.

**Figure 2** analyzes relation between the drug targets, non-drug targets, and disease associations. **Figure 2A** reveals that the disease-associated proteins are likely to be drug targets. About 60% of proteins that are associated with at least one disease are drug targets. The fraction of drug targets increases for the proteins that are associated with more disease. This increase is sharper for a lower number of diseases and plateaus for proteins with about 10 or more disease associations. Therefore, we hypothesize that the non-drug targets with a relatively large number of disease associations can be used as a proxy for possibly druggable proteins. We use the inflection point in **Figure 2A**, which corresponds to proteins with ≥13 disease associations among which 75% are drug targets, to define the set of possibly druggable proteins. **Figure 2B** is a Venn diagram that visualizes overlap between the disease associated proteins (black borders), the drug targets (dataset D; green border), and the non-drug targets (dataset N; red border). We define the set of the non-drug targets that are associated with 13 or more diseases as possibly druggable proteins (Nd dataset; orange area in **Figure 2B**). **Figure 2B** also shows that virtually all drug targets are associated with at least one disease (black border with number of diseases K ≥ 1), while a large portion of the non-drug targets lacks any disease associations (brown area in **Figure 2B**). The latter set of proteins constitutes the set of the non-druggable proteins (Nn dataset).

We test reliability of annotations of the possibly druggable and non-druggable proteins using the 124 human-like drug targets from the D+ dataset that were annotated based on their high sequence similarity to drug targets in other organisms. We found only 4% (5 of the 124) of the human-like drug targets among the 4,869 non-drug targets that are not associated with diseases compared to 67% (83 human-like drug targets) that are among the 4,287 non-drug targets that are associated with 13 or more diseases. The high degree of the latter overlap suggests that the Nd dataset should include a substantial number of druggable proteins. We note that the 4% overlap with the non-drug targets that lack diseases associations likely stems from incompleteness of the diseases association data.

**Figure 3:**
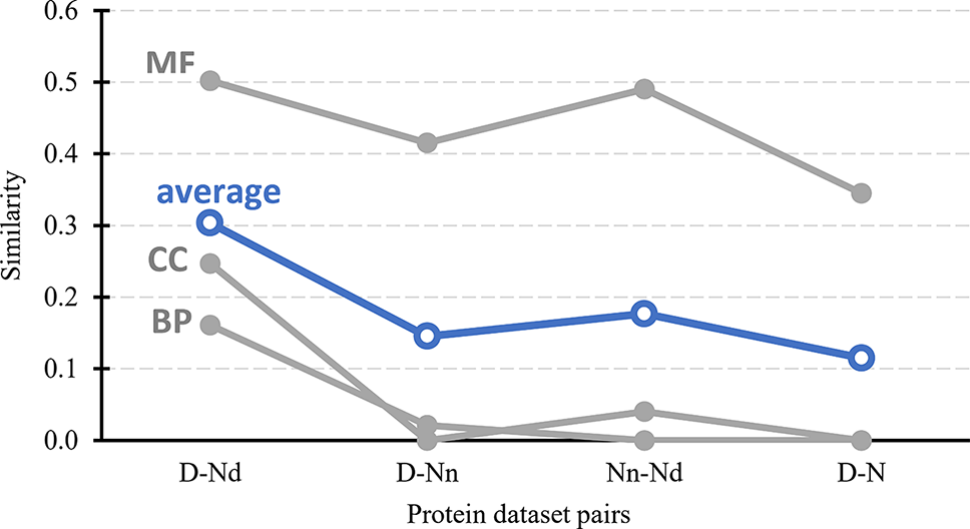
Similarity in cellular processes and subcellular locations between the drug targets (D dataset), possibly druggable proteins (Nd dataset), non-druggable proteins (Nn dataset), and non-drug targets (N dataset). We measure similarity for four pairs of these datasets (D *vs*. Nd, D *vs*. Nn, D *vs*. N, and Nn *vs*. Nd) based on the comparison of the corresponding sets of GO terms associated with these datasets, i.e., GO terms over-represented in a given dataset when compared to the entire human proteome. The GO terms are divided into three categories: MF (molecular functions), BP (biological processes), and CC (cellular components). Similarity was measured with the GOSemSim package (Li et al., 2010). We describe details of these calculations in section “Statistical and similarity analyses”. The gray markers show the similarity for each GO-term category while the blue markers are the average across the three categories.

**Figure 3** further tests the validity of the hypothesis that the Nd and Nn datasets include the possibly druggable and the non-druggable proteins, respectively. It quantifies similarity in the context of cellular functions and subcellular location between the drug targets, possibly druggable proteins, non-druggable proteins, and the non-drug targets. First, we generate a set of GO terms that are associated with each of these datasets, i.e., GO terms over-represented in a given dataset when compared to the human proteome. We perform this analysis separately for each of the three GO terms categories: molecular functions, biological processes, and cellular components; the latter is a proxy for the subcellular location. Next, we calculate similarity between the corresponding sets of dataset-specific GO terms; we describe the details in section “Statistical and similarity analyses”. The gray lines in **Figure 3** shows the similarity values for each GO term category while the blue lines show the average across the three categories. The left-most set of results reveals that the cellular functions and subcellular location of the drug targets (D dataset) are similar to the possibly druggable proteins (Nd dataset), which aligns with our hypothesis that the Nd dataset in fact includes druggable proteins. The second set of results, which compares the drug targets against the non-druggable proteins (Nn dataset), shows lack of similarity in the biological processes and subcellular locations and modestly reduced levels of similarity in the molecular functions. The corresponding average similarity = 0.145 is lower by a factor of two when compared with the similarity = 0.303 between the drug targets and possibly druggable proteins. The other two sets of results, which compare the possibly druggable against the non-druggable proteins and the drug targets against the non-drug targets, similarly reveal the lack of similarity in the biological processes and subcellular locations, while showing similarity in the molecular functions. The average similarities for these two dataset pairs are low and equal 0.177 and 0.115, respectively, suggesting that the corresponding two pairs of datasets include proteins involved in distinct cellular processes and subcellular locations. To sum up, the above analysis demonstrates that drug targets and the possibly druggable proteins share much higher levels of functional and subcellular location similarity compared to the similarity between possibly druggable proteins, non-druggable proteins, and non-drug targets. This finding, which uses an independent source of information compared to the approach we used to annotate the possibly druggable proteins, supports validity of our annotations of the possibly druggable and the non-druggable proteins.

### Comparative Analysis of the Sequence-Derived Structural and Functional Characteristics of the Drug Targets, Possibly Druggable, and Non-Druggable Proteins

Our ability to identify novel druggable proteins relies on the understanding of functional and sequence-derived characteristics that differentiate drug targets from the non-drug targets. We focus specifically on the characteristics that can be quantified from the protein sequence and/or identifier, which allows for a proteome-wide deployment. We compare a broad range of these characteristics between the drug targets, non-drug targets, possibly druggable proteins, and non-druggable proteins. We also investigate differences between the above protein sets and the expanded set of drug targets that includes human and human-like targets (D+ dataset), highly promiscuous drug targets that interact with many drugs (Dh datasets), and drug targets that interact with a low number of drugs (Dl dataset).

#### Characteristics Derived From the Protein Sequence

**Figure 4:**
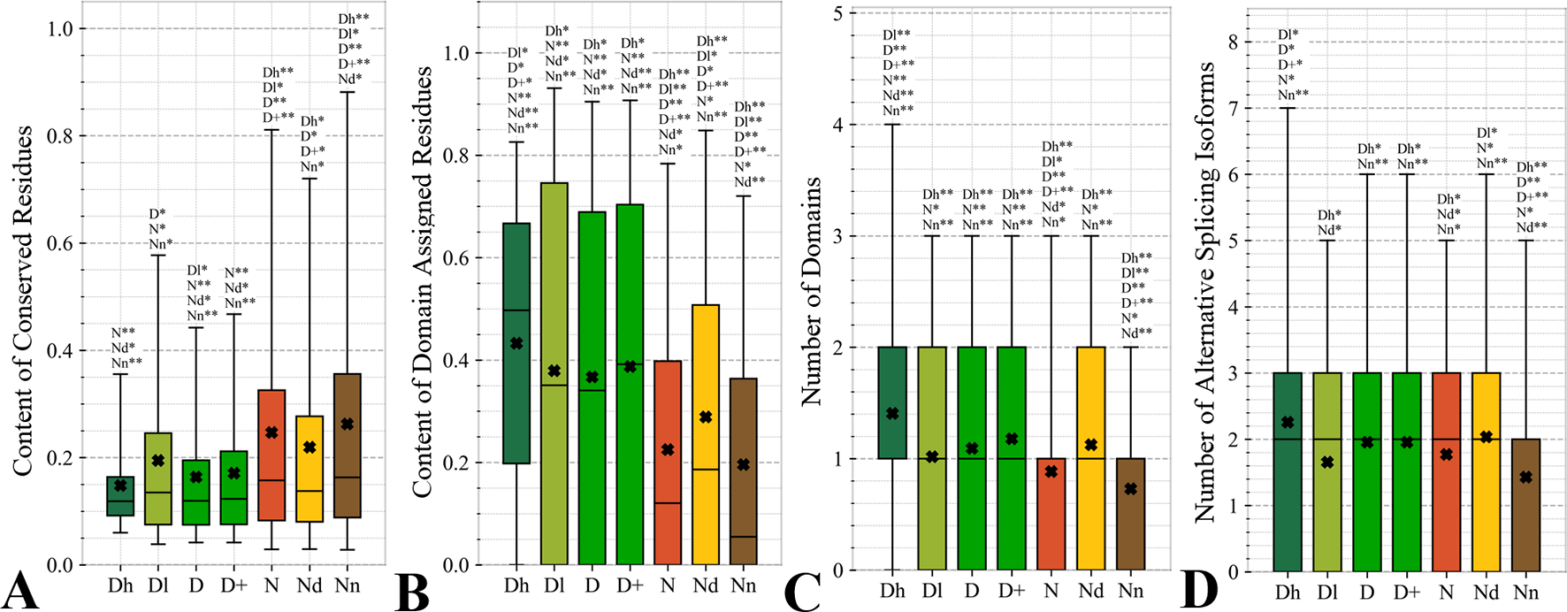
Distributions of the values of the sequence-derived characteristics for the highly promiscuous drug targets (Dh), drug targets that interact with a low number of drugs (Dl), all drug targets (D), all human and human-like targets (D+), non-drug targets (N), possibly druggable proteins (Nd), and non-druggable proteins (Nn). (Panels **A**) shows the amount of conserved residues. Panels **B** and **C** focus on the protein domains while Panel **D** quantifies the number of splicing isoforms. The whiskers show the 5 and 95 percentiles, the top and bottom of the box correspond to the first and third quartiles, the middle bar is the median, and the cross marker is the average. The annotation above the whiskers show the significance of differences with the other protein sets; only significant differences are listed where N* means p-value 0.05 and N** means p-value 0.0001 when compared with the N dataset. We explain calculation of statistical tests in section “Statistical and similarity analyses”.

**Figure 5:**
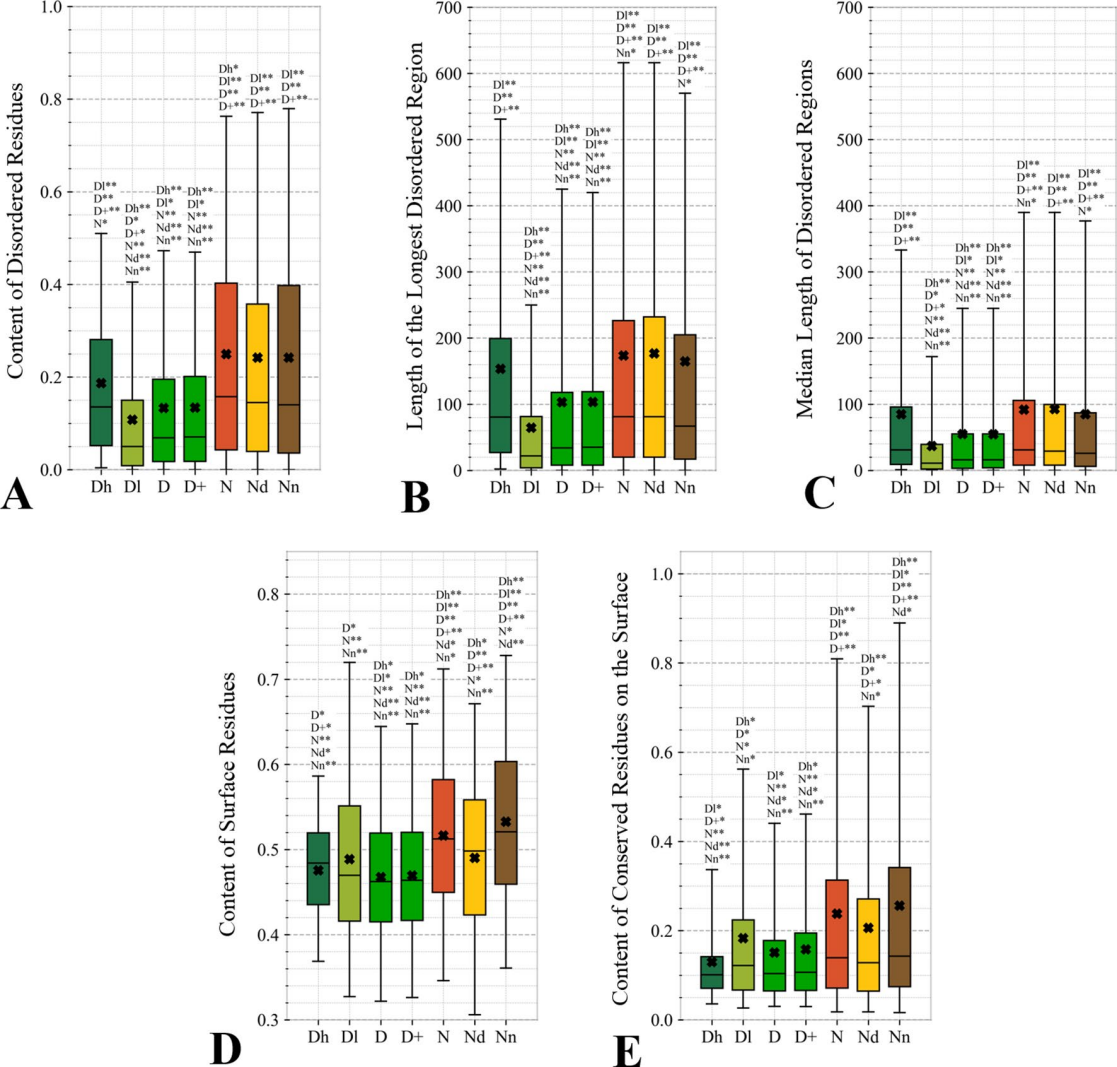
Distributions of the values of the sequence-derived structural characteristics predicted from the protein sequence for the highly promiscuous drug targets (Dh), drug targets that interact with a low number of drugs (Dl), all drug targets (D), all human and human-like targets (D+), non-drug targets (N), possibly druggable proteins (Nd), and non-druggable proteins (Nn). Panels **A**, **B**, and **C** quantify the abundance of intrinsic disorder while Panels **D** and **E** quantify the amount of surface and the amount of conserved residues on the surface, respectively. The whiskers show the 5 and 95 percentiles, the top and bottom of the box correspond to the first and third quartiles, the middle bar is the median, and the cross marker is the average. The annotation above the whiskers show the significance of differences with the other protein sets; only significant differences are listed where N* means p-value 0.05 and N** means p-value 0.0001 when compared with the N dataset. We explain calculation of statistical tests in section “Statistical and similarity analyses”.

**Figure 4** focuses on the characteristics derived directly from the protein sequence, including the residue-level conservation (content of conserved residues in protein chains), number of domains and the content of domain-annotated residues, and the number of the alternative splicing isoforms. **Figure 4A** shows that the drug targets (both D and D+ datasets) have significantly fewer conserved residues than the non-drug targets, possibly druggable proteins and the non-druggable proteins (*p*-value < 0.05). The possibly druggable proteins (orange bars) have significantly lower numbers of conserved residues compared to the non-druggable proteins (brown bars) (*p*-value < 0.05). Moreover, the highly promiscuous drug targets have significantly lower numbers of the conserved amino acids than the non-drug targets and the non-druggable proteins (*p*-value < 0.05), while maintaining similar levels compared to the possibly druggable proteins. Altogether, relatively low numbers of the conserved residues are characteristics for the drug targets and these numbers are also relatively low among the possibly druggable proteins. Interestingly, the residue-level conservation of the residues on the protein surface, where the protein-drug interaction occurs, follows the same pattern (**Figure 5E**). This finding complements prior results that show that drug targets have lower evolutionary rates and higher similarity to orthologous genes (Lv et al., 2016).

**Figures 4B, C** reveal that the drug targets (both D and D+ datasets) have substantially more domains and have larger amounts of domain-annotated residues when compared to the non-druggable proteins (*p*-value < 0.0001). At the same time, they a similar number domains when contrasted with the possibly druggable proteins. Furthermore, the possibly druggable proteins have significantly higher levels of domain annotations when contrasted against the non-druggable proteins (*p*-value < 0.0001). The underlying reasons for this enrichment could be two-fold. First, there could be proportionally more multi-domain proteins among the drug targets and the possibly druggable proteins. Consequently, inclusion of a larger number of domains could increase the likelihood that these proteins host at least one druggable domain. However, our result could also mean that these proteins are more studied and understood, and thus their domain annotations are more complete. Moreover, the fact that at least close to half of proteins in all considered datasets have domain annotations, which suggests that they are functionally annotated, suggests that our functional similarity analysis in **Figure 3** should be robust.

The drug targets (both D and D+ datasets) and the possibly druggable proteins have significantly more splicing isoforms compared to the non-druggable proteins (*p*-value < 0.05) and this increase is even higher for the promiscuous drug targets (*p*-value < 0.001). This suggests that enrichment in the number of alternative splicing variants could serve as a marker for druggability. The alternative splicing was found to contribute to drug resistance (Siegfried and Karni, 2018; Zhao, 2019), which supports veracity of our result. Interestingly, recent studies suggest that targeting alternative splicing events could lead to therapeutic opportunities (Le et al., 2015; Siegfried and Karni, 2018). Our analysis also reveals that majority of the drug targets and the possibly druggable proteins have multiple isoforms. Thus, gene level analysis of drug targets may not be adequate, considering that these genes would encode multiple proteins.

Overall, we identified three potential sequence-derived markers of druggability. The drug targets and possibly druggable proteins share lower numbers of conserved residues and are more likely to have multiple domains and isoforms when compared to the non-druggable proteins. We also note that the results for the original set of human drug targets (D dataset) are consistent with the results for the expanded set of drug targets (D+ dataset).

#### Sequence-Derived Structural Properties

This study is the first to analyze two relevant sequence-derived structural characteristics that can be accurately predicted from the protein sequence: intrinsic disorder and solvent accessibility. Proteins with disordered regions are associated with a wide range of human diseases (Uversky et al., 2008; Uversky et al., 2014; Uversky, 2014b; Babu, 2016) while solvent accessibility determines protein surface where the drug-protein interaction happens. We note that while authors in (Kim et al., 2017) computed putative solvent accessibility, they only used it to analyze results concerning enrichment in the PTMs.

**Figures 5A–C** quantify two key aspects of the disorder: the overall content of disordered residues and the length of disordered regions. Proteins with higher disorder content are functionally distinct from structured proteins while long disordered regions are thought to correspond to disordered protein domains (Tompa et al., 2009; Pentony and Jones, 2010; Peng et al., 2014a). We observe that drug targets (both D and D+ datasets) are significantly less disordered (by a factor of two) and include much shorter disordered regions when compared with the non-drug targets, including both possibly druggable and non-druggable proteins (*p*-value < 0.001). This is in agreement with a recent study that demonstrates that the current drug targets are biased to exclude disordered proteins (Hu et al., 2016). There are several reasons for this bias. The protein structures are used during the rational drug design process (Gane and Dean, 2000; Lundstrom, 2006; Mavromoustakos et al., 2011; Lounnas et al., 2013) and to gain mechanistic insights into the protein-drug interactions (Pielak et al., 2009; Tan et al., 2013; Christopoulos, 2014) (Altschul et al., 1997; Wang and Samudrala, 2006; Calderone et al., 2013; Orchard et al., 2014; UniProt: the universal protein knowledgebase, 2016). The structures are also indispensable for modeling associated with drug repurposing and repositioning (Moriaud et al., 2011; Ma et al., 2013). This is while proteins with disordered regions are much less likely to have structures (Hu et al., 2018), partly because since they are explicitly avoided in the structural genomics pipeline (Linding et al., 2003; Oldfield et al., 2005; Mizianty et al., 2014). Interestingly, the highly promiscuous drug targets are enriched in disorder when contrasted with the overall set of drug targets and the low promiscuity drug targets (*p*-value < 0.0001), while their disorder levels are comparable to the possibly druggable proteins. This coincides with the observation that disordered regions are capable of interactions with multiple partners (Oldfield et al., 2008; Hu et al., 2017). Our results suggests that although low disorder amounts are a strong marker for the current drug targets, the set of possibly druggable proteins includes large amounts of disorder. In fact, the disordered proteins may become the key to unlocking a substantial portion of yet to be discovered druggable targets (Uversky, 2012; Hu et al., 2016), especially given their association with numerous human diseases (Uversky et al., 2008; Uversky et al., 2014; Uversky, 2014b; Babu, 2016).

The amount of the putative surface residues for the drug targets (both D and D+ datasets) is significantly smaller that for the non-drug targets, including the possibly druggable and non-druggable proteins (*p*-value < 0.0001), see **Figure 5D**. This could be driven by the fact that drug targets are often membrane proteins (Yildirim et al., 2007; Rajendran et al., 2010), which means that they have relatively low surface area compared to other proteins. They are also mostly structured proteins (Hu et al., 2016) that are more likely to have globular shape with more buried residues compared to more irregularly shaped/elongated disordered proteins (Peng et al., 2014b; Uversky, 2017). Moreover, presence of disordered regions on the protein surface also leads to an increase of the surface area compared to structured conformations (Wu et al., 2015). Interestingly, the possibly druggable proteins have comparable content of the putative surface residues with the low promiscuity drug targets, which is also significantly smaller when contrasted with the non-druggable proteins (*p*-value < 0.0001). This again, like in the case of the results in **Figure 4**, shows that the possibly druggable proteins are more similar to drug targets than to the non-druggable proteins. Finally, we observe that the number of conserved residues on the putative surface (**Figure 5E**) maintains the same relation between the different protein sets as the overall number of conserved residues shown in **Figure 4A**, i.e., significantly lower for drug targets (both D and D+ datasets), and lower for the possibly druggable proteins compared to the non-druggable proteins (*p*-value < 0.05).

#### Topological Features of the Protein-Protein Interaction Networks

**Figure 6:**
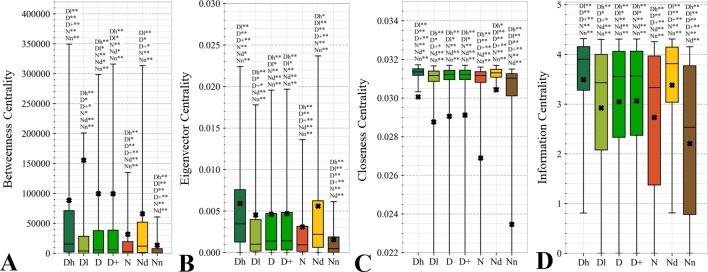
Distributions of the values of the selected orthogonal PPI network properties for the highly promiscuous drug targets (Dh), drug targets that interact with a low number of drugs (Dl), all drug targets (D), all human and human-like targets (D+), non-drug targets (N), possibly druggable proteins (Nd), and non-druggable proteins (Nn). Panels **A**, **B**, **C**, and **D** concern the betweenness centrality, eigenvector centrality, closeness centrality, and information centrality measures, respectively. The whiskers show the 5 and 95 percentiles, the top and bottom of the box correspond to the first and third quartiles, the middle bar is the median, and the cross marker is the average. The annotation above the whiskers show the significance of differences with the other protein sets; only significant differences are listed where N* means p-value 0.05 and N** means p-value 0.0001 when compared with the N dataset. We explain calculation of statistical tests in section “Statistical and similarity analyses”.

Topological features of the PPI networks are among the most studied characteristics of the drug targets (Zhu et al., 2009b; Zhu et al., 2009c; Bull and Doig, 2015; Mitsopoulos et al., 2015; Feng et al., 2017; Kim et al., 2017). A unique aspect of our analysis is that we focus on a set of orthogonal measures, i.e., measures that have low mutual correlations. This offers a more focused and balanced analysis given the high degree of similarity between many of these measures. **Figure 6** reveals that the entire set of four measures of centrality has significantly higher values for the drug targets (both D and D+ datasets) compared to the non-druggable proteins (*p*-value < 0.0001). Our results are in line with several prior studies that correspondingly show that drug targets have more connected and denser local network neighborhoods (Zhu et al., 2009b; Zhu et al., 2009c; Mitsopoulos et al., 2015; Lv et al., 2016). This finding suggests that drug targets are possibly more relevant biologically or are at a higher point of control and thus can better modify physiology, making them better therapeutic targets. The novel element in our study is that we find that all considered network centrality measures for the possibly druggable are even higher than for the drug targets (orange *vs*. green bars in **Figure 6**; *p*-value < 0.05). Consequently, they are also significantly higher than for the non-druggable proteins (orange *vs*. brown bars in **Figure 6**; *p*-value < 0.0001). Thus, our study suggests that these measures can be used as markers of druggability.

**Figure 7:**
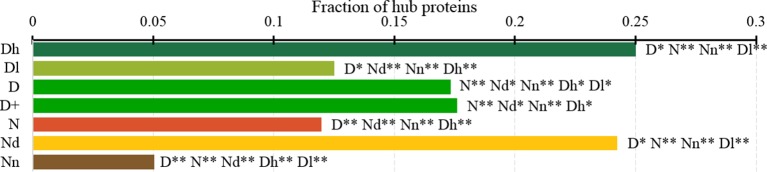
Fraction of hub proteins among the highly promiscuous drug targets (Dh), drug targets that interact with a low number of drugs (Dl), all drug targets (D), all human and human-like targets (D+), non-drug targets (N), possibly druggable proteins (Nd), and non-druggable proteins (Nn). The annotation next to the bars show the significance of differences with the other protein sets; only significant differences are listed where N* means p-value 0.05 and N** means p-value 0.0001 when compared with the N dataset. We explain calculation of statistical tests in section “Statistical and similarity analyses”.

**Figure 7** analyzes the abundance of the PPI network hubs among the drug targets, possibly druggable and non-druggable proteins. Approximately 17% of the drug targets (for both D and D+ datasets) are hubs and this rate is significantly higher than the 12% rate for the non-drug targets (green *vs*. red bars; *p*-value < 0.0001). Similarly large difference was observed in (Mitsopoulos et al., 2015). Our study reveals additional important details. We observe that the rate of hubs is very high among the highly promiscuous drug targets (25%) and the possibly druggable proteins (24%), and these rates are significantly higher than the 12% rate for the non-drug targets (*p*-value < 0.0001) and the 5% rate for the non-druggable proteins (*p*-value < 0.0001). This suggests that high connectivity in the PPI network is a strong marker for druggability.

### Functions and Subcellular Locations of Drug Targets and Possibly Druggable Proteins

**Figure 8:**
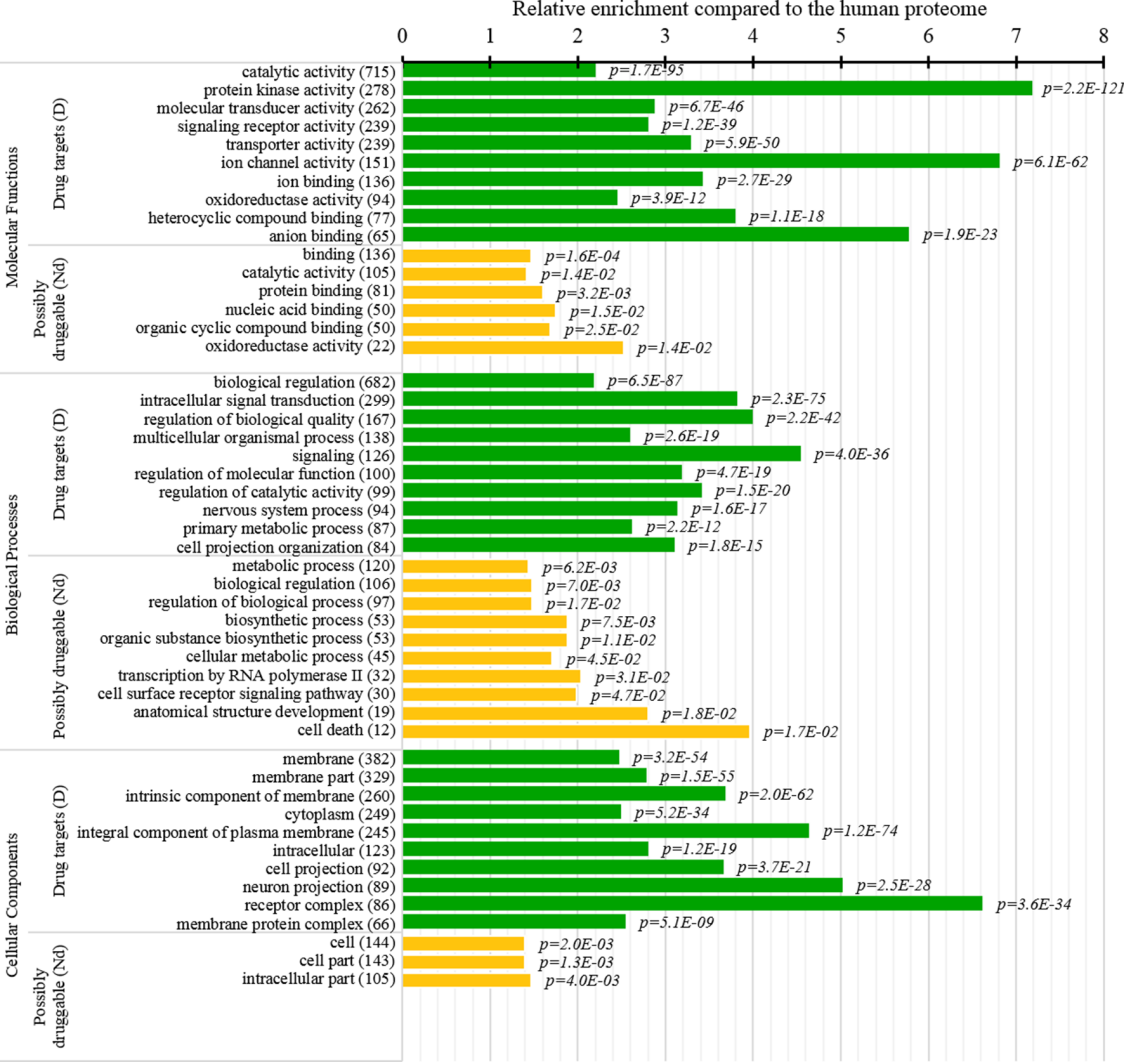
Molecular functions, processes, and subcellular locations that are enriched among the drug targets (D dataset) and the possibly druggable proteins (Nd dataset). We show the top 10 (with the highest counts) over-represented/significantly enriched GO terms for the drug targets (green bars) and the possibly druggable proteins (orange bars). The bars quantify the ratios of enrichment relative to the human proteome and the corresponding p-values are shown on the right. GO terms are identified on the left, including their names and the number of the correspnding proteins in the given dataset. We explain calculation of statistical tests in section “Statistical and similarity analyses”.

Several studies analyzed cellular functions and subcellular locations of the drug targets (Lauss et al., 2007; Bakheet and Doig, 2009; Wang et al., 2013b). The green bars in **Figure 8** provide a list of significantly enriched functions and locations for our set of drug targets. Our results indicate that most of the drug targets are enzymes, including kinases and oxidoreductases, followed by substatial numbers of channels, and in particular ion channels. They are often involved in binding, signalling, regulation, and transport. These finding are in close agreement with the results in (Bakheet and Doig, 2009). **Figure 8** also shows that drug targets are primarily found in membranes, with a large numbers also found in the cytoplasm and the intracellular space. Consistent results are found in (Bakheet and Doig, 2009; Wang et al., 2013b), and these subcellular locations also agree with the observation that membrane proteins are the prime targets for the development of therapeutics (Yildirim et al., 2007; Rajendran et al., 2010).

**Figure 9:**
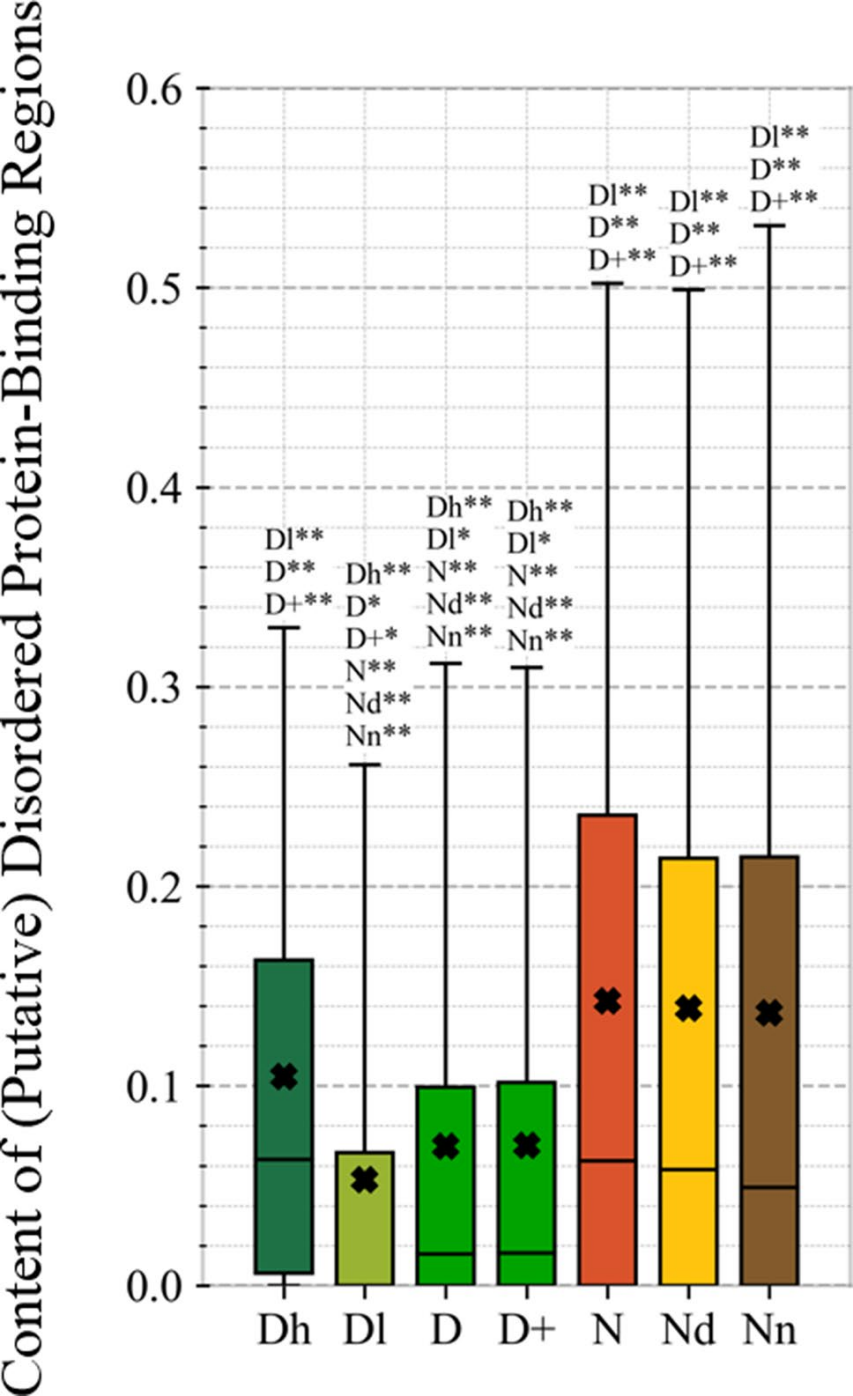
Content of putative protein binding regions in the highly promiscuous drug targets (Dh), drug targets that interact with a low number of drugs (Dl), all drug targets (D), all human and human-like targets (D+), non-drug targets (N), possibly druggable proteins (Nd), and non-druggable proteins (Nn). The annotation next to the bars show the significance of differences with the other protein sets; only significant differences are listed where N* means p-value 0.05 and N** means p-value 0.0001 when compared with the N dataset. We explain calculation of statistical tests in section “Statistical and similarity analyses”.

This study is the first to perform this type of analysis for the possibly druggable proteins (orange bars in **Figure 8**). Our analysis suggests that the possibly druggable proteins share functional similarities with the drug targets. They are similarly involved in the catalysis, signaling, and binding. However, the possibly druggable proteins tend to bind proteins and nucleic acids, instead of anions and ions which are the main partners for the drug targets. Moreover, the possibly druggable proteins are often involved in the metabolic and biosynthesis processes, and in the cell death cycle. The preference for the protein-protein and protein-nucleic acids binding and the cell death cycle involvement are supported by their significant enrichment in the intrinsic disorder (compared to the drug targets, see **Figures 5A, B**), and the fact that disordered regions are known to facilitate these types of functions (Vuzman and Levy, 2012; Uversky et al., 2013; Fuxreiter et al., 2014; Peng et al., 2015; Basu and Bahadur, 2016; Wang et al., 2016b; Hu et al., 2017; Srivastava et al., 2018). We further investigate this in **Figure 9** that analyzes the differences in the content of the putative disordered protein-protein binding regions. These results confirm the enrichment in the corresponding functional annotations for the possibly druggable proteins. The possibly druggable proteins include a substantial amount of the disordered protein-binding regions, on average about 14% of residues. Moreover, the drug targets (both D and D+ datasets) are significantly depleted in these protein-binding regions (on average only 7% of residues) when compared with the possibly druggable proteins (*p*-value < 0.0001). Interestingly, **Figure 8** also reveals that the possibly druggable proteins are localized across the cell and they do not have a specifically associated subcellular location, unlike the drug targets that are found mostly in the membranes and cytoplasm. Overall, our empirical analysis provides new insights into the cellular functions and subcellular locations of the druggable proteins.

## Summary and Conclusions

Recent research approximates that the druggable human proteome has about 4,500 proteins (Finan et al., 2017), while there are about 1,600 current drug targets (1,750 drug targets if we include proteins that share high sequence similarity to drug targets that were annotated in other organisms). Annotation of the remaining druggable human proteins would facilitate development and screening of drugs, drug repurposing and repositioning, understanding and mitigation of drug side-effects, and prediction of drug–protein interactions. We contrast the drug targets against the possibly druggable and non-druggable proteins to identify markers that could be used to identify novel druggable proteins. This is in contrast to the prior studies that compare drug targets against non-drug targets (Zheng et al., 2006; Lauss et al., 2007; Bakheet and Doig, 2009; Zhu et al., 2009b; Zhu et al., 2009c; Bull and Doig, 2015; Mitsopoulos et al., 2015; Feng et al., 2017; Kim et al., 2017), thus producing markers that describe current drug target and which implicitly exclude the druggable proteins that are included in the non-drug target set. We annotate the possibly druggable and non-druggable proteins based on the presence and promiscuity of disease associations, and we validate these annotations *via* functional similarity analysis.

**Figure 10:**
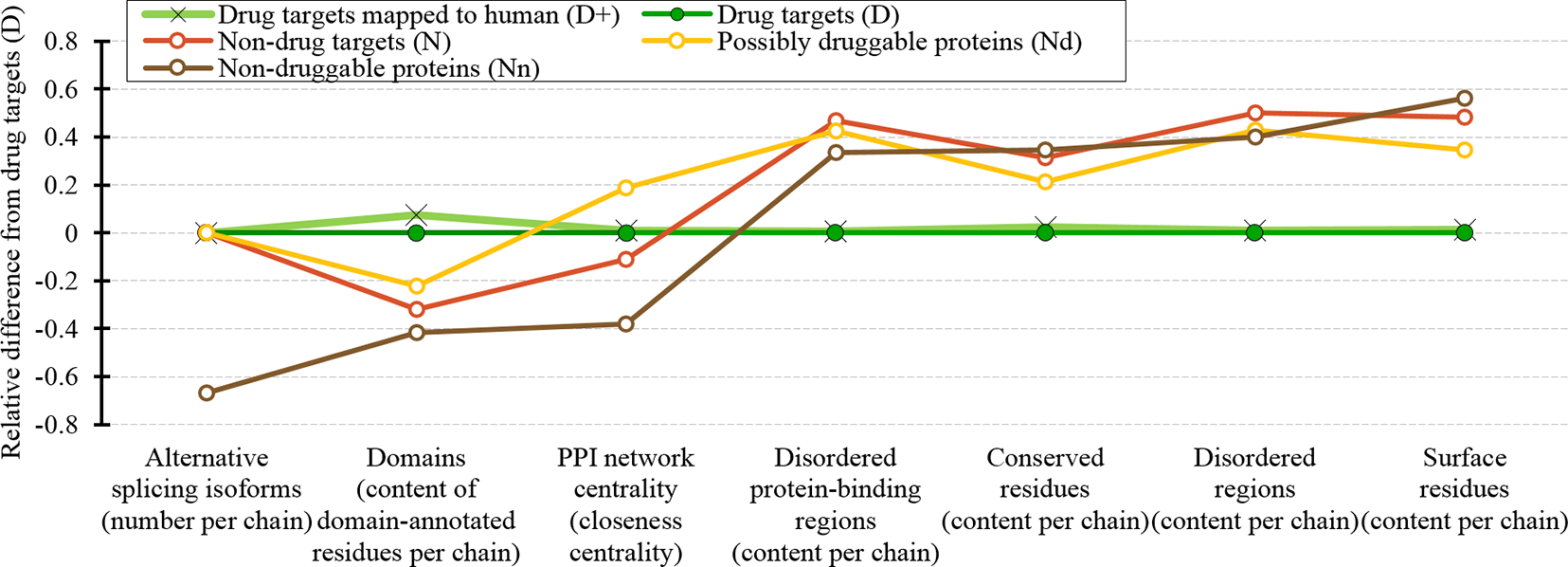
Overview of the sequence-derived markers for the drug targets (D), all human and human-like targets (D+), non-drug targets (N), possibly druggable proteins (Nd), and non-druggable proteins (Nn). The y-axis quantifies the relative difference of the values of a given protein set X compared to the values of the drug targets (D) set defined as: [median(X)-median(D))/IQR(D), where IQR means the interquartile range. The markers are sorted in the ascending order by the difference for the non-druggable proteins (in brown).

We cover a wide range of sequence-derived characteristics to define these markers. These characteristics can be computed across the entire human proteome, allowing for a complete sweep of all potential candidate proteins. We investigate several important characteristic that were missed in the past studies including putative intrinsic disorder, residue-level conservation, presence and number of alternative splicing isoforms, inclusion of domains, and putative solvent accessibility (surface area), as well as the key features from the prior works, such as the topological features of PPIs, cellular functions and subcellular locations. **Figure 10** summarizes the results. It shows the difference in the values of the key markers when comparing the possibly druggable proteins (in orange), the non-druggable proteins (in brown), all non-drug targets (in red), and the expanded set of human and human-like drug targets (in light green) against the human drug targets (in dark green). We observe that the possibly druggable proteins are significantly more similar to the drug targets than the non-druggable proteins for majority of the markers. These markers include high abundance of alternative splicing isoforms, relatively large number of domains, higher degree of centrality in the corresponding PPI network (and correspondingly much higher rate of hubs), lower number of conserved residues, and lower number of residues on the putative (sequence-derived) surface. Thus, these factors could serve as high-quality markers for druggability. “Results and discussion” section discusses these findings in the context of the current literature. Moreover, **Figure 10** shows that drug targets (both D and D+ datasets) have significantly depleted levels of intrinsic disorder and intrinsically disordered protein-binding regions when compared with the much higher and comparable levels among the possibly druggable and non-druggable proteins. This suggests that the high levels of disorder combined with the presence of the abovementioned markers should be used together to effectively enlarge the current collection of drug targets. This is in accord with several recent studies that postulate inclusion of the disorder-enriched proteins into the set of druggable proteins (Cuchillo and Michel, 2012; Uversky, 2012; Chen and Tou, 2013; Joshi and Vendruscolo, 2015; Ambadipudi and Zweckstetter, 2016; Hu et al., 2016; Yu et al., 2016).

Our analysis also shows that the possibly druggable proteins are functionally similar to the drug targets, being involved in the catalysis, signaling, and binding. The main difference is that the possibly druggable proteins target interactions with proteins and nucleic acids, unlike the current drug targets that favor interactions with anions and ions. **Figure 10** points to the high amount of the disordered protein-binding regions for the possibly druggable proteins compared to the drug targets, which is in concert with the disordered nature of the druggable proteins. This is in agreement with the literature that shows that disordered regions often facilitate PPIs (Mohan et al., 2006; Vacic et al., 2007; Fuxreiter et al., 2014; Yan et al., 2016; Hu et al., 2017). Finally, we show that the possibly druggable proteins are involved in the metabolic and biosynthesis processes and that they are localized across the cell, without a preference for specific subcellular locations. This is unlike the current drug targets that are located primarily in the membranes.

To sum up, our empirical analysis has led us to formulate several markers that may help with identifying novel druggable human proteins and has produced interesting insights into the cellular functions and subcellular locations of potentially druggable proteins.

## Data Availability Statement

All datasets generated for this study are included in the article/**Supplementary Material**.

## Author Contributions

LK conceptualized the study. LK and ML designed the study. SG organized the source databases. SG and XL performed acquisition of data. SG and LK organized and performed statistical analysis. All authors organized, analyzed and interpreted the results. LK and SG wrote the first draft of the manuscript. All authors contributed to manuscript revision, read and approved the submitted version, and provided approval for publication of the content.

## Funding

This research was supported in part by the Robert J. Mattauch Endowment funds to LK, the National Natural Science Foundation of China (No. 61832019), and the 111 Project (No. B18059).

## Conflict of Interest

The authors declare that the research was conducted in the absence of any commercial or financial relationships that could be construed as a potential conflict of interest.
