# Supplementary material for: Sequence-Derived Markers of Drug Targets and Potentially Druggable Human Proteins
Source: Front Genet. 2019 Nov 15;10:1075. doi: 10.3389/fgene.2019.01075 (PMC6872670; doi:10.3389/fgene.2019.01075)
Supplement: Supplementary file 1 [file DataSheet_1.docx]

**Supplementary Materials**

**“Structural and Functional Characteristics of Drug Targets and Druggable Human Proteins”**

**Sina Ghadermarzi^1^, Xingyi Li^2^, Min Li^2^*, and Lukasz Kurgan^1^***

^1^Department of Computer Science, Virginia Commonwealth University, Richmond, VA 23284, U.S.A.

^2^School of Computer Science and Engineering, Central South University, Changsha, 410083, P.R. China

*corresponding authors:

Lukasz Kurgan: Department of Computer Science, Virginia Commonwealth University, 401 West Main Street, Room E4225, Richmond, Virginia 23284, USA; Email: lkurgan@vcu.edu; Phone: (804) 827-398

Min Li: School of Computer Science and Engineering, Central South University, Changsha, 410083, P.R. China. Email: limin@mail.csu.edu.cn; Phone: +86 731 8887 9560


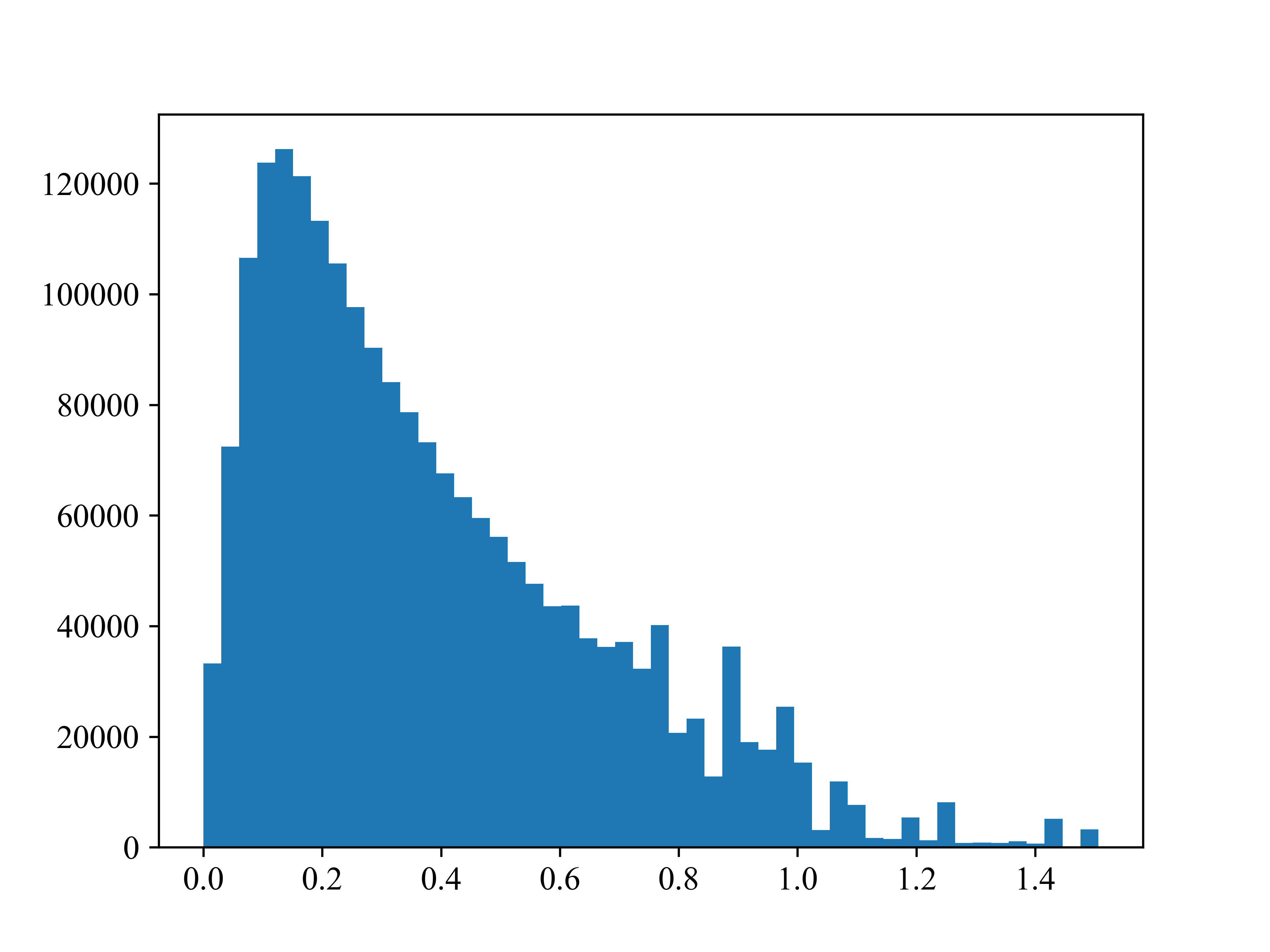


Conservation Scores

Number of proteins with scores in the range

**Supplementary Figure S1. Histogram of the conservation scores for the residues in the D and N datasets.** A threshold = 0.63, which corresponds to the 80^th^ percentile of the distribution, was selected to binarize the score.


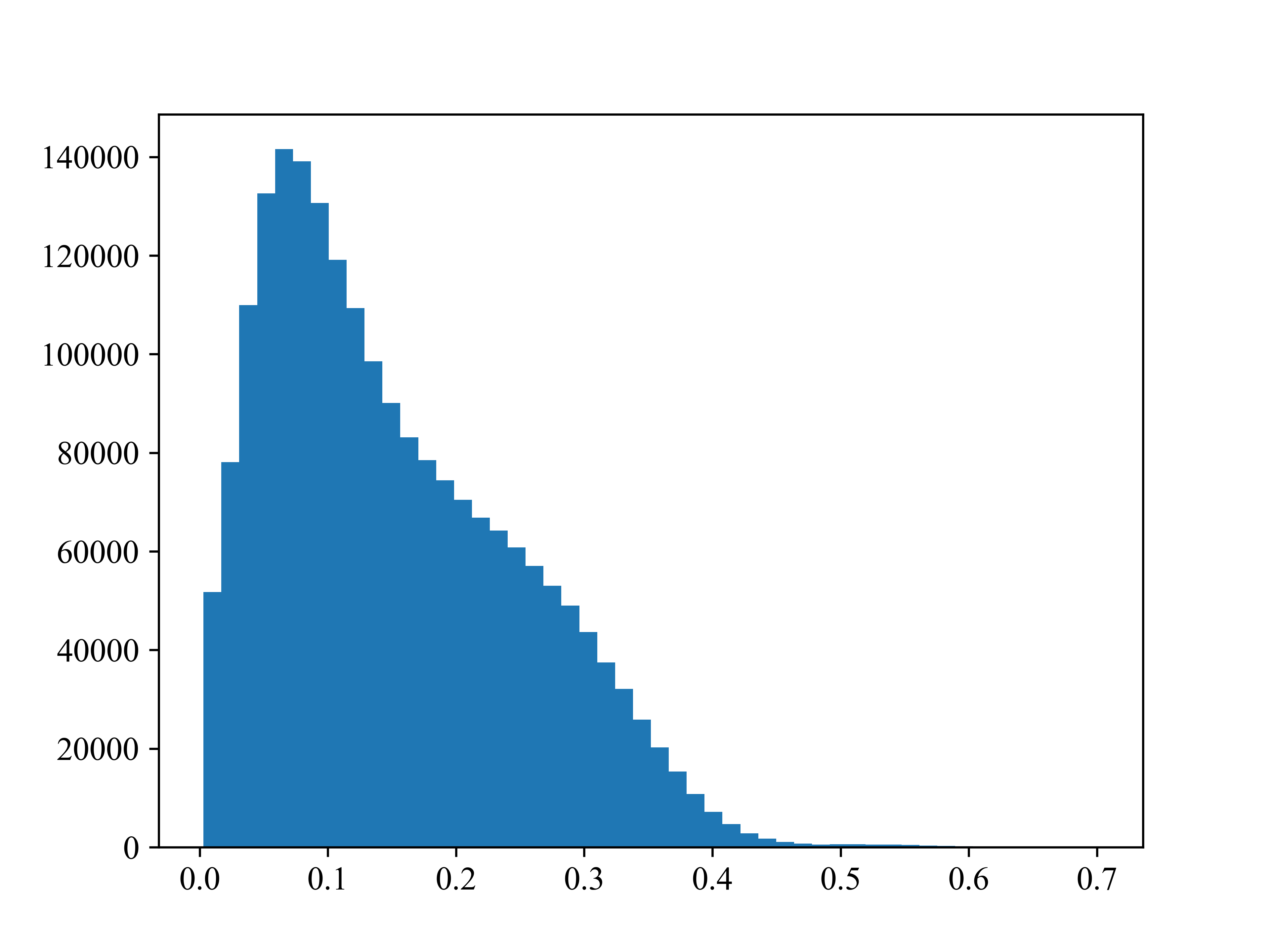


Number of proteins with predictions scores in the range

ASAquick Predictions

**Supplementary Figure S2. Distribution of the putative solvent accessibility values generated with the ASAquick method for the residues in the D and N datasets.** A threshold = 0.15 splits this bimodal distribution and was selected to binarize the putative solvent accessibility scores, i.e., solvent exposed residues > 0.15, buried ≤ 0.15.


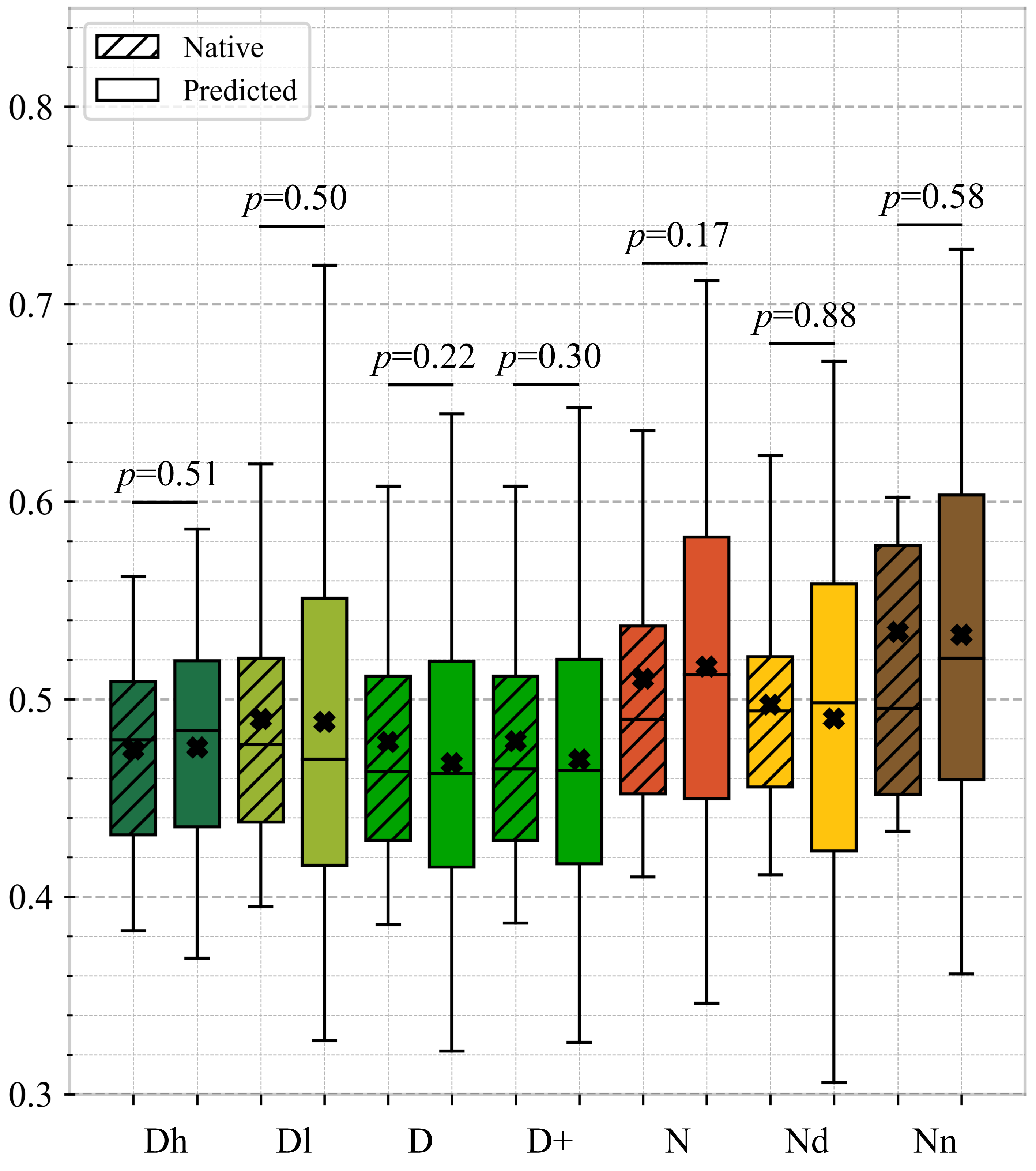


**Supplementary Figure S3. Comparison of the content of surface residues (fraction of the surface residues in a given protein) that were computed using the native relative solvent accessibility extracted from protein structures vs. when using putative relative solvent accessibility produced by the ASAquick method for the proteins in the D, D+, Dh, Dl, N, Nn and Nd datasets.** The whiskers show the 5 and 95 percentiles, the top and bottom of the box correspond to the first and third quartiles, the middle bar is the median, and the cross marker is the average. The p-values shown above the whiskers quantify the significance of differences between the content generated from the native solvent accessibility and the putative solvent accessibility for each dataset. We explain calculation of statistical tests in section 2.4.

**Supplementary Table S1. Matrix of correlations between centrality measures.** The (Pearson) correlations between the measures of Betweenness Centrality (BC), Eigenvector Centrality (EC), Closeness Centrality (CC), Information Centrality (IC), Degree Centrality (DC), Subgraph Centrality (SC), Network Centrality (NC) and Local Average Connectivity (LAC) computed over the combined set of proteins from the D and N datasets. There gray shaded numbers are high correlations (>0.8) between the two measures and the green shading highlights the selected measures.

|  | DC | IC | EC | SC | BC | CC | NC | LAC |
| --- | --- | --- | --- | --- | --- | --- | --- | --- |
| DC | 1.00 |  |  |  |  |  |  |  |
| IC | 0.41 | 1.00 |  |  |  |  |  |  |
| EC | 0.91 | 0.42 | 1.00 |  |  |  |  |  |
| SC | 0.82 | 0.15 | 0.83 | 1.00 |  |  |  |  |
| BC | 0.77 | 0.12 | 0.54 | 0.62 | 1.00 |  |  |  |
| CC | 0.17 | 0.23 | 0.18 | 0.08 | 0.06 | 1.00 |  |  |
| NC | 0.93 | 0.27 | 0.87 | 0.93 | 0.75 | 0.12 | 1.00 |  |
| LAC | 0.67 | 0.53 | 0.80 | 0.51 | 0.26 | 0.20 | 0.65 | 1.00 |

## **Drug targets dataset (D dataset)**

The list of proteins in the drug targets dataset in the following format
*<Sequential index>*|<*Uniprot ID>*(<*number of disease annotation>*) [<*comma separated* *list of PubChem CIDs of the interacting drugs>*]

The drug targets are sorted by the number of drug interactions.

1|P51511(26) [119031]

2|Q16762(12) [165331]

3|P55809(7) [16684434]

4|P51168(43) [16231]

5|P04350(15) [11351021]

6|P02818(88) [104625]

7|P32929(18) [1051]

8|Q01726(55) [16197727]

9|Q9NWM0(18) [446425]

10|P34130(17) [3671]

11|Q14376(12) [3561]

12|Q7Z418(5) [444899]

13|P14210(291) [42642645]

14|P22695(4) [3034285]

15|P51801(25) [4488]

16|Q8IZF0(5) [2520]

17|O00767(75) [24988881]

18|Q16719(14) [1051]

19|O43252(7) [10238]

20|Q99798(28) [14925]

21|Q15758(29) [193613]

22|P26440(17) [174251]

23|Q9HC62(8) [4362]

24|P09429(194) [12041]

25|Q8IUR0(0) [46937142]

26|P48551(48) [23724530]

27|Q92993(50) [10130120]

28|O14514(12) [6323481]

29|Q2TB90(2) [2090]

30|O00305(9) [208898]

31|P49190(3) [16132393]

32|P40306(10) [11556711]

33|P84077(14) [188347]

34|P12319(21) [45266800]

35|P02751(299) [3779]

36|Q9HCT0(0) [1369]

37|P12314(43) [157922]

38|P35225(275) [4031]

39|O14521(105) [4462]

40|P06400(137) [160355]

41|P61956(20) [54676538]

42|Q04828(34) [192197]

43|Q13324(44) [11223423]

44|Q9H1C0(6) [10322404]

45|P28845(65) [11670435]

46|O14732(5) [10112]

47|P12104(50) [448769]

48|P80511(60) [2161]

49|Q99571(24) [114709]

50|Q7Z4N2(9) [32051]

51|Q8NET8(13) [1195]

52|Q8IWT1(7) [11967800]

53|P14174(176) [5289613]

54|P38571(39) [11722]

55|Q14500(2) [888]

56|P10635(214) [3487]

57|P78540(24) [446122]

58|Q9Y697(0) [1051]

59|Q05315(20) [4362]

60|Q9BWD1(14) [165339]

61|Q9HB14(1) [3562]

62|Q13535(104) [3973]

63|P49895(22) [657298]

64|Q9UKU7(33) [165390]

65|O75908(11) [166558]

66|P35318(166) [3151]

67|P31151(40) [1369]

68|O43194(9) [32051]

69|P46459(12) [16842]

70|Q9BTU6(4) [60961]

71|P51449(25) [444795]

72|Q9Y5K3(5) [10198924]

73|P07737(40) [5288573]

74|P62136(14) [1973720]

75|Q53GD3(3) [10198924]

76|Q14116(333) [5478883]

77|P32320(52) [100016]

78|P21439(57) [153997]

79|P37268(24) [9874248]

80|P09467(34) [24770445]

81|P19623(7) [446425]

82|P20783(57) [3671]

83|P23284(19) [145742]

84|A0A0A6YYG9(0) [588963]

85|Q9UGN5(9) [23725625]

86|O60895(17) [70691388]

87|O75907(17) [72281]

88|P05231(873) [3671]

89|P48552(28) [9549223]

90|P26599(93) [3117]

91|P51580(51) [1349907]

92|Q96CA5(24) [46940575]

93|O15270(10) [1051]

94|P27930(41) [444499]

95|P21912(97) [4462]

96|Q9HD40(7) [1051]

97|Q96SW2(16) [134780]

98|Q9NUV7(2) [1051]

99|O75116(58) [448042]

100|P51684(83) [2090]

101|P24347(60) [119031]

102|Q13885(8) [11351021]

103|Q9NRS4(19) [1701]

104|O14880(4) [6]

105|P29218(12) [10112]

106|P39905(125) [3671]

107|Q9NYG8(0) [5070]

108|Q9NPC2(15) [3562]

109|P24298(104) [1051]

110|Q16348(6) [19003]

111|Q13507(21) [934]

112|P43657(12) [10322404]

113|P15531(134) [446090]

114|P62993(38) [3003921]

115|P19320(186) [216325]

116|O60427(18) [24988881]

117|P51170(37) [16231]

118|P16930(35) [446167]

119|O75751(88) [199]

120|P05162(18) [101798]

121|O60669(9) [10413]

122|Q8NHU3(7) [10198924]

123|Q9UQQ2(58) [10113978]

124|Q9Y6F1(9) [23725625]

125|Q9Y259(19) [10198924]

126|P27448(2) [72271]

127|Q6UVM3(0) [5413]

128|P45452(138) [466151]

129|P06756(67) [10950142]

130|Q13477(16) [9865554]

131|P48061(249) [107782]

132|P30926(14) [10517]

133|P29973(17) [24316]

134|P43005(22) [107883]

135|Q9NS85(9) [11967800]

136|P18433(32) [6914659]

137|O43617(0) [46937142]

138|P21730(54) [11151928]

139|Q5T3U5(9) [5722]

140|Q9HCR9(37) [110635]

141|Q99418(2) [2812]

142|P54750(2) [443955]

143|O15511(2) [588963]

144|Q9ULZ9(20) [119031]

145|P51164(4) [2333]

146|P68133(44) [5289288]

147|Q16853(33) [3675]

148|Q9BZM2(1) [155815]

149|P54284(3) [208898]

150|Q99584(6) [2161]

151|P01009(195) [1567]

152|P04629(159) [126565]

153|Q96AG4(9) [183797]

154|P15169(8) [10308002]

155|P49682(151) [15560447]

156|Q9Y4L1(10) [216210]

157|P14635(75) [160355]

158|Q14833(15) [68841]

159|P63316(16) [3033825]

160|P54760(98) [10458325]

161|O00763(13) [4091]

162|P51649(20) [1110]

163|Q15149(47) [10168]

164|P43489(39) [3083542]

165|P38435(18) [2197]

166|Q99677(0) [10322404]

167|P00390(51) [65359]

168|P55085(110) [107985]

169|Q9Y5X9(25) [4670]

170|Q9HC29(223) [1552036]

171|P61158(5) [588963]

172|P18545(18) [3758]

173|Q9UQD0(59) [216327]

174|Q14242(50) [10239]

175|P25092(21) [16158208]

176|Q9H427(0) [3562]

177|Q9HB15(3) [3562]

178|Q15465(177) [24776445]

179|P08842(109) [5287541]

180|P99999(13) [54675783]

181|P02585(0) [3333]

182|Q9NR21(0) [23725625]

183|P28065(61) [11556711]

184|O14957(1) [3034285]

185|Q9UI33(18) [11967800]

186|P59998(0) [588963]

187|P22830(34) [157922]

188|P51810(12) [6047]

189|P04181(23) [1051]

190|O00750(12) [56949517]

191|P10599(121) [219104]

192|Q8N4M1(0) [10198924]

193|Q96EN8(1) [1051]

194|P06744(78) [191445]

195|Q9H239(9) [119031]

196|Q9UHC9(20) [150311]

197|Q6XYB5(0) [1051]

198|Q9NY91(15) [44814423]

199|P10586(16) [9547959]

200|P05362(361) [11965427]

201|P31213(38) [57363]

202|P20292(42) [123723]

203|Q9Y296(5) [46937142]

204|P06280(73) [176077]

205|P05067(179) [53257383]

206|O60894(17) [70691388]

207|P24158(56) [107706]

208|Q07699(27) [11967800]

209|Q8WW43(5) [11560787]

210|Q495M3(1) [439280]

211|Q8IWA5(1) [10198924]

212|P31327(38) [121396]

213|O00220(83) [4495]

214|Q13002(16) [5282253]

215|P23945(66) [5361]

216|P35790(34) [10198924]

217|Q96LD8(4) [10104227]

218|Q96I15(25) [1051]

219|P08574(16) [3034285]

220|P52895(28) [10133]

221|Q99643(72) [4462]

222|P11226(332) [101798]

223|Q6YP21(2) [1051]

224|O75899(4) [11281011]

225|O94903(0) [1051]

226|Q7Z3E1(58) [23725625]

227|Q9HCX4(4) [16231]

228|O60603(326) [130704]

229|P04040(268) [3406]

230|Q8TE04(6) [39042]

231|P09884(18) [119182]

232|P52788(31) [446425]

233|P17174(16) [1051]

234|P00488(85) [1030]

235|Q9Y2K7(10) [6914666]

236|Q8TCG2(2) [60961]

237|P19021(39) [10972]

238|O60928(16) [1727]

239|Q96B36(20) [44516953]

240|Q99062(36) [70683024]

241|P12259(293) [204102]

242|Q99572(91) [49864916]

243|Q14896(30) [11689883]

244|O14763(77) [4495]

245|Q9Y617(37) [1051]

246|P07948(26) [5328940]

247|P35222(216) [10112]

248|P04637(848) [237]

249|Q5T4U5(0) [448875]

250|P00746(17) [21439]

251|Q9Y5R8(2) [46937142]

252|P11172(36) [161647]

253|Q99259(51) [1051]

254|P09466(96) [175468]

255|P61927(2) [451597]

256|Q86YB8(1) [16842]

257|Q6WRI0(0) [1051]

258|O15143(3) [588963]

259|Q8N5Z0(0) [1051]

260|Q03518(94) [208908]

261|Q16739(29) [51634]

262|P06732(10) [4635864]

263|Q9Y694(3) [11948288]

264|P29279(179) [71351]

265|Q99943(7) [501254]

266|Q15910(182) [66558664]

267|Q96BZ4(3) [10198924]

268|P32754(26) [115355]

269|P12004(291) [5804]

270|Q9UPY5(18) [3086668]

271|Q99973(26) [5282440]

272|P14550(78) [3406]

273|P47871(11) [44278361]

274|P22888(55) [25074887]

275|P51172(3) [16231]

276|Q99735(2) [6]

277|P10997(59) [107807]

278|P21281(10) [1701]

279|P01019(325) [10257882]

280|O95864(31) [24988881]

281|Q9Y600(8) [1051]

282|P30419(3) [446386]

283|P28702(28) [82146]

284|Q9UNA0(19) [9933197]

285|P49585(50) [10198924]

286|P30291(29) [24856436]

287|P00736(17) [4413]

288|O43603(7) [44623946]

289|P00480(37) [124992]

290|P17658(1) [5413]

291|Q9UDW1(0) [3034285]

292|O95477(213) [4912]

293|Q8WWI5(10) [10198924]

294|Q8N4W3(0) [70683024]

295|Q15059(6) [24871506]

296|Q9Y5R2(13) [119031]

297|Q9UK17(18) [516892]

298|P68366(6) [11351021]

299|P08684(157) [2812]

300|X6R8A1(0) [5936]

301|O95263(26) [3108]

302|P52961(3) [1808]

303|P08865(63) [16155604]

304|O43174(28) [9799888]

305|Q96KS0(23) [11256664]

306|Q02241(12) [10368812]

307|Q96A70(19) [1051]

308|P42330(53) [46883536]

309|Q8TDS7(1) [239]

310|P08700(100) [2161]

311|P13929(4) [1005]

312|Q01469(27) [448769]

313|P10912(92) [178024]

314|P30273(15) [45266800]

315|Q6PEY2(0) [11351021]

316|Q8N142(5) [12717]

317|P29460(148) [123648]

318|P35568(99) [24871491]

319|Q96BH3(1) [1014]

320|Q00987(271) [11609586]

321|Q9Y6K0(0) [10198924]

322|P15692(694) [54675783]

323|P52790(5) [46181428]

324|P51512(25) [119031]

325|Q8NCM2(4) [441074]

326|Q13443(37) [16070111]

327|Q9H015(34) [1045]

328|P16298(3) [42601552]

329|P01579(599) [444972]

330|Q16651(23) [17975425]

331|Q8TBG4(3) [1051]

332|Q8TD30(8) [1051]

333|Q9BUF5(2) [11351021]

334|Q8IVS2(31) [78058]

335|Q96EB6(199) [445154]

336|Q86SZ2(0) [46937142]

337|P23526(34) [23190]

338|P14927(5) [3034285]

339|Q9GZP0(36) [3038522]

340|P48664(4) [11412540]

341|Q13489(79) [46940575]

342|Q9BVA1(12) [11351021]

343|O60896(10) [70691388]

344|P12643(119) [2482]

345|P05413(29) [448769]

346|P51800(16) [4488]

347|Q96F10(26) [446425]

348|Q9BQE3(2) [11351021]

349|Q5JUK3(8) [5413]

350|Q15119(3) [6102763]

351|P35219(28) [11967800]

352|O15145(2) [588963]

353|P25963(118) [9820526]

354|O95255(59) [2333]

355|Q96IY4(79) [10308002]

356|Q96GA7(0) [1051]

357|Q08257(58) [54676038]

358|P12235(34) [25419]

359|Q8TBC4(1) [16720766]

360|Q06203(1) [2265]

361|P05981(17) [445843]

362|P02775(40) [11668]

363|Q8N119(11) [119031]

364|P13051(35) [46937051]

365|P49662(15) [42601552]

366|P17735(79) [1051]

367|P21589(71) [92199]

368|P68363(26) [11351021]

369|Q9Y6L7(0) [16741116]

370|Q9NRE1(30) [119031]

371|Q96BD0(7) [5311236]

372|P61278(159) [6058]

373|Q9UNK4(12) [155815]

374|P16581(178) [9811353]

375|P07098(3) [46936391]

376|O75390(20) [449575]

377|Q9NTG7(43) [24756910]

378|Q99788(14) [10473088]

379|P13716(46) [137]

380|P25106(90) [65015]

381|O95551(11) [3885]

382|P24752(43) [39941]

383|Q9GZT4(17) [1051]

384|P01130(155) [157922]

385|P52789(41) [46181428]

386|Q9HBL8(1) [1432578]

387|Q86TP1(10) [3108]

388|Q9BZM1(0) [155815]

389|Q9NZ20(10) [155815]

390|P51160(11) [5318980]

391|Q9BY64(5) [3380]

392|P01133(295) [10458325]

393|Q9NPA2(20) [119031]

394|P00973(27) [17754220]

395|Q9NWT6(16) [1286536]

396|P17787(11) [10131048]

397|Q96LB2(12) [2719]

398|P14151(98) [1057]

399|Q92753(0) [444795]

400|Q9HB55(8) [54671203]

401|Q9NY72(2) [11967800]

402|Q9H1D0(25) [888]

403|Q9NP87(8) [5282176]

404|Q02790(40) [46883536]

405|Q2NL67(3) [23725625]

406|Q99705(6) [6536]

407|Q8TCT1(1) [10198924]

408|Q9BY21(5) [10051843]

409|P35520(139) [286]

410|O00398(1) [10051843]

411|Q9H2S1(1) [6000]

412|Q92851(60) [42601552]

413|P08514(83) [44820670]

414|Q15056(12) [1595804]

415|Q9H306(0) [119031]

416|Q9UGP5(15) [5282176]

417|Q8NCG7(0) [3034010]

418|Q9UKP6(26) [10173280]

419|P69905(85) [23925]

420|P23368(7) [10132]

421|Q02643(40) [56928011]

422|P47985(30) [3034285]

423|P16442(129) [46937084]

424|O95470(8) [1051]

425|P07741(168) [5287565]

426|P16278(191) [2132]

427|P36537(1) [21138]

428|P10620(11) [6]

429|Q9H3S4(9) [5327150]

430|P05154(43) [16130644]

431|O00311(21) [57899889]

432|Q05932(25) [104758]

433|P51878(23) [42601552]

434|P11274(129) [24826799]

435|P00751(52) [159772]

436|Q99542(32) [119031]

437|Q8NE62(46) [10198924]

438|Q9UMX1(35) [447966]

439|Q03431(49) [16132393]

440|P55786(25) [15547703]

441|P41212(79) [57379345]

442|Q06609(98) [11282283]

443|Q5JW85(0) [444455]

444|P07910(20) [216210]

445|Q9Y5Z0(13) [44251605]

446|P41439(6) [6037]

447|P51677(70) [5486198]

448|P11216(9) [1051]

449|O75865(0) [46937142]

450|Q96C86(14) [147071]

451|Q9H6Z9(41) [11256664]

452|P29762(26) [3312]

453|Q7Z4W1(8) [1003]

454|Q8NEB9(11) [51001932]

455|Q02641(3) [208898]

456|Q86Y34(1) [21700]

457|Q96QK1(9) [183797]

458|P01008(69) [5282448]

459|O75900(7) [119031]

460|P51575(18) [1051]

461|O14949(8) [3034285]

462|Q07973(85) [10672195]

463|Q9UBU3(85) [11526696]

464|Q9BXA5(13) [1110]

465|O95954(8) [1051]

466|Q8IUZ5(0) [1051]

467|P36897(128) [447966 , 10090485]

468|Q9Y4P1(8) [2090 , 7329]

469|O15392(127) [476861 , 6237]

470|P27707(37) [119182 , 4470573]

471|P19113(24) [1051 , 172997]

472|Q15825(4) [4032 , 3604]

473|P48544(39) [1727 , 3488]

474|Q16881(84) [219104 , 104799]

475|Q9Y5S1(20) [16231 , 644019]

476|P06133(9) [10133 , 21138]

477|Q09472(122) [969516 , 65064]

478|Q16281(21) [6076 , 24316]

479|P49773(25) [168120 , 17753791]

480|P25025(135) [9838712 , 9865554]

481|P11474(35) [16122612 , 5280961]

482|P29972(76) [24316 , 5413]

483|Q92934(55) [153999 , 24978538]

484|P05091(153) [3117 , 4510]

485|Q14123(2) [17754438 , 443955]

486|P49354(2) [447278 , 216454]

487|Q9C0B1(69) [6914666 , 11256664]

488|Q14994(62) [4034 , 2812]

489|P34981(13) [114750 , 2949854]

490|Q02763(101) [11751922 , 11485656]

491|P02689(3) [15433 , 165339]

492|P54577(10) [10180201 , 185915]

493|P05093(134) [132971 , 5994]

494|P98170(130) [49836020 , 46940575]

495|P02766(134) [1369 , 46883536]

496|P31040(59) [4462 , 21307]

497|Q9Y2I1(15) [199 , 208820]

498|Q9BYF1(85) [11238823 , 448281]

499|P63208(9) [24768548 , 24768549]

500|P37059(26) [3503 , 10114]

501|P30405(22) [46883536 , 8189]

502|P49366(15) [17134 , 5288371]

503|K9J956(0) [11223423 , 5282340]

504|O60885(63) [16722836 , 11364421]

505|Q9HAB3(74) [23663870 , 10413]

506|P37088(46) [5546 , 16231]

507|P53609(2) [216454 , 447278]

508|Q9UKQ2(16) [449500 , 17754166]

509|P04818(174) [60953 , 104758]

510|P10619(38) [3010818 , 10324367]

511|P09238(61) [448002 , 119031]

512|P40238(73) [9852519 , 9846180]

513|Q9UJM8(3) [676157 , 11915]

514|Q13563(54) [16231 , 934]

515|P62942(16) [445643 , 5284616]

516|Q9Y3R4(0) [2950 , 10168]

517|Q6IB77(50) [12717 , 1088]

518|O60882(9) [448002 , 119031]

519|P25116(121) [10459564 , 10077130]

520|P04271(159) [42608445 , 2823014]

521|O00214(11) [119138 , 101798]

522|Q9UIG8(8) [5280360 , 5311236]

523|Q04609(53) [6102781 , 44306373]

524|P02741(317) [1014 , 39042]

525|P11836(130) [337359 , 5478883]

526|P51452(6) [5480 , 10212]

527|P54855(17) [2913 , 1057]

528|P15086(12) [11705956 , 23653503]

529|Q96QZ0(1) [3371 , 636403]

530|O15382(4) [1053 , 1051]

531|P35610(126) [166558 , 4912]

532|P35557(71) [46908929 , 39562]

533|Q16613(8) [446811 , 446814]

534|Q13126(66) [10041129 , 188350]

535|P05186(61) [5478883 , 26879]

536|P14091(14) [24800541 , 16126898]

537|Q12908(24) [53492727 , 10133]

538|Q13093(58) [9939609 , 9918381]

539|Q9Y2Q3(182) [5326960 , 6]

540|P30531(11) [9860294 , 60648]

541|P03952(20) [24800541 , 204102]

542|P17538(4) [17754112 , 65389]

543|Q401N2(21) [32051 , 6000]

544|Q14145(58) [246835 , 3108]

545|Q9Y4D2(0) [3034010 , 3572]

546|P50440(14) [12717 , 1088]

547|P00747(480) [564 , 5526]

548|P15085(9) [16741275 , 24768537]

549|Q8IWU9(30) [4652 , 3337]

550|P61073(340) [65015 , 11151928]

551|Q9UL51(13) [65637 , 9869877]

552|P41250(19) [1088 , 12717]

553|P16444(28) [6435415 , 104838]

554|O14939(26) [10198924 , 445639]

555|P61160(18) [447966 , 588963]

556|P00505(36) [447742 , 1051]

557|O95665(2) [54385 , 119192]

558|P23434(10) [12717 , 1088]

559|Q07817(142) [24978538 , 12597]

560|Q13133(35) [2758 , 16734800]

561|Q13332(7) [2088 , 3305]

562|O00182(50) [101798 , 119138]

563|P63165(43) [3151041 , 54676538]

564|Q08493(3) [3671 , 5092]

565|Q9NY46(5) [216327 , 11967800]

566|P30989(46) [119192 , 44623946]

567|O14924(9) [3351 , 10219]

568|Q9HAW9(17) [10894 , 21138]

569|O95718(38) [448537 , 5280961]

570|P25098(54) [1973720 , 1870615]

571|Q96RD7(16) [3371 , 636403]

572|Q8TEK3(19) [41214 , 57345410]

573|P55211(98) [24800541 , 42601552]

574|Q969I3(0) [12717 , 1088]

575|P21673(58) [5494411 , 446425]

576|P08100(48) [15433 , 5414]

577|Q5VSF9(0) [199 , 11622909]

578|Q06187(178) [24821094 , 59174488]

579|Q12884(65) [44513473 , 11516136]

580|P55157(105) [5745206 , 9853053]

581|P30872(36) [9941444 , 6918265]

582|P35080(7) [70931 , 8189]

583|P16435(54) [10331844 , 4493]

584|P55212(27) [12000240 , 42601552]

585|P61626(61) [1031 , 13373305]

586|Q15761(11) [11491176 , 20629114]

587|O00408(8) [24316 , 4197]

588|P98073(4) [2090 , 54676538]

589|P17931(158) [119138 , 101798]

590|P18505(7) [31304 , 104781]

591|O14727(65) [2090 , 1973720]

592|Q9NP56(3) [3182 , 3108]

593|Q9Y210(54) [444899 , 441298]

594|P34949(28) [5351 , 5320]

595|Q9P0Z9(5) [1088 , 12717]

596|Q9H4B7(9) [11351021 , 11643449]

597|Q14032(31) [1088 , 12717]

598|Q9UBS5(23) [11281011 , 5361323]

599|Q14643(40) [2519 , 5957]

600|P15090(46) [448768 , 448769]

601|O95264(13) [6918107 , 108000]

602|P09603(106) [11485656 , 6450551]

603|Q8TCU5(6) [182137 , 2366]

604|O43525(25) [121892 , 5413]

605|P19793(57) [449171 , 82146]

606|O00443(11) [56649450 , 51001932]

607|Q9Y2R2(146) [1973720 , 3246767]

608|Q9UGI6(18) [6000 , 2993]

609|P35398(28) [65076 , 5997]

610|P15538(29) [4211 , 4174]

611|O43451(18) [444020 , 441314]

612|P05412(136) [16129582 , 114829]

613|O00206(465) [44589052 , 36314]

614|Q7L0J3(6) [9837243 , 9942725]

615|Q12882(94) [16130199 , 43157]

616|Q92953(2) [1727 , 5413]

617|P16662(34) [21138 , 28718]

618|P17752(33) [4652 , 3337]

619|Q8WU03(0) [12717 , 1088]

620|P62937(56) [5284373 , 145742]

621|Q92843(32) [24978538 , 3503]

622|P07900(157) [11955716 , 6505803]

623|P14061(39) [10114 , 3503]

624|Q16515(9) [16231 , 4413]

625|Q9UBL9(21) [9812416 , 2265]

626|Q9NSA2(0) [1727 , 5413]

627|Q9UBR2(19) [24800541 , 42601552]

628|Q9NY47(15) [3446 , 3333]

629|Q96RD6(7) [3371 , 636403]

630|O94760(27) [107984 , 132862]

631|P13726(190) [6540268 , 4261]

632|P04118(2) [446977 , 5414]

633|P53634(41) [24901101 , 13186895]

634|Q09428(91) [3478 , 65981]

635|O60658(3) [3108 , 3758]

636|Q9UM07(60) [46937075 , 159772]

637|P42892(63) [24800541 , 3038505]

638|P55263(28) [11987868 , 216210]

639|Q96GD0(45) [1050 , 1051]

640|Q92959(22) [5280360 , 1005]

641|Q9HAW7(49) [2361 , 11790 , 2950]

642|Q9UKL4(8) [3371 , 957 , 636403]

643|Q5T442(19) [636403 , 957 , 3371]

644|Q9H598(4) [5665 , 1088 , 12717]

645|Q9NTQ9(9) [957 , 636403 , 3371]

646|P42226(97) [10219 , 2179 , 2391]

647|Q96S37(13) [255968 , 5342 , 2333]

648|Q9BPV8(3) [9854012 , 5361 , 5957]

649|P05108(52) [2812 , 4211 , 2145]

650|O76082(49) [107738 , 19003 , 10219]

651|Q16602(15) [11319053 , 51049968 , 25019940]

652|P09210(15) [2478 , 1547484 , 2179]

653|Q07343(19) [449193 , 5092 , 3671]

654|P36383(8) [957 , 636403 , 3371]

655|Q7RTT9(3) [2756 , 2520 , 441074]

656|Q96QT4(47) [5957 , 4413 , 6076]

657|P23378(16) [12717 , 1088 , 1051]

658|P35558(21) [447371 , 1005 , 447076]

659|P0DP25(120) [3333 , 16362 , 3389]

660|Q9BYV1(7) [12717 , 1051 , 1088]

661|P06858(148) [2796 , 4670 , 3463]

662|P36382(36) [3371 , 636403 , 957]

663|P35212(28) [636403 , 957 , 3371]

664|P17302(200) [957 , 636403 , 3371]

665|V9HW50(0) [347402 , 3406 , 77022]

666|Q96KN9(0) [3371 , 957 , 636403]

667|Q15125(87) [2467 , 3559 , 65638]

668|Q9P1Z3(2) [9869877 , 132999 , 65637]

669|P19634(59) [9868115 , 16231 , 151172]

670|P48165(40) [957 , 3371 , 636403]

671|Q14831(9) [33032 , 68841 , 446355]

672|P55055(54) [16734800 , 12057 , 2758]

673|P57789(6) [3562 , 5070 , 444899]

674|O95069(27) [444899 , 3562 , 5070]

675|Q99527(44) [5757 , 104741 , 2733526]

676|P43004(38) [52941485 , 11412540 , 107883]

677|P17252(77) [153999 , 6918670 , 176167]

678|Q9NR96(215) [2090 , 3652 , 12449]

679|Q9UP38(22) [16779 , 31475 , 14868]

680|P06870(64) [204102 , 107706 , 6540268]

681|P06881(157) [6918509 , 25019940 , 132412]

682|P08034(68) [3371 , 636403 , 957]

683|O00222(15) [68841 , 33032 , 213056]

684|P00439(51) [44257 , 146719 , 4652]

685|O76083(5) [5722 , 447108 , 3758]

686|P09871(13) [5329098 , 5212 , 4369524]

687|P10415(544) [4021 , 24978538 , 12597]

688|Q9GZZ6(0) [101616 , 89594 , 441071]

689|O15440(29) [5722 , 60871 , 3108]

690|O94992(17) [3616 , 1752606 , 2847505]

691|O95452(63) [636403 , 3371 , 957]

692|Q13946(15) [3758 , 3182 , 44591583]

693|Q8IXJ6(36) [168120 , 2396 , 24756910]

694|P49327(81) [1046 , 11852 , 7329]

695|P49116(31) [11751922 , 445354 , 444795]

696|Q71U36(25) [4030 , 11351021 , 2082]

697|Q5TD07(0) [132127 , 89105 , 216210]

698|O60741(23) [9869877 , 65637 , 132999]

699|Q5XKG2(0) [10036135 , 126569 , 10112]

700|P29033(127) [3371 , 636403 , 957]

701|O00519(37) [4943 , 3000715 , 24771824]

702|Q15596(40) [11149479 , 445920 , 11368987]

703|Q9NQS5(2) [8180 , 2969 , 3893]

704|P07202(91) [1349907 , 657298 , 31072]

705|Q9NYB5(4) [6675 , 5311236 , 4911]

706|P48147(16) [25155578 , 122623 , 10096344]

707|Q14790(179) [42601552 , 12000240 , 24800541]

708|Q9Y239(63) [51349607 , 51349463 , 51349461]

709|P23786(64) [21109 , 13671153 , 11811445]

710|O15427(25) [10112 , 3758 , 10413]

711|Q9Y6H8(8) [3371 , 957 , 636403]

712|P35499(43) [11967800 , 216327 , 4178]

713|Q969S8(8) [5311 , 11538455 , 2466]

714|P14735(25) [2016 , 10909430 , 4343310]

715|O15303(8) [33032 , 68841 , 213056]

716|Q8N144(2) [957 , 636403 , 3371]

717|Q96NT5(18) [130731 , 126941 , 3715]

718|Q99250(37) [216327 , 1775 , 11967800]

719|P41440(73) [130731 , 104758 , 126941]

720|P02144(41) [79124 , 11646 , 79084]

721|P07327(17) [5287890 , 3406 , 77022]

722|Q9NSA0(5) [10258 , 11948288 , 8820]

723|O95377(11) [3371 , 636403 , 957]

724|P35523(21) [64929 , 4488 , 32051]

725|O43614(10) [25195495 , 24965990 , 25128145]

726|P09923(36) [72139 , 3243850 , 2327]

727|Q03013(16) [17753973 , 447108 , 6]

728|P06239(78) [5494449 , 3062316 , 10302451]

729|Q8TDU6(30) [222528 , 10133 , 64971]

730|P20711(45) [2327 , 34359 , 38853]

731|Q9BXC0(4) [10413 , 61503 , 938]

732|P09172(47) [3117 , 9796181 , 1024]

733|O75600(0) [12717 , 1088 , 1051]

734|Q00653(73) [16739648 , 5359476 , 237]

735|P22557(33) [1051 , 1088 , 12717]

736|P48449(10) [1949 , 15433 , 445987]

737|Q9NR97(59) [57469 , 46241268 , 159603]

738|P28472(27) [3277 , 104781 , 31304]

739|P31930(2) [3034285 , 4064 , 10972974]

740|Q16773(2) [676157 , 1051 , 1053]

741|Q9NZQ8(8) [1103 , 3371 , 77999]

742|Q9Y3Q4(9) [65637 , 9869877 , 132999]

743|P31391(52) [6918265 , 2247 , 16129681]

744|P00740(95) [204102 , 11641515 , 1746]

745|P16455(179) [4564 , 3025944 , 4578]

746|Q96P20(189) [2081 , 1720828 , 26695]

747|Q969M2(0) [957 , 636403 , 3371]

748|Q9Y345(16) [12717 , 1088 , 24946690]

749|O75712(30) [957 , 636403 , 3371]

750|P54219(13) [3822 , 6018 , 5770]

751|P20618(5) [11347535 , 25183872 , 11556711]

752|A6NN92(0) [957 , 3371 , 636403]

753|Q9UGM1(4) [441071 , 89594 , 101616]

754|Q05586(44) [71077 , 22880 , 750]

755|Q6PEY0(1) [636403 , 957 , 3371]

756|Q9Y251(150) [2247 , 25194 , 10621]

757|P00492(90) [2265 , 1349907 , 100684]

758|P13612(46) [119 , 11563636 , 9935681]

759|Q14416(16) [446355 , 33032 , 213056]

760|Q9NPC1(21) [9823886 , 5280724 , 5280492]

761|Q9NQA5(13) [888 , 4189 , 3198]

762|Q16099(5) [10036135 , 107883 , 5282253]

763|Q8NFK1(2) [957 , 3371 , 636403]

764|P05121(400) [10224267 , 6450819 , 46883536]

765|P57773(0) [3371 , 636403 , 957]

766|Q8N695(29) [3825 , 3342 , 3672]

767|P07384(34) [6857711 , 42601552 , 24800541]

768|Q15046(12) [447966 , 10344820 , 11213558]

769|Q8IUN5(0) [6307 , 2732 , 3647]

770|Q92698(18) [178144 , 2179 , 327045]

771|Q7Z2H8(5) [439280 , 3744 , 6305]

772|P56373(23) [2265 , 9812416 , 1051]

773|P14679(88) [1057 , 7638 , 9015]

774|Q92952(2) [2993 , 10237 , 6000 , 44623946]

775|P46721(14) [6324616 , 10133 , 11967809 , 5311236]

776|Q00534(84) [5330286 , 448008 , 46220502 , 11285002]

777|P22607(188) [9809715 , 53235510 , 11154925 , 51039095]

778|Q01538(5) [208908 , 2396 , 151194 , 176870]

779|Q7RTX7(0) [60663 , 5280723 , 5280360 , 5994]

780|P48443(18) [3312 , 108143 , 82146 , 449171]

781|Q86XQ3(0) [5994 , 5280723 , 60663 , 5280360]

782|P12268(7) [37542 , 5281078 , 2723601 , 446541]

783|P02763(14) [54680692 , 2997 , 72106 , 54676537]

784|Q96P56(2) [5280360 , 5280723 , 60663 , 5994]

785|P31350(33) [60750 , 3657 , 657237 , 119182]

786|Q15077(11) [145729 , 6133 , 161647 , 6031]

787|Q15413(17) [6914273 , 888 , 2519 , 5957]

788|P14920(13) [234556 , 10531257 , 10531300 , 12473]

789|P48067(7) [1088 , 24946690 , 235905 , 12717]

790|Q92847(64) [11526696 , 6918297 , 9828911 , 178024]

791|Q9NR82(8) [3932 , 5413 , 121892 , 214350]

792|Q9NY33(1) [11500899 , 11493219 , 11573427 , 11516136]

793|P11413(133) [13752 , 612424 , 3117 , 12449]

794|P02708(29) [3450 , 5314 , 4032 , 441289]

795|Q15722(37) [11508736 , 5280492 , 9823886 , 177941]

796|P48048(37) [1989 , 2368 , 3478 , 199]

797|P80404(12) [5950 , 1060 , 1051 , 5665]

798|Q13304(6) [18068 , 5957 , 6031 , 8629]

799|P21462(22) [5284373 , 222528 , 5342 , 10133]

800|P04798(249) [16007088 , 2361 , 11790 , 3404]

801|P51788(21) [157920 , 4594 , 32051 , 444899]

802|P00813(109) [439693 , 20279 , 3108 , 657237]

803|P68371(14) [2082 , 4030 , 4622 , 11351021]

804|P09382(122) [101798 , 119138 , 11953346 , 1567]

805|P46059(12) [19003 , 44560294 , 65526 , 12897341]

806|Q9HAW8(16) [2361 , 21138 , 11790 , 2950]

807|Q99835(57) [25027363 , 24775005 , 49848070 , 24776445]

808|Q5NUL3(12) [11005 , 24857286 , 5280934 , 445639]

809|P20815(110) [4506 , 208908 , 115237 , 16072188]

810|Q16478(2) [167842 , 5282253 , 10036135 , 126569]

811|P55011(38) [4849 , 3440 , 5839 , 2471]

812|Q8N1Q1(14) [11967800 , 6852128 , 3295 , 36811]

813|P16066(25) [10026 , 4510 , 213037 , 16132416]

814|Q14534(11) [47641 , 5510 , 1549008 , 2484]

815|Q8NEC5(1) [5280360 , 5280723 , 60663 , 5994]

816|P49768(105) [49867930 , 11269353 , 9843750 , 46883536]

817|P50135(31) [2165 , 216210 , 24466 , 5227]

818|O60911(9) [46228924 , 24901101 , 13186895 , 10324367]

819|P16233(9) [446977 , 5414 , 3034010 , 23724878]

820|P21802(428) [51039095 , 37720 , 9809715 , 53235510]

821|O15439(64) [10133 , 2333 , 5722 , 3108]

822|P08263(62) [2179 , 15906 , 165339 , 2478]

823|P04070(45) [204102 , 122267 , 216210 , 183797]

824|P11597(91) [159325 , 49836058 , 11556427 , 6918540]

825|Q99808(30) [3074 , 3108 , 60849 , 3926]

826|P23921(47) [60750 , 119182 , 3657 , 657237]

827|P51787(80) [3081185 , 3702 , 4044 , 4488]

828|Q9NYA1(81) [448008 , 107970 , 44158029 , 44158028]

829|O43193(2) [447043 , 15984937 , 9918079 , 12560]

830|P34896(60) [12717 , 1088 , 1051 , 445062]

831|P34998(134) [9884366 , 11596613 , 11223423 , 5282340]

832|Q03426(51) [447277 , 445713 , 1195 , 445995]

833|Q7Z2W7(36) [644019 , 16666 , 16078 , 2812]

834|O43613(10) [24965990 , 25128145 , 2743305 , 25195495]

835|P49721(4) [25183872 , 11556711 , 11347535 , 24800541]

836|Q96DB2(11) [9865515 , 5311 , 11538455 , 10309899]

837|P03956(310) [9824350 , 5362422 , 119031 , 466151]

838|P19099(75) [2540 , 12229396 , 132971 , 4174 , 44139752]

839|P32745(21) [2247 , 16129681 , 5311430 , 9941444 , 71349]

840|Q16322(3) [5413 , 2520 , 1727 , 3823 , 16362]

841|P28074(6) [11556711 , 11347535 , 24800541 , 25183872 , 387447]

842|P53778(9) [10341154 , 3038525 , 10297982 , 46883775 , 156422]

843|Q9NZK7(2) [155815 , 5321765 , 23640750 , 175540 , 447739]

844|Q14330(3) [12717 , 644019 , 104850 , 1088 , 16078]

845|P11511(142) [60198 , 2145 , 2187 , 3902 , 13769]

846|Q8TD43(17) [5957 , 1103 , 6083 , 60961 , 2812]

847|Q15391(3) [8629 , 445675 , 6031 , 17473 , 18068]

848|O95342(47) [104865 , 53461739 , 2794 , 10133 , 4912]

849|S4R453(0) [3639 , 2343 , 3647 , 6307 , 2315]

850|Q9BQB6(51) [4760 , 54676038 , 54680692 , 54676537 , 449171]

851|P35346(29) [16129681 , 5311430 , 71349 , 9941444 , 2247]

852|O15554(17) [5070 , 4507 , 2812 , 216327 , 2733]

853|P68871(110) [2540 , 122335 , 46937073 , 2265 , 44129642]

854|P21964(201) [5281081 , 9838389 , 36811 , 4369285 , 4659569]

855|O60840(31) [3784 , 1547484 , 153994 , 208898 , 4485]

856|Q9UL62(6) [77999 , 5994 , 2726 , 3371 , 5280961]

857|P06737(15) [46936633 , 1175 , 1051 , 445383 , 9547901]

858|Q14654(63) [3033825 , 4201 , 5505 , 47528 , 3488]

859|Q15858(63) [56959 , 129228 , 216327 , 219078 , 11967800]

860|P05089(75) [123895 , 657085 , 23644529 , 446122 , 5287648]

861|P46663(35) [71364 , 16102897 , 439201 , 11498853 , 11953367]

862|P06493(72) [5005498 , 11285002 , 5289419 , 2856 , 46926350]

863|O75311(2) [4485 , 32051 , 441071 , 31304 , 16078]

864|O43526(27) [53276 , 3932 , 214350 , 121892 , 5413]

865|P52732(11) [44224257 , 25167017 , 10368812 , 3503 , 6851740]

866|P78348(17) [16231 , 32051 , 4413 , 3672 , 934]

867|P47900(30) [159296 , 5957 , 440141 , 5361 , 44623946]

868|Q01668(27) [2162 , 2520 , 4507 , 4485 , 3784]

869|O60760(225) [2737071 , 4122 , 24764436 , 6540277 , 121026]

870|Q9GZU7(9) [1973720 , 3151041 , 265580 , 10621 , 21109]

871|Q14973(18) [158781 , 3749 , 5284373 , 21138 , 10133]

872|Q96G91(4) [15993 , 5361 , 6133 , 5957 , 5892]

873|P20839(31) [446541 , 37542 , 2265 , 5281078 , 2723601]

874|P24530(129) [216235 , 16004692 , 6918493 , 178103 , 104865]

875|Q92523(10) [10917 , 13671153 , 4746 , 21109 , 11811445]

876|P42263(30) [126569 , 2196 , 2910 , 4843 , 127894]

877|Q96RP8(1) [2520 , 2157 , 3356 , 441074 , 5413]

878|P41180(156) [158797 , 10345214 , 1103 , 156419 , 6305]

879|P51843(107) [72462 , 107782 , 4122 , 42725 , 35803]

880|P04278(110) [102146 , 10633 , 44450327 , 19582 , 27812]

881|P00918(65) [5284549 , 3284 , 2720 , 2343 , 2910]

882|P48058(8) [126569 , 4843 , 2196 , 2910 , 127894]

883|P56696(15) [121892 , 3932 , 2351 , 5413 , 214350]

884|Q9GZV3(5) [19646 , 36811 , 249 , 10198924 , 187]

885|Q5XXA6(33) [4488 , 3386 , 16129778 , 60663 , 3371]

886|Q9P0L9(17) [16231 , 3371 , 313 , 525 , 311]

887|P01584(709) [54675783 , 61635 , 123600 , 10341154 , 3671]

888|Q9H0H5(14) [54676538 , 11293 , 2016 , 12449 , 54675783]

889|P07101(85) [6140 , 6057 , 3125 , 441350 , 44257]

890|P33316(8) [11536503 , 145729 , 11680992 , 11637769 , 554527]

891|P50052(90) [11238823 , 172198 , 2540 , 9825285 , 10257882]

892|P39086(8) [447196 , 10036135 , 5282253 , 5284627 , 127894]

893|P10826(81) [444795 , 5289501 , 5381 , 60164 , 449171]

894|P35869(166) [3117 , 2750 , 2361 , 41684 , 21307]

895|P32239(41) [16129675 , 9870520 , 4506 , 108187 , 3960]

896|P62508(11) [5280961 , 11742 , 448537 , 5284643 , 9549223]

897|P08709(118) [183797 , 204102 , 1792 , 42601552 , 24800541]

898|O43497(13) [16362 , 5576 , 1547484 , 11967800 , 60663]

899|Q00975(12) [5282138 , 199 , 2950 , 16135415 , 5745207]

900|Q9Y2T6(12) [16078 , 2391 , 4671 , 644019 , 104850]

901|P09668(26) [42601552 , 24800541 , 44398651 , 44398659 , 13186895 , 24901101]

902|Q8TCC7(13) [123979 , 10258 , 148200 , 11948288 , 1175 , 44814423]

903|P49019(23) [56950369 , 9060 , 16098 , 938 , 51576 , 71567]

904|Q05940(27) [5770 , 3822 , 22297 , 6018 , 101616 , 1615]

905|Q13936(32) [4485 , 2520 , 39186 , 4499 , 60663 , 60753]

906|P40763(375) [400769 , 131411 , 5284513 , 16659841 , 2750 , 129869]

907|P42261(21) [2910 , 2196 , 4843 , 4118151 , 127894 , 126569]

908|P41594(29) [9926832 , 15585158 , 162834 , 44557636 , 5042 , 129228]

909|Q9Y243(51) [43860 , 25227436 , 176167 , 148177 , 24788740 , 6918736]

910|Q16850(7) [5335 , 441383 , 43233 , 2812 , 1349907 , 5510]

911|P10276(88) [444795 , 449171 , 108143 , 5289501 , 5381 , 60164]

912|Q9HC97(6) [54676038 , 2391 , 3440 , 2471 , 5722 , 3845]

913|P49286(23) [10220503 , 10305301 , 896 , 115348 , 82148 , 208902]

914|P20648(40) [3883 , 4594 , 9578005 , 4679 , 9568614 , 5029]

915|Q12791(84) [2733 , 216327 , 5757 , 214350 , 5413 , 37393]

916|Q92736(57) [888 , 2519 , 6914273 , 4914 , 5361 , 5957]

917|O15496(47) [53323583 , 53319614 , 51346870 , 25166397 , 10067704 , 155815]

918|P14902(73) [3505109 , 69590 , 405012 , 6305 , 11979 , 11122]

919|P42336(403) [16654980 , 3973 , 49784945 , 10427712 , 51001932 , 50905713]

920|Q99816(31) [903966 , 2016 , 13791 , 10168 , 4343310 , 3108]

921|P00326(77) [1048 , 77022 , 13017 , 3406 , 11815987 , 17429]

922|P15559(164) [68238 , 89105 , 2361 , 394347 , 54676038 , 5813717]

923|P50281(151) [42601552 , 44383797 , 448002 , 24768528 , 119031 , 9933197]

924|P00519(120) [24826799 , 5291 , 10302451 , 5328940 , 644241 , 3062316]

925|P10827(44) [9863447 , 5819 , 5803 , 8730 , 5920 , 9862248]

926|P21817(70) [6914273 , 5361 , 2519 , 5957 , 4914 , 888]

927|Q15842(17) [4201 , 3488 , 32778 , 3033825 , 5505 , 443423]

928|Q8TDV5(12) [5283468 , 4671 , 27902 , 5311093 , 11691484 , 56971]

929|P36544(24) [46196517 , 9930121 , 25147644 , 60854 , 4032 , 9794392]

930|P03951(23) [11304895 , 183797 , 6852140 , 204102 , 6852141 , 6857702]

931|Q9UNI1(7) [446501 , 10324367 , 24820112 , 4634717 , 444650 , 46228924]

932|O15552(9) [1032 , 24857286 , 264 , 176 , 6590 , 7991]

933|Q9NRA0(28) [219100 , 44158028 , 448008 , 15604015 , 107970 , 44158029]

934|Q9Y5Y9(30) [5411 , 56959 , 3610 , 5467 , 7176 , 10770]

935|O14843(0) [7991 , 24857286 , 1032 , 176 , 6590 , 264]

936|Q9NS75(19) [4887 , 177941 , 5717 , 60842 , 6913104 , 11508736]

937|Q9HCF6(9) [5591 , 77999 , 4044 , 4485 , 4829 , 5280335]

938|Q9UBX1(10) [46937105 , 24901101 , 5289258 , 13186895 , 10324367 , 46228924]

939|Q15661(14) [11840929 , 3447 , 37126 , 657077 , 4413 , 159772]

940|Q14832(19) [446355 , 9834591 , 213056 , 33032 , 5042 , 188803]

941|P11217(24) [446801 , 124823 , 1051 , 657140 , 445723 , 445722]

942|O95259(40) [3559 , 71329 , 3696 , 2247 , 2798 , 441074 , 5405]

943|Q8TF76(0) [2856 , 2396 , 3973 , 5005498 , 9549303 , 6419766 , 447966]

944|Q86V90(0) [11967800 , 66553195 , 52195 , 2893 , 7699 , 5497 , 10770]

945|P56817(26) [24748050 , 3699841 , 24748051 , 449218 , 44251605 , 25134249 , 49837968]

946|Q9Y233(17) [11610553 , 3108 , 5722 , 204105 , 44141871 , 11581936 , 10239]

947|P48167(6) [1088 , 656665 , 4485 , 12717 , 32051 , 31304 , 10303232]

948|P51582(3) [161647 , 6830 , 5957 , 145729 , 6133 , 148197 , 9875516]

949|Q15788(30) [656952 , 11395145 , 10274777 , 10286462 , 6102690 , 6102691 , 656953]

950|Q06432(0) [65866 , 2162 , 39186 , 4507 , 4485 , 3784 , 2950]

951|P00742(86) [11634458 , 10182969 , 4581 , 10280735 , 5496659 , 9875401 , 11641515]

952|P50579(25) [1069140 , 2082 , 4030 , 19910 , 3334 , 6855 , 23647763]

953|P21554(97) [104850 , 2543 , 9826744 , 6398473 , 10278470 , 16078 , 11226090]

954|Q9UBY5(26) [11568387 , 11625765 , 44394293 , 44407394 , 10051843 , 10322404 , 44407386]

955|P29371(30) [219077 , 5311424 , 44623946 , 10328936 , 104974 , 23649245 , 133090]

956|P13569(192) [1548943 , 3488 , 4497 , 5280961 , 16220172 , 3333 , 5280443]

957|Q06278(10) [43157 , 2435 , 3108 , 2179 , 3324 , 11967800 , 4055]

958|P23458(87) [44205240 , 16722836 , 25126798 , 46216796 , 25062766 , 16659841 , 46866319]

959|Q04206(155) [114829 , 16109598 , 9820526 , 127864 , 16109538 , 16109600 , 10181390]

960|Q13698(26) [39186 , 4485 , 3333 , 2520 , 153994 , 208898 , 1547484]

961|P00797(228) [24800541 , 6324659 , 44317193 , 44251605 , 44345978 , 16126898 , 5493444]

962|Q07820(124) [3503 , 24978538 , 54675783 , 722193 , 12597 , 1780 , 141870]

963|P11926(90) [445062 , 123865 , 1051 , 1055 , 3009 , 446425 , 1045]

964|Q92633(43) [10322404 , 44394293 , 44407386 , 44407394 , 11625765 , 10051843 , 11568387]

965|Q86VL8(0) [3108 , 119570 , 16362 , 3151 , 2756 , 216210 , 10531]

966|P23415(18) [31304 , 16078 , 4485 , 32051 , 656665 , 6167 , 441071]

967|Q9NYK1(95) [159603 , 12620 , 46241268 , 10309114 , 57469 , 60737 , 3652]

968|Q9HBW0(67) [62532 , 44407394 , 11625765 , 10322404 , 44394293 , 44407386 , 11568387]

969|P49841(96) [6419766 , 5005498 , 11285002 , 11313622 , 176167 , 2856 , 5326739]

970|P30874(63) [56237 , 448601 , 5311430 , 16129681 , 71349 , 2247 , 9941444]

971|P53582(2) [4030 , 2740174 , 3334 , 19910 , 1069140 , 2082 , 6855]

972|P78527(67) [16203712 , 3973 , 16204163 , 51001932 , 16204164 , 16203710 , 16204165]

973|O60755(1) [1878823 , 1993 , 187 , 108144 , 1599306 , 31729 , 1568843]

974|P35498(65) [208898 , 216327 , 4506 , 4753 , 11967800 , 5734 , 5284583]

975|P51681(256) [3002977 , 11614352 , 49871007 , 3001322 , 9574343 , 11285792 , 3009355]

976|Q9UHC3(13) [69590 , 32051 , 199 , 338 , 16231 , 4413 , 3033 , 2244]

977|Q9BYP7(2) [447966 , 5005498 , 9549303 , 3973 , 2856 , 6419766 , 11712649 , 2396]

978|O95180(11) [60663 , 1547484 , 3333 , 941361 , 2200 , 11967800 , 934 , 516892]

979|Q9H244(38) [5957 , 11273179 , 9854012 , 60606 , 6918456 , 9871419 , 5472 , 16066663]

980|Q9Y3S1(12) [6419766 , 5005498 , 3973 , 447966 , 2396 , 2856 , 9549303 , 11712649]

981|P78536(109) [11452716 , 11402671 , 24768528 , 24800541 , 42601552 , 119031 , 448002 , 23627203]

982|P48050(5) [1103 , 1045 , 888 , 444899 , 54683953 , 1102 , 5413 , 32051]

983|Q9UNQ0(171) [2950 , 119373 , 151115 , 176870 , 6063342 , 148201 , 2361 , 11790]

984|Q9UN88(9) [4506 , 2789 , 2441 , 3261 , 31304 , 104781 , 2170 , 31640]

985|P41231(38) [6133 , 145729 , 5361 , 44623946 , 161647 , 148197 , 9875516 , 5957]

986|P01375(1069) [216326 , 40632 , 6918412 , 5639 , 4413 , 3083542 , 5426 , 4740]

987|Q13822(38) [44407394 , 44394293 , 4031 , 13211563 , 11568387 , 10322404 , 11625765 , 44407386]

988|P34947(28) [5005498 , 6419766 , 11712649 , 3973 , 9549303 , 2396 , 447966 , 2856]

989|P48169(6) [3448 , 10237 , 32051 , 4266 , 3373 , 31304 , 107926 , 104781]

990|Q86TI2(12) [10376704 , 11516136 , 44513473 , 11573427 , 11493219 , 11949652 , 10932707 , 11500899]

991|P23416(2) [6167 , 31304 , 32051 , 656665 , 1088 , 441071 , 16078 , 12717]

992|O14842(11) [11005 , 445639 , 121871 , 77999 , 445580 , 5280934 , 985 , 24857286]

993|P55017(60) [6307 , 2315 , 2910 , 2343 , 3639 , 4170 , 2720 , 4870]

994|Q5T6X5(12) [750 , 5950 , 9750 , 5962 , 6322 , 5951 , 6262 , 5961]

995|O94759(21) [444899 , 784 , 5892 , 32051 , 3371 , 2812 , 4189 , 3198]

996|Q9HBX9(10) [65599 , 3324 , 1473386 , 4118928 , 35802 , 210320 , 824727 , 2844395]

997|Q6V1X1(9) [11516136 , 11500899 , 11573427 , 11949652 , 11493219 , 44513473 , 10932707 , 10376704]

998|P08473(121) [5362417 , 24800541 , 656629 , 3038505 , 443380 , 4369380 , 1234 , 42601552]

999|Q96FL8(5) [2756 , 10531 , 216210 , 4993 , 3151 , 2749 , 3108 , 16362 , 2247]

1000|O43781(2) [160355 , 3540 , 6918454 , 153999 , 5005498 , 3973 , 10172943 , 3078519 , 3542]

1001|P48039(22) [10305301 , 82148 , 44623946 , 10220503 , 208902 , 115348 , 23581869 , 10531 , 896]

1002|Q12879(37) [2130 , 68736 , 22880 , 3821 , 180081 , 888 , 750 , 6468 , 71077]

1003|P42262(21) [4843 , 127894 , 3003157 , 2196 , 10518 , 2910 , 126569 , 10036135 , 167842]

1004|P80365(66) [5060832 , 36054272 , 5289613 , 4432326 , 36054265 , 10114 , 4570352 , 11670435 , 5151632]

1005|P34995(24) [49843471 , 448457 , 5311044 , 5280363 , 5280723 , 5282411 , 5280360 , 45266502 , 5312153]

1006|P39900(115) [448002 , 24800541 , 10565532 , 119031 , 24751752 , 46937107 , 25271580 , 46937106 , 46937108]

1007|Q15118(39) [10267580 , 6918454 , 11712649 , 447966 , 10296883 , 3973 , 24748573 , 448008 , 2396]

1008|P62805(20) [24800541 , 3025986 , 5005498 , 42601552 , 4592 , 11338033 , 448008 , 160355 , 17754027]

1009|Q96RG2(2) [447966 , 3973 , 2396 , 448008 , 2856 , 6419766 , 11712649 , 9549303 , 5005498]

1010|Q13258(17) [11462174 , 448457 , 5280363 , 45270144 , 42641863 , 49843471 , 11508736 , 5280723 , 5280360]

1011|P22310(15) [17100 , 102210 , 121892 , 2913 , 4761 , 5405 , 28718 , 2725 , 11230]

1012|P18054(52) [3610 , 3503 , 4493 , 1780 , 10168 , 3698 , 107715 , 3117 , 19910]

1013|P13631(27) [3312 , 5289501 , 60164 , 9887303 , 449171 , 444795 , 108143 , 5381 , 2605]

1014|P09619(154) [5329102 , 9809715 , 25031915 , 10366136 , 151194 , 5329099 , 11485656 , 9933475 , 10074640]

1015|P09237(155) [128564 , 44302022 , 119031 , 42601552 , 10565532 , 9933197 , 466151 , 5362422 , 24800541]

1016|Q13370(24) [3698 , 3758 , 17754438 , 110635 , 5663 , 24316 , 4197 , 2753 , 2754]

1017|P21397(67) [4235 , 3675 , 68802 , 3759 , 26757 , 4380 , 10192617 , 5530 , 60824]

1018|Q9UHL4(0) [23646087 , 11516136 , 44513473 , 11573427 , 44387758 , 11500899 , 10096344 , 23646100 , 11493219]

1019|P43250(9) [5005498 , 447966 , 2856 , 9549303 , 6419766 , 3973 , 2396 , 11712649 , 448008]

1020|P00374(103) [46883536 , 21109 , 126941 , 446753 , 448810 , 104758 , 446752 , 148121 , 5583]

1021|Q12809(74) [71329 , 3114 , 2157 , 60753 , 4932 , 2247 , 3081185 , 2769 , 5405]

1022|P32297(25) [10235 , 10517 , 10176764 , 3389 , 4032 , 115237 , 1615 , 3559 , 3604]

1023|O15399(3) [71077 , 888 , 22880 , 2130 , 6468 , 3821 , 750 , 68736 , 180081]

1024|Q5TEH5(0) [24800541 , 204102 , 447733 , 152951 , 10324367 , 447732 , 42601552 , 46228924 , 11641515 , 183797]

1025|P05023(16) [8612 , 5359268 , 441207 , 3647 , 2749 , 33887 , 6437380 , 6604423 , 28620 , 2724385]

1026|P55210(47) [24800541 , 448573 , 42601552 , 2179 , 24144 , 12000240 , 10219 , 3108 , 10168 , 3503]

1027|Q4U2R8(10) [11948288 , 4911 , 3639 , 10258 , 2333 , 44814423 , 181976 , 148200 , 123979 , 1175]

1028|P43088(19) [5282226 , 5311100 , 9868491 , 5312153 , 5311221 , 5280360 , 5311027 , 11508736 , 448457 , 5280363]

1029|P09960(31) [24768560 , 44129621 , 1082702 , 7023019 , 22690393 , 2776332 , 44129624 , 44129620 , 15547703 , 22692237]

1030|P42574(291) [24800541 , 12000240 , 42601552 , 44395211 , 54675783 , 207112 , 447400 , 46937133 , 44395477 , 46937089]

1031|P43116(55) [5282381 , 5280363 , 25114442 , 11508736 , 18376177 , 5280723 , 5280360 , 9890801 , 49843471 , 448457]

1032|P43119(16) [11508736 , 45270144 , 5282415 , 5311044 , 5311243 , 6918140 , 5282381 , 11462174 , 5280723 , 10501053]

1033|Q96RJ0(2) [1614 , 36303 , 2366 , 1615 , 4581 , 5610 , 5826 , 1001 , 32893 , 2200]

1034|P43681(50) [5310966 , 9824145 , 5310967 , 10131048 , 170361 , 5850 , 3604 , 4032 , 25147644 , 23576]

1035|P30559(24) [5311010 , 9895468 , 644077 , 11634973 , 53330936 , 11340891 , 172997 , 439302 , 60943 , 14257660]

1036|Q9H228(4) [44394248 , 44394116 , 44394169 , 44394220 , 44344193 , 44394117 , 44394149 , 107970 , 44394161 , 44394247]

1037|P78508(37) [3478 , 1989 , 4201 , 5503 , 4543 , 32778 , 91610 , 121891 , 9565 , 2727]

1038|Q9HC16(23) [1552036 , 3244425 , 1811924 , 3781338 , 2082 , 4343310 , 68684 , 12449 , 151506 , 21109]

1039|P47989(60) [1349907 , 5288320 , 1046 , 446425 , 134018 , 65457 , 2094 , 675 , 43157 , 11979]

1040|P31639(18) [11988953 , 10453870 , 9871420 , 44814423 , 9824918 , 5278 , 9887712 , 25195624 , 11949646 , 24812758]

1041|Q9HBH1(1) [5583 , 213043 , 5578 , 159596 , 24466 , 6178111 , 4064 , 148121 , 4993 , 9571037]

1042|P21453(30) [44394248 , 44394116 , 327045 , 44394117 , 44394161 , 44344193 , 107970 , 44394169 , 44394220 , 44394247 , 44394149]

1043|P30518(37) [216237 , 9895468 , 5311010 , 439302 , 644076 , 644077 , 3038506 , 60943 , 119369 , 172997 , 151171]

1044|P21731(34) [123879 , 6918030 , 54343 , 5362391 , 9938840 , 5311100 , 5312138 , 6449876 , 2449 , 5280363 , 5280360]

1045|P07437(24) [5978 , 9854073 , 2082 , 13342 , 36314 , 4030 , 148124 , 6445540 , 40839 , 6167 , 11354606]

1046|O60341(43) [46868080 , 46868074 , 46868078 , 46867800 , 46867937 , 5530 , 46867935 , 46867802 , 46867943 , 46868082 , 46867941]

1047|P00750(161) [204102 , 152951 , 183797 , 6540268 , 42601552 , 11641515 , 564 , 24800541 , 1507 , 445843 , 4474224]

1048|Q9P0X4(2) [8280 , 3291 , 9883933 , 5576 , 11967800 , 2520 , 1547484 , 60663 , 5486971 , 16362 , 6476]

1049|Q494W8(3) [16005981 , 46196517 , 10176607 , 10518 , 3003157 , 101616 , 5475 , 24795080 , 187 , 10888091 , 11151363]

1050|P61925(0) [16122633 , 16122635 , 11175137 , 11314340 , 11608401 , 3540 , 10172943 , 3542 , 12412578 , 11840906 , 16122634]

1051|Q13490(51) [44567560 , 44567555 , 24939290 , 24737642 , 44567563 , 44567572 , 46940575 , 49836020 , 44567564 , 44567571 , 44567568]

1052|Q13621(60) [4121 , 4849 , 3440 , 41781 , 2732 , 2315 , 3278 , 4873 , 5560 , 2471 , 3647]

1053|P37288(9) [72081 , 9895468 , 216237 , 439302 , 3038506 , 60943 , 5311010 , 644077 , 151171 , 119369 , 644076]

1054|P27169(224) [12279383 , 102914 , 11559 , 18303 , 44578434 , 86736 , 559675 , 44578433 , 86852 , 14936438 , 257369 , 13204]

1055|P35236(4) [3647 , 10212 , 16654690 , 16654689 , 16654688 , 13001 , 16217011 , 3246767 , 1973720 , 10168 , 1780 , 16654691]

1056|Q96PF2(3) [76098 , 16122633 , 11608401 , 6419766 , 448008 , 2856 , 11712649 , 2396 , 9549303 , 447966 , 3973 , 5005498]

1057|O15244(33) [199 , 21138 , 3151 , 4342 , 123600 , 216210 , 44814423 , 10531 , 3108 , 16231 , 155774 , 16362]

1058|P11388(82) [2179 , 5362259 , 36462 , 2950 , 3885 , 9865515 , 115150 , 4212 , 72116 , 10180 , 108143 , 31703]

1059|P14555(104) [1372 , 51346870 , 53323583 , 10954847 , 11063341 , 10067704 , 53319614 , 3711 , 25166397 , 3710 , 155815 , 446400]

1060|Q99500(11) [107970 , 44394247 , 44394116 , 44394169 , 44394117 , 44361291 , 44394220 , 44394149 , 44394161 , 44344193 , 2727678 , 44394248]

1061|P33527(140) [148201 , 2396 , 444455 , 2333 , 11967809 , 1715 , 5342 , 13806 , 3037617 , 8820 , 11790 , 31200]

1062|Q9Y5Y4(23) [11508736 , 448457 , 45270144 , 5280363 , 42641863 , 11292191 , 3715 , 49843471 , 11462174 , 123879 , 5280360 , 44158492]

1063|Q9HBY8(1) [6419766 , 447966 , 2396 , 11608401 , 9549303 , 11712649 , 16122633 , 448008 , 76098 , 5005498 , 3973 , 2856]

1064|P13866(18) [44560296 , 25195624 , 44560297 , 24812758 , 44814423 , 9887712 , 11949646 , 44560294 , 9824918 , 11988953 , 44560302 , 44560286]

1065|P30536(74) [33746 , 6433109 , 31640 , 3033621 , 3261 , 11967800 , 204105 , 2717 , 2811 , 37632 , 3380 , 3117]

1066|P09874(197) [63306 , 657038 , 1511 , 23725625 , 25070031 , 936 , 9796068 , 148140 , 44819241 , 9925908 , 44403396 , 9931954]

1067|P04626(334) [208908 , 10437018 , 11511120 , 9915743 , 10184653 , 9874913 , 6444692 , 11349170 , 10458325 , 11488320 , 11620908 , 6918508 , 153241]

1068|P04054(103) [5311052 , 3599 , 71415 , 6714002 , 131505 , 2090 , 5311067 , 237 , 10133 , 84088 , 155815 , 129148 , 7329]

1069|P00915(18) [5284627 , 216468 , 5355 , 2732 , 5734 , 5284549 , 1986 , 3295 , 4107 , 68844 , 4100 , 370 , 3038]

1070|Q92831(41) [25066642 , 53258426 , 56663055 , 56683417 , 53258425 , 53258302 , 3581192 , 56666522 , 44448303 , 65064 , 56669931 , 56676753 , 56663053]

1071|P03372(392) [3606 , 154257 , 448537 , 5991 , 104741 , 5035 , 2733526 , 5756 , 5870 , 5757 , 216416 , 2800 , 5284643]

1072|Q13224(33) [3821 , 71077 , 52919 , 180081 , 219101 , 3559 , 68736 , 888 , 6468 , 156328 , 22880 , 750 , 2130]

1073|O95977(4) [44394169 , 44394247 , 44344210 , 107970 , 44394149 , 44394248 , 44394117 , 44394161 , 327045 , 44394220 , 44344193 , 44344194 , 44344404]

1074|O43570(23) [5734 , 216468 , 68844 , 5355 , 2732 , 1986 , 5284549 , 3295 , 5284627 , 4100 , 119607 , 3038 , 2662]

1075|P08185(29) [3033993 , 5755 , 5875 , 443980 , 4506 , 6918155 , 31307 , 6215 , 5311412 , 9642 , 15209 , 5311000 , 5311167]

1076|P47901(7) [60943 , 644077 , 5311010 , 9895468 , 10298385 , 14257660 , 119369 , 644076 , 53330936 , 10034060 , 11340891 , 439302 , 11634973]

1077|P10632(57) [11967800 , 17754438 , 148201 , 16362 , 2247 , 21109 , 3151 , 3698 , 59823 , 170364 , 16363 , 208908 , 4506]

1078|P22460(27) [216327 , 4932 , 1727 , 104826 , 3356 , 39186 , 2474 , 9571004 , 5413 , 4485 , 441074 , 2798 , 208898]

1079|Q08499(60) [154575 , 166553 , 3025803 , 11561674 , 9896267 , 5092 , 151170 , 5722 , 5723 , 17754438 , 449193 , 3758 , 3182 , 3671]

1080|P22894(90) [94413 , 128564 , 151506 , 69521 , 54671203 , 12473 , 1066 , 42601552 , 53317936 , 9933197 , 24800541 , 73761 , 119031 , 1269845]

1081|P43115(43) [11508736 , 5312153 , 49843471 , 5282381 , 5280363 , 5280360 , 5311100 , 3559 , 1615 , 5311044 , 6918140 , 115237 , 2159 , 3389]

1082|P18507(27) [37632 , 4506 , 4999 , 3033621 , 2441 , 2811 , 4890 , 3261 , 31640 , 31304 , 2170 , 2789 , 104781 , 3369]

1083|P20248(71) [4564 , 6852201 , 4565 , 160355 , 4566 , 447656 , 447655 , 1540 , 10224714 , 447961 , 9601217 , 449087 , 5289411 , 447649]

1084|Q02083(13) [74288 , 575358 , 1355 , 44398718 , 46229793 , 46229839 , 46229841 , 46230476 , 20514378 , 46229840 , 46229792 , 46229791 , 46229794 , 46229746]

1085|P30556(235) [60919 , 172198 , 5281037 , 130881 , 132706 , 3749 , 2541 , 3082475 , 60846 , 3961 , 65999 , 9825285 , 158781 , 60921]

1086|A8MPY1(1) [4890 , 4506 , 104781 , 37632 , 31304 , 4999 , 2811 , 4266 , 31640 , 3369 , 2441 , 3261 , 3448 , 3033621]

1087|O95749(7) [4395717 , 53319062 , 44452371 , 447277 , 5276507 , 25023860 , 53321690 , 25023863 , 5276503 , 447278 , 16122553 , 24832031 , 5276520 , 130956]

1088|P47870(7) [2811 , 5281077 , 2441 , 31640 , 3369 , 4506 , 2170 , 31304 , 4890 , 2789 , 37632 , 3261 , 3033621 , 104781]

1089|Q02127(24) [15433 , 4064 , 54684141 , 3899 , 15942657 , 1543 , 159866 , 54723327 , 10239 , 54727975 , 54727972 , 4112111 , 51347395 , 54727985]

1090|O00748(29) [79690 , 138508 , 13005 , 62539 , 10902085 , 3321360 , 176445 , 10879668 , 146089 , 26257 , 237515 , 44455840 , 11747 , 76915]

1091|Q9UKV0(98) [419176 , 6445533 , 6918638 , 9804992 , 4261 , 11538455 , 5352062 , 6918837 , 24756910 , 10313 , 5311 , 10309899 , 88129 , 53340666]

1092|P11940(18) [659036 , 2866904 , 327044 , 327045 , 3082 , 3132640 , 288875 , 752652 , 5392 , 11061 , 24892221 , 547914 , 1595804 , 2327]

1093|O00591(7) [3369 , 3033621 , 2811 , 37632 , 2789 , 104781 , 31304 , 4999 , 4890 , 4506 , 3261 , 2441 , 31640 , 2170]

1094|Q99928(4) [3033621 , 4506 , 104781 , 31640 , 4999 , 2441 , 2811 , 2170 , 31304 , 2789 , 4890 , 37632 , 3369 , 3261]

1095|Q8WUI4(14) [10313 , 88129 , 6918837 , 53340666 , 11538455 , 5352062 , 9804992 , 6918638 , 4261 , 10309899 , 419176 , 2746 , 6445533 , 9865515 , 24756910]

1096|P78334(6) [37632 , 2811 , 104781 , 3369 , 4890 , 2441 , 3033621 , 3261 , 2170 , 2789 , 4999 , 4506 , 33746 , 31304 , 31640]

1097|Q8N1C3(1) [2789 , 3261 , 4506 , 2170 , 104781 , 3033621 , 4999 , 2811 , 4890 , 31304 , 37632 , 33746 , 3369 , 31640 , 2441]

1098|O95136(25) [44394117 , 44394247 , 44344194 , 44394220 , 1720828 , 44344404 , 44394169 , 44344210 , 44394161 , 44394248 , 44344193 , 44394149 , 107970 , 44394116 , 1568843]

1099|P28476(5) [3033621 , 4890 , 31640 , 3261 , 4999 , 4266 , 3369 , 130021 , 2441 , 104781 , 4506 , 2811 , 37632 , 31304 , 3448]

1100|P24046(1) [3448 , 37632 , 130021 , 31304 , 31640 , 104781 , 2811 , 4506 , 4999 , 3033621 , 3369 , 4266 , 4890 , 3261 , 2441]

1101|P08754(7) [11532642 , 11696933 , 11553599 , 11595656 , 11561507 , 11675664 , 11661302 , 11641511 , 11660784 , 11690308 , 11625733 , 11641883 , 11674801 , 11689506 , 11532035]

1102|Q9UQL6(19) [88129 , 2746 , 4996 , 24756910 , 9865515 , 419176 , 11538455 , 9804992 , 5311 , 6918638 , 10313 , 53340666 , 6445533 , 5352062 , 10309899]

1103|O00418(12) [3078519 , 3542 , 3973 , 5005498 , 6918454 , 10172943 , 11712649 , 447966 , 9549303 , 160355 , 2856 , 3540 , 6419766 , 2396 , 153999]

1104|P34972(66) [115237 , 10624 , 568235 , 16078 , 11695365 , 119828 , 11515193 , 44274794 , 10253143 , 20514378 , 44406743 , 42617987 , 4218308 , 104850 , 1548942]

1105|P05771(50) [16122633 , 76098 , 11608401 , 153999 , 176167 , 448008 , 6918670 , 2856 , 6419766 , 2396 , 447966 , 3973 , 11712649 , 9549303 , 5005498 , 11442891]

1106|P15121(87) [16058629 , 16058630 , 53359 , 1613 , 153948 , 5724 , 10114 , 25210476 , 11292191 , 4369325 , 337359 , 1944 , 157839 , 160024 , 1549120 , 448658]

1107|P37231(357) [170364 , 644213 , 400010 , 5591 , 5280961 , 400769 , 3033 , 447458 , 154000 , 2750 , 4829 , 5329099 , 4075 , 77999 , 204109 , 3715]

1108|Q13315(279) [806859 , 13791 , 76915 , 3435 , 660051 , 2392140 , 1580955 , 2122 , 3973 , 53708 , 141870 , 307963 , 1599306 , 4342 , 20544 , 3108]

1109|O14764(21) [31304 , 3369 , 3261 , 4890 , 3033621 , 2441 , 33746 , 4999 , 37632 , 3448 , 2170 , 104781 , 2789 , 2811 , 31640 , 4506]

1110|Q14432(14) [19987169 , 2754 , 3033825 , 3973 , 53708 , 3671 , 24316 , 16960 , 2182 , 5663 , 204105 , 4197 , 449193 , 17754438 , 2753 , 3698]

1111|P08235(89) [31378 , 5839 , 5311505 , 11876263 , 5754 , 3034004 , 5994 , 5833 , 3333 , 68873 , 5743 , 443872 , 4497 , 16734800 , 5755 , 10133]

1112|P30968(45) [16130938 , 16137348 , 49800011 , 36523 , 3038517 , 11250647 , 25077993 , 10348973 , 657181 , 50225 , 25077405 , 16136245 , 16131215 , 25074887 , 25074470 , 5311128]

1113|P25774(35) [11695261 , 46228924 , 10152654 , 9547943 , 13186895 , 5288616 , 10201696 , 24901101 , 10324367 , 5288259 , 11514361 , 14060550 , 46937176 , 11651881 , 24800541 , 11464526]

1114|Q13547(78) [9804992 , 6918837 , 5222465 , 9865515 , 11538455 , 5311 , 53340666 , 5352062 , 4775 , 3121 , 2746 , 6445533 , 6918638 , 4261 , 264 , 4996 , 11609955]

1115|P05129(28) [5005498 , 176167 , 2856 , 6419766 , 11712649 , 16122633 , 2396 , 6918670 , 448008 , 76098 , 9549303 , 447966 , 11314340 , 25227436 , 11608401 , 153999 , 3973]

1116|P14324(13) [4674 , 23730246 , 57637 , 23730244 , 5245 , 1195 , 23730364 , 23730248 , 68740 , 60852 , 448400 , 1023 , 130956 , 2088 , 44452371 , 23730136 , 15230201]

1117|Q14191(83) [612424 , 24816706 , 7475369 , 10168 , 2016 , 13752 , 16467159 , 1973720 , 22430877 , 4680274 , 1568843 , 7329 , 2090 , 2743305 , 16347 , 12449 , 3746037]

1118|Q15822(3) [10235 , 3604 , 115237 , 1615 , 1057 , 441290 , 2968 , 3389 , 24244 , 39765 , 2366 , 4032 , 21233 , 50192 , 3559 , 5831 , 5281042]

1119|P27815(33) [154575 , 204105 , 1676 , 3108 , 19987169 , 9827968 , 110635 , 5722 , 1712095 , 4197 , 5092 , 3671 , 151170 , 3182 , 16960 , 9896267 , 449193 , 2754]

1120|P20813(194) [16362 , 148201 , 11332763 , 2361 , 11229234 , 12229396 , 59823 , 2355 , 448632 , 154104 , 182137 , 51049968 , 16363 , 2247 , 11790 , 4506 , 3151 , 1349907]

1121|O75469(183) [1684 , 176870 , 2812 , 104741 , 4763 , 3401 , 16734800 , 5757 , 3333 , 55245 , 4485 , 10133 , 441298 , 446313 , 36314 , 56950369 , 10229498 , 53232]

1122|P35408(65) [3389 , 9890801 , 5311044 , 3559 , 5280360 , 2159 , 1615 , 11677589 , 49843471 , 5280723 , 9803828 , 6918140 , 115237 , 5282381 , 5282411 , 5280363 , 448457 , 11508736]

1123|Q9Y5N1(13) [44418490 , 3077 , 16119086 , 2366 , 2913 , 24771368 , 11697697 , 9948102 , 44418482 , 6450823 , 25070031 , 16049886 , 9976892 , 24745335 , 774 , 16119082 , 3389 , 41376 , 17747460]

1124|Q16644(3) [3542 , 6419766 , 3078519 , 5005498 , 2856 , 448008 , 76098 , 11608401 , 11712649 , 9549303 , 10172943 , 153999 , 6918454 , 3540 , 160355 , 447966 , 2396 , 16122633 , 3973]

1125|Q9Y2D0(0) [72139 , 36811 , 462919 , 6307 , 216468 , 5287541 , 76509 , 11117301 , 2732 , 12066940 , 3295 , 14611919 , 19772348 , 389641 , 5356 , 169682 , 11967800 , 16122590 , 12066941]

1126|P30542(32) [11270783 , 21874557 , 5311037 , 158795 , 10117987 , 11561692 , 9860294 , 3758 , 3035850 , 9576912 , 2519 , 216466 , 219024 , 6439091 , 64627 , 9953065 , 123683 , 60961 , 2153]

1127|Q14524(140) [3180 , 4935 , 3025 , 3356 , 3114 , 56339 , 4060 , 7699 , 3292 , 38945 , 441074 , 52195 , 4178 , 34633 , 4913 , 4906 , 48041 , 3676 , 10770]

1128|P10275(333) [6010 , 13765 , 3397 , 9880 , 9904 , 4493 , 224004 , 251636 , 2375 , 5995 , 261000 , 6013 , 10635 , 55245 , 15951529 , 6446 , 9824562 , 5878 , 6011]

1129|Q13639(24) [3052762 , 53354764 , 9805719 , 6918314 , 216236 , 71451950 , 3052778 , 2769 , 11430856 , 3388 , 11961293 , 9860294 , 5362436 , 177336 , 656665 , 154104 , 68867 , 108182 , 119584]

1130|P06401(132) [104741 , 6279 , 16734800 , 130904 , 9270 , 6917715 , 55245 , 5994 , 5311505 , 9577221 , 13109 , 36709 , 4369524 , 6230 , 11683 , 40973 , 6540478 , 9051 , 13559281]

1131|P38398(209) [4404908 , 1432578 , 1720828 , 2090 , 17113 , 24817194 , 3108 , 3240818 , 3746037 , 19646 , 1878823 , 680935 , 265580 , 1568843 , 2291046 , 2914644 , 5005498 , 5198 , 65758 , 24792593]

1132|O75762(42) [16590 , 3026 , 6989 , 16315 , 49381 , 11715 , 12228 , 6005 , 24823 , 5469318 , 3117 , 65036 , 16666 , 21782 , 11617 , 177 , 7847 , 880 , 89594 , 16078]

1133|P08912(1) [174174 , 444031 , 6646 , 4848 , 2381 , 24199 , 9577995 , 9571002 , 3494 , 71183 , 5910 , 441071 , 4926 , 2160 , 4167 , 60809 , 187 , 107867 , 2551 , 442021]

1134|Q05513(35) [3540 , 2856 , 2396 , 9549303 , 176167 , 11712649 , 10172943 , 11608401 , 16122633 , 6918454 , 76098 , 6419766 , 447966 , 160355 , 3973 , 153999 , 3542 , 5005498 , 444899 , 3078519]

1135|P35218(14) [36811 , 216468 , 19772348 , 5287541 , 11117301 , 72139 , 6852128 , 389641 , 12066941 , 3295 , 12066940 , 16122590 , 76509 , 462919 , 169682 , 2732 , 6307 , 5356 , 10112 , 11967800 , 14611919]

1136|Q92887(109) [47318 , 5074 , 3478 , 158781 , 4122 , 176870 , 21138 , 16231 , 11286230 , 2247 , 2333 , 2812 , 119373 , 3108 , 3639 , 5405 , 3559 , 119259 , 3333 , 31729 , 10133]

1137|P29274(49) [11270783 , 123683 , 60961 , 11561692 , 65710 , 9576912 , 64627 , 3035850 , 10680 , 158795 , 5311037 , 6439091 , 208820 , 2153 , 219024 , 10117987 , 216466 , 2519 , 855908 , 9860294 , 21874557]

1138|O15245(59) [2179 , 155774 , 175540 , 1349907 , 3478 , 3559 , 2265 , 3108 , 3639 , 5405 , 4342 , 3333 , 16231 , 119259 , 183797 , 199 , 1549093 , 21138 , 2812 , 2576 , 123600]

1139|Q92769(69) [3994 , 2466 , 49855250 , 88129 , 53340666 , 24756910 , 10313 , 9804992 , 6918837 , 4261 , 5352062 , 11538455 , 264 , 6445533 , 9865515 , 6918638 , 10309899 , 419176 , 2746 , 5311 , 4996]

1140|P23141(32) [185195 , 2893 , 79690 , 76915 , 62539 , 176445 , 10013998 , 26257 , 237515 , 11493344 , 138508 , 10902085 , 4912 , 13005 , 39042 , 3321360 , 146089 , 11747 , 182197 , 44455840 , 10879668]

1141|P00533(966) [10184653 , 6445562 , 176870 , 22024915 , 124437 , 6918508 , 71496458 , 6918403 , 5329099 , 153241 , 11511120 , 10437018 , 4510 , 3081361 , 123631 , 11349170 , 6444692 , 11488320 , 9915743 , 208908 , 10458325]

1142|P34913(50) [47379736 , 11053022 , 44235174 , 3001386 , 49782570 , 6420120 , 4359 , 104741 , 53323158 , 25073288 , 49782569 , 44234911 , 6420121 , 25070135 , 53320344 , 11167602 , 44235634 , 4357 , 44234913 , 43608139 , 4358 , 11160856]

1143|O00141(74) [3540 , 5326739 , 448008 , 3078519 , 5005498 , 447966 , 3542 , 10172943 , 1694 , 2856 , 10267580 , 3973 , 9549303 , 2396 , 6419766 , 160355 , 16122633 , 11608401 , 6918454 , 153999 , 76098 , 11712649]

1144|Q8IUX4(6) [3108 , 327045 , 2832737 , 3003803 , 3377088 , 3244341 , 42725 , 6472026 , 72139 , 67686 , 3442589 , 4343310 , 16269005 , 32681 , 100472 , 6301 , 166553 , 16347 , 1937568 , 18573524 , 19910 , 2220273]

1145|Q8NER1(103) [104826 , 2998 , 1548943 , 1548942 , 65036 , 2913 , 42617987 , 16007367 , 11256560 , 24752296 , 3245451 , 3559 , 20514378 , 21138 , 588415 , 4628 , 638024 , 5311093 , 9910486 , 2170 , 2435 , 1719873]

1146|P18031(66) [449162 , 17759780 , 1757 , 445784 , 114829 , 47318 , 17758920 , 444764 , 9547959 , 10305301 , 447994 , 1854 , 447695 , 2194 , 1628 , 447450 , 25111933 , 9547919 , 6914659 , 10114 , 1829 , 9547958 , 5327154]

1147|P23280(6) [1057 , 6852128 , 12066941 , 216468 , 14611919 , 19772348 , 5356 , 3295 , 16129778 , 3161908 , 2732 , 169682 , 72139 , 16122590 , 11967800 , 389641 , 36811 , 10112 , 5287541 , 11117301 , 76509 , 6307 , 12066940]

1148|O00167(11) [3156995 , 44602029 , 3238160 , 2775706 , 307963 , 2090 , 1985 , 1745499 , 701332 , 6469502 , 4031 , 263177 , 1392 , 13791 , 2490338 , 3236502 , 1614257 , 3108 , 10212 , 1552036 , 3237439 , 327045 , 824727]

1149|P56524(49) [11844893 , 9865515 , 23634895 , 11538455 , 6445533 , 11844892 , 10309899 , 10313 , 419176 , 4261 , 24756910 , 2746 , 11844891 , 6918638 , 23634892 , 23634893 , 9804992 , 23634894 , 53340666 , 5352062 , 88129 , 6918837 , 23634770]

1150|P23219(140) [3825 , 54677470 , 5280581 , 4781 , 4614 , 1983 , 5161 , 5359 , 4075 , 6335412 , 156391 , 60726 , 3033 , 3342 , 4495 , 54676228 , 4044 , 3394 , 338 , 2244 , 3672 , 3826 , 4037]

1151|O15379(56) [2466 , 10313 , 49855250 , 264 , 3994 , 5352062 , 6918638 , 5311 , 2746 , 24756910 , 6445533 , 9804992 , 208908 , 4261 , 419176 , 11609955 , 53340666 , 4996 , 10309899 , 11538455 , 88129 , 9865515 , 6918837]

1152|Q99685(15) [20449950 , 2359 , 5043631 , 70261 , 45484105 , 139950 , 13072144 , 12704 , 4362 , 7347 , 65389 , 44550866 , 4526314 , 12351263 , 44274794 , 13258570 , 25057 , 3117 , 44560662 , 3572 , 512831 , 606562 , 44560663]

1153|Q9H3N8(20) [3559 , 115237 , 25070031 , 2159 , 11697697 , 3389 , 119828 , 1547484 , 10624 , 17747460 , 2170 , 41376 , 24745335 , 3077 , 16106 , 2725 , 2247 , 2818 , 2913 , 774 , 4761 , 119570 , 9976892]

1154|Q5JVE8(0) [10182969 , 216210 , 183797 , 10250490 , 9549258 , 446345 , 204102 , 11634458 , 11641515 , 447362 , 6540267 , 447359 , 10343728 , 6540268 , 42628060 , 104625 , 446346 , 24800541 , 152951 , 10095865 , 42601552 , 9549257 , 24794406]

1155|Q16445(9) [10518 , 2170 , 2441 , 31304 , 104781 , 3261 , 10133 , 10531 , 3448 , 4064 , 107926 , 31640 , 3003157 , 10237 , 2789 , 65914 , 4506 , 4266 , 3380 , 3373 , 2893 , 2576 , 32051]

1156|P12821(491) [91270 , 107807 , 5464343 , 5484727 , 5463984 , 5362124 , 5462501 , 5311447 , 42601552 , 5388962 , 56330 , 107994 , 44093 , 5464344 , 5362119 , 92400 , 72022 , 5464097 , 3033702 , 5464096 , 32681 , 5362129 , 55891 , 6604423]

1157|P09917(156) [3052 , 3600 , 4992 , 71398 , 60490 , 124087 , 54675783 , 1066 , 60923 , 71751 , 12473 , 6439232 , 69521 , 10368812 , 133021 , 3610 , 94413 , 151506 , 3086671 , 73761 , 1269845 , 53317936 , 56237 , 11508736]

1158|P10145(524) [81530 , 2265 , 15250 , 3118 , 11604 , 7798 , 11167 , 25644 , 16231 , 2950 , 28803 , 8196 , 1057 , 15286 , 123600 , 11293 , 61247 , 10168 , 19996 , 14242 , 11852 , 3672772 , 6540 , 10868]

1159|Q02880(22) [2179 , 5379 , 42890 , 124093 , 4421 , 9952884 , 4212 , 3948 , 62959 , 152946 , 41867 , 10180 , 149096 , 2764 , 60464 , 3229 , 4583 , 4539 , 9571107 , 31703 , 287180 , 2762 , 3357 , 51081]

1160|P08253(384) [5362422 , 94413 , 1269845 , 119031 , 53317936 , 69521 , 151506 , 466151 , 16108938 , 128564 , 6918336 , 24768528 , 24800541 , 10039403 , 60937 , 73761 , 42601552 , 3342298 , 10610500 , 448002 , 10492779 , 12473 , 1066 , 9933197]

1161|O94956(9) [6324616 , 119373 , 3404 , 130881 , 3333 , 5311236 , 2333 , 176870 , 46181428 , 5405 , 2812 , 3433 , 3463 , 1017 , 3117 , 2247 , 3478 , 21138 , 3488 , 5335 , 3108 , 175540 , 11967809 , 11286230 , 119259]

1162|P08913(32) [5702063 , 4893 , 5707 , 5268 , 439260 , 2726 , 443951 , 3519 , 5504 , 5816 , 2803 , 2216 , 8969 , 47811 , 213046 , 2435 , 28864 , 5775 , 4636 , 5311068 , 4850 , 54746 , 31101 , 4205 , 6005]

1163|P30939(2) [21138 , 4440 , 5078 , 8969 , 1150 , 5002 , 197706 , 5073 , 8226 , 10531 , 3389 , 4106 , 77993 , 8223 , 28693 , 182137 , 2818 , 60149 , 23897 , 11610526 , 60857 , 4585 , 60809 , 5358 , 3559]

1164|P47898(4) [1615 , 8223 , 44623946 , 8969 , 3389 , 9966051 , 115237 , 119570 , 2818 , 11954293 , 9860294 , 10624 , 3822 , 11430856 , 5074 , 4106 , 28693 , 11961293 , 5358 , 197706 , 10100 , 2159 , 3559 , 71768094 , 1355]

1165|P18825(19) [838 , 213046 , 219050 , 31101 , 4636 , 5707 , 443951 , 4205 , 6005 , 439260 , 2216 , 5268 , 2435 , 28864 , 54746 , 5775 , 3519 , 2803 , 4850 , 5702063 , 5504 , 4893 , 47811 , 8969 , 2726 , 5311068]

1166|P18089(68) [4636 , 5311068 , 4893 , 28864 , 2726 , 4205 , 3519 , 8969 , 4740 , 31101 , 838 , 5702063 , 5775 , 4768 , 5504 , 219050 , 439260 , 54746 , 2803 , 2435 , 5707 , 5736 , 47811 , 5268 , 443951 , 6005]

1167|P06276(73) [10879668 , 1933 , 15083623 , 1547484 , 77991 , 10902085 , 54766 , 10198924 , 26257 , 2131 , 62539 , 5983 , 1935 , 5936 , 5420 , 11747 , 79690 , 138508 , 13005 , 3108 , 446980 , 237515 , 3321360 , 20689 , 15192 , 76915]

1168|P29275(30) [6439091 , 9953065 , 219024 , 2893 , 10117987 , 11561692 , 21874557 , 3035850 , 158795 , 5311037 , 16071896 , 10531 , 123683 , 1676 , 216466 , 2519 , 9860294 , 16960 , 19987169 , 9576912 , 11270783 , 1188 , 7 , 1892 , 2153 , 60961]

1169|P27487(117) [5494422 , 6918537 , 24800541 , 23646100 , 4369359 , 11426348 , 42608447 , 10932707 , 205131 , 10198228 , 11243969 , 2734054 , 10376704 , 23646087 , 9887755 , 46209133 , 11516136 , 11450633 , 11659120 , 11949652 , 25022354 , 44513473 , 5459374 , 11572962 , 44387758 , 15991577 , 10096344]

1170|Q9NPD5(42) [3117 , 3404 , 158781 , 176870 , 5212 , 3463 , 3478 , 175540 , 2333 , 2812 , 119259 , 5284373 , 24988881 , 5335 , 3108 , 46181428 , 14982 , 2247 , 5405 , 11967809 , 3333 , 130881 , 3433 , 119373 , 21138 , 11286230 , 6324616]

1171|P22748(20) [2343 , 3295 , 6852128 , 5356 , 36811 , 72139 , 389641 , 5284627 , 169682 , 12066941 , 11117301 , 12066940 , 5287541 , 2315 , 4100 , 6307 , 10112 , 1003 , 11967800 , 3038 , 16122590 , 19772348 , 76509 , 2732 , 3647 , 1986 , 14611919 , 3639]

1172|Q8TDS4(23) [53320057 , 24954204 , 53325309 , 53325627 , 16098 , 24955289 , 938 , 53324022 , 51576 , 53326893 , 15605500 , 53321367 , 637542 , 53326575 , 56950369 , 24955288 , 53317449 , 10931 , 53318740 , 53326616 , 53324024 , 53325308 , 5310993 , 53318768 , 53321351 , 24955642 , 53318741 , 25025505]

1173|Q9Y6L6(62) [3333 , 3433 , 11286230 , 24988881 , 2812 , 3463 , 54687 , 46181428 , 11967809 , 3478 , 5212 , 5405 , 158781 , 3117 , 175540 , 119259 , 2333 , 6324616 , 21138 , 130881 , 5335 , 3108 , 5284373 , 14982 , 2247 , 176870 , 3404 , 119373]

1174|P29475(164) [10198539 , 347590 , 11149707 , 10261068 , 123895 , 15689 , 24894151 , 19049152 , 9797857 , 1649 , 447029 , 19049087 , 15552206 , 22617750 , 19049121 , 3311 , 12778906 , 107984 , 447030 , 1893 , 18721726 , 132862 , 10221335 , 44396192 , 1433 , 18721725 , 18721724 , 18721729]

1175|P43235(77) [5288867 , 6918602 , 44391415 , 24901101 , 44398651 , 5289428 , 44398660 , 46228924 , 10062713 , 10152654 , 44398689 , 44398780 , 5288988 , 10062714 , 44398665 , 11514361 , 5288259 , 11464526 , 44398659 , 14060550 , 13186895 , 44391459 , 5288616 , 11651881 , 44398650 , 10201696 , 10324367 , 11695261 , 44398688]

1176|P35228(332) [18721729 , 11149707 , 15552206 , 24894151 , 2146 , 123895 , 18721726 , 10104379 , 107984 , 19049121 , 1649 , 22617750 , 1433 , 44322997 , 19049152 , 15689 , 107782 , 44396192 , 10198539 , 18721724 , 5754 , 12778906 , 18721725 , 9797857 , 657073 , 132862 , 10261068 , 19049087 , 10221335]

1177|P02768(251) [1057 , 8820 , 151170 , 3559 , 3503 , 3108 , 3739 , 11634973 , 54677971 , 123600 , 33746 , 54676038 , 9976892 , 16130199 , 3194 , 24776445 , 11552706 , 54677972 , 54676537 , 119259 , 2997 , 46937068 , 3639 , 11372270 , 21138 , 2725 , 449193 , 54680692 , 6604423 , 115358]

1178|O95747(1) [10427712 , 176870 , 10127622 , 11427553 , 10113978 , 11314340 , 11712649 , 3025986 , 208908 , 16722836 , 176167 , 153999 , 11667893 , 11656518 , 6918454 , 11485656 , 17755052 , 11213558 , 24889392 , 151194 , 11338033 , 156414 , 10074640 , 11234052 , 11409972 , 3038522 , 11364421 , 24779724 , 447077 , 15983966]

1179|P46063(19) [327045 , 2750 , 5282060 , 15945601 , 3746037 , 31236 , 3243567 , 2540 , 1781 , 669633 , 2768975 , 5289501 , 107738 , 3238778 , 1973720 , 948382 , 11852 , 1057 , 2396 , 1780 , 2179 , 51040 , 2333 , 2758 , 10168 , 3698 , 54675783 , 3503 , 3885 , 1694]

1180|Q92772(5) [11656518 , 11213558 , 11667893 , 208908 , 11712649 , 3038522 , 176167 , 447077 , 11338033 , 3025986 , 6918454 , 176870 , 11409972 , 17755052 , 11314340 , 10127622 , 11234052 , 11427553 , 10113978 , 156414 , 11485656 , 151194 , 24779724 , 10427712 , 16722836 , 153999 , 11364421 , 10074640 , 24889392 , 15983966]

1181|Q13523(9) [15983966 , 176167 , 11364421 , 11213558 , 11338033 , 156414 , 176870 , 16722836 , 151194 , 11234052 , 3038522 , 153999 , 11656518 , 447077 , 208908 , 10427712 , 6918454 , 11667893 , 11409972 , 17755052 , 11314340 , 11712649 , 24889392 , 11427553 , 11485656 , 10074640 , 10127622 , 10113978 , 3025986 , 24779724]

1182|Q9BQI3(15) [11234052 , 24779724 , 10113978 , 3025986 , 176870 , 15983966 , 156414 , 447077 , 10127622 , 11314340 , 10427712 , 11485656 , 6918454 , 11656518 , 11213558 , 11409972 , 17755052 , 151194 , 11667893 , 11338033 , 208908 , 16722836 , 11364421 , 24889392 , 11427553 , 3038522 , 153999 , 10074640 , 176167 , 11712649]

1183|Q52WX2(0) [24779724 , 176870 , 11667893 , 176167 , 11338033 , 447077 , 3025986 , 24889392 , 15983966 , 153999 , 11364421 , 6918454 , 10074640 , 10127622 , 10113978 , 156414 , 11213558 , 17755052 , 151194 , 11712649 , 11314340 , 3038522 , 11234052 , 11427553 , 10427712 , 16722836 , 208908 , 11485656 , 11656518 , 11409972]

1184|Q8TDR2(0) [11213558 , 447077 , 11656518 , 10113978 , 11427553 , 11409972 , 153999 , 11364421 , 17755052 , 11314340 , 11712649 , 16722836 , 3025986 , 151194 , 24889392 , 156414 , 10127622 , 11338033 , 176167 , 24779724 , 11667893 , 176870 , 10074640 , 11485656 , 10427712 , 15983966 , 208908 , 6918454 , 11234052 , 3038522]

1185|Q15208(10) [3025986 , 11409972 , 447077 , 11485656 , 10074640 , 11338033 , 15983966 , 153999 , 11364421 , 151194 , 208908 , 11427553 , 11656518 , 11667893 , 10427712 , 10127622 , 17755052 , 11712649 , 176870 , 3038522 , 11314340 , 16722836 , 10113978 , 24779724 , 11213558 , 6918454 , 156414 , 176167 , 24889392 , 11234052]

1186|O75385(15) [3038522 , 11485656 , 15983966 , 6918454 , 10074640 , 16722836 , 3025986 , 11234052 , 153999 , 151194 , 10127622 , 24779724 , 447077 , 17755052 , 11667893 , 11656518 , 176167 , 11338033 , 11427553 , 208908 , 11364421 , 10113978 , 11314340 , 11409972 , 11712649 , 176870 , 11213558 , 24889392 , 10427712 , 156414]

1187|Q9UPZ9(39) [11364421 , 24889392 , 16722836 , 11213558 , 10427712 , 3038522 , 11667893 , 11314340 , 208908 , 447077 , 24779724 , 156414 , 176167 , 10127622 , 11656518 , 6918454 , 3025986 , 15983966 , 151194 , 11338033 , 11427553 , 153999 , 10074640 , 176870 , 11409972 , 11485656 , 11712649 , 10113978 , 11234052 , 17755052]

1188|Q6XUX3(4) [10127622 , 11213558 , 16722836 , 11364421 , 17755052 , 11667893 , 176167 , 11485656 , 11314340 , 11712649 , 153999 , 151194 , 11234052 , 156414 , 447077 , 208908 , 24889392 , 3038522 , 176870 , 10427712 , 11409972 , 11656518 , 11338033 , 6918454 , 3025986 , 10074640 , 10113978 , 24779724 , 11427553 , 15983966]

1189|P32298(13) [208908 , 10127622 , 3025986 , 11314340 , 11485656 , 11213558 , 11409972 , 15983966 , 176870 , 10113978 , 447077 , 11427553 , 151194 , 11364421 , 24889392 , 3038522 , 11338033 , 153999 , 11667893 , 10074640 , 11234052 , 176167 , 11712649 , 16722836 , 24779724 , 10427712 , 6918454 , 17755052 , 11656518 , 156414]

1190|Q9UEW8(9) [208908 , 11656518 , 11364421 , 24779724 , 11712649 , 11314340 , 15983966 , 10113978 , 176870 , 151194 , 3038522 , 153999 , 17755052 , 11427553 , 16722836 , 6918454 , 24889392 , 11667893 , 11485656 , 11409972 , 11234052 , 447077 , 10074640 , 10127622 , 176167 , 11213558 , 10427712 , 3025986 , 156414 , 11338033]

1191|O43283(5) [11485656 , 11234052 , 153999 , 10074640 , 156414 , 176870 , 10427712 , 208908 , 11314340 , 24779724 , 176167 , 11338033 , 11409972 , 6918454 , 17755052 , 16722836 , 15983966 , 11427553 , 11712649 , 24889392 , 447077 , 3038522 , 151194 , 10113978 , 11213558 , 11364421 , 10127622 , 3025986 , 11667893 , 11656518]

1192|O00238(24) [11656518 , 11364421 , 11712649 , 3038522 , 11409972 , 153999 , 10427712 , 11234052 , 11485656 , 15983966 , 11427553 , 10074640 , 11213558 , 447077 , 6918454 , 11338033 , 24779724 , 16722836 , 3025986 , 10127622 , 11314340 , 176167 , 176870 , 151194 , 156414 , 24889392 , 11667893 , 17755052 , 208908 , 10113978]

1193|P0C264(0) [11427553 , 11338033 , 153999 , 15983966 , 10427712 , 156414 , 11667893 , 208908 , 11364421 , 151194 , 16722836 , 17755052 , 11213558 , 10127622 , 447077 , 11656518 , 11712649 , 24779724 , 11409972 , 3038522 , 176870 , 176167 , 11314340 , 10074640 , 10113978 , 24889392 , 6918454 , 3025986 , 11234052 , 11485656]

1194|Q9Y2K2(8) [11712649 , 24889392 , 11667893 , 176167 , 11656518 , 3038522 , 10427712 , 153999 , 3025986 , 6918454 , 16722836 , 151194 , 10127622 , 11314340 , 11364421 , 11234052 , 11338033 , 156414 , 10113978 , 11485656 , 15983966 , 10074640 , 447077 , 24779724 , 208908 , 11213558 , 11427553 , 176870 , 11409972 , 17755052]

1195|P0C1S8(0) [208908 , 176167 , 176870 , 10427712 , 11427553 , 11409972 , 10127622 , 17755052 , 11667893 , 11213558 , 11234052 , 156414 , 6918454 , 11656518 , 3025986 , 151194 , 16722836 , 11364421 , 447077 , 3038522 , 24779724 , 10074640 , 10113978 , 11338033 , 11712649 , 24889392 , 11314340 , 15983966 , 11485656 , 153999]

1196|O60331(4) [24779724 , 10113978 , 10427712 , 11667893 , 3038522 , 11656518 , 11234052 , 11314340 , 153999 , 3025986 , 151194 , 10074640 , 11213558 , 17755052 , 11485656 , 15983966 , 16722836 , 24889392 , 6918454 , 208908 , 11409972 , 11712649 , 11427553 , 11338033 , 176167 , 156414 , 447077 , 11364421 , 176870 , 10127622]

1197|O75747(3) [11364421 , 208908 , 11213558 , 11234052 , 16722836 , 11314340 , 11427553 , 10074640 , 11656518 , 10127622 , 10427712 , 176870 , 156414 , 11409972 , 17755052 , 24779724 , 11338033 , 3038522 , 11485656 , 153999 , 15983966 , 447077 , 11667893 , 176167 , 6918454 , 24889392 , 151194 , 3025986 , 10113978 , 11712649]

1198|Q9Y2H9(0) [153999 , 11409972 , 10127622 , 11712649 , 156414 , 3038522 , 11234052 , 17755052 , 6918454 , 16722836 , 15983966 , 11213558 , 11485656 , 11427553 , 11667893 , 176870 , 10113978 , 151194 , 10427712 , 208908 , 24779724 , 3025986 , 11656518 , 24889392 , 11314340 , 10074640 , 447077 , 176167 , 11364421 , 11338033]

1199|Q8IVW4(1) [10127622 , 24889392 , 11712649 , 10427712 , 11213558 , 208908 , 6918454 , 15983966 , 10074640 , 3038522 , 176167 , 447077 , 11409972 , 24779724 , 3025986 , 17755052 , 11314340 , 11667893 , 11364421 , 16722836 , 11338033 , 11234052 , 11656518 , 156414 , 11485656 , 11427553 , 10113978 , 153999 , 176870 , 151194]

1200|Q6ZN16(0) [11485656 , 10113978 , 10427712 , 176870 , 3038522 , 15983966 , 11234052 , 11338033 , 10127622 , 11712649 , 11213558 , 11656518 , 11427553 , 11364421 , 11314340 , 11667893 , 16722836 , 11409972 , 153999 , 156414 , 447077 , 176167 , 10074640 , 24779724 , 24889392 , 3025986 , 6918454 , 151194 , 17755052 , 208908]

1201|O76039(37) [176870 , 10113978 , 151194 , 10427712 , 156414 , 153999 , 15983966 , 11364421 , 17755052 , 11213558 , 3025986 , 10074640 , 11427553 , 11338033 , 208908 , 3038522 , 447077 , 11314340 , 11667893 , 176167 , 16722836 , 11656518 , 11409972 , 24779724 , 6918454 , 24889392 , 11485656 , 10127622 , 11234052 , 11712649]

1202|Q99759(15) [11485656 , 10127622 , 24779724 , 208908 , 11712649 , 11213558 , 11234052 , 11338033 , 176167 , 10113978 , 11667893 , 6918454 , 3038522 , 24889392 , 11409972 , 151194 , 176870 , 153999 , 11427553 , 11364421 , 11314340 , 11656518 , 10074640 , 447077 , 15983966 , 10427712 , 16722836 , 156414 , 17755052 , 3025986]

1203|Q9BVS4(5) [11667893 , 153999 , 6918454 , 24779724 , 11234052 , 10074640 , 447077 , 10127622 , 15983966 , 3038522 , 16722836 , 17755052 , 3025986 , 11409972 , 11338033 , 10427712 , 11314340 , 156414 , 11364421 , 11712649 , 176870 , 151194 , 10113978 , 11427553 , 11656518 , 176167 , 11213558 , 11485656 , 24889392 , 208908]

1204|O14936(30) [6918454 , 447077 , 11409972 , 16722836 , 11338033 , 10427712 , 11213558 , 176870 , 11667893 , 11314340 , 24779724 , 11656518 , 3038522 , 11485656 , 24889392 , 176167 , 11364421 , 10074640 , 208908 , 10127622 , 15983966 , 11427553 , 156414 , 153999 , 3025986 , 11234052 , 17755052 , 10113978 , 151194 , 11712649]

1205|P42679(16) [176870 , 10127622 , 447077 , 11409972 , 17755052 , 3025986 , 11656518 , 11364421 , 153999 , 3038522 , 24889392 , 10427712 , 10074640 , 151194 , 208908 , 176167 , 156414 , 10113978 , 16722836 , 11314340 , 11667893 , 11427553 , 11234052 , 11213558 , 15983966 , 6918454 , 11338033 , 24779724 , 11485656 , 11712649]

1206|Q8WU08(1) [176870 , 10074640 , 208908 , 153999 , 156414 , 3038522 , 15983966 , 16722836 , 3025986 , 6918454 , 11234052 , 17755052 , 176167 , 151194 , 10113978 , 24779724 , 11338033 , 11364421 , 11656518 , 24889392 , 10427712 , 11667893 , 447077 , 11314340 , 10127622 , 11427553 , 11712649 , 11213558 , 11409972 , 11485656]

1207|Q15835(6) [24889392 , 151194 , 11427553 , 10127622 , 11712649 , 176167 , 156414 , 16722836 , 11314340 , 11364421 , 208908 , 11338033 , 10074640 , 15983966 , 3025986 , 11213558 , 24779724 , 11656518 , 10113978 , 11667893 , 11234052 , 17755052 , 153999 , 11485656 , 10427712 , 3038522 , 447077 , 6918454 , 11409972 , 176870]

1208|Q12852(13) [208908 , 11409972 , 24889392 , 10127622 , 156414 , 153999 , 11364421 , 10427712 , 11656518 , 11234052 , 11314340 , 11338033 , 447077 , 15983966 , 11712649 , 3025986 , 10113978 , 176870 , 10074640 , 11213558 , 11427553 , 16722836 , 6918454 , 176167 , 151194 , 17755052 , 11485656 , 3038522 , 24779724 , 11667893]

1209|Q9NRH2(3) [10074640 , 447077 , 11338033 , 11364421 , 24889392 , 16722836 , 156414 , 11234052 , 17755052 , 11667893 , 11314340 , 3038522 , 11712649 , 176870 , 10127622 , 11409972 , 10427712 , 11213558 , 3025986 , 151194 , 11427553 , 6918454 , 176167 , 10113978 , 15983966 , 208908 , 153999 , 24779724 , 11485656 , 11656518]

1210|O15197(17) [24889392 , 156414 , 11338033 , 11656518 , 11667893 , 3038522 , 11409972 , 17755052 , 11213558 , 151194 , 11314340 , 10427712 , 10074640 , 15983966 , 447077 , 10113978 , 24779724 , 11364421 , 176870 , 11485656 , 208908 , 11712649 , 176167 , 153999 , 11234052 , 11427553 , 10127622 , 3025986 , 16722836 , 6918454]

1211|Q00532(4) [176870 , 3038522 , 24889392 , 10113978 , 11656518 , 11338033 , 10427712 , 11409972 , 15983966 , 3025986 , 6918454 , 10074640 , 24779724 , 11213558 , 16722836 , 11427553 , 10127622 , 11314340 , 11712649 , 11364421 , 11234052 , 17755052 , 156414 , 11667893 , 176167 , 11485656 , 153999 , 151194 , 447077 , 208908]

1212|Q8TBX8(2) [10113978 , 11656518 , 156414 , 10427712 , 24889392 , 11712649 , 11364421 , 15983966 , 447077 , 176870 , 11427553 , 24779724 , 10074640 , 153999 , 11667893 , 11409972 , 11485656 , 151194 , 6918454 , 16722836 , 3025986 , 11338033 , 176167 , 3038522 , 11314340 , 17755052 , 11234052 , 10127622 , 11213558 , 208908]

1213|P57058(8) [153999 , 151194 , 11667893 , 11314340 , 17755052 , 11409972 , 11364421 , 11213558 , 10127622 , 176167 , 208908 , 15983966 , 11234052 , 176870 , 156414 , 6918454 , 447077 , 10427712 , 11485656 , 16722836 , 11656518 , 11338033 , 3025986 , 24779724 , 11427553 , 24889392 , 10113978 , 10074640 , 3038522 , 11712649]

1214|Q13163(21) [11213558 , 11656518 , 10427712 , 10074640 , 11427553 , 11338033 , 151194 , 10113978 , 11409972 , 3038522 , 11485656 , 24779724 , 15983966 , 447077 , 11364421 , 17755052 , 176870 , 24889392 , 11314340 , 3025986 , 156414 , 16722836 , 176167 , 11712649 , 208908 , 11667893 , 11234052 , 10127622 , 6918454 , 153999]

1215|Q32MK0(0) [11338033 , 11213558 , 11485656 , 176167 , 151194 , 6918454 , 17755052 , 11409972 , 10427712 , 10113978 , 11712649 , 16722836 , 11314340 , 3025986 , 10074640 , 208908 , 176870 , 11234052 , 11364421 , 11656518 , 24889392 , 24779724 , 153999 , 3038522 , 156414 , 11667893 , 11427553 , 10127622 , 447077 , 15983966]

1216|O75460(39) [10113978 , 10074640 , 3038522 , 11213558 , 15983966 , 11314340 , 16722836 , 176870 , 10127622 , 6918454 , 153999 , 11712649 , 151194 , 208908 , 24779724 , 11427553 , 11409972 , 447077 , 3025986 , 156414 , 11338033 , 11485656 , 10427712 , 11667893 , 176167 , 24889392 , 17755052 , 11656518 , 11234052 , 11364421]

1217|Q56UN5(1) [15983966 , 10427712 , 11234052 , 11712649 , 11213558 , 16722836 , 176870 , 24779724 , 3038522 , 11656518 , 447077 , 10074640 , 153999 , 176167 , 10127622 , 11667893 , 11314340 , 11485656 , 11427553 , 24889392 , 11364421 , 151194 , 11338033 , 156414 , 10113978 , 3025986 , 6918454 , 11409972 , 17755052 , 208908]

1218|Q96Q40(14) [11364421 , 10127622 , 24779724 , 11427553 , 153999 , 11712649 , 10427712 , 176167 , 3038522 , 11409972 , 156414 , 176870 , 11338033 , 11485656 , 3025986 , 6918454 , 11656518 , 151194 , 24889392 , 11314340 , 10074640 , 11234052 , 11213558 , 16722836 , 10113978 , 11667893 , 17755052 , 208908 , 15983966 , 447077]

1219|O95382(4) [151194 , 11667893 , 17755052 , 15983966 , 176870 , 3038522 , 11712649 , 153999 , 10427712 , 6918454 , 11338033 , 11656518 , 447077 , 208908 , 11213558 , 156414 , 10074640 , 11427553 , 11364421 , 16722836 , 3025986 , 11409972 , 10127622 , 11234052 , 24889392 , 11314340 , 11485656 , 176167 , 10113978 , 24779724]

1220|Q9BVE2(0) [151194 , 11338033 , 3038522 , 11364421 , 17755052 , 10427712 , 447077 , 11234052 , 11667893 , 11409972 , 10074640 , 24779724 , 3025986 , 11314340 , 10113978 , 153999 , 156414 , 176870 , 11427553 , 11213558 , 10127622 , 11656518 , 15983966 , 6918454 , 16722836 , 11485656 , 208908 , 11712649 , 176167 , 24889392]

1221|P20794(23) [11409972 , 24889392 , 10427712 , 11427553 , 11712649 , 447077 , 11656518 , 6918454 , 11314340 , 3025986 , 11485656 , 11338033 , 176870 , 156414 , 3038522 , 16722836 , 11667893 , 208908 , 10113978 , 11234052 , 10074640 , 17755052 , 24779724 , 151194 , 10127622 , 11213558 , 176167 , 11364421 , 15983966 , 153999]

1222|Q9Y2U5(8) [11234052 , 16722836 , 176167 , 3038522 , 11338033 , 10127622 , 10074640 , 11427553 , 156414 , 176870 , 24779724 , 151194 , 11364421 , 17755052 , 11409972 , 447077 , 15983966 , 10113978 , 11712649 , 11314340 , 208908 , 11667893 , 11213558 , 24889392 , 11485656 , 6918454 , 3025986 , 11656518 , 10427712 , 153999]

1223|P45985(35) [11234052 , 16722836 , 10074640 , 153999 , 11314340 , 6918454 , 24779724 , 3025986 , 11338033 , 447077 , 176167 , 11427553 , 3038522 , 208908 , 17755052 , 11213558 , 11712649 , 10127622 , 24889392 , 10427712 , 156414 , 11656518 , 11667893 , 10113978 , 11364421 , 15983966 , 11409972 , 160355 , 11485656 , 151194 , 176870]

1224|Q5S007(52) [11712649 , 156414 , 11409972 , 11234052 , 6918454 , 153999 , 15983966 , 176167 , 16722836 , 176870 , 11485656 , 11338033 , 10427712 , 448008 , 11314340 , 151194 , 11364421 , 3038522 , 11667893 , 11656518 , 24779724 , 11427553 , 208908 , 10113978 , 10074640 , 10127622 , 447077 , 17755052 , 3025986 , 24889392 , 11213558]

1225|Q5VZY9(0) [3025986 , 11213558 , 11485656 , 11364421 , 24889392 , 176870 , 160355 , 11409972 , 3038522 , 15983966 , 11338033 , 11667893 , 447077 , 24779724 , 11314340 , 151194 , 176167 , 153999 , 6918454 , 17755052 , 10074640 , 11427553 , 11656518 , 11234052 , 156414 , 208908 , 10427712 , 11712649 , 10127622 , 16722836 , 10113978]

1226|Q9UBF8(1) [10427712 , 3025986 , 11213558 , 11234052 , 11427553 , 6918454 , 11656518 , 24889392 , 24779724 , 156414 , 11667893 , 176167 , 16722836 , 11314340 , 10127622 , 11338033 , 11712649 , 153999 , 11485656 , 17755052 , 11364421 , 447077 , 10113978 , 151194 , 10074640 , 448008 , 11409972 , 3038522 , 176870 , 15983966 , 208908]

1227|Q9BRS2(2) [176870 , 160355 , 11667893 , 11338033 , 11427553 , 11314340 , 156414 , 10113978 , 15983966 , 10427712 , 151194 , 16722836 , 11485656 , 153999 , 3038522 , 10127622 , 176167 , 11409972 , 17755052 , 11234052 , 6918454 , 10074640 , 208908 , 447077 , 11364421 , 11712649 , 24889392 , 11656518 , 11213558 , 3025986 , 24779724]

1228|Q8NEV4(3) [208908 , 11485656 , 11234052 , 11656518 , 3025986 , 10127622 , 447077 , 11427553 , 151194 , 10427712 , 24889392 , 176167 , 160355 , 153999 , 156414 , 10074640 , 6918454 , 11314340 , 11712649 , 17755052 , 11213558 , 24779724 , 11409972 , 3038522 , 11364421 , 15983966 , 11338033 , 10113978 , 176870 , 16722836 , 11667893]

1229|Q99755(2) [24779724 , 11234052 , 6918454 , 176167 , 10127622 , 10113978 , 11712649 , 11213558 , 11338033 , 3038522 , 11409972 , 11427553 , 24889392 , 176870 , 11364421 , 11656518 , 153999 , 11667893 , 447077 , 15983966 , 160355 , 11485656 , 11314340 , 16722836 , 17755052 , 10427712 , 3025986 , 208908 , 10074640 , 151194 , 156414]

1230|Q9C098(8) [10427712 , 208908 , 176167 , 153999 , 160355 , 17755052 , 11364421 , 24889392 , 447077 , 151194 , 11213558 , 10127622 , 10074640 , 16722836 , 10113978 , 156414 , 11234052 , 3038522 , 11409972 , 11667893 , 11656518 , 15983966 , 3025986 , 6918454 , 176870 , 11314340 , 11485656 , 11712649 , 11427553 , 11338033 , 24779724]

1231|Q16512(17) [447077 , 16722836 , 11409972 , 176870 , 156414 , 11427553 , 11364421 , 3025986 , 11485656 , 153999 , 11314340 , 15983966 , 24889392 , 11712649 , 151194 , 11656518 , 208908 , 6918454 , 10127622 , 176167 , 160355 , 3038522 , 11234052 , 17755052 , 10113978 , 10427712 , 24779724 , 11213558 , 11667893 , 11338033 , 10074640]

1232|Q8NE63(0) [24779724 , 448008 , 10427712 , 11213558 , 3025986 , 11427553 , 6918454 , 16722836 , 11656518 , 10113978 , 447077 , 11667893 , 11314340 , 24889392 , 156414 , 11712649 , 153999 , 11485656 , 15983966 , 151194 , 11409972 , 176167 , 10127622 , 11338033 , 11364421 , 10074640 , 17755052 , 208908 , 3038522 , 11234052 , 176870]

1233|Q9Y6R4(9) [10427712 , 11667893 , 10113978 , 11234052 , 176167 , 3038522 , 11364421 , 160355 , 11213558 , 11427553 , 10127622 , 176870 , 208908 , 156414 , 11409972 , 11338033 , 24779724 , 3025986 , 11314340 , 11485656 , 15983966 , 11656518 , 16722836 , 6918454 , 24889392 , 11712649 , 10074640 , 153999 , 151194 , 17755052 , 447077]

1234|Q02779(12) [6918454 , 3025986 , 15983966 , 447077 , 11314340 , 11656518 , 160355 , 176870 , 10113978 , 11234052 , 24889392 , 24779724 , 10127622 , 10427712 , 10074640 , 11667893 , 11409972 , 11338033 , 156414 , 11427553 , 11364421 , 11213558 , 17755052 , 11712649 , 208908 , 3038522 , 153999 , 11485656 , 151194 , 176167 , 16722836]

1235|Q9Y616(31) [11427553 , 10113978 , 11656518 , 6918454 , 11409972 , 11485656 , 16722836 , 160355 , 11314340 , 156414 , 11667893 , 176870 , 17755052 , 15983966 , 11364421 , 11712649 , 10074640 , 151194 , 11338033 , 24779724 , 10127622 , 3025986 , 208908 , 153999 , 10427712 , 176167 , 24889392 , 3038522 , 11213558 , 447077 , 11234052]

1236|P11308(75) [16231 , 70464 , 5918 , 6307 , 3969 , 31729 , 2327 , 119259 , 10621 , 4578 , 10168 , 114811 , 2831167 , 3474 , 658365 , 50248 , 2179 , 5335 , 2794 , 2016 , 2082 , 5593 , 2482 , 2307977 , 327044 , 5510 , 19646 , 2815581 , 13752 , 2132993 , 166553]

1237|Q9UQ88(6) [11667893 , 11364421 , 151194 , 16722836 , 17755052 , 10127622 , 11427553 , 11213558 , 11485656 , 11338033 , 208908 , 11409972 , 10074640 , 153999 , 24779724 , 156414 , 11656518 , 11314340 , 10427712 , 6918454 , 11712649 , 10113978 , 3025986 , 160355 , 11234052 , 176870 , 176167 , 447077 , 3038522 , 15983966 , 24889392]

1238|Q8IY84(3) [11712649 , 156414 , 11485656 , 11667893 , 15983966 , 10127622 , 11409972 , 11364421 , 11213558 , 24889392 , 10074640 , 176167 , 176870 , 151194 , 10113978 , 208908 , 11427553 , 17755052 , 11656518 , 447077 , 11314340 , 6918454 , 10427712 , 11338033 , 24779724 , 3025986 , 153999 , 16722836 , 11442891 , 11234052 , 3038522]

1239|Q12851(0) [11314340 , 176167 , 447077 , 17755052 , 11485656 , 3038522 , 10074640 , 24779724 , 15983966 , 153999 , 11712649 , 11364421 , 176870 , 10427712 , 16722836 , 11338033 , 151194 , 11667893 , 448008 , 156414 , 11409972 , 24889392 , 11234052 , 208908 , 10127622 , 3025986 , 10113978 , 11427553 , 11213558 , 11656518 , 6918454]

1240|Q9NRM7(27) [3025986 , 11338033 , 151194 , 176167 , 10427712 , 17755052 , 11667893 , 176870 , 447077 , 11485656 , 11314340 , 24779724 , 11213558 , 11364421 , 11712649 , 11409972 , 11656518 , 11234052 , 11427553 , 160355 , 156414 , 3038522 , 10074640 , 10127622 , 10113978 , 24889392 , 16722836 , 6918454 , 15983966 , 208908 , 153999]

1241|V9GXZ4(0) [11427553 , 176870 , 17755052 , 11656518 , 11409972 , 11712649 , 15983966 , 11338033 , 24779724 , 3025986 , 160355 , 16722836 , 156414 , 10427712 , 10127622 , 3038522 , 10074640 , 153999 , 11485656 , 11234052 , 6918454 , 11667893 , 447077 , 11364421 , 11213558 , 176167 , 151194 , 10113978 , 24889392 , 208908 , 11314340]

1242|Q8N752(0) [10127622 , 17755052 , 10113978 , 10427712 , 447077 , 153999 , 11427553 , 208908 , 6918454 , 24779724 , 11667893 , 3038522 , 15983966 , 16722836 , 11314340 , 11409972 , 10074640 , 24889392 , 151194 , 11656518 , 11712649 , 160355 , 11213558 , 3025986 , 11485656 , 11234052 , 11338033 , 11364421 , 176870 , 176167 , 156414]

1243|P21860(115) [11485656 , 447077 , 11314340 , 208908 , 151194 , 24889392 , 6918454 , 11234052 , 11338033 , 11488320 , 3025986 , 153999 , 15983966 , 17755052 , 11667893 , 10074640 , 16722836 , 3038522 , 10113978 , 10427712 , 11427553 , 10127622 , 11656518 , 24779724 , 11364421 , 156414 , 176167 , 176870 , 11409972 , 11213558 , 11712649]

1244|O94921(7) [11712649 , 11409972 , 176870 , 11314340 , 153999 , 11364421 , 24889392 , 447077 , 11667893 , 10127622 , 15983966 , 10113978 , 11427553 , 160355 , 11485656 , 16722836 , 3025986 , 10427712 , 151194 , 24779724 , 11234052 , 156414 , 6918454 , 11213558 , 11656518 , 17755052 , 10074640 , 176167 , 208908 , 3038522 , 11338033]

1245|Q6P3R8(0) [15983966 , 24779724 , 11364421 , 447077 , 208908 , 156414 , 3025986 , 10427712 , 10127622 , 11338033 , 11667893 , 160355 , 6918454 , 11656518 , 3038522 , 11314340 , 11234052 , 11409972 , 176167 , 10113978 , 16722836 , 10074640 , 11213558 , 11485656 , 11427553 , 24889392 , 151194 , 176870 , 17755052 , 11712649 , 153999]

1246|Q13705(22) [10427712 , 16722836 , 10127622 , 11409972 , 10113978 , 151194 , 11427553 , 11234052 , 15983966 , 11213558 , 208908 , 156414 , 11314340 , 11667893 , 24889392 , 11364421 , 3025986 , 176167 , 3038522 , 153999 , 11485656 , 17755052 , 10074640 , 176870 , 11712649 , 6918454 , 11338033 , 11656518 , 447077 , 24779724 , 160355]

1247|Q6DT37(0) [151194 , 24889392 , 3025986 , 176870 , 160355 , 11338033 , 176167 , 6918454 , 17755052 , 11667893 , 11485656 , 3038522 , 11656518 , 24779724 , 15983966 , 11213558 , 447077 , 10113978 , 11314340 , 11234052 , 11427553 , 208908 , 16722836 , 11364421 , 11712649 , 156414 , 10074640 , 10427712 , 11409972 , 10127622 , 153999]

1248|P35590(31) [17755052 , 10427712 , 11427553 , 11485656 , 447077 , 11667893 , 3038522 , 24889392 , 10074640 , 208908 , 11234052 , 11338033 , 6918454 , 11712649 , 3025986 , 10113978 , 11409972 , 10127622 , 160355 , 15983966 , 11213558 , 151194 , 11314340 , 156414 , 24779724 , 176870 , 153999 , 16722836 , 11364421 , 11656518 , 176167]

1249|P27037(28) [10427712 , 208908 , 176870 , 24889392 , 3025986 , 6918454 , 11427553 , 17755052 , 11213558 , 10074640 , 153999 , 447077 , 3038522 , 151194 , 10113978 , 11364421 , 11485656 , 11314340 , 156414 , 11338033 , 11656518 , 160355 , 16722836 , 10127622 , 176167 , 15983966 , 24779724 , 11712649 , 11667893 , 11409972 , 11234052]

1250|Q9BY41(22) [6445533 , 88129 , 264 , 56683746 , 56661654 , 419176 , 56677092 , 208908 , 5352062 , 2466 , 6918638 , 56680386 , 9865515 , 10309899 , 24756910 , 449096 , 10313 , 4261 , 53340666 , 56673753 , 11609955 , 9804992 , 6918837 , 10379137 , 11538455 , 4996 , 5311 , 2746 , 56663366 , 53345880 , 3994]

1251|O14730(4) [160355 , 10074640 , 153999 , 24779724 , 11427553 , 156414 , 10427712 , 176870 , 447077 , 11314340 , 11712649 , 15983966 , 11338033 , 3038522 , 11656518 , 11667893 , 11234052 , 208908 , 11364421 , 17755052 , 11213558 , 10113978 , 11409972 , 24889392 , 11485656 , 10127622 , 3025986 , 6918454 , 176167 , 16722836 , 151194]

1252|P22694(13) [6918454 , 160355 , 10074640 , 17755052 , 24889392 , 11712649 , 11314340 , 3038522 , 24779724 , 10113978 , 11213558 , 11364421 , 176167 , 151194 , 16722836 , 447077 , 11409972 , 11656518 , 11667893 , 176870 , 208908 , 10427712 , 156414 , 3025986 , 11234052 , 11485656 , 11427553 , 11338033 , 10127622 , 153999 , 15983966]

1253|Q13873(56) [176870 , 11667893 , 151194 , 176167 , 156414 , 10427712 , 6918454 , 15983966 , 11314340 , 24889392 , 11485656 , 10113978 , 11234052 , 153999 , 3038522 , 10127622 , 17755052 , 16722836 , 11409972 , 10074640 , 11364421 , 11213558 , 11656518 , 208908 , 447077 , 3025986 , 11427553 , 160355 , 11338033 , 24779724 , 11712649]

1254|Q16659(7) [11667893 , 10427712 , 11427553 , 16722836 , 15983966 , 11364421 , 160355 , 3025986 , 11338033 , 11409972 , 11234052 , 10074640 , 3038522 , 156414 , 11485656 , 153999 , 11712649 , 11656518 , 17755052 , 11314340 , 6918454 , 151194 , 10127622 , 208908 , 447077 , 11213558 , 176870 , 176167 , 24889392 , 24779724 , 10113978]

1255|P31152(2) [11338033 , 10113978 , 208908 , 160355 , 151194 , 10074640 , 10127622 , 11234052 , 11427553 , 17755052 , 11485656 , 24889392 , 3038522 , 11712649 , 11213558 , 176870 , 156414 , 16722836 , 15983966 , 176167 , 11409972 , 11656518 , 24779724 , 11364421 , 10427712 , 11667893 , 11314340 , 6918454 , 153999 , 447077 , 3025986]

1256|Q8NI60(3) [11667893 , 176870 , 15983966 , 24889392 , 10074640 , 11364421 , 11314340 , 176167 , 11338033 , 11234052 , 11427553 , 11712649 , 153999 , 10127622 , 24779724 , 156414 , 17755052 , 10427712 , 3038522 , 160355 , 16722836 , 11656518 , 10113978 , 447077 , 11213558 , 6918454 , 11485656 , 3025986 , 151194 , 11409972 , 208908]

1257|Q13164(38) [176167 , 11234052 , 153999 , 11213558 , 15983966 , 6918454 , 447077 , 10127622 , 10427712 , 11314340 , 176870 , 151194 , 11427553 , 11338033 , 10113978 , 24889392 , 156414 , 3038522 , 11712649 , 11485656 , 16722836 , 24779724 , 11667893 , 160355 , 208908 , 11364421 , 11656518 , 17755052 , 3025986 , 10074640 , 11409972]

1258|Q8WXR4(1) [10074640 , 208908 , 11667893 , 156414 , 6918454 , 11656518 , 160355 , 11427553 , 11314340 , 11213558 , 24779724 , 24889392 , 11485656 , 10113978 , 176870 , 17755052 , 151194 , 11234052 , 447077 , 3038522 , 15983966 , 176167 , 153999 , 11338033 , 11364421 , 3025986 , 10427712 , 10127622 , 11409972 , 16722836 , 11712649]

1259|Q9NRP7(0) [3025986 , 3038522 , 153999 , 10427712 , 17755052 , 11409972 , 11314340 , 11338033 , 160355 , 11712649 , 11364421 , 11427553 , 11485656 , 11234052 , 10074640 , 176870 , 151194 , 16722836 , 10127622 , 10113978 , 208908 , 15983966 , 11656518 , 447077 , 176167 , 6918454 , 11667893 , 11213558 , 24779724 , 156414 , 24889392]

1260|Q9NYL2(22) [24779724 , 11234052 , 3038522 , 11427553 , 208908 , 176167 , 11364421 , 151194 , 11338033 , 10074640 , 176870 , 153999 , 11409972 , 11712649 , 11656518 , 15983966 , 6918454 , 10127622 , 11667893 , 3025986 , 16722836 , 17755052 , 11485656 , 11213558 , 447077 , 10427712 , 10113978 , 156414 , 160355 , 11314340 , 24889392]

1261|Q86YV6(0) [176870 , 11234052 , 10113978 , 176167 , 11427553 , 11656518 , 208908 , 11364421 , 153999 , 3038522 , 10074640 , 15983966 , 11338033 , 11485656 , 156414 , 17755052 , 11667893 , 11213558 , 24889392 , 16722836 , 151194 , 6918454 , 24779724 , 10427712 , 11409972 , 11314340 , 3025986 , 447077 , 160355 , 10127622 , 11712649]

1262|Q9BX84(12) [3025986 , 208908 , 888 , 176870 , 156414 , 10127622 , 24889392 , 11213558 , 11656518 , 3038522 , 11364421 , 11712649 , 17755052 , 11314340 , 11485656 , 151194 , 447077 , 10113978 , 15983966 , 11338033 , 11427553 , 153999 , 11409972 , 6918454 , 10427712 , 24779724 , 11667893 , 16722836 , 10074640 , 176167 , 11234052]

1263|Q8NFD2(19) [11234052 , 153999 , 24779724 , 447077 , 24889392 , 6918454 , 10127622 , 11409972 , 11338033 , 10427712 , 11667893 , 156414 , 15983966 , 11427553 , 11314340 , 17755052 , 11712649 , 176167 , 16722836 , 10074640 , 11656518 , 10113978 , 11213558 , 208908 , 11364421 , 3025986 , 160355 , 151194 , 176870 , 3038522 , 11485656]

1264|O14578(12) [3038522 , 160355 , 156414 , 11427553 , 10427712 , 11213558 , 208908 , 10113978 , 24889392 , 11314340 , 11234052 , 10127622 , 11338033 , 16722836 , 447077 , 6918454 , 11656518 , 11667893 , 176870 , 10074640 , 11409972 , 17755052 , 11712649 , 3025986 , 11485656 , 24779724 , 153999 , 151194 , 15983966 , 11364421 , 176167]

1265|Q8IVH8(14) [3025986 , 11234052 , 153999 , 11213558 , 16722836 , 11364421 , 24779724 , 11427553 , 24889392 , 10074640 , 11656518 , 10427712 , 156414 , 176167 , 17755052 , 447077 , 15983966 , 11409972 , 11314340 , 11485656 , 10113978 , 151194 , 11338033 , 6918454 , 10127622 , 11712649 , 11667893 , 208908 , 3038522 , 160355 , 176870]

1266|Q13546(59) [10074640 , 17755052 , 10127622 , 176167 , 176870 , 24779724 , 10113978 , 3025986 , 11485656 , 15983966 , 447077 , 160355 , 24889392 , 11213558 , 151194 , 11364421 , 11667893 , 3038522 , 11234052 , 11409972 , 16722836 , 11338033 , 153999 , 11712649 , 11314340 , 11427553 , 156414 , 10427712 , 208908 , 6918454 , 11656518]

1267|P78356(2) [3025986 , 11427553 , 24889392 , 10074640 , 11409972 , 11213558 , 151194 , 10127622 , 176870 , 156414 , 11712649 , 15983966 , 6918454 , 3038522 , 11667893 , 208908 , 11234052 , 11485656 , 10427712 , 447077 , 24779724 , 10113978 , 16722836 , 11314340 , 153999 , 17755052 , 11364421 , 11338033 , 11656518 , 160355 , 176167]

1268|P49336(19) [11656518 , 16722836 , 11427553 , 11409972 , 3038522 , 15983966 , 11213558 , 447077 , 11485656 , 10427712 , 3025986 , 24889392 , 11364421 , 10113978 , 151194 , 176870 , 24779724 , 17755052 , 208908 , 11314340 , 160355 , 11712649 , 176167 , 10127622 , 11667893 , 10074640 , 11338033 , 11234052 , 153999 , 6918454 , 156414]

1269|Q9H093(4) [24889392 , 156414 , 447077 , 10074640 , 11234052 , 11364421 , 3025986 , 11656518 , 11712649 , 16722836 , 151194 , 11314340 , 17755052 , 10127622 , 11667893 , 208908 , 153999 , 176870 , 15983966 , 160355 , 3038522 , 11213558 , 6918454 , 11427553 , 10113978 , 11338033 , 24779724 , 11409972 , 11485656 , 10427712 , 176167]

1270|Q00537(1) [11712649 , 3038522 , 11338033 , 10427712 , 11667893 , 10113978 , 6918454 , 151194 , 3025986 , 447077 , 208908 , 10074640 , 11427553 , 176870 , 160355 , 11234052 , 17755052 , 24889392 , 11364421 , 176167 , 11656518 , 15983966 , 11485656 , 24779724 , 153999 , 11409972 , 11213558 , 16722836 , 10127622 , 11314340 , 156414]

1271|O95835(25) [16722836 , 160355 , 11409972 , 11234052 , 10113978 , 11364421 , 3025986 , 11427553 , 156414 , 153999 , 10127622 , 208908 , 11338033 , 176870 , 11314340 , 11712649 , 176167 , 17755052 , 151194 , 24889392 , 11667893 , 11656518 , 6918454 , 447077 , 24779724 , 3038522 , 11213558 , 10074640 , 11485656 , 10427712 , 15983966]

1272|Q9BWU1(13) [176870 , 156414 , 160355 , 24889392 , 11409972 , 10427712 , 447077 , 11338033 , 10113978 , 15983966 , 11485656 , 6918454 , 11213558 , 10074640 , 10127622 , 208908 , 11667893 , 153999 , 17755052 , 11364421 , 176167 , 3038522 , 16722836 , 11314340 , 11427553 , 11712649 , 24779724 , 151194 , 11234052 , 3025986 , 11656518]

1273|Q9H1R3(1) [11213558 , 3038522 , 3025986 , 17755052 , 10113978 , 6918454 , 10074640 , 11656518 , 153999 , 176870 , 447077 , 151194 , 11409972 , 11485656 , 16722836 , 24889392 , 15983966 , 11667893 , 10127622 , 176167 , 208908 , 11314340 , 11712649 , 11364421 , 156414 , 10427712 , 11234052 , 24779724 , 11427553 , 160355 , 11338033]

1274|Q96D53(4) [11656518 , 176167 , 151194 , 160355 , 3025986 , 11213558 , 11712649 , 17755052 , 11314340 , 16722836 , 11427553 , 10427712 , 447966 , 24889392 , 11667893 , 15983966 , 11409972 , 10113978 , 3038522 , 208908 , 153999 , 11485656 , 156414 , 11234052 , 11338033 , 176870 , 24779724 , 10127622 , 447077 , 11364421 , 10074640 , 6918454]

1275|O14976(4) [10427712 , 153999 , 11409972 , 24889392 , 11338033 , 11485656 , 11656518 , 11364421 , 3025986 , 151194 , 208908 , 11667893 , 156414 , 3038522 , 16722836 , 160355 , 17755052 , 176167 , 11234052 , 6918454 , 15983966 , 11213558 , 11427553 , 447966 , 24779724 , 176870 , 10074640 , 447077 , 11314340 , 10113978 , 11712649 , 10127622]

1276|Q96NX5(1) [151194 , 11409972 , 10113978 , 153999 , 3025986 , 10427712 , 447077 , 11427553 , 11712649 , 11234052 , 24779724 , 11213558 , 24889392 , 3038522 , 11314340 , 16722836 , 6918454 , 10127622 , 11656518 , 11338033 , 15983966 , 10074640 , 11364421 , 11667893 , 156414 , 176167 , 208908 , 160355 , 176870 , 17755052 , 11485656 , 447966]

1277|Q9H3Y6(2) [11213558 , 3038522 , 10427712 , 10113978 , 151194 , 16722836 , 10074640 , 11485656 , 11234052 , 11427553 , 17755052 , 176870 , 11409972 , 156414 , 11364421 , 11314340 , 24779724 , 10127622 , 153999 , 448008 , 11712649 , 208908 , 24889392 , 176167 , 11656518 , 447077 , 3025986 , 15983966 , 6918454 , 160355 , 11338033 , 11667893]

1278|Q86UX6(1) [15983966 , 10127622 , 17755052 , 11213558 , 10113978 , 160355 , 447966 , 176870 , 3025986 , 11338033 , 447077 , 156414 , 11427553 , 10074640 , 11364421 , 208908 , 24889392 , 11314340 , 176167 , 3038522 , 11667893 , 11409972 , 11485656 , 153999 , 16722836 , 151194 , 11656518 , 24779724 , 6918454 , 11234052 , 11712649 , 10427712]

1279|Q96PY6(19) [17755052 , 6918454 , 15983966 , 447077 , 153999 , 11485656 , 156414 , 24889392 , 176167 , 448008 , 11409972 , 151194 , 3025986 , 10427712 , 10113978 , 11427553 , 11667893 , 11656518 , 24779724 , 11364421 , 16722836 , 208908 , 10074640 , 11712649 , 11314340 , 3038522 , 11234052 , 176870 , 11338033 , 10127622 , 160355 , 11213558]

1280|Q9Y2H1(2) [160355 , 10127622 , 17755052 , 208908 , 156414 , 11409972 , 24889392 , 153999 , 3038522 , 447966 , 11234052 , 11667893 , 176870 , 16722836 , 10427712 , 15983966 , 11485656 , 11427553 , 11213558 , 10113978 , 151194 , 176167 , 11364421 , 11338033 , 6918454 , 11314340 , 11712649 , 10074640 , 24779724 , 3025986 , 11656518 , 447077]

1281|Q9UKI8(19) [160355 , 3038522 , 11364421 , 24779724 , 11712649 , 11427553 , 153999 , 176870 , 17755052 , 11409972 , 156414 , 176167 , 6918454 , 11656518 , 11314340 , 151194 , 24889392 , 10113978 , 11338033 , 11667893 , 11234052 , 11485656 , 10127622 , 3025986 , 11213558 , 447966 , 208908 , 447077 , 16722836 , 10074640 , 10427712 , 15983966]

1282|Q9Y4K4(4) [6918454 , 11427553 , 447966 , 10074640 , 15983966 , 447077 , 11667893 , 17755052 , 10113978 , 11656518 , 176870 , 11213558 , 11338033 , 11234052 , 10427712 , 208908 , 3038522 , 176167 , 16722836 , 24889392 , 11485656 , 11364421 , 160355 , 153999 , 11712649 , 151194 , 11409972 , 156414 , 10127622 , 24779724 , 3025986 , 11314340]

1283|Q9NSY1(1) [11427553 , 10427712 , 10127622 , 153999 , 10074640 , 176167 , 11712649 , 11314340 , 11234052 , 6918454 , 11656518 , 24779724 , 3025986 , 15983966 , 151194 , 447077 , 11485656 , 160355 , 11213558 , 16722836 , 11364421 , 17755052 , 11667893 , 447966 , 11338033 , 208908 , 3038522 , 156414 , 10113978 , 11409972 , 24889392 , 176870]

1284|Q16790(79) [16122590 , 389641 , 216468 , 5287541 , 11967800 , 45028895 , 10112 , 169682 , 12066940 , 57413968 , 11117301 , 1003 , 36811 , 3295 , 12066941 , 72139 , 11147931 , 5356 , 6852128 , 3647 , 159772 , 14611919 , 643477 , 2343 , 11367571 , 19772348 , 3639 , 2732 , 76509 , 20368972 , 6307 , 462919]

1285|Q9Y463(15) [24889392 , 15983966 , 6918454 , 11712649 , 160355 , 11338033 , 11667893 , 10427712 , 176870 , 11314340 , 11409972 , 448008 , 24779724 , 10127622 , 3038522 , 10074640 , 11364421 , 156414 , 11485656 , 11656518 , 17755052 , 447077 , 153999 , 11213558 , 11427553 , 10113978 , 3025986 , 151194 , 11234052 , 208908 , 176167 , 16722836]

1286|Q92918(11) [160355 , 17755052 , 176167 , 11213558 , 16722836 , 11234052 , 11338033 , 24889392 , 3025986 , 11314340 , 176870 , 11485656 , 11409972 , 153999 , 447966 , 10113978 , 11656518 , 11667893 , 11364421 , 24779724 , 11427553 , 3038522 , 447077 , 10427712 , 208908 , 11712649 , 6918454 , 151194 , 156414 , 10074640 , 15983966 , 10127622]

1287|P63096(3) [11625733 , 44592882 , 11690308 , 44592949 , 44592924 , 44592884 , 11595656 , 44592923 , 44592887 , 44592925 , 44592953 , 11641883 , 11641511 , 11532642 , 44592824 , 11689506 , 11532035 , 11553599 , 44592825 , 11660784 , 44592948 , 11675664 , 44592903 , 11661302 , 44592821 , 11674801 , 44592883 , 44592951 , 44592858 , 44592822 , 11696933 , 11561507]

1288|P36507(52) [11409972 , 10127622 , 3038522 , 24779724 , 11427553 , 156414 , 10427712 , 11234052 , 11656518 , 11213558 , 11712649 , 11485656 , 151194 , 11364421 , 447077 , 208908 , 176167 , 176870 , 16722836 , 11314340 , 10113978 , 17755052 , 11707110 , 11667893 , 6918454 , 15983966 , 160355 , 11338033 , 153999 , 24889392 , 10074640 , 3025986]

1289|P37023(73) [176167 , 6918454 , 11314340 , 11364421 , 11712649 , 11427553 , 11234052 , 11213558 , 208908 , 16722836 , 11485656 , 17755052 , 156414 , 151194 , 176870 , 11656518 , 3038522 , 10127622 , 10113978 , 447077 , 160355 , 10074640 , 153999 , 24779724 , 11409972 , 3025986 , 15983966 , 24889392 , 11338033 , 10427712 , 11667893 , 447966]

1290|Q2M2I8(5) [11234052 , 3025986 , 153999 , 11364421 , 11314340 , 11712649 , 10113978 , 17755052 , 24889392 , 11409972 , 176167 , 156414 , 11667893 , 16722836 , 10127622 , 447966 , 11485656 , 208908 , 160355 , 447077 , 15983966 , 151194 , 176870 , 11338033 , 11656518 , 11427553 , 11213558 , 10074640 , 6918454 , 24779724 , 10427712 , 3038522]

1291|P39877(8) [45482342 , 44563042 , 45482332 , 44563073 , 51346870 , 16118318 , 10067704 , 45482340 , 16118461 , 25166397 , 4670 , 16118459 , 45482337 , 16118319 , 45482339 , 44562726 , 44563074 , 44562673 , 45482338 , 44241583 , 45482333 , 16118320 , 45482334 , 44562729 , 44563075 , 155815 , 44562672 , 45482335 , 53323583 , 44563043 , 53319614 , 44563072]

1292|P37173(190) [3038522 , 10127622 , 16722836 , 447077 , 24779724 , 176167 , 15983966 , 10113978 , 17755052 , 11656518 , 160355 , 151194 , 11712649 , 11364421 , 11213558 , 208908 , 3025986 , 176870 , 11338033 , 11667893 , 6918454 , 10427712 , 153999 , 447966 , 11314340 , 11427553 , 11409972 , 11485656 , 24889392 , 10074640 , 11234052 , 156414]

1293|Q9HAZ1(0) [160355 , 10074640 , 11485656 , 153999 , 10427712 , 10113978 , 208908 , 10127622 , 156414 , 11234052 , 11427553 , 447077 , 17755052 , 3025986 , 11656518 , 447966 , 15983966 , 6918454 , 11338033 , 3038522 , 151194 , 11213558 , 16722836 , 176167 , 24889392 , 11667893 , 11712649 , 176870 , 11409972 , 11364421 , 11314340 , 24779724]

1294|Q9H2G2(6) [10427712 , 11338033 , 176167 , 151194 , 24889392 , 11712649 , 11409972 , 153999 , 6918454 , 17755052 , 11667893 , 3038522 , 160355 , 447966 , 11656518 , 11213558 , 11314340 , 10074640 , 15983966 , 11485656 , 208908 , 24779724 , 447077 , 11234052 , 11364421 , 10113978 , 11427553 , 156414 , 16722836 , 10127622 , 3025986 , 176870]

1295|O75716(2) [447077 , 153999 , 11667893 , 11427553 , 11712649 , 160355 , 24889392 , 10113978 , 3025986 , 11409972 , 17755052 , 151194 , 10427712 , 11314340 , 10127622 , 208908 , 11485656 , 11364421 , 10074640 , 11234052 , 176870 , 11338033 , 11213558 , 24779724 , 16722836 , 3038522 , 156414 , 15983966 , 176167 , 447966 , 6918454 , 11656518]

1296|Q04771(41) [208908 , 11213558 , 3025986 , 10427712 , 11364421 , 176870 , 10127622 , 24779724 , 15983966 , 11427553 , 24889392 , 10074640 , 11234052 , 156414 , 11338033 , 17755052 , 151194 , 3038522 , 11409972 , 160355 , 447966 , 11485656 , 153999 , 10113978 , 11656518 , 11712649 , 11667893 , 176167 , 6918454 , 16722836 , 11314340 , 447077]

1297|Q9UBN7(59) [9804992 , 6918638 , 50908800 , 88129 , 52949685 , 10309899 , 5311 , 24756910 , 419176 , 2466 , 5352062 , 11844892 , 11844893 , 4261 , 52947269 , 11609955 , 52947250 , 53340666 , 52948832 , 52950031 , 6445533 , 11538455 , 11844891 , 10313 , 208908 , 6918837 , 4996 , 9865515 , 3994 , 2746 , 49855250 , 23640756]

1298|Q9NY57(4) [24779724 , 176870 , 11314340 , 24889392 , 11338033 , 3025986 , 11213558 , 15983966 , 208908 , 10427712 , 6918454 , 11656518 , 10127622 , 17755052 , 156414 , 11234052 , 16722836 , 11712649 , 153999 , 11667893 , 151194 , 176167 , 3038522 , 10113978 , 447077 , 10074640 , 11409972 , 447966 , 11427553 , 11485656 , 11364421 , 160355]

1299|Q9UF33(5) [447966 , 176167 , 11712649 , 10427712 , 10074640 , 151194 , 447077 , 11213558 , 11338033 , 11409972 , 15983966 , 6918454 , 11667893 , 3038522 , 10127622 , 17755052 , 3025986 , 11485656 , 10113978 , 11364421 , 153999 , 16722836 , 11656518 , 11234052 , 160355 , 24779724 , 11427553 , 176870 , 11314340 , 208908 , 156414 , 24889392]

1300|P36894(50) [6918454 , 10113978 , 24779724 , 10427712 , 11712649 , 10074640 , 208908 , 153999 , 11234052 , 24889392 , 160355 , 11427553 , 11364421 , 11667893 , 3025986 , 11338033 , 10127622 , 176167 , 11213558 , 156414 , 17755052 , 11409972 , 16722836 , 11656518 , 447077 , 11485656 , 3038522 , 11314340 , 447966 , 176870 , 15983966 , 151194]

1301|Q9P2K8(17) [11338033 , 447077 , 153999 , 447966 , 10127622 , 10074640 , 176167 , 11409972 , 15983966 , 151194 , 11234052 , 11656518 , 160355 , 11364421 , 3025986 , 24779724 , 156414 , 17755052 , 11667893 , 3038522 , 176870 , 16722836 , 10113978 , 11712649 , 11213558 , 10427712 , 208908 , 11427553 , 11485656 , 11314340 , 24889392 , 6918454]

1302|Q9UKE5(10) [11712649 , 11213558 , 447966 , 11234052 , 176167 , 10113978 , 11667893 , 11656518 , 15983966 , 3025986 , 10427712 , 16722836 , 11338033 , 11314340 , 10074640 , 24779724 , 11427553 , 208908 , 447077 , 17755052 , 11364421 , 10127622 , 160355 , 6918454 , 24889392 , 11409972 , 151194 , 3038522 , 11485656 , 153999 , 176870 , 156414]

1303|Q13237(6) [10074640 , 10127622 , 11667893 , 17755052 , 3038522 , 15983966 , 176167 , 153999 , 16722836 , 11427553 , 24779724 , 11314340 , 208908 , 151194 , 11656518 , 11364421 , 11409972 , 11712649 , 176870 , 10113978 , 448008 , 156414 , 160355 , 3025986 , 11485656 , 24889392 , 11338033 , 11234052 , 447077 , 10427712 , 11213558 , 6918454]

1304|O15118(82) [10219 , 1720828 , 2197 , 107985 , 5074 , 14369 , 1608140 , 17113 , 1552036 , 4380 , 5405 , 5289501 , 42725 , 65758 , 2161 , 1599306 , 4122 , 1580955 , 166553 , 1878823 , 3503 , 24817194 , 10206 , 162834 , 41684 , 3240818 , 3455 , 1568843 , 19910 , 19646 , 1238 , 680935]

1305|Q13233(47) [11213558 , 16666708 , 10074640 , 10127622 , 17755052 , 11667893 , 176167 , 16722836 , 3038522 , 153999 , 11427553 , 24779724 , 10172827 , 11314340 , 11656518 , 151194 , 11364421 , 11234052 , 176870 , 11409972 , 11712649 , 156414 , 10113978 , 447077 , 24889392 , 11338033 , 15983966 , 3025986 , 11485656 , 208908 , 10427712 , 6918454]

1306|Q99640(6) [153999 , 15983966 , 447077 , 11234052 , 10127622 , 6918454 , 156414 , 10074640 , 24779724 , 447966 , 16722836 , 3038522 , 176167 , 11712649 , 10427712 , 10113978 , 151194 , 17755052 , 11409972 , 11656518 , 160355 , 3025986 , 11338033 , 176870 , 11427553 , 208908 , 11364421 , 11213558 , 11667893 , 11485656 , 24889392 , 11314340]

1307|P53671(13) [15983966 , 17755052 , 447077 , 176167 , 11314340 , 447966 , 11667893 , 11712649 , 11338033 , 6918454 , 10074640 , 11234052 , 208908 , 156414 , 24779724 , 10127622 , 11213558 , 10427712 , 16722836 , 3038522 , 24889392 , 176870 , 11364421 , 153999 , 11485656 , 11409972 , 3025986 , 10113978 , 151194 , 11656518 , 160355 , 11427553]

1308|Q13131(72) [11656518 , 16722836 , 6918454 , 176167 , 11338033 , 11667893 , 447966 , 11409972 , 151194 , 176870 , 17755052 , 11213558 , 11314340 , 11234052 , 447077 , 11364421 , 10427712 , 15983966 , 10113978 , 24779724 , 208908 , 160355 , 11712649 , 10074640 , 11485656 , 153999 , 10127622 , 11427553 , 3038522 , 448008 , 24889392 , 156414 , 3025986]

1309|P21918(10) [47811 , 3389 , 68950 , 3372 , 1615 , 59227 , 107930 , 681 , 1547484 , 11154555 , 5265 , 16 , 31101 , 42601552 , 2818 , 1355 , 6005 , 115237 , 3822 , 3559 , 37459 , 119828 , 10624 , 18104 , 5281881 , 2726 , 2159 , 9860294 , 28864 , 5452 , 54746 , 12454 , 119570]

1310|P50613(22) [24889392 , 24779724 , 11364421 , 153999 , 208908 , 11712649 , 176870 , 156414 , 11338033 , 160355 , 16722836 , 11409972 , 3025986 , 11213558 , 15983966 , 11656518 , 6918454 , 11667893 , 10113978 , 11427553 , 11314340 , 11285002 , 17755052 , 11485656 , 3038522 , 6918852 , 11234052 , 176167 , 447077 , 10427712 , 10127622 , 151194 , 10074640]

1311|P29376(5) [447077 , 11314340 , 176870 , 11712649 , 15983966 , 153999 , 11427553 , 151194 , 16722836 , 11656518 , 6918454 , 156414 , 11485656 , 3038522 , 208908 , 11409972 , 3025986 , 176167 , 10074640 , 11667893 , 10427712 , 24889392 , 17755052 , 447966 , 11234052 , 11364421 , 24779724 , 10127622 , 10113978 , 448008 , 11213558 , 160355 , 11338033]

1312|Q96L34(6) [447077 , 11234052 , 447966 , 153999 , 24889392 , 6918454 , 11409972 , 10074640 , 208908 , 151194 , 16722836 , 3025986 , 176167 , 17755052 , 11656518 , 160355 , 24779724 , 10113978 , 11712649 , 11364421 , 11314340 , 156414 , 11485656 , 3038522 , 176870 , 10427712 , 448008 , 11427553 , 11213558 , 11338033 , 11667893 , 15983966 , 10127622]

1313|O00444(29) [3038522 , 11213558 , 24889392 , 176167 , 11667893 , 10127622 , 11610113 , 10427712 , 156414 , 153999 , 11485656 , 16722836 , 11656518 , 11234052 , 24779724 , 160355 , 11364421 , 10113978 , 176870 , 10074640 , 11409972 , 11314340 , 3025986 , 151194 , 11338033 , 15983966 , 447077 , 447966 , 11427553 , 11712649 , 208908 , 17755052 , 6918454]

1314|P49759(2) [24779724 , 208908 , 11364421 , 11667893 , 176167 , 11234052 , 11409972 , 16722836 , 11338033 , 3025986 , 6918454 , 17755052 , 4564 , 10427712 , 447966 , 11656518 , 10127622 , 447077 , 11485656 , 153999 , 11213558 , 160355 , 156414 , 11712649 , 3038522 , 10113978 , 11314340 , 11427553 , 24889392 , 151194 , 10074640 , 176870 , 15983966]

1315|Q8TD19(1) [156414 , 447077 , 160355 , 176167 , 11234052 , 17755052 , 11667893 , 10113978 , 11409972 , 176870 , 153999 , 3038522 , 24889392 , 16722836 , 11712649 , 11427553 , 11338033 , 15983966 , 11656518 , 6918454 , 11485656 , 24779724 , 447966 , 11213558 , 208908 , 151194 , 11314340 , 10074640 , 10127622 , 3025986 , 448008 , 11364421 , 10427712]

1316|Q9H0K1(10) [448008 , 24889392 , 11314340 , 3038522 , 11427553 , 17755052 , 156414 , 151194 , 11656518 , 24779724 , 6918454 , 11234052 , 10127622 , 176167 , 11213558 , 11712649 , 447077 , 10113978 , 16722836 , 10074640 , 160355 , 15983966 , 176870 , 10427712 , 208908 , 11409972 , 11364421 , 447966 , 3025986 , 11485656 , 11338033 , 11667893 , 153999]

1317|Q9ULX7(8) [3295 , 69809 , 6852128 , 5287541 , 20184392 , 389641 , 216468 , 76509 , 4100 , 44424363 , 11117301 , 14611919 , 169682 , 44424366 , 11967800 , 44424364 , 15729847 , 5356 , 19772348 , 25258361 , 2732 , 68844 , 72139 , 96876 , 25258363 , 5284549 , 12066941 , 6307 , 12066940 , 25258360 , 16122590 , 36811 , 1986]

1318|Q9P289(6) [24889392 , 11234052 , 11485656 , 3025986 , 156414 , 160355 , 153999 , 151194 , 15983966 , 10074640 , 11667893 , 11409972 , 208908 , 11656518 , 10427712 , 11364421 , 16722836 , 10127622 , 3038522 , 176167 , 17755052 , 176870 , 448008 , 24779724 , 10113978 , 11712649 , 11314340 , 6918454 , 11338033 , 11213558 , 447966 , 447077 , 11427553]

1319|Q13470(2) [11338033 , 11485656 , 11427553 , 3025986 , 11667893 , 447966 , 16722836 , 11314340 , 11656518 , 11364421 , 11234052 , 10113978 , 151194 , 208908 , 160355 , 3038522 , 176167 , 11213558 , 447077 , 11712649 , 24779724 , 10127622 , 6918454 , 10074640 , 11409972 , 24889392 , 17755052 , 10427712 , 448008 , 153999 , 156414 , 176870 , 15983966]

1320|P33981(32) [208908 , 11213558 , 11485656 , 11364421 , 11667893 , 15983966 , 11234052 , 176167 , 160355 , 10127622 , 11409972 , 447966 , 10074640 , 156414 , 153999 , 6918454 , 11712649 , 11314340 , 3025986 , 10113978 , 17755052 , 151194 , 11338033 , 11656518 , 10427712 , 448008 , 176870 , 16722836 , 24889392 , 3038522 , 24779724 , 11427553 , 447077]

1321|Q00526(6) [153999 , 176167 , 447077 , 176870 , 151194 , 11712649 , 11364421 , 24779724 , 11427553 , 24889392 , 10113978 , 11667893 , 10127622 , 16722836 , 3025986 , 11285002 , 11314340 , 6918454 , 160355 , 11234052 , 11656518 , 11213558 , 15983966 , 156414 , 10427712 , 208908 , 17755052 , 11485656 , 3038522 , 11338033 , 11409972 , 10074640 , 448008]

1322|Q16816(0) [10127622 , 11213558 , 448008 , 11427553 , 16722836 , 176870 , 153999 , 3025986 , 11485656 , 6918454 , 151194 , 11656518 , 11712649 , 208908 , 10113978 , 17755052 , 176167 , 3038522 , 11409972 , 447966 , 11338033 , 24889392 , 24779724 , 11234052 , 10427712 , 447077 , 160355 , 15983966 , 156414 , 11364421 , 10074640 , 11314340 , 11667893]

1323|Q16584(23) [11667893 , 11409972 , 447077 , 16722836 , 156414 , 10074640 , 176870 , 11314340 , 42642645 , 11485656 , 11656518 , 176167 , 6918454 , 11338033 , 11213558 , 151194 , 11427553 , 153999 , 3038522 , 447966 , 17755052 , 10127622 , 24889392 , 208908 , 15983966 , 11712649 , 24779724 , 10113978 , 11234052 , 10427712 , 160355 , 11364421 , 3025986]

1324|P28566(4) [10531 , 119828 , 28693 , 8226 , 197706 , 4440 , 8969 , 115237 , 4585 , 2818 , 3559 , 4106 , 5358 , 60854 , 1355 , 77993 , 5073 , 2159 , 3396 , 5736 , 182137 , 3389 , 5078 , 5002 , 10624 , 1150 , 443884 , 8223 , 60809 , 119570 , 60149 , 1615 , 60857]

1325|P54646(68) [208908 , 10113978 , 448008 , 15983966 , 11485656 , 3025986 , 11213558 , 10074640 , 16722836 , 24779724 , 151194 , 156414 , 11314340 , 11667893 , 11656518 , 17755052 , 11364421 , 153999 , 10427712 , 447077 , 3038522 , 176870 , 160355 , 11338033 , 24889392 , 176167 , 11712649 , 11427553 , 447966 , 11234052 , 11409972 , 10127622 , 6918454]

1326|Q14164(21) [447077 , 11234052 , 10127622 , 11485656 , 11667893 , 11442891 , 208908 , 24779724 , 153999 , 10074640 , 11712649 , 11314340 , 448008 , 3038522 , 11409972 , 11552706 , 176870 , 6918454 , 11656518 , 16722836 , 176167 , 11338033 , 24889392 , 151194 , 3025986 , 17755052 , 10427712 , 10113978 , 11427553 , 15983966 , 156414 , 160355 , 11213558 , 11364421]

1327|O60733(129) [45482339 , 45482338 , 45482342 , 44562726 , 45482340 , 45482337 , 45482343 , 44241583 , 44346482 , 11360688 , 45482332 , 44562673 , 44563072 , 16118459 , 9906670 , 44563042 , 44329617 , 44330207 , 16118461 , 16118318 , 16118320 , 44563074 , 4670 , 44563043 , 16118319 , 25166397 , 44562672 , 45482333 , 45482334 , 44562729 , 44563075 , 45482335 , 45482341 , 44563073]

1328|P50750(39) [11427553 , 17755052 , 3025986 , 6918454 , 10113978 , 10427712 , 11656518 , 151194 , 11285002 , 3038522 , 153999 , 24779724 , 11485656 , 11712649 , 15983966 , 11314340 , 11234052 , 11667893 , 11409972 , 16722836 , 24889392 , 11364421 , 46926350 , 208908 , 447077 , 176167 , 5330286 , 156414 , 10074640 , 160355 , 176870 , 11338033 , 10127622 , 11213558]

1329|P31644(6) [3003157 , 10518 , 107926 , 4506 , 4064 , 4266 , 4912 , 65914 , 2441 , 4999 , 10531 , 10133 , 31304 , 3380 , 37632 , 32051 , 2789 , 3373 , 3033621 , 2893 , 4890 , 2811 , 5556 , 216456 , 10237 , 2118 , 2170 , 3448 , 9908684 , 104781 , 31640 , 3261 , 2576 , 3369]

1330|P29597(62) [11409972 , 11314340 , 176167 , 11712649 , 6918454 , 46866319 , 15983966 , 11213558 , 11427553 , 11656518 , 176870 , 448008 , 3038522 , 151194 , 153999 , 16722836 , 10127622 , 3025986 , 10427712 , 17755052 , 10074640 , 447077 , 160355 , 208908 , 11667893 , 11485656 , 447966 , 11234052 , 156414 , 24889392 , 11364421 , 11338033 , 24779724 , 10113978]

1331|Q13153(80) [16722836 , 11314340 , 11485656 , 3025986 , 11712649 , 153999 , 24779724 , 11234052 , 25227462 , 15983966 , 6918454 , 208908 , 448008 , 176167 , 160355 , 176870 , 151194 , 447966 , 11656518 , 11338033 , 10427712 , 11667893 , 11427553 , 10113978 , 10127622 , 447077 , 10074640 , 3038522 , 11364421 , 11213558 , 156414 , 24889392 , 17755052 , 11409972]

1332|Q9Y468(12) [3156743 , 3242481 , 1973720 , 4396341 , 282398 , 2384580 , 1815815 , 32681 , 719632 , 1066 , 2348 , 2812 , 246835 , 3261980 , 16129778 , 54676538 , 645503 , 3842656 , 3245728 , 1580955 , 893703 , 3236936 , 68089 , 44201975 , 246831 , 4814 , 680935 , 2732927 , 1870615 , 659036 , 18573526 , 820311 , 661085 , 290012]

1333|P68400(29) [15983966 , 16722836 , 17755052 , 156414 , 11364421 , 24889392 , 10113978 , 176167 , 24779724 , 160355 , 10074640 , 447077 , 3025986 , 11409972 , 448008 , 10127622 , 11213558 , 11338033 , 11427553 , 447966 , 11234052 , 10427712 , 208908 , 11656518 , 6918454 , 11712649 , 11485656 , 153999 , 3038522 , 11314340 , 1694 , 151194 , 176870 , 11667893 , 24748573]

1334|P08254(270) [1269845 , 16108938 , 44302165 , 151506 , 44302430 , 10492779 , 4369141 , 44305706 , 128564 , 1066 , 24800541 , 44302432 , 132519 , 10039403 , 10474389 , 119031 , 44270901 , 3342298 , 12473 , 53317936 , 44302167 , 17754156 , 448002 , 9933197 , 44329644 , 24178109 , 15485452 , 94413 , 73761 , 69521 , 44333947 , 10610500 , 1948 , 42601552 , 10073353]

1335|Q07869(168) [2315 , 154000 , 16103190 , 3463 , 11236126 , 204109 , 11395145 , 2750 , 16103189 , 16103197 , 10467 , 9890879 , 16103192 , 170364 , 16734800 , 10274777 , 5694 , 39042 , 2763 , 11149906 , 3339 , 3503 , 206044 , 11711595 , 114924 , 447458 , 5289501 , 15797 , 10229498 , 9909438 , 4829 , 11483970 , 3034285 , 2796 , 6603901]

1336|O94806(3) [10427712 , 11409972 , 11667893 , 11234052 , 160355 , 153999 , 76098 , 10113978 , 24779724 , 11364421 , 15983966 , 10127622 , 156414 , 16722836 , 208908 , 24889392 , 16122633 , 11213558 , 17755052 , 11314340 , 11608401 , 11485656 , 11338033 , 151194 , 11427553 , 447077 , 3025986 , 11712649 , 11656518 , 176167 , 6918454 , 176870 , 10074640 , 448008 , 3038522]

1337|P08172(17) [3696 , 2381 , 2229 , 441071 , 2160 , 71183 , 4848 , 11519070 , 187 , 11434515 , 10938 , 442021 , 15376 , 71203 , 2230 , 30843 , 107867 , 4629 , 3450 , 444031 , 3494 , 6172 , 2784 , 174174 , 4634 , 60809 , 154059 , 4934 , 443879 , 2370 , 44259 , 3042 , 24199 , 5910 , 2551]

1338|P35462(32) [57267 , 54746 , 4917 , 6918314 , 219050 , 5095 , 37459 , 5355 , 4926 , 5073 , 688272 , 6005 , 54562 , 31101 , 47811 , 4850 , 2726 , 60149 , 28864 , 11697676 , 3559 , 16362 , 443951 , 119570 , 5736 , 3964 , 3033769 , 5281881 , 5265 , 681 , 59227 , 2818 , 115368 , 3151 , 57242 , 4452]

1339|P27338(30) [21910 , 3052776 , 71164 , 71627 , 4380 , 68555 , 5576 , 4688 , 26757 , 5530 , 3435 , 11967800 , 2134 , 71307 , 15433 , 5494443 , 4235 , 5289613 , 3759 , 4064 , 3748 , 21558 , 3675 , 68804 , 46937129 , 29142 , 2750 , 4362 , 11616886 , 12717 , 23659732 , 131682 , 233979 , 16750123 , 16739244 , 10192617]

1340|P28222(30) [5736 , 5073 , 2818 , 219050 , 443951 , 4106 , 9966051 , 4440 , 5358 , 77993 , 60149 , 5074 , 77992 , 54746 , 8969 , 3388 , 28693 , 71351 , 197706 , 123606 , 60795 , 4830 , 9805719 , 10531 , 60854 , 60809 , 66004 , 1150 , 47811 , 5078 , 28864 , 4636 , 60857 , 3822 , 4585 , 31101]

1341|P29474(359) [10221335 , 24894151 , 1433 , 10103242 , 1649 , 39836 , 19049087 , 15552206 , 22617750 , 44516953 , 1894 , 12778906 , 181426 , 18721726 , 9793827 , 132862 , 44396192 , 47938 , 44313550 , 449633 , 19049152 , 9797857 , 18721724 , 18721725 , 10198539 , 19049121 , 347590 , 11149707 , 10261068 , 44313313 , 107984 , 123895 , 15689 , 18721729 , 44322997 , 10104379]

1342|Q9NYY3(28) [15983966 , 9549303 , 6918454 , 153999 , 11427553 , 10113978 , 11409972 , 11485656 , 3025986 , 447077 , 10427712 , 2856 , 11712649 , 11213558 , 3973 , 3038522 , 151194 , 10127622 , 11656518 , 2396 , 6419766 , 24779724 , 11667893 , 156414 , 176870 , 24889392 , 447966 , 11364421 , 11234052 , 11314340 , 208908 , 11338033 , 5005498 , 16722836 , 176167 , 17755052 , 10074640]

1343|P43166(3) [20184392 , 5284549 , 19772348 , 16122590 , 10112 , 3295 , 44424363 , 12066941 , 11117301 , 25258361 , 6852128 , 216468 , 11967800 , 96876 , 5734 , 12066940 , 36811 , 5356 , 15729847 , 68844 , 76509 , 2732 , 25258363 , 5355 , 6307 , 1986 , 5287541 , 5284627 , 69809 , 169682 , 389641 , 4100 , 25258360 , 72139 , 44424364 , 14611919 , 44424366]

1344|O14733(130) [3973 , 11338033 , 3038522 , 447966 , 11656518 , 11234052 , 11364421 , 17755052 , 10127622 , 151194 , 16722836 , 11213558 , 156414 , 6419766 , 9549303 , 10113978 , 176870 , 447077 , 153999 , 11314340 , 15983966 , 24889392 , 11485656 , 3025986 , 11667893 , 5005498 , 2396 , 208908 , 6918454 , 11712649 , 176167 , 10074640 , 24779724 , 2856 , 11427553 , 10427712 , 11409972]

1345|P11229(12) [5749 , 187 , 3494 , 44259 , 4634 , 2784 , 3290 , 2370 , 11434515 , 2230 , 71183 , 442021 , 154059 , 5910 , 2911 , 9577995 , 15376 , 10938 , 60809 , 4848 , 11519070 , 3042 , 2818 , 107867 , 2551 , 4629 , 9862248 , 2229 , 5572 , 444031 , 174174 , 2160 , 2381 , 9571002 , 71203 , 441071 , 4934]

1346|Q86Y07(4) [11213558 , 11485656 , 10427712 , 208908 , 153999 , 24779724 , 11409972 , 24889392 , 3025986 , 11234052 , 10113978 , 11314340 , 11338033 , 11667893 , 11656518 , 176870 , 11427553 , 11364421 , 6419766 , 17755052 , 15983966 , 16722836 , 447966 , 2396 , 3038522 , 5005498 , 176167 , 156414 , 151194 , 10074640 , 3973 , 11712649 , 2856 , 9549303 , 10127622 , 6918454 , 447077]

1347|Q9Y253(26) [3676681 , 1973720 , 2435 , 11852 , 265341 , 4593 , 54676538 , 10621 , 722121 , 2768975 , 5289501 , 10168 , 1967 , 2016 , 265580 , 5282176 , 54675783 , 3746037 , 3698 , 1870753 , 21138 , 316274 , 2090 , 5198 , 2179 , 701332 , 1369 , 3151041 , 70464 , 8209243 , 3885 , 1870615 , 327044 , 265436 , 5405 , 299996 , 31475]

1348|P51617(57) [447077 , 3038522 , 11338033 , 3025986 , 10074640 , 11314340 , 24779724 , 11364421 , 11409972 , 2856 , 151194 , 2396 , 10113978 , 5005498 , 16722836 , 24889392 , 11667893 , 11213558 , 176167 , 11234052 , 10127622 , 156414 , 15983966 , 9549303 , 17755052 , 208908 , 153999 , 11656518 , 10427712 , 6918454 , 6419766 , 11427553 , 3973 , 11485656 , 447966 , 176870 , 11712649]

1349|Q7L7X3(3) [11656518 , 3038522 , 153999 , 24889392 , 11364421 , 447077 , 151194 , 10074640 , 24779724 , 11485656 , 11409972 , 10113978 , 17755052 , 176167 , 6419766 , 208908 , 9549303 , 3973 , 6918454 , 11712649 , 11213558 , 447966 , 11427553 , 176870 , 11234052 , 3025986 , 16722836 , 10427712 , 2856 , 11314340 , 11667893 , 10127622 , 15983966 , 156414 , 2396 , 5005498 , 11338033]

1350|Q6PHR2(5) [176167 , 15983966 , 11427553 , 6918454 , 10427712 , 11213558 , 156414 , 24779724 , 16722836 , 11656518 , 24889392 , 11667893 , 10127622 , 2396 , 153999 , 5005498 , 447077 , 151194 , 11314340 , 11338033 , 11712649 , 10113978 , 11364421 , 447966 , 11485656 , 17755052 , 2856 , 11234052 , 10074640 , 11409972 , 3025986 , 176870 , 208908 , 6419766 , 3973 , 3038522 , 9549303]

1351|Q8IYT8(2) [11409972 , 11712649 , 153999 , 2856 , 6419766 , 11314340 , 11427553 , 447077 , 10427712 , 11364421 , 208908 , 11667893 , 176167 , 176870 , 11234052 , 151194 , 17755052 , 11485656 , 9549303 , 11213558 , 10127622 , 3973 , 24889392 , 2396 , 156414 , 15983966 , 16722836 , 6918454 , 11338033 , 3025986 , 447966 , 5005498 , 11656518 , 24779724 , 3038522 , 10113978 , 10074640]

1352|Q9H2K8(3) [6918454 , 11234052 , 6419766 , 2396 , 16722836 , 11667893 , 447966 , 10127622 , 10074640 , 11338033 , 15983966 , 3038522 , 153999 , 11364421 , 10427712 , 208908 , 11656518 , 11427553 , 24779724 , 10113978 , 17755052 , 9549303 , 176870 , 176167 , 11712649 , 24889392 , 3973 , 5005498 , 11314340 , 3025986 , 447077 , 11485656 , 11409972 , 156414 , 2856 , 11213558 , 151194]

1353|Q9UL54(8) [2856 , 11364421 , 11338033 , 11427553 , 11667893 , 11234052 , 208908 , 3973 , 11314340 , 5005498 , 10427712 , 9549303 , 17755052 , 151194 , 11656518 , 176870 , 6918454 , 24779724 , 3025986 , 15983966 , 11213558 , 156414 , 3038522 , 16722836 , 2396 , 24889392 , 176167 , 11712649 , 10113978 , 153999 , 10074640 , 10127622 , 447077 , 11409972 , 447966 , 11485656 , 6419766]

1354|Q8TD08(7) [156414 , 15983966 , 24779724 , 10172943 , 176870 , 11427553 , 11712649 , 3038522 , 153999 , 3078519 , 24889392 , 3542 , 11409972 , 176167 , 151194 , 447077 , 11667893 , 6918454 , 10113978 , 17755052 , 11213558 , 10074640 , 11656518 , 16722836 , 11234052 , 3973 , 11314340 , 11485656 , 3025986 , 208908 , 5005498 , 11338033 , 10427712 , 10127622 , 160355 , 3540 , 11364421]

1355|Q9Y6E0(8) [2396 , 11427553 , 9549303 , 5005498 , 151194 , 10427712 , 3973 , 24779724 , 11338033 , 3025986 , 447077 , 2856 , 447966 , 11234052 , 11656518 , 10127622 , 6419766 , 208908 , 11364421 , 15983966 , 11314340 , 11485656 , 11213558 , 176167 , 10113978 , 160355 , 3038522 , 11667893 , 11712649 , 11409972 , 176870 , 17755052 , 16722836 , 153999 , 6918454 , 156414 , 24889392 , 10074640]

1356|O43318(51) [11656518 , 15983966 , 11427553 , 11314340 , 447077 , 24779724 , 11234052 , 10113978 , 11338033 , 2396 , 10127622 , 11409972 , 11364421 , 176870 , 17755052 , 9549303 , 11485656 , 11285002 , 2856 , 153999 , 3973 , 10074640 , 3038522 , 176167 , 447966 , 5005498 , 11667893 , 156414 , 3025986 , 151194 , 11213558 , 11712649 , 10427712 , 208908 , 6419766 , 24889392 , 6918454 , 16722836]

1357|Q8WTQ7(2) [6918454 , 208908 , 2396 , 5005498 , 11667893 , 3025986 , 11485656 , 24889392 , 15983966 , 11314340 , 448008 , 447077 , 176870 , 156414 , 11364421 , 10113978 , 9549303 , 6419766 , 11213558 , 16722836 , 151194 , 17755052 , 11234052 , 11656518 , 447966 , 11712649 , 10127622 , 3038522 , 11338033 , 3973 , 11409972 , 10427712 , 11427553 , 2856 , 24779724 , 153999 , 10074640 , 176167]

1358|Q9UBE8(21) [2856 , 11656518 , 6918454 , 160355 , 11213558 , 11427553 , 11338033 , 11314340 , 151194 , 17755052 , 24779724 , 9549303 , 2396 , 11485656 , 176167 , 5005498 , 156414 , 10074640 , 15983966 , 176870 , 3973 , 11667893 , 10127622 , 10427712 , 11364421 , 11712649 , 153999 , 3025986 , 447077 , 16722836 , 208908 , 24889392 , 3038522 , 11409972 , 447966 , 11234052 , 6419766 , 10113978]

1359|P52564(17) [10427712 , 6918454 , 11213558 , 151194 , 24889392 , 11234052 , 5005498 , 176870 , 10127622 , 3025986 , 11485656 , 447966 , 447077 , 3973 , 176167 , 11338033 , 10113978 , 11314340 , 11667893 , 11364421 , 17755052 , 2856 , 160355 , 10074640 , 11712649 , 6419766 , 11427553 , 16722836 , 11656518 , 24779724 , 208908 , 11409972 , 15983966 , 153999 , 156414 , 3038522 , 2396 , 9549303]

1360|Q9BUB5(11) [10127622 , 3025986 , 17755052 , 3973 , 24889392 , 3542 , 5005498 , 16722836 , 11364421 , 11213558 , 448008 , 24779724 , 447077 , 3078519 , 15983966 , 160355 , 11409972 , 11314340 , 11338033 , 3038522 , 3540 , 10427712 , 11485656 , 176167 , 10172943 , 11427553 , 11656518 , 6918454 , 151194 , 208908 , 10113978 , 153999 , 11667893 , 156414 , 10074640 , 176870 , 11234052 , 11712649]

1361|Q8NG66(4) [15983966 , 6419766 , 11409972 , 447077 , 11314340 , 2856 , 10074640 , 9549303 , 153999 , 10427712 , 10113978 , 16722836 , 24889392 , 447966 , 11485656 , 11364421 , 11338033 , 10127622 , 176870 , 3038522 , 44462760 , 2396 , 156414 , 11427553 , 24779724 , 208908 , 11213558 , 151194 , 11656518 , 11667893 , 11712649 , 5005498 , 17755052 , 6918454 , 3973 , 176167 , 11234052 , 3025986]

1362|Q15831(117) [11364421 , 10127622 , 208908 , 24889392 , 11712649 , 447966 , 151194 , 11427553 , 3973 , 176167 , 11314340 , 176870 , 16722836 , 156414 , 153999 , 10074640 , 160355 , 3025986 , 11338033 , 15983966 , 9549303 , 2856 , 3038522 , 10113978 , 5005498 , 6419766 , 11667893 , 11656518 , 2396 , 6918454 , 11234052 , 17755052 , 11485656 , 24779724 , 11213558 , 10427712 , 11409972 , 447077]

1363|Q96RR4(14) [208908 , 10427712 , 24889392 , 11485656 , 3078519 , 24779724 , 447077 , 16722836 , 3038522 , 10172943 , 10127622 , 3973 , 3542 , 15983966 , 11213558 , 5005498 , 6918454 , 176167 , 447966 , 176870 , 11338033 , 3025986 , 3540 , 11427553 , 17755052 , 11409972 , 11314340 , 160355 , 153999 , 10113978 , 11234052 , 11656518 , 11667893 , 11364421 , 151194 , 11712649 , 156414 , 10074640]

1364|P07477(38) [1507 , 10820951 , 9547945 , 4413 , 9547939 , 25134248 , 24963037 , 177837 , 17754078 , 9547940 , 25113614 , 46937030 , 24963036 , 11840929 , 446605 , 1800 , 23629654 , 44141860 , 446978 , 199994 , 448748 , 10324367 , 1746 , 445756 , 4469371 , 2536 , 444753 , 44331389 , 445843 , 2014 , 444581 , 446604 , 6540268 , 1001 , 448062 , 24963035 , 46228924 , 204102]

1365|Q9UPE1(1) [153999 , 5005498 , 3038522 , 176167 , 11712649 , 3973 , 447077 , 2396 , 6419766 , 176870 , 11338033 , 11485656 , 2856 , 11409972 , 447966 , 10074640 , 17755052 , 11656518 , 6918454 , 448008 , 10427712 , 156414 , 10127622 , 11427553 , 15983966 , 3025986 , 11213558 , 24779724 , 11234052 , 9549303 , 11314340 , 24889392 , 11667893 , 16722836 , 10113978 , 151194 , 11364421 , 208908]

1366|P15056(354) [24889392 , 176167 , 10127622 , 153999 , 17755052 , 11364421 , 11656518 , 447966 , 160355 , 216239 , 11409972 , 10113978 , 42611257 , 24779724 , 11213558 , 11712649 , 16722836 , 151194 , 24794418 , 208908 , 44462760 , 11314340 , 176870 , 10427712 , 11427553 , 448008 , 3025986 , 11167602 , 11338033 , 15983966 , 447077 , 6918454 , 11667893 , 156414 , 11234052 , 11485656 , 3038522 , 10074640]

1367|P21709(38) [10127622 , 16722836 , 5005498 , 11213558 , 10113978 , 3038522 , 3973 , 11234052 , 11427553 , 11485656 , 24889392 , 208908 , 11712649 , 176870 , 6918454 , 11314340 , 447966 , 11667893 , 6419766 , 10427712 , 176167 , 153999 , 151194 , 2856 , 2396 , 10074640 , 17755052 , 11338033 , 11409972 , 11656518 , 447077 , 11364421 , 156414 , 15983966 , 9549303 , 24779724 , 3025986 , 160355]

1368|Q8N4C8(5) [11667893 , 208908 , 6419766 , 16722836 , 3038522 , 5005498 , 176167 , 9549303 , 11409972 , 3973 , 11712649 , 11338033 , 2856 , 24779724 , 24889392 , 10113978 , 153999 , 10427712 , 151194 , 11485656 , 6918454 , 11656518 , 10127622 , 11234052 , 176870 , 11314340 , 15983966 , 156414 , 448008 , 447966 , 3025986 , 10074640 , 11213558 , 11364421 , 11427553 , 17755052 , 2396 , 447077]

1369|Q09013(45) [11234052 , 153999 , 17755052 , 11338033 , 10127622 , 11409972 , 3973 , 6918454 , 11314340 , 176870 , 160355 , 16722836 , 208908 , 11712649 , 447966 , 11667893 , 176167 , 10427712 , 11485656 , 10074640 , 3025986 , 10113978 , 6419766 , 11364421 , 11213558 , 9549303 , 151194 , 2856 , 447077 , 11656518 , 24779724 , 156414 , 5005498 , 11427553 , 3038522 , 15983966 , 24889392 , 2396]

1370|P54762(32) [11712649 , 11485656 , 3025986 , 11213558 , 15983966 , 2856 , 24889392 , 17755052 , 11656518 , 10074640 , 11364421 , 11409972 , 6918454 , 176167 , 3038522 , 16722836 , 11234052 , 10427712 , 153999 , 6419766 , 11338033 , 11427553 , 447966 , 10127622 , 160355 , 10113978 , 11667893 , 2396 , 151194 , 156414 , 11314340 , 24779724 , 447077 , 208908 , 9549303 , 5005498 , 3973 , 176870]

1371|Q8N5S9(2) [10127622 , 160355 , 447077 , 11314340 , 11485656 , 208908 , 24889392 , 11234052 , 6918454 , 11656518 , 10113978 , 15983966 , 11667893 , 156414 , 11427553 , 151194 , 3973 , 11213558 , 17755052 , 3038522 , 5005498 , 176167 , 24779724 , 11338033 , 10427712 , 16722836 , 11409972 , 11364421 , 3542 , 3025986 , 447966 , 176870 , 10172943 , 153999 , 3078519 , 11712649 , 3540 , 10074640]

1372|Q9UIK4(25) [11314340 , 176167 , 6419766 , 2396 , 2856 , 3025986 , 447966 , 11667893 , 11485656 , 153999 , 11234052 , 11338033 , 208908 , 156414 , 10427712 , 11213558 , 16722836 , 11712649 , 10127622 , 5005498 , 17755052 , 3038522 , 24889392 , 11427553 , 6918454 , 9549303 , 176870 , 11409972 , 160355 , 151194 , 447077 , 24779724 , 11656518 , 3973 , 10074640 , 15983966 , 10113978 , 11364421]

1373|P41743(25) [11485656 , 447966 , 24889392 , 11338033 , 24779724 , 10127622 , 11234052 , 447077 , 11364421 , 15983966 , 10074640 , 176167 , 2856 , 448008 , 208908 , 5005498 , 11213558 , 2396 , 10427712 , 176870 , 11427553 , 10113978 , 6918454 , 6419766 , 11656518 , 153999 , 9549303 , 11314340 , 3038522 , 11712649 , 3973 , 16722836 , 17755052 , 151194 , 11409972 , 11667893 , 3025986 , 156414]

1374|P36896(14) [11213558 , 176167 , 5005498 , 156414 , 17755052 , 2856 , 11409972 , 16722836 , 6918454 , 176870 , 3038522 , 11656518 , 2396 , 3973 , 15983966 , 151194 , 11314340 , 24779724 , 10427712 , 10074640 , 208908 , 153999 , 11712649 , 11485656 , 447077 , 9549303 , 11234052 , 11427553 , 160355 , 24889392 , 447966 , 11364421 , 10113978 , 6419766 , 3025986 , 11667893 , 11338033 , 10127622]

1375|P78362(9) [6419766 , 176870 , 156414 , 16722836 , 3038522 , 11213558 , 447966 , 11485656 , 2856 , 176167 , 11409972 , 11314340 , 447077 , 11656518 , 153999 , 11338033 , 10427712 , 11667893 , 11427553 , 9549303 , 3025986 , 24779724 , 11364421 , 2396 , 208908 , 10074640 , 11234052 , 3973 , 5005498 , 160355 , 17755052 , 24889392 , 151194 , 10113978 , 11712649 , 6918454 , 15983966 , 10127622]

1376|P29322(55) [160355 , 176167 , 2396 , 447966 , 11364421 , 10113978 , 11667893 , 208908 , 153999 , 11656518 , 3038522 , 6918454 , 11712649 , 10427712 , 151194 , 10074640 , 16722836 , 156414 , 11234052 , 17755052 , 176870 , 5005498 , 9549303 , 11213558 , 24889392 , 15983966 , 10127622 , 2856 , 11314340 , 3973 , 11427553 , 6419766 , 24779724 , 11338033 , 447077 , 3025986 , 11409972 , 11485656]

1377|Q86Z02(2) [2856 , 16722836 , 11314340 , 447966 , 448008 , 11234052 , 10427712 , 3973 , 447077 , 151194 , 10074640 , 9549303 , 11485656 , 11213558 , 17755052 , 2396 , 11427553 , 6918454 , 176870 , 15983966 , 11712649 , 153999 , 156414 , 3038522 , 208908 , 11667893 , 24889392 , 10127622 , 176167 , 5005498 , 10113978 , 24779724 , 11338033 , 6419766 , 3025986 , 11364421 , 11409972 , 11656518]

1378|Q9UEE5(4) [447077 , 16722836 , 11364421 , 160355 , 11409972 , 11667893 , 3025986 , 11234052 , 2856 , 11314340 , 10427712 , 6419766 , 11712649 , 176167 , 5005498 , 208908 , 10113978 , 24779724 , 156414 , 24889392 , 15983966 , 153999 , 151194 , 17755052 , 447966 , 3038522 , 3973 , 11427553 , 11213558 , 11338033 , 11656518 , 6918454 , 2396 , 9549303 , 10074640 , 11485656 , 176870 , 10127622]

1379|Q9H4B4(19) [10427712 , 11656518 , 176870 , 15983966 , 153999 , 6419766 , 2396 , 176167 , 447077 , 3038522 , 11338033 , 11427553 , 3973 , 447966 , 24779724 , 11712649 , 24889392 , 10074640 , 17755052 , 11667893 , 11234052 , 11364421 , 208908 , 10113978 , 160355 , 156414 , 5005498 , 11213558 , 11485656 , 6918454 , 2856 , 3025986 , 9549303 , 10127622 , 16722836 , 11314340 , 11409972 , 151194]

1380|Q99683(58) [176870 , 447077 , 6419766 , 10074640 , 3038522 , 11338033 , 10427712 , 10113978 , 11667893 , 151194 , 11364421 , 176167 , 11314340 , 24779724 , 10127622 , 156414 , 11213558 , 17755052 , 9549303 , 16722836 , 2396 , 208908 , 3025986 , 11485656 , 15983966 , 3973 , 447966 , 5005498 , 11656518 , 6918454 , 153999 , 11712649 , 24889392 , 11234052 , 2856 , 11427553 , 160355 , 11409972]

1381|Q86UE8(7) [6419766 , 160355 , 153999 , 447077 , 2396 , 15983966 , 10427712 , 447966 , 3025986 , 208908 , 11485656 , 11409972 , 151194 , 11314340 , 176870 , 11364421 , 2856 , 24779724 , 16722836 , 11338033 , 11213558 , 156414 , 11656518 , 9549303 , 11667893 , 10113978 , 6918454 , 176167 , 3038522 , 3973 , 11234052 , 11427553 , 5005498 , 24889392 , 10127622 , 17755052 , 10074640 , 11712649]

1382|O43353(30) [2856 , 447077 , 176870 , 11427553 , 2396 , 3973 , 11409972 , 24889392 , 153999 , 5005498 , 176167 , 11667893 , 3038522 , 6918454 , 10113978 , 151194 , 6419766 , 11314340 , 9549303 , 11234052 , 10127622 , 15983966 , 10074640 , 16722836 , 11712649 , 11338033 , 24779724 , 156414 , 10427712 , 160355 , 11485656 , 208908 , 3025986 , 17755052 , 447966 , 11364421 , 11213558 , 11656518]

1383|Q9BYT3(2) [176167 , 6419766 , 2856 , 11427553 , 6918454 , 2396 , 11656518 , 11409972 , 10074640 , 160355 , 10113978 , 3038522 , 16722836 , 156414 , 10427712 , 153999 , 24779724 , 11314340 , 151194 , 9549303 , 24889392 , 208908 , 11712649 , 5005498 , 447966 , 3025986 , 176870 , 15983966 , 11667893 , 11338033 , 447077 , 11364421 , 10127622 , 11485656 , 3973 , 17755052 , 11213558 , 11234052]

1384|Q9Y6M4(3) [160355 , 6419766 , 16722836 , 11656518 , 151194 , 11364421 , 447077 , 11409972 , 3025986 , 11338033 , 11234052 , 447966 , 11712649 , 11314340 , 5005498 , 3973 , 3038522 , 24889392 , 10074640 , 2856 , 10113978 , 6918454 , 11427553 , 10127622 , 2396 , 11667893 , 11485656 , 9549303 , 156414 , 153999 , 208908 , 24779724 , 176167 , 176870 , 17755052 , 10427712 , 11213558 , 448008 , 15983966]

1385|Q9Y5S2(3) [6419766 , 10127622 , 153999 , 3038522 , 208908 , 11409972 , 10113978 , 24889392 , 9549303 , 10427712 , 11234052 , 447966 , 11213558 , 11712649 , 16722836 , 11364421 , 447077 , 11338033 , 15983966 , 2396 , 11656518 , 5005498 , 3973 , 11667893 , 160355 , 2856 , 10074640 , 176870 , 17755052 , 448008 , 3025986 , 156414 , 11485656 , 11427553 , 151194 , 24779724 , 6918454 , 11314340 , 176167]

1386|P54753(8) [447077 , 24889392 , 447966 , 11314340 , 3973 , 176167 , 10427712 , 11712649 , 11485656 , 10113978 , 6918454 , 16722836 , 11213558 , 3038522 , 6419766 , 10127622 , 176870 , 160355 , 11427553 , 208908 , 11667893 , 156414 , 448008 , 2396 , 24779724 , 5005498 , 17755052 , 11234052 , 2856 , 11338033 , 10074640 , 3025986 , 11656518 , 9549303 , 151194 , 15983966 , 11364421 , 153999 , 11409972]

1387|Q13043(13) [447966 , 208908 , 11485656 , 3025986 , 11213558 , 24779724 , 10113978 , 11409972 , 11314340 , 11364421 , 16722836 , 5005498 , 160355 , 156414 , 17755052 , 6419766 , 11712649 , 2396 , 11667893 , 176870 , 6918454 , 2856 , 10074640 , 176167 , 3973 , 15983966 , 11427553 , 11338033 , 151194 , 447077 , 10427712 , 24889392 , 153999 , 11234052 , 448008 , 9549303 , 10127622 , 3038522 , 11656518]

1388|P34903(25) [3033621 , 2576 , 10133 , 65914 , 2789 , 2118 , 10531 , 10518 , 5732 , 4064 , 5556 , 23581869 , 4999 , 32051 , 3016 , 104781 , 4912 , 4266 , 107926 , 4506 , 2802 , 3369 , 31640 , 2811 , 2441 , 10237 , 2170 , 2893 , 216456 , 37632 , 31304 , 131664 , 3261 , 9908684 , 3003157 , 3380 , 4890 , 3373 , 3448]

1389|O75914(25) [10074640 , 156414 , 11364421 , 160355 , 6419766 , 17755052 , 15983966 , 11667893 , 151194 , 11485656 , 9549303 , 3038522 , 176870 , 6918454 , 11656518 , 208908 , 5005498 , 153999 , 11427553 , 11314340 , 11234052 , 16722836 , 11409972 , 11712649 , 447966 , 447077 , 2856 , 11338033 , 10427712 , 10127622 , 448008 , 10113978 , 24779724 , 3025986 , 3973 , 11213558 , 2396 , 176167 , 24889392]

1390|Q9UHD2(55) [11234052 , 2396 , 9549303 , 3973 , 11485656 , 156414 , 448008 , 10427712 , 3038522 , 24779724 , 447077 , 11427553 , 5005498 , 11364421 , 11213558 , 10127622 , 11656518 , 11314340 , 10113978 , 2856 , 151194 , 208908 , 17755052 , 447966 , 24748573 , 11409972 , 176167 , 6419766 , 16722836 , 11338033 , 176870 , 11712649 , 15983966 , 11667893 , 153999 , 3025986 , 10074640 , 24889392 , 6918454]

1391|Q13882(21) [17755052 , 11409972 , 6419766 , 3973 , 3038522 , 10127622 , 11656518 , 447077 , 11712649 , 151194 , 11364421 , 15983966 , 2396 , 11314340 , 9549303 , 11213558 , 24889392 , 156414 , 11485656 , 176870 , 3025986 , 10113978 , 160355 , 208908 , 11667893 , 153999 , 6918454 , 5005498 , 16722836 , 176167 , 11338033 , 448008 , 10074640 , 11427553 , 447966 , 24779724 , 2856 , 11234052 , 10427712]

1392|O43293(26) [11667893 , 17755052 , 160355 , 11234052 , 208908 , 11213558 , 6918454 , 2856 , 16722836 , 9549303 , 10127622 , 11656518 , 11409972 , 151194 , 176167 , 10427712 , 11485656 , 3025986 , 156414 , 176870 , 11314340 , 2396 , 3038522 , 5005498 , 11427553 , 3973 , 24779724 , 6419766 , 10074640 , 153999 , 24748573 , 15983966 , 447077 , 11338033 , 11364421 , 10113978 , 447966 , 11712649 , 24889392]

1393|A0A0B4J2F2(7) [11712649 , 11485656 , 3038522 , 447966 , 448008 , 24889392 , 11314340 , 153999 , 447077 , 11656518 , 176167 , 15983966 , 16722836 , 2856 , 156414 , 17755052 , 3973 , 151194 , 208908 , 10113978 , 176870 , 24779724 , 11213558 , 9549303 , 11409972 , 11364421 , 10127622 , 3025986 , 10427712 , 11427553 , 2396 , 160355 , 11667893 , 11338033 , 10074640 , 6918454 , 5005498 , 11234052 , 6419766]

1394|P42681(1) [11409972 , 156414 , 15983966 , 11485656 , 16722836 , 176167 , 11656518 , 11427553 , 176870 , 10427712 , 208908 , 11364421 , 3025986 , 10127622 , 3973 , 10113978 , 448008 , 447966 , 11667893 , 153999 , 151194 , 6419766 , 3038522 , 2396 , 24779724 , 160355 , 10074640 , 6918454 , 447077 , 24889392 , 11314340 , 11338033 , 5005498 , 9549303 , 2856 , 11213558 , 11234052 , 17755052 , 11712649]

1395|P15735(11) [9549303 , 3025986 , 11667893 , 17755052 , 447077 , 156414 , 176870 , 208908 , 3038522 , 10074640 , 2856 , 10427712 , 2396 , 24779724 , 11656518 , 11314340 , 176167 , 6419766 , 16722836 , 11338033 , 11409972 , 15983966 , 11234052 , 153999 , 6918454 , 447966 , 11712649 , 448008 , 11427553 , 10113978 , 11485656 , 11213558 , 160355 , 3973 , 24889392 , 151194 , 5005498 , 10127622 , 11364421]

1396|Q9UK32(10) [24779724 , 160355 , 448008 , 3973 , 10113978 , 176167 , 3038522 , 5005498 , 208908 , 15983966 , 11314340 , 2396 , 10127622 , 11485656 , 10074640 , 3025986 , 11364421 , 11656518 , 151194 , 153999 , 11234052 , 156414 , 11427553 , 9549303 , 16722836 , 176870 , 11409972 , 24889392 , 11338033 , 447077 , 2856 , 6918454 , 11213558 , 10427712 , 11712649 , 6419766 , 17755052 , 447966 , 11667893]

1397|Q08881(91) [208908 , 11364421 , 3025986 , 15983966 , 447077 , 10074640 , 11409972 , 11338033 , 10427712 , 11314340 , 5005498 , 176167 , 9549303 , 10127622 , 11712649 , 17755052 , 11485656 , 11234052 , 11667893 , 24889392 , 447966 , 160355 , 6918454 , 10113978 , 11656518 , 6419766 , 3038522 , 11213558 , 2856 , 3973 , 156414 , 16722836 , 2396 , 448008 , 24779724 , 151194 , 11427553 , 153999 , 176870]

1398|P47869(8) [2802 , 4890 , 2170 , 4266 , 2811 , 3369 , 104781 , 107926 , 10133 , 32051 , 3380 , 10518 , 4912 , 2893 , 9908684 , 31304 , 3033621 , 4506 , 4999 , 5732 , 4064 , 23581869 , 3003157 , 5556 , 10237 , 131664 , 2789 , 3261 , 2441 , 65914 , 2576 , 31640 , 3448 , 216456 , 3016 , 2118 , 3373 , 37632 , 10531]

1399|U3KQB3(0) [10427712 , 3973 , 5005498 , 11314340 , 10074640 , 151194 , 24779724 , 176167 , 9549303 , 3025986 , 11364421 , 2396 , 11409972 , 11656518 , 156414 , 6918454 , 10127622 , 176870 , 208908 , 11485656 , 24889392 , 2856 , 11213558 , 11667893 , 447077 , 11427553 , 447966 , 16722836 , 6419766 , 160355 , 11338033 , 15983966 , 11712649 , 153999 , 3038522 , 10113978 , 17755052 , 448008 , 11234052]

1400|Q00535(65) [11285002 , 176167 , 11364421 , 11656518 , 15983966 , 11485656 , 11409972 , 153999 , 448991 , 16722836 , 46926350 , 10127622 , 11314340 , 447966 , 5005498 , 11712649 , 156414 , 10427712 , 3038522 , 11667893 , 11338033 , 10113978 , 6918454 , 3025986 , 447077 , 208908 , 10074640 , 11427553 , 24779724 , 11213558 , 151194 , 176870 , 17754027 , 160355 , 448008 , 11234052 , 17755052 , 24889392 , 4592]

1401|P54764(39) [16722836 , 10427712 , 10127622 , 11338033 , 447966 , 10113978 , 10074640 , 11314340 , 11667893 , 11427553 , 6419766 , 160355 , 151194 , 2396 , 6918454 , 5005498 , 447077 , 9549303 , 3973 , 176870 , 11485656 , 3025986 , 11712649 , 11213558 , 11364421 , 2856 , 448008 , 24779724 , 208908 , 11409972 , 11656518 , 24889392 , 17755052 , 15983966 , 3038522 , 156414 , 176167 , 153999 , 11234052]

1402|Q5VT25(5) [11234052 , 11667893 , 17755052 , 10127622 , 2856 , 448008 , 10427712 , 153999 , 156414 , 24889392 , 160355 , 9549303 , 11427553 , 3038522 , 5005498 , 11338033 , 10113978 , 15983966 , 2396 , 6419766 , 11656518 , 10074640 , 11485656 , 24779724 , 447966 , 11213558 , 208908 , 16722836 , 6918454 , 11409972 , 176167 , 176870 , 3973 , 11364421 , 3025986 , 447077 , 151194 , 11314340 , 11712649]

1403|O94804(2) [11213558 , 11667893 , 17755052 , 2856 , 10113978 , 11314340 , 3025986 , 11427553 , 11338033 , 151194 , 5005498 , 11656518 , 176167 , 6918454 , 3973 , 176870 , 160355 , 10074640 , 3038522 , 11712649 , 10427712 , 2396 , 11234052 , 11485656 , 153999 , 11364421 , 448008 , 15983966 , 10127622 , 208908 , 447077 , 156414 , 6419766 , 16722836 , 24779724 , 447966 , 24889392 , 11409972 , 9549303]

1404|O15111(65) [24779724 , 2396 , 11712649 , 156414 , 6419766 , 15983966 , 10127622 , 6918454 , 447966 , 208908 , 127864 , 448008 , 11667893 , 10427712 , 447077 , 11338033 , 5005498 , 3025986 , 11485656 , 3973 , 11234052 , 16722836 , 11314340 , 10113978 , 10074640 , 151194 , 11656518 , 11213558 , 17755052 , 153999 , 9549303 , 176167 , 11364421 , 3038522 , 176870 , 11409972 , 2856 , 11427553 , 24889392]

1405|Q8IU85(6) [11712649 , 176167 , 447077 , 156414 , 11213558 , 17755052 , 11409972 , 16722836 , 6918454 , 9549303 , 10113978 , 24889392 , 176870 , 5005498 , 153999 , 151194 , 10427712 , 11234052 , 11485656 , 448008 , 447966 , 11314340 , 3973 , 15983966 , 11667893 , 11427553 , 11656518 , 11364421 , 2856 , 11338033 , 24779724 , 2396 , 3025986 , 160355 , 208908 , 6419766 , 10074640 , 10127622 , 3038522]

1406|Q04912(61) [447077 , 3038522 , 448008 , 3973 , 15983966 , 176870 , 5005498 , 156414 , 24889392 , 176167 , 10427712 , 10127622 , 2396 , 9549303 , 151194 , 45142457 , 11314340 , 3025986 , 11485656 , 11656518 , 10113978 , 6918454 , 208908 , 11667893 , 11213558 , 2856 , 16722836 , 153999 , 447966 , 17755052 , 11409972 , 11712649 , 6419766 , 10074640 , 24779724 , 11234052 , 11364421 , 11338033 , 11427553]

1407|P19784(19) [17755052 , 11485656 , 11314340 , 16224058 , 11409972 , 176167 , 3038522 , 11213558 , 9549303 , 3973 , 6419766 , 11338033 , 5005498 , 208908 , 3025986 , 447966 , 10113978 , 10074640 , 11364421 , 447077 , 11427553 , 156414 , 160355 , 11234052 , 10127622 , 24779724 , 15983966 , 151194 , 153999 , 11712649 , 2856 , 24889392 , 176870 , 2396 , 6918454 , 10427712 , 16722836 , 11656518 , 11667893]

1408|Q07912(20) [11485656 , 6419766 , 10074640 , 160355 , 5005498 , 16722836 , 447966 , 208908 , 151194 , 24779724 , 3973 , 156414 , 3025986 , 9549303 , 447077 , 11712649 , 11667893 , 11213558 , 153999 , 17755052 , 10427712 , 2396 , 11409972 , 15983966 , 24889392 , 2856 , 3038522 , 176167 , 11234052 , 448008 , 176870 , 10113978 , 10127622 , 6918454 , 11338033 , 11314340 , 11427553 , 11656518 , 11364421]

1409|Q6NVW1(0) [11485656 , 3973 , 11409972 , 10127622 , 3038522 , 24779724 , 11712649 , 10427712 , 11234052 , 11314340 , 2396 , 448008 , 11364421 , 11213558 , 11656518 , 17755052 , 6918454 , 156414 , 176167 , 151194 , 447077 , 6419766 , 11338033 , 208908 , 2856 , 3025986 , 447966 , 24889392 , 153999 , 160355 , 16722836 , 15983966 , 11667893 , 9549303 , 10074640 , 176870 , 11427553 , 5005498 , 10113978]

1410|P53667(76) [17755052 , 176870 , 15983966 , 11409972 , 24889392 , 2856 , 10074640 , 24779724 , 2396 , 11364421 , 3973 , 156414 , 6918454 , 5005498 , 11213558 , 9549303 , 11712649 , 10127622 , 11427553 , 3025986 , 447966 , 447077 , 44462760 , 11485656 , 151194 , 11667893 , 10113978 , 3038522 , 160355 , 153999 , 11656518 , 16722836 , 176167 , 208908 , 6419766 , 11314340 , 11234052 , 11338033 , 10427712]

1411|O15146(42) [11667893 , 11485656 , 11213558 , 3038522 , 24889392 , 3973 , 176167 , 5005498 , 208908 , 10127622 , 15983966 , 153999 , 6918454 , 447077 , 11712649 , 176870 , 11427553 , 11338033 , 3025986 , 151194 , 447966 , 11409972 , 17755052 , 2856 , 2396 , 11314340 , 11364421 , 10113978 , 9549303 , 10427712 , 10074640 , 11656518 , 11234052 , 24779724 , 16722836 , 160355 , 156414 , 448008 , 6419766]

1412|P42338(275) [16722836 , 24889392 , 447077 , 11625818 , 10127622 , 11712649 , 11314340 , 176870 , 3038522 , 11234052 , 11364421 , 208908 , 151171 , 15983966 , 11667893 , 11338033 , 11213558 , 11656518 , 156414 , 11409972 , 50905713 , 24779724 , 51001932 , 176167 , 153999 , 11485656 , 3973 , 16736978 , 11647372 , 49784945 , 17755052 , 10074640 , 151194 , 10113978 , 6918454 , 10296883 , 11427553 , 10427712 , 3025986]

1413|P49760(4) [24889392 , 11213558 , 24779724 , 16722836 , 11667893 , 11338033 , 11364421 , 11409972 , 11485656 , 3025986 , 6918454 , 10127622 , 11656518 , 17755052 , 11234052 , 6419766 , 11314340 , 9549303 , 3973 , 3038522 , 2396 , 160355 , 11427553 , 156414 , 448008 , 208908 , 2856 , 176870 , 10074640 , 15983966 , 447966 , 10113978 , 153999 , 5005498 , 11712649 , 10427712 , 176167 , 447077 , 151194]

1414|Q15375(20) [11409972 , 11656518 , 6918454 , 176167 , 24889392 , 447077 , 11712649 , 11485656 , 176870 , 16722836 , 3038522 , 24779724 , 156414 , 11667893 , 15983966 , 2856 , 6419766 , 153999 , 447966 , 11364421 , 2396 , 11213558 , 11338033 , 11314340 , 9549303 , 11234052 , 10074640 , 5005498 , 151194 , 10127622 , 208908 , 3973 , 10427712 , 10113978 , 24875320 , 17755052 , 160355 , 11427553 , 3025986]

1415|P42685(12) [11234052 , 156414 , 11485656 , 10127622 , 15983966 , 3973 , 17755052 , 16722836 , 176167 , 11364421 , 11427553 , 10113978 , 176870 , 160355 , 10427712 , 208908 , 11667893 , 153999 , 448008 , 11167602 , 151194 , 3038522 , 6419766 , 447077 , 24779724 , 2396 , 10074640 , 6918454 , 11314340 , 11656518 , 3025986 , 2856 , 11213558 , 11338033 , 5005498 , 9549303 , 11712649 , 11409972 , 447966 , 24889392]

1416|P29317(63) [153999 , 11485656 , 447077 , 11427553 , 156414 , 3038522 , 10427712 , 11667893 , 176167 , 151194 , 11314340 , 24779724 , 11364421 , 17755052 , 11338033 , 448008 , 16722836 , 11213558 , 10127622 , 2396 , 15983966 , 9549303 , 3025986 , 208908 , 10113978 , 447966 , 6918454 , 10074640 , 11167602 , 3973 , 11656518 , 11409972 , 5005498 , 176870 , 11712649 , 2856 , 24889392 , 11234052 , 160355 , 6419766]

1417|O60285(18) [3973 , 24779724 , 3038522 , 6419766 , 151194 , 153999 , 11314340 , 10127622 , 208908 , 176167 , 6918454 , 447966 , 11427553 , 11485656 , 5330286 , 160355 , 156414 , 448008 , 2856 , 16722836 , 176870 , 10427712 , 17755052 , 15983966 , 9549303 , 11409972 , 5005498 , 10074640 , 11656518 , 3025986 , 11667893 , 11338033 , 2396 , 447077 , 11712649 , 11364421 , 11234052 , 10113978 , 24889392 , 11213558]

1418|P51817(2) [5005498 , 16722836 , 208908 , 25227436 , 6419766 , 151194 , 11234052 , 160355 , 6918454 , 11409972 , 176870 , 11314340 , 11338033 , 11667893 , 447966 , 448008 , 10074640 , 2396 , 24779724 , 3038522 , 447077 , 176167 , 11364421 , 9549303 , 153999 , 11485656 , 10127622 , 24889392 , 11656518 , 156414 , 11427553 , 17755052 , 15983966 , 10113978 , 3025986 , 11213558 , 11712649 , 10427712 , 3973 , 2856]

1419|P14616(15) [11712649 , 11213558 , 15983966 , 11485656 , 3025986 , 10113978 , 3973 , 9549303 , 11656518 , 6918454 , 5005498 , 11409972 , 208908 , 11427553 , 11234052 , 176870 , 447077 , 24889392 , 160355 , 153999 , 2856 , 11338033 , 3038522 , 156414 , 176167 , 151194 , 6419766 , 10427712 , 11364421 , 11667893 , 447966 , 17755052 , 24779724 , 11640390 , 16722836 , 448008 , 2396 , 11314340 , 10074640 , 10127622]

1420|P49840(16) [11364421 , 11667893 , 10074640 , 447077 , 17755052 , 5326739 , 2396 , 11485656 , 3038522 , 11656518 , 11314340 , 24779724 , 176870 , 16722836 , 10427712 , 11409972 , 10127622 , 153999 , 448008 , 156414 , 160355 , 176167 , 2856 , 11338033 , 15983966 , 447966 , 11213558 , 11712649 , 6419766 , 5005498 , 6918454 , 10113978 , 3973 , 11234052 , 151194 , 208908 , 24889392 , 9549303 , 3025986 , 11427553]

1421|P07332(20) [5005498 , 11213558 , 151194 , 11656518 , 11712649 , 11364421 , 176870 , 11234052 , 448008 , 3025986 , 24889392 , 156414 , 11485656 , 160355 , 11338033 , 10113978 , 15983966 , 6419766 , 10427712 , 447077 , 3973 , 10074640 , 2396 , 208908 , 11427553 , 11667893 , 9549303 , 176167 , 10127622 , 24779724 , 6918454 , 17755052 , 153999 , 2856 , 3038522 , 16722836 , 45142457 , 447966 , 11409972 , 11314340]

1422|P49761(0) [10074640 , 10127622 , 10113978 , 153999 , 5005498 , 10427712 , 176167 , 11213558 , 24779724 , 11656518 , 156414 , 11338033 , 208908 , 2856 , 24889392 , 176870 , 24748573 , 3025986 , 6918454 , 17755052 , 11712649 , 11234052 , 6419766 , 447077 , 151194 , 9549303 , 3973 , 11364421 , 447966 , 3038522 , 2396 , 15983966 , 11485656 , 11314340 , 16722836 , 11667893 , 160355 , 11427553 , 448008 , 11409972]

1423|P78368(6) [24779724 , 3025986 , 11364421 , 15983966 , 208908 , 2396 , 3973 , 24889392 , 160355 , 11712649 , 11234052 , 17755052 , 10113978 , 151194 , 10127622 , 6918454 , 6419766 , 176870 , 156414 , 11427553 , 5005498 , 11213558 , 447966 , 2856 , 448008 , 11667893 , 16722836 , 11485656 , 3038522 , 11314340 , 176167 , 129236 , 153999 , 10427712 , 9549303 , 447077 , 11656518 , 11409972 , 11338033 , 10074640]

1424|Q06418(33) [17755052 , 176167 , 24889392 , 448008 , 11427553 , 11213558 , 447966 , 3973 , 3025986 , 11712649 , 11314340 , 11234052 , 11656518 , 15983966 , 10427712 , 2396 , 24794418 , 11364421 , 10127622 , 447077 , 156414 , 10074640 , 11338033 , 5005498 , 3038522 , 6419766 , 151194 , 6918454 , 11409972 , 208908 , 9549303 , 16722836 , 2856 , 160355 , 11667893 , 24779724 , 153999 , 176870 , 10113978 , 11485656]

1425|Q13627(29) [11364421 , 160355 , 11712649 , 151194 , 447077 , 153999 , 11234052 , 11409972 , 176167 , 5005498 , 24779724 , 448008 , 24894158 , 3025986 , 10172943 , 156414 , 6918454 , 16722836 , 208908 , 10127622 , 3078519 , 10427712 , 3542 , 10074640 , 17755052 , 1694 , 3038522 , 11667893 , 3973 , 3540 , 11213558 , 176870 , 15983966 , 10113978 , 11338033 , 11485656 , 11314340 , 11656518 , 11427553 , 24889392]

1426|Q15303(94) [11314340 , 10074640 , 176167 , 16722836 , 11712649 , 6918454 , 2856 , 17755052 , 11485656 , 11213558 , 11234052 , 10427712 , 447077 , 11667893 , 3025986 , 11338033 , 3973 , 15983966 , 24779724 , 447966 , 6419766 , 448008 , 208908 , 5005498 , 11409972 , 10437018 , 10127622 , 11656518 , 11511120 , 11364421 , 9549303 , 153999 , 156414 , 24889392 , 11427553 , 2396 , 176870 , 3038522 , 10113978 , 151194]

1427|Q12866(58) [11485656 , 153999 , 11656518 , 6918454 , 10113978 , 11234052 , 3038522 , 17755052 , 2856 , 11712649 , 10427712 , 11667893 , 9549303 , 176167 , 15983966 , 2396 , 24794418 , 176870 , 151194 , 11364421 , 11338033 , 6419766 , 11427553 , 11314340 , 10074640 , 447966 , 3973 , 11213558 , 24889392 , 24779724 , 156414 , 11409972 , 5005498 , 448008 , 10127622 , 3025986 , 208908 , 447077 , 160355 , 16722836 , 45142457]

1428|P08922(112) [11667893 , 15983966 , 448008 , 11427553 , 57379345 , 153999 , 11712649 , 156414 , 176870 , 24889392 , 208908 , 10127622 , 10074640 , 5005498 , 9549303 , 10113978 , 176167 , 11656518 , 11338033 , 11409972 , 6918454 , 11234052 , 17755052 , 16722836 , 447077 , 11626560 , 3973 , 160355 , 447966 , 151194 , 10427712 , 3038522 , 11314340 , 11364421 , 6419766 , 24779724 , 11485656 , 2856 , 3025986 , 11213558 , 2396]

1429|Q96L35(0) [208908 , 176870 , 6419766 , 153999 , 11364421 , 3025986 , 15983966 , 10127622 , 24889392 , 160355 , 24779724 , 10113978 , 5005498 , 447077 , 11213558 , 11485656 , 3038522 , 17755052 , 10458325 , 11409972 , 2856 , 10427712 , 448008 , 11427553 , 151194 , 11338033 , 16722836 , 9549303 , 6918454 , 11656518 , 176167 , 447966 , 11667893 , 11234052 , 2396 , 11712649 , 11314340 , 10296883 , 156414 , 3973 , 10074640]

1430|P30530(68) [11213558 , 208908 , 17755052 , 5005498 , 447966 , 176167 , 6419766 , 11338033 , 2856 , 11667893 , 24889392 , 3025986 , 9549303 , 176870 , 16722836 , 6918454 , 15983966 , 10113978 , 10074640 , 11409972 , 24794418 , 10296883 , 10127622 , 3038522 , 24779724 , 2396 , 11485656 , 3973 , 11427553 , 448008 , 11712649 , 11234052 , 10427712 , 11314340 , 151194 , 11656518 , 153999 , 11364421 , 156414 , 160355 , 447077]

1431|Q14289(99) [3038522 , 6419766 , 11712649 , 11314340 , 17755052 , 11234052 , 447966 , 16722836 , 24889392 , 11338033 , 447077 , 10074640 , 11364421 , 11656518 , 3239295 , 208908 , 176167 , 5005498 , 4566 , 11485656 , 2396 , 176870 , 153999 , 160355 , 15983966 , 11667893 , 10113978 , 2856 , 6918454 , 3025986 , 11427553 , 9549303 , 156414 , 11213558 , 10427712 , 11409972 , 10127622 , 24779724 , 448008 , 151194 , 3973]

1432|Q16832(38) [160355 , 11167602 , 448008 , 16722836 , 24779724 , 3038522 , 216239 , 11712649 , 10113978 , 11485656 , 447077 , 10427712 , 3973 , 208908 , 10127622 , 5005498 , 24889392 , 176167 , 11234052 , 9549303 , 11338033 , 11213558 , 11364421 , 3025986 , 447966 , 6419766 , 2856 , 11314340 , 153999 , 17755052 , 11667893 , 15983966 , 156414 , 11656518 , 176870 , 11409972 , 151194 , 10074640 , 6918454 , 2396 , 11427553]

1433|Q8TDC3(3) [448008 , 447966 , 153999 , 11338033 , 10074640 , 11712649 , 11485656 , 11314340 , 11364421 , 3038522 , 24779724 , 15983966 , 5005498 , 6419766 , 160355 , 208908 , 11234052 , 2856 , 17755052 , 10127622 , 9549303 , 176870 , 16722836 , 10113978 , 176167 , 11608401 , 2396 , 3973 , 11667893 , 11427553 , 76098 , 11409972 , 3025986 , 11656518 , 10427712 , 156414 , 6918454 , 16122633 , 24889392 , 151194 , 447077 , 11213558]

1434|P43405(61) [447966 , 11712649 , 10127622 , 24889392 , 3973 , 176167 , 151194 , 208908 , 11427553 , 10427712 , 11213558 , 11364421 , 448008 , 11671467 , 447077 , 57379345 , 11409972 , 11485656 , 17755052 , 11667893 , 11234052 , 6419766 , 6918454 , 16722836 , 11656518 , 10296883 , 2396 , 5005498 , 3038522 , 2856 , 9549303 , 10113978 , 24779724 , 160355 , 10074640 , 153999 , 156414 , 15983966 , 176870 , 11314340 , 11338033 , 3025986]

1435|P47712(81) [45482332 , 45482343 , 45482335 , 44562673 , 45482341 , 204106 , 44562729 , 45482337 , 44563073 , 45482339 , 44346400 , 44563075 , 44563072 , 9853499 , 44563074 , 11371200 , 45482342 , 11360688 , 44563043 , 16118319 , 16118318 , 45482340 , 4670 , 3033993 , 10125241 , 16118461 , 25166397 , 9906670 , 45482333 , 44241583 , 197197 , 45482338 , 44563042 , 16118459 , 44330207 , 45482334 , 16118320 , 11372460 , 44329617 , 44562672 , 44562726 , 44346482]

1436|Q9BZL6(14) [11712649 , 11338033 , 156414 , 6419766 , 151194 , 16122633 , 11364421 , 5005498 , 11656518 , 24779724 , 3038522 , 11213558 , 208908 , 3973 , 10074640 , 17755052 , 11608401 , 11427553 , 9549303 , 11667893 , 153999 , 448008 , 3025986 , 10427712 , 11234052 , 11409972 , 11485656 , 24889392 , 447077 , 2856 , 15983966 , 6918454 , 10127622 , 160355 , 176167 , 2396 , 10113978 , 11314340 , 76098 , 447966 , 176870 , 16722836]

1437|O00329(287) [10113978 , 10296883 , 11656518 , 151194 , 10074640 , 11234052 , 3973 , 24779724 , 11712649 , 50905713 , 11338033 , 16722836 , 10427712 , 3038522 , 49784945 , 176870 , 176167 , 17755052 , 51001932 , 151171 , 10127622 , 15983966 , 5330286 , 447077 , 11314340 , 156414 , 6918454 , 11485656 , 6852165 , 3025986 , 16736978 , 11625818 , 11647372 , 11427553 , 11409972 , 153999 , 24889392 , 11667893 , 448008 , 11213558 , 208908 , 11364421]

1438|Q15746(54) [151194 , 447966 , 11409972 , 24889392 , 16722836 , 11712649 , 11338033 , 176870 , 11667893 , 3542 , 11234052 , 10172943 , 3025986 , 17755052 , 10127622 , 11364421 , 3540 , 11314340 , 156414 , 3078519 , 9549303 , 10113978 , 10074640 , 3038522 , 6918454 , 24779724 , 11213558 , 15983966 , 11427553 , 160355 , 2396 , 2856 , 11485656 , 447077 , 3973 , 153999 , 208908 , 6419766 , 5005498 , 176167 , 11656518 , 10427712]

1439|Q13554(7) [10074640 , 11427553 , 10427712 , 156414 , 10113978 , 153999 , 11656518 , 11409972 , 76098 , 11213558 , 176870 , 176167 , 5005498 , 2856 , 11712649 , 208908 , 24889392 , 11314340 , 11338033 , 11234052 , 24779724 , 447077 , 447966 , 16122633 , 10127622 , 11364421 , 6419766 , 6918454 , 23624249 , 11667893 , 9549303 , 3025986 , 17755052 , 15983966 , 151194 , 2396 , 16722836 , 160355 , 11608401 , 3973 , 11485656 , 3038522]

1440|Q96RI1(93) [10168 , 28803 , 10245972 , 39042 , 16362 , 11852 , 3401 , 10868 , 10229498 , 1989 , 104741 , 10026128 , 2750 , 2812 , 16181 , 7346 , 41684 , 6540 , 7329 , 37175 , 16734800 , 115157 , 21138 , 520196 , 8178 , 2391 , 62485 , 5326713 , 8041 , 447715 , 2998 , 3034285 , 3503 , 31253 , 16316 , 19996 , 5289501 , 6 , 8907 , 10133 , 3082 , 3333]

1441|Q7KZI7(112) [11338033 , 17755052 , 11712649 , 3038522 , 9549303 , 16722836 , 176167 , 2856 , 11608401 , 11667893 , 10427712 , 3025986 , 11213558 , 5005498 , 11234052 , 447966 , 208908 , 448008 , 10074640 , 10127622 , 160355 , 447077 , 11364421 , 156414 , 3973 , 15983966 , 176870 , 11485656 , 76098 , 16122633 , 6419766 , 24779724 , 153999 , 11314340 , 6918454 , 10113978 , 11427553 , 24889392 , 2396 , 11656518 , 11409972 , 151194]

1442|Q8N568(0) [6918454 , 208908 , 10127622 , 447966 , 16122633 , 16722836 , 176870 , 176167 , 76098 , 9549303 , 11712649 , 160355 , 153999 , 11314340 , 3025986 , 11409972 , 11234052 , 11485656 , 10074640 , 10427712 , 11608401 , 6419766 , 11338033 , 11427553 , 11656518 , 2396 , 11364421 , 24889392 , 448008 , 3038522 , 5005498 , 17755052 , 156414 , 447077 , 2856 , 11667893 , 10113978 , 24779724 , 3973 , 151194 , 15983966 , 11213558]

1443|Q04759(25) [3973 , 2856 , 11608401 , 17755052 , 447077 , 15983966 , 3038522 , 10074640 , 176870 , 156414 , 3025986 , 208908 , 11485656 , 11314340 , 76098 , 447966 , 11712649 , 11234052 , 11338033 , 11364421 , 11656518 , 160355 , 11427553 , 24779724 , 24889392 , 448008 , 176167 , 11213558 , 10427712 , 16722836 , 5005498 , 6419766 , 11667893 , 153999 , 2396 , 9549303 , 151194 , 16122633 , 10127622 , 11409972 , 10113978 , 6918454]

1444|Q14012(7) [2856 , 15983966 , 24779724 , 6419766 , 10172943 , 176870 , 153999 , 11234052 , 11409972 , 10074640 , 11712649 , 10427712 , 3038522 , 3078519 , 160355 , 3973 , 3542 , 11485656 , 11656518 , 5005498 , 6918454 , 176167 , 151194 , 11213558 , 11427553 , 11314340 , 9549303 , 156414 , 16722836 , 2396 , 11667893 , 208908 , 11338033 , 3025986 , 447966 , 10113978 , 17755052 , 447077 , 3540 , 24889392 , 10127622 , 11364421]

1445|P11802(160) [57379345 , 11656518 , 5330286 , 11667893 , 176167 , 6918852 , 3038522 , 16722836 , 156414 , 5289419 , 11409972 , 151194 , 46220502 , 5287969 , 6918834 , 11314340 , 448008 , 448991 , 11610113 , 176870 , 10427712 , 11485656 , 15983966 , 447077 , 3025986 , 153999 , 24779724 , 208908 , 11234052 , 11364421 , 11213558 , 11338033 , 5327686 , 24889392 , 11712649 , 10074640 , 11285002 , 10113978 , 11427553 , 6918454 , 10127622 , 17755052]

1446|Q9H2X6(32) [2396 , 153999 , 11712649 , 9549303 , 24779724 , 15983966 , 10172943 , 176870 , 6419766 , 11364421 , 156414 , 447077 , 3540 , 11338033 , 5005498 , 208908 , 3025986 , 160355 , 11485656 , 10127622 , 3973 , 447966 , 16722836 , 10427712 , 11667893 , 17755052 , 6918454 , 10074640 , 176167 , 151194 , 11314340 , 11213558 , 11656518 , 3542 , 11234052 , 2856 , 10113978 , 11409972 , 3038522 , 24889392 , 11427553 , 3078519]

1447|Q13557(8) [447966 , 176870 , 2396 , 447077 , 10427712 , 208908 , 448008 , 11712649 , 3038522 , 11314340 , 11427553 , 10074640 , 10113978 , 153999 , 11364421 , 11213558 , 3973 , 24889392 , 176167 , 11667893 , 5005498 , 15983966 , 16722836 , 151194 , 2856 , 11608401 , 160355 , 11338033 , 11234052 , 24779724 , 6419766 , 16122633 , 10127622 , 6918454 , 11409972 , 11485656 , 76098 , 9549303 , 3025986 , 11656518 , 156414 , 17755052]

1448|Q13976(65) [176167 , 11409972 , 2396 , 447966 , 17755052 , 153999 , 9549303 , 24889392 , 10127622 , 2856 , 11213558 , 208908 , 447077 , 11364421 , 11656518 , 3025986 , 10074640 , 3973 , 24779724 , 11712649 , 11427553 , 5005498 , 11338033 , 176870 , 160355 , 11667893 , 16122633 , 448008 , 11608401 , 10113978 , 15983966 , 151194 , 11234052 , 11314340 , 76098 , 156414 , 16722836 , 6918454 , 11485656 , 10427712 , 6419766 , 3038522]

1449|Q13464(52) [24889392 , 447077 , 6419766 , 6918454 , 3025986 , 10074640 , 2396 , 16722836 , 24779724 , 5005498 , 176167 , 11667893 , 153999 , 448008 , 3973 , 11409972 , 2856 , 11314340 , 3038522 , 208908 , 151194 , 10113978 , 11364421 , 11234052 , 10127622 , 11427553 , 11338033 , 11552706 , 11656518 , 448042 , 9549303 , 25227436 , 17755052 , 15983966 , 10296883 , 447966 , 11213558 , 11485656 , 11712649 , 156414 , 176870 , 10427712]

1450|Q16513(4) [10427712 , 24779724 , 447966 , 11213558 , 11314340 , 15983966 , 151194 , 11656518 , 11427553 , 6918454 , 10127622 , 208908 , 11409972 , 3078519 , 11712649 , 9549303 , 11364421 , 11667893 , 3025986 , 11485656 , 10074640 , 2856 , 153999 , 24889392 , 11338033 , 5005498 , 3542 , 447077 , 176167 , 3973 , 2396 , 16722836 , 160355 , 3038522 , 176870 , 11234052 , 10172943 , 17755052 , 3540 , 10113978 , 6419766 , 156414]

1451|Q9NWZ3(35) [16722836 , 11314340 , 3025986 , 2396 , 17755052 , 176870 , 5005498 , 208908 , 447077 , 10074640 , 15983966 , 11667893 , 11409972 , 3038522 , 6419766 , 10113978 , 11427553 , 76098 , 151194 , 24779724 , 11485656 , 10427712 , 24889392 , 11364421 , 11338033 , 11213558 , 11608401 , 448008 , 16122633 , 176167 , 447966 , 156414 , 153999 , 11234052 , 11987888 , 3973 , 11656518 , 6918454 , 10127622 , 9549303 , 11712649 , 2856]

1452|P24723(21) [16722836 , 76098 , 447966 , 10074640 , 11234052 , 5005498 , 6419766 , 2856 , 160355 , 11656518 , 15983966 , 11364421 , 447077 , 3025986 , 156414 , 11608401 , 10427712 , 11314340 , 9549303 , 24889392 , 11712649 , 11213558 , 3973 , 24779724 , 10113978 , 11338033 , 17755052 , 11667893 , 153999 , 10127622 , 176167 , 6918454 , 11427553 , 2396 , 16122633 , 11409972 , 10114 , 448008 , 208908 , 176870 , 3038522 , 11485656 , 151194]

1453|O75676(1) [16122633 , 448008 , 10074640 , 153999 , 24779724 , 10127622 , 2396 , 447077 , 10427712 , 11485656 , 9549303 , 6419766 , 15983966 , 208908 , 10113978 , 24889392 , 11427553 , 76098 , 11234052 , 3025986 , 11656518 , 25227436 , 156414 , 11712649 , 6918454 , 5005498 , 11338033 , 3973 , 3038522 , 447966 , 11667893 , 176167 , 151194 , 11213558 , 16722836 , 17755052 , 176870 , 11608401 , 2856 , 11314340 , 11409972 , 160355 , 11364421]

1454|Q14680(18) [151194 , 2396 , 24779724 , 176167 , 176870 , 3025986 , 16722836 , 11409972 , 10074640 , 447077 , 11213558 , 3542 , 11338033 , 24889392 , 11427553 , 447966 , 156414 , 10113978 , 11364421 , 6419766 , 10172943 , 5005498 , 11234052 , 11485656 , 160355 , 3078519 , 3973 , 3540 , 153999 , 11712649 , 448008 , 10127622 , 10427712 , 2856 , 11667893 , 6918454 , 15983966 , 9549303 , 11656518 , 3038522 , 208908 , 17755052 , 11314340]

1455|Q8TDX7(4) [11667893 , 11485656 , 448008 , 17755052 , 11314340 , 10427712 , 176167 , 3038522 , 156414 , 11427553 , 6419766 , 16722836 , 6918454 , 160355 , 2396 , 153999 , 11409972 , 11712649 , 2856 , 3542 , 151194 , 15983966 , 11656518 , 24779724 , 10127622 , 10074640 , 5005498 , 3540 , 208908 , 3973 , 11338033 , 3025986 , 11234052 , 10172943 , 9549303 , 447966 , 447077 , 11364421 , 24889392 , 3078519 , 11213558 , 176870 , 10113978]

1456|Q96SB4(22) [3025986 , 17755052 , 10127622 , 11234052 , 447966 , 11667893 , 208908 , 24779724 , 176870 , 10074640 , 448008 , 151194 , 11712649 , 11314340 , 16722836 , 3078519 , 11485656 , 3038522 , 3973 , 11409972 , 6918454 , 153999 , 11656518 , 3542 , 176167 , 2396 , 9549303 , 10113978 , 5005498 , 2856 , 6419766 , 10172943 , 156414 , 11213558 , 10427712 , 447077 , 11427553 , 11338033 , 24889392 , 11364421 , 3540 , 15983966 , 160355]

1457|O75604(10) [655601 , 3151 , 3760 , 3926 , 4156 , 228244 , 2799 , 2333 , 2247 , 10219 , 4030 , 54677971 , 5289501 , 6307 , 24144 , 2170 , 4343310 , 10206 , 2540 , 5722 , 2812 , 12454 , 2478 , 3132640 , 2090 , 2178 , 1780 , 4842 , 6708773 , 16129778 , 3108 , 655083 , 2453 , 32681 , 3474 , 2318 , 54677972 , 13791 , 19675 , 5405 , 16362 , 54676538 , 3503]

1458|Q9NQU5(10) [10113978 , 11338033 , 5005498 , 151194 , 208908 , 6419766 , 156414 , 11213558 , 176870 , 2396 , 11234052 , 16722836 , 2856 , 447966 , 10074640 , 3078519 , 6918454 , 11656518 , 11667893 , 176167 , 15983966 , 3542 , 10127622 , 160355 , 3038522 , 448008 , 11712649 , 3973 , 153999 , 11485656 , 3025986 , 11314340 , 17755052 , 24889392 , 9549303 , 11427553 , 447077 , 24779724 , 10427712 , 3540 , 11409972 , 11364421 , 10172943]

1459|Q92630(10) [11409972 , 176870 , 16722836 , 17755052 , 10074640 , 3540 , 10427712 , 9549303 , 5005498 , 176167 , 6419766 , 3542 , 160355 , 10113978 , 151194 , 11314340 , 11712649 , 6918454 , 11213558 , 153999 , 2856 , 11667893 , 24889392 , 3038522 , 2396 , 447077 , 3973 , 15983966 , 3078519 , 448008 , 10172943 , 10127622 , 24779724 , 11234052 , 156414 , 11364421 , 11656518 , 11485656 , 3025986 , 11338033 , 11427553 , 447966 , 208908]

1460|P08631(8) [9549303 , 17755052 , 176167 , 24889392 , 11608401 , 11656518 , 6918454 , 11409972 , 3973 , 3038522 , 2396 , 151194 , 16122633 , 176870 , 24779724 , 208908 , 448008 , 11314340 , 76098 , 11712649 , 10074640 , 11485656 , 153999 , 11234052 , 10113978 , 447077 , 11667893 , 447966 , 11364421 , 10127622 , 6419766 , 15983966 , 11427553 , 10296883 , 160355 , 11213558 , 3025986 , 156414 , 16722836 , 2856 , 5005498 , 11338033 , 10427712]

1461|P43403(55) [11667893 , 17755052 , 11234052 , 6419766 , 6918454 , 2856 , 2396 , 11485656 , 5005498 , 10113978 , 11656518 , 10296883 , 4566 , 9549303 , 10074640 , 153999 , 160355 , 11314340 , 11427553 , 3025986 , 156414 , 15983966 , 176870 , 11338033 , 11712649 , 10127622 , 3973 , 447966 , 16722836 , 176167 , 24889392 , 151194 , 208908 , 11213558 , 11364421 , 10427712 , 24779724 , 448008 , 11409972 , 447077 , 57379345 , 3038522 , 11442891]

1462|Q9P286(14) [208908 , 11213558 , 447966 , 11656518 , 10172943 , 16722836 , 10127622 , 176870 , 176167 , 9549303 , 3540 , 160355 , 11234052 , 11712649 , 153999 , 11314340 , 3025986 , 11409972 , 10427712 , 11485656 , 10074640 , 6419766 , 11338033 , 2396 , 11427553 , 3973 , 11364421 , 448008 , 17755052 , 156414 , 3038522 , 447077 , 3542 , 5005498 , 3078519 , 24889392 , 2856 , 11667893 , 10113978 , 151194 , 15983966 , 24779724 , 6918454]

1463|Q02156(45) [76098 , 11427553 , 16122633 , 4064 , 208908 , 160355 , 6918454 , 24889392 , 11314340 , 11667893 , 447077 , 24779724 , 447966 , 2396 , 10074640 , 5005498 , 9549303 , 3025986 , 11338033 , 3973 , 16722836 , 2856 , 153999 , 11213558 , 11234052 , 11712649 , 11364421 , 6918670 , 156414 , 11485656 , 11409972 , 11656518 , 15983966 , 17755052 , 10113978 , 10427712 , 11608401 , 176167 , 10127622 , 176870 , 151194 , 3038522 , 6419766]

1464|Q9HC98(8) [3078519 , 176870 , 2396 , 447077 , 11712649 , 448008 , 153999 , 3973 , 11314340 , 3038522 , 2856 , 160355 , 11427553 , 11667893 , 10113978 , 24889392 , 10127622 , 10074640 , 15983966 , 10427712 , 3540 , 11364421 , 151194 , 10172943 , 11213558 , 447966 , 176167 , 3542 , 11338033 , 5005498 , 11234052 , 16722836 , 6419766 , 24779724 , 11485656 , 3025986 , 6918454 , 17755052 , 11409972 , 208908 , 9549303 , 11656518 , 156414]

1465|P51813(17) [11712649 , 3973 , 2856 , 11656518 , 16722836 , 208908 , 151194 , 11234052 , 24889392 , 5005498 , 11409972 , 160355 , 6918454 , 176870 , 4708 , 448008 , 11338033 , 11314340 , 447966 , 11667893 , 2396 , 10074640 , 11608401 , 3038522 , 447077 , 176167 , 16122633 , 24779724 , 76098 , 9549303 , 11364421 , 153999 , 11485656 , 10127622 , 156414 , 6419766 , 3025986 , 11427553 , 15983966 , 10113978 , 10427712 , 11213558 , 17755052]

1466|Q9BXA7(6) [153999 , 10127622 , 11234052 , 11656518 , 11364421 , 11338033 , 6419766 , 11485656 , 3025986 , 447966 , 151194 , 156414 , 11427553 , 2856 , 57379345 , 3973 , 10074640 , 10427712 , 9549303 , 24889392 , 11608401 , 10113978 , 11314340 , 160355 , 11712649 , 11213558 , 176167 , 6918454 , 76098 , 208908 , 11409972 , 24779724 , 17755052 , 176870 , 11667893 , 3038522 , 2396 , 15983966 , 448008 , 447077 , 5005498 , 16122633 , 16722836]

1467|Q9H422(3) [11364421 , 156414 , 15983966 , 3038522 , 208908 , 176167 , 11667893 , 447966 , 3542 , 6419766 , 9549303 , 11712649 , 10074640 , 11213558 , 3540 , 2856 , 11427553 , 24748573 , 11485656 , 2396 , 6918454 , 447077 , 5005498 , 11338033 , 176870 , 3973 , 11409972 , 10172943 , 16722836 , 3078519 , 153999 , 24889392 , 10113978 , 24779724 , 10127622 , 10427712 , 151194 , 11234052 , 160355 , 11314340 , 17755052 , 3025986 , 11656518]

1468|Q86V86(19) [447077 , 10172943 , 11409972 , 176870 , 2856 , 11364421 , 448008 , 3025986 , 17755052 , 10113978 , 24795070 , 11712649 , 11338033 , 2396 , 9549303 , 24779724 , 11485656 , 11213558 , 6918454 , 447966 , 11667893 , 3973 , 24889392 , 6419766 , 11427553 , 5005498 , 15983966 , 11314340 , 156414 , 10127622 , 208908 , 10074640 , 3540 , 11234052 , 153999 , 151194 , 3038522 , 10427712 , 3542 , 176167 , 16722836 , 11656518 , 160355 , 3078519]

1469|Q9HBH9(5) [2856 , 153999 , 11213558 , 11667893 , 208908 , 447966 , 17755052 , 3025986 , 16722836 , 160355 , 3542 , 10113978 , 11364421 , 24889392 , 3540 , 11427553 , 3038522 , 448008 , 3973 , 10074640 , 57379345 , 11712649 , 6419766 , 176870 , 10172943 , 3078519 , 5005498 , 156414 , 24779724 , 11234052 , 176167 , 447077 , 10127622 , 15983966 , 2396 , 10427712 , 11656518 , 6918454 , 11409972 , 9549303 , 11338033 , 151194 , 11314340 , 11485656]

1470|Q9UM73(138) [176870 , 24779724 , 11234052 , 16722836 , 11338033 , 151194 , 11712649 , 11656518 , 10296883 , 11364421 , 10074640 , 10113978 , 10427712 , 57379345 , 208908 , 156414 , 6419766 , 9549303 , 448008 , 2396 , 11213558 , 160355 , 11409972 , 11667893 , 153999 , 24889392 , 11626560 , 49806720 , 11427553 , 3025986 , 11485656 , 11751922 , 6918454 , 447077 , 11314340 , 15983966 , 10127622 , 2856 , 17755052 , 5005498 , 3038522 , 176167 , 3973 , 447966]

1471|Q05655(90) [10427712 , 17755052 , 447077 , 11608401 , 24889392 , 16122633 , 151194 , 24779724 , 10114 , 2856 , 11314340 , 3038522 , 6918670 , 6419766 , 208908 , 11364421 , 11213558 , 156414 , 15983966 , 176167 , 9549303 , 11656518 , 10113978 , 11667893 , 6918454 , 76098 , 447966 , 11409972 , 16722836 , 11234052 , 10074640 , 10127622 , 11427553 , 2396 , 176870 , 10569483 , 3025986 , 160355 , 11485656 , 3973 , 11712649 , 11338033 , 5005498 , 153999]

1472|Q13177(22) [176167 , 9549303 , 76098 , 160355 , 153999 , 3025986 , 16122633 , 24779724 , 11213558 , 15983966 , 10427712 , 6918454 , 208908 , 6419766 , 447966 , 11427553 , 11656518 , 176870 , 3973 , 448008 , 3038522 , 11712649 , 11314340 , 17755052 , 11485656 , 10074640 , 11409972 , 447077 , 11608401 , 2856 , 57379345 , 11338033 , 11667893 , 10113978 , 2396 , 11234052 , 151194 , 11364421 , 10127622 , 24889392 , 10296883 , 16722836 , 5005498 , 156414]

1473|P45984(47) [11656518 , 176167 , 11667893 , 3038522 , 5005498 , 17755052 , 11409972 , 3542 , 15983966 , 3973 , 151194 , 176870 , 447966 , 11213558 , 2856 , 10296883 , 3078519 , 448008 , 11314340 , 2396 , 447077 , 10113978 , 24779724 , 11364421 , 208908 , 160355 , 11485656 , 11234052 , 10172943 , 6419766 , 10427712 , 11712649 , 10074640 , 11427553 , 11285002 , 24889392 , 9549303 , 10127622 , 153999 , 16722836 , 6918454 , 3540 , 3025986 , 156414 , 11338033]

1474|Q5JY90(0) [3025986 , 11485656 , 16722836 , 448008 , 15983966 , 11314340 , 160355 , 176167 , 11409972 , 3038522 , 6419766 , 57379345 , 11427553 , 10296883 , 156414 , 11656518 , 17755052 , 24779724 , 6918454 , 10113978 , 11667893 , 2396 , 11712649 , 10074640 , 11608401 , 153999 , 2856 , 176870 , 10127622 , 151194 , 9549303 , 16122633 , 5005498 , 447966 , 3973 , 76098 , 11234052 , 447077 , 10427712 , 24889392 , 11364421 , 11751922 , 208908 , 11213558 , 11338033]

1475|P07333(93) [3025986 , 11485656 , 160355 , 11338033 , 10113978 , 6419766 , 11751922 , 10427712 , 3973 , 2396 , 11409972 , 176167 , 11314340 , 24779724 , 11427553 , 6918454 , 24767976 , 151194 , 17755052 , 153999 , 9884318 , 11656518 , 11712649 , 176870 , 16722836 , 3038522 , 24889392 , 156414 , 15983966 , 5330286 , 447077 , 448008 , 10366136 , 208908 , 11667893 , 447966 , 5005498 , 11213558 , 10127622 , 11364421 , 2856 , 9933475 , 11234052 , 10074640 , 9549303]

1476|Q9P1W9(20) [10074640 , 11427553 , 10113978 , 160355 , 10127622 , 3540 , 153999 , 24748573 , 3038522 , 10427712 , 6918454 , 9549303 , 24779724 , 447966 , 3078519 , 10172943 , 5005498 , 176870 , 11213558 , 11656518 , 16722836 , 3025986 , 2856 , 11364421 , 11338033 , 11234052 , 208908 , 11314340 , 24795070 , 11712649 , 151194 , 3973 , 447077 , 448008 , 11485656 , 24889392 , 2396 , 156414 , 15983966 , 11667893 , 11409972 , 17755052 , 3542 , 176167 , 6419766]

1477|O15530(24) [24889392 , 24779724 , 3025986 , 11314340 , 10127622 , 10427712 , 11213558 , 11364421 , 6419766 , 176870 , 3078519 , 10074640 , 448008 , 447077 , 11485656 , 2396 , 447966 , 17755052 , 2856 , 11442891 , 15983966 , 11409972 , 10027278 , 11712649 , 3540 , 11656518 , 11338033 , 6918454 , 11667893 , 11234052 , 9549303 , 160355 , 151194 , 208908 , 3973 , 11427553 , 10113978 , 153999 , 10172943 , 156414 , 5005498 , 16722836 , 3038522 , 176167 , 3542]

1478|O96013(23) [24779724 , 17755052 , 10172943 , 25227462 , 10113978 , 11485656 , 3078519 , 10074640 , 160355 , 156414 , 6918454 , 11213558 , 11427553 , 3540 , 9549303 , 16058637 , 11712649 , 10427712 , 176167 , 11314340 , 6419766 , 3542 , 11234052 , 3973 , 208908 , 2396 , 447966 , 3025986 , 24889392 , 15983966 , 11338033 , 151194 , 447077 , 176870 , 3038522 , 2856 , 11364421 , 16722836 , 11442891 , 153999 , 11656518 , 11409972 , 5005498 , 11667893 , 10127622]

1479|Q99549(1) [4593 , 3973 , 4278 , 2179 , 1547484 , 3117 , 10382715 , 2327 , 5233 , 2170 , 3333 , 1694 , 361655 , 5405 , 101616 , 2812 , 4362 , 53708 , 2913 , 1967 , 36811 , 21138 , 3503 , 4843 , 4493 , 5227 , 5074 , 3455 , 2265 , 16362 , 114924 , 2754 , 133621 , 5289501 , 3151 , 23897 , 1045 , 2717 , 3542 , 3885 , 6603842 , 1649 , 2277 , 3311 , 20544]

1480|P07947(19) [9549303 , 11338033 , 151194 , 25066467 , 15983966 , 10302451 , 3025986 , 24889392 , 176870 , 11364421 , 11712649 , 153999 , 59174488 , 10297043 , 3973 , 10127622 , 24779724 , 5005498 , 10074640 , 11314340 , 2856 , 156414 , 11667893 , 16722836 , 6918454 , 2396 , 6419766 , 447077 , 24795070 , 24826799 , 11427553 , 160355 , 17755052 , 10113978 , 208908 , 3038522 , 11234052 , 448008 , 11485656 , 176167 , 11409972 , 10427712 , 11213558 , 447966 , 11656518 , 11442891]

1481|P51955(36) [11667893 , 176167 , 10427712 , 11485656 , 11409972 , 3540 , 2396 , 151194 , 447966 , 10172943 , 11656518 , 11338033 , 153999 , 6918454 , 160355 , 2856 , 11608401 , 11427553 , 24779724 , 24889392 , 10113978 , 11213558 , 448008 , 15983966 , 5005498 , 3542 , 3038522 , 10074640 , 11314340 , 16122633 , 6419766 , 11364421 , 17755052 , 11712649 , 3025986 , 9549303 , 76098 , 10127622 , 11234052 , 3973 , 447077 , 176870 , 16722836 , 208908 , 3078519 , 156414]

1482|P48730(20) [11364421 , 11427553 , 10113978 , 160355 , 10074640 , 11667893 , 153999 , 17755052 , 24779724 , 129236 , 6419766 , 447077 , 11314340 , 11712649 , 9549303 , 2856 , 3038522 , 3542 , 3078519 , 16722836 , 151194 , 156414 , 2396 , 11656518 , 6918454 , 5005498 , 15983966 , 10127622 , 24889392 , 447966 , 176870 , 10172943 , 11608401 , 76098 , 16122633 , 11234052 , 11338033 , 11213558 , 3025986 , 3540 , 10427712 , 11485656 , 3973 , 176167 , 208908 , 11409972]

1483|P23443(65) [3973 , 2750 , 11213558 , 6918454 , 11409972 , 9549303 , 6419766 , 11427553 , 151194 , 11314340 , 17755052 , 24779724 , 3078519 , 176870 , 10267580 , 11667893 , 3025986 , 10074640 , 11485656 , 153999 , 11364421 , 176167 , 2396 , 11712649 , 2856 , 448008 , 15983966 , 3038522 , 160355 , 208908 , 156414 , 10172943 , 10427712 , 24889392 , 11656518 , 3540 , 447966 , 10127622 , 10113978 , 11234052 , 11338033 , 1694 , 447077 , 16722836 , 5005498 , 3542]

1484|Q8IWQ3(2) [11656518 , 24889392 , 3078519 , 3540 , 10172943 , 2856 , 9549303 , 11213558 , 11667893 , 11409972 , 151194 , 11234052 , 10074640 , 3038522 , 3973 , 24779724 , 15983966 , 10427712 , 3025986 , 11485656 , 447077 , 2396 , 16122633 , 11427553 , 6419766 , 11314340 , 3542 , 10127622 , 448008 , 11712649 , 76098 , 153999 , 11608401 , 17755052 , 176870 , 160355 , 176167 , 10113978 , 208908 , 447966 , 11364421 , 156414 , 11338033 , 5005498 , 6918454 , 16722836]

1485|Q15139(47) [3540 , 176870 , 11364421 , 10172943 , 6419766 , 11234052 , 447077 , 153999 , 3025986 , 160355 , 24779724 , 76098 , 11712649 , 10074640 , 11338033 , 5005498 , 24889392 , 10113978 , 2396 , 17755052 , 208908 , 11427553 , 10127622 , 16122633 , 176167 , 6918454 , 16722836 , 3973 , 447966 , 11656518 , 11667893 , 3542 , 3038522 , 9549303 , 11314340 , 11409972 , 156414 , 11213558 , 151194 , 11485656 , 448008 , 3078519 , 2856 , 10427712 , 15983966 , 11608401]

1486|P41240(37) [1694 , 11314340 , 6419766 , 5005498 , 447077 , 9549303 , 3973 , 176870 , 10074640 , 15983966 , 156414 , 16722836 , 24889392 , 10302451 , 3078519 , 153999 , 151194 , 10427712 , 2396 , 6918454 , 3542 , 11364421 , 11656518 , 17755052 , 3025986 , 10113978 , 11409972 , 11712649 , 176167 , 3038522 , 3540 , 448008 , 160355 , 10172943 , 11234052 , 208908 , 24779724 , 447966 , 11427553 , 2856 , 10127622 , 11973736 , 11485656 , 11667893 , 11213558 , 11338033]

1487|P42684(19) [176167 , 11314340 , 644241 , 2856 , 11427553 , 10113978 , 10427712 , 11409972 , 153999 , 448008 , 11712649 , 447966 , 447077 , 3038522 , 6419766 , 5328940 , 156414 , 24779724 , 2396 , 10074640 , 15983966 , 16122633 , 160355 , 16722836 , 6918454 , 11608401 , 11364421 , 3062316 , 5005498 , 11656518 , 3025986 , 11213558 , 10127622 , 11338033 , 17755052 , 176870 , 76098 , 9549303 , 208908 , 11667893 , 24889392 , 11234052 , 5291 , 151194 , 10302451 , 11485656 , 3973]

1488|P22455(112) [3025986 , 160355 , 6918454 , 9809715 , 11656518 , 11314340 , 156414 , 17755052 , 10074640 , 10113978 , 2856 , 176870 , 53235510 , 2396 , 153999 , 447077 , 10427712 , 3973 , 51039095 , 208908 , 448008 , 3038522 , 24889392 , 11427553 , 15983966 , 11364421 , 16722836 , 151194 , 11213558 , 11667893 , 11338033 , 5005498 , 11234052 , 6419766 , 11409972 , 11485656 , 11608401 , 24826799 , 24779724 , 76098 , 9549303 , 57379345 , 16122633 , 176167 , 10127622 , 11712649 , 447966]

1489|Q13188(15) [176167 , 11213558 , 10427712 , 151194 , 11667893 , 11234052 , 2856 , 10074640 , 11485656 , 17755052 , 10113978 , 3038522 , 2396 , 11427553 , 16122633 , 5005498 , 176870 , 11364421 , 11314340 , 10172943 , 3078519 , 156414 , 11409972 , 9549303 , 3540 , 24779724 , 10127622 , 24889392 , 3973 , 76098 , 153999 , 11712649 , 448008 , 208908 , 11608401 , 15983966 , 160355 , 11338033 , 11751922 , 3025986 , 3542 , 11656518 , 6419766 , 447077 , 447966 , 6918454 , 16722836]

1490|P04049(156) [151194 , 156414 , 9549303 , 11234052 , 11364421 , 11338033 , 176167 , 11409972 , 11667893 , 10127622 , 25161177 , 24779724 , 447077 , 15983966 , 5005498 , 11608401 , 160355 , 2856 , 2396 , 11213558 , 448008 , 208908 , 3038522 , 10427712 , 153999 , 76098 , 11167602 , 11314340 , 176870 , 16122633 , 11712649 , 6419766 , 10113978 , 11656518 , 3973 , 17755052 , 11427553 , 6918454 , 447721 , 42611257 , 10074640 , 16722836 , 44462760 , 3025986 , 11485656 , 24889392 , 447966]

1491|P51812(74) [11409972 , 6419766 , 11427553 , 11314340 , 11712649 , 11667893 , 10113978 , 10427712 , 3542 , 3025986 , 11213558 , 17755052 , 11608401 , 447077 , 3973 , 3540 , 25227436 , 11234052 , 76098 , 5005498 , 160355 , 6918454 , 24889392 , 11656518 , 10127622 , 156414 , 11338033 , 15983966 , 447966 , 448008 , 2396 , 176167 , 10074640 , 3078519 , 2856 , 16722836 , 208908 , 151194 , 16122633 , 24779724 , 3038522 , 9549303 , 11364421 , 153999 , 11485656 , 10172943 , 176870]

1492|P49137(22) [11656518 , 2396 , 11314340 , 11234052 , 76098 , 153999 , 11427553 , 11338033 , 10127622 , 11712649 , 24889392 , 16122633 , 447077 , 3038522 , 10172943 , 6419766 , 16058637 , 3973 , 17755052 , 448008 , 156414 , 176870 , 10113978 , 11364421 , 3540 , 11485656 , 3542 , 11409972 , 160355 , 447966 , 208908 , 9549303 , 16722836 , 11667893 , 11213558 , 2856 , 11608401 , 3025986 , 151194 , 15983966 , 176167 , 24779724 , 10427712 , 5005498 , 10074640 , 3078519 , 6918454]

1493|P46098(19) [108000 , 11151363 , 11961293 , 2159 , 216236 , 3052778 , 5074 , 5284583 , 44623946 , 119828 , 71768094 , 115237 , 656665 , 446220 , 3510 , 119570 , 6337614 , 3033818 , 5011 , 6918107 , 888 , 9966051 , 46196517 , 1615 , 39186 , 9794392 , 21138 , 42601552 , 4168 , 187 , 65997 , 71785 , 1355 , 25070031 , 154104 , 2264 , 2170 , 6000 , 16005981 , 3559 , 4914 , 10624 , 24795080 , 4595 , 2099 , 5284566 , 3389]

1494|Q8IW41(2) [447966 , 208908 , 11314340 , 16722836 , 3542 , 1694 , 24889392 , 3973 , 11608401 , 5005498 , 24779724 , 6419766 , 76098 , 11409972 , 2396 , 151194 , 11485656 , 16122633 , 3025986 , 11667893 , 11712649 , 160355 , 10172943 , 3038522 , 176167 , 2856 , 176870 , 447077 , 17755052 , 10427712 , 448008 , 15983966 , 10113978 , 11427553 , 6918454 , 10127622 , 10074640 , 11656518 , 3540 , 11364421 , 156414 , 11338033 , 153999 , 11234052 , 3078519 , 11213558 , 9549303]

1495|Q15418(21) [24779724 , 11364421 , 10172943 , 2856 , 11485656 , 11667893 , 11409972 , 3540 , 5005498 , 176167 , 11234052 , 6419766 , 16722836 , 3542 , 11338033 , 76098 , 3025986 , 9549303 , 11213558 , 3078519 , 6918454 , 17755052 , 11608401 , 447966 , 10127622 , 11656518 , 153999 , 16122633 , 447077 , 208908 , 11712649 , 160355 , 3038522 , 10113978 , 11314340 , 2396 , 3973 , 11427553 , 10074640 , 156414 , 448008 , 1694 , 24889392 , 151194 , 176870 , 10427712 , 15983966]

1496|S4R381(0) [208908 , 447966 , 11485656 , 153999 , 11338033 , 151194 , 57379345 , 5005498 , 2856 , 176167 , 6918454 , 1633 , 6419766 , 16122633 , 10113978 , 24889392 , 447077 , 24826799 , 10074640 , 448008 , 10427712 , 16722836 , 11409972 , 447622 , 15983966 , 11364421 , 24779724 , 11712649 , 11667893 , 3025986 , 11427553 , 11656518 , 11167602 , 11234052 , 11213558 , 76098 , 11314340 , 11608401 , 3973 , 160355 , 10127622 , 2396 , 9549303 , 176870 , 156414 , 3038522 , 17755052]

1497|Q02750(136) [11712649 , 3025986 , 3973 , 10427712 , 11234052 , 447077 , 10172943 , 6419766 , 11364421 , 24889392 , 10113978 , 2856 , 447966 , 3542 , 176167 , 448008 , 3038522 , 9549303 , 176870 , 11485656 , 11409972 , 11338033 , 10074640 , 1694 , 24779724 , 151194 , 11427553 , 16722836 , 3540 , 10127622 , 11314340 , 11656518 , 208908 , 6918454 , 15983966 , 11707110 , 11213558 , 5005498 , 153999 , 2396 , 156414 , 11667893 , 160355 , 17755052 , 10172827 , 3078519 , 25227436 , 16222096]

1498|O14920(65) [11409972 , 11485656 , 3025986 , 9549303 , 153999 , 11667893 , 17755052 , 11213558 , 11656518 , 11608401 , 76098 , 6918454 , 10113978 , 5005498 , 447077 , 2856 , 11314340 , 10127622 , 6419766 , 11234052 , 24779724 , 176870 , 11338033 , 156414 , 11552706 , 10427712 , 2396 , 160355 , 448008 , 3540 , 24889392 , 208908 , 11364421 , 447966 , 3038522 , 16122633 , 151194 , 15983966 , 10172943 , 3542 , 10074640 , 11712649 , 176167 , 11442891 , 16722836 , 3078519 , 11427553 , 3973]

1499|P45983(168) [11712649 , 10172943 , 10127622 , 3078519 , 16722836 , 10427712 , 24889392 , 11656518 , 2396 , 24779724 , 11364421 , 11667893 , 10113978 , 10275001 , 11409972 , 11485656 , 160355 , 151194 , 176870 , 2856 , 16058637 , 153999 , 156414 , 6918454 , 11234052 , 11314340 , 6419766 , 10296883 , 10267580 , 11427553 , 1694 , 9549303 , 3025986 , 3540 , 11338033 , 447077 , 15983966 , 447966 , 3038522 , 5005498 , 10430360 , 176167 , 208908 , 3973 , 17755052 , 11213558 , 10074640 , 3542]

1500|P08069(215) [208908 , 153999 , 447966 , 6918454 , 72435 , 51039095 , 11656518 , 5005498 , 11485656 , 10074640 , 11213558 , 10296883 , 447077 , 57379345 , 11427553 , 10113978 , 10127622 , 11409972 , 10427712 , 448008 , 16122633 , 11338033 , 3025986 , 24889392 , 3973 , 11667893 , 24785538 , 11608401 , 76098 , 16722836 , 160355 , 6419766 , 176167 , 11364421 , 156414 , 15983966 , 176870 , 2856 , 11640390 , 11234052 , 2396 , 9549303 , 3038522 , 17755052 , 24779724 , 11314340 , 151194 , 11712649]

1501|Q499Y8(0) [6918454 , 151194 , 10113978 , 11656518 , 3078519 , 160355 , 11314340 , 447077 , 24779724 , 3038522 , 9549303 , 11338033 , 15983966 , 6419766 , 153999 , 11712649 , 11667893 , 3540 , 2856 , 5005498 , 176870 , 44224261 , 10172943 , 2396 , 447966 , 11213558 , 3025986 , 10427712 , 176167 , 11552706 , 16750106 , 208908 , 3973 , 24889392 , 16722836 , 156414 , 11409972 , 11427553 , 5171 , 10127622 , 3542 , 10074640 , 17755052 , 11234052 , 11485656 , 16129582 , 10296883 , 11364421]

1502|P31751(81) [9549303 , 25227436 , 2396 , 11667893 , 6419766 , 10113978 , 11485656 , 448008 , 11427553 , 11364421 , 11409972 , 176167 , 16122634 , 11213558 , 16122633 , 11234052 , 3973 , 10427712 , 3540 , 11608401 , 3025986 , 10127622 , 11175137 , 11338033 , 208908 , 10172943 , 447966 , 151194 , 11314340 , 447077 , 24889392 , 5005498 , 176870 , 156414 , 16722836 , 6918454 , 11656518 , 24779724 , 17755052 , 2856 , 153999 , 10074640 , 160355 , 11712649 , 3078519 , 15983966 , 3542 , 3038522]

1503|O15264(8) [11338033 , 3038522 , 3973 , 176167 , 10267580 , 151194 , 10172943 , 5005498 , 17755052 , 10113978 , 24779724 , 10427712 , 15983966 , 3540 , 16722836 , 11364421 , 156414 , 2856 , 11656518 , 11409972 , 16122633 , 11608401 , 3078519 , 160355 , 24889392 , 11234052 , 3025986 , 447966 , 208908 , 11712649 , 153999 , 10074640 , 448008 , 3542 , 11667893 , 447077 , 11485656 , 2396 , 76098 , 10127622 , 176870 , 6918454 , 11314340 , 9549303 , 6419766 , 1694 , 11427553 , 11213558]

1504|O75582(11) [25227436 , 24889392 , 151194 , 9549303 , 10127622 , 208908 , 3078519 , 3540 , 11485656 , 76098 , 17755052 , 11608401 , 11234052 , 176870 , 176167 , 3973 , 11213558 , 447077 , 11667893 , 11427553 , 11364421 , 11409972 , 3542 , 10427712 , 1694 , 448008 , 10113978 , 3038522 , 10172943 , 10267580 , 6419766 , 153999 , 24779724 , 11314340 , 2856 , 6918454 , 2396 , 11656518 , 447966 , 11712649 , 15983966 , 160355 , 11338033 , 10074640 , 5005498 , 3025986 , 16122633 , 16722836 , 156414]

1505|Q03164(107) [4343310 , 5381 , 3108 , 2234553 , 3842920 , 8026 , 50942 , 3246767 , 13986 , 5475 , 6470206 , 30717 , 2315667 , 2739563 , 24792601 , 12938 , 10718 , 3236558 , 361939 , 2090 , 19529 , 1561922 , 3295 , 13791 , 4380 , 3603333 , 722121 , 2179 , 2075 , 2200 , 4097 , 7475369 , 3377088 , 655916 , 327045 , 2799 , 11296583 , 22430877 , 547914 , 24817194 , 7191 , 42725 , 94280 , 24792593 , 14369 , 2832895 , 3117 , 235434 , 3138364]

1506|Q03181(82) [213013 , 3034285 , 10168 , 11236126 , 5289501 , 11483970 , 10467 , 11293 , 7329 , 10868 , 2950 , 10229498 , 2265 , 114924 , 6603901 , 21805 , 1046 , 25644 , 11604 , 8041 , 444795 , 4197 , 18056 , 115157 , 12589 , 5921 , 3672772 , 204109 , 9803963 , 3503 , 1123 , 21307 , 28803 , 16362 , 8095 , 3969 , 8467 , 2750 , 62485 , 41684 , 11742 , 11852 , 206044 , 11395145 , 11711595 , 19996 , 16734800 , 16316 , 39042]

1507|Q14DU5(0) [3078519 , 447966 , 447077 , 208908 , 11427553 , 10127622 , 3025986 , 6419766 , 11667893 , 156414 , 176870 , 10113978 , 11314340 , 1694 , 2396 , 151194 , 448008 , 3540 , 3542 , 11712649 , 25227436 , 3973 , 16122633 , 11656518 , 11608401 , 11409972 , 176167 , 11213558 , 9549303 , 24889392 , 15983966 , 11364421 , 10074640 , 57379345 , 2856 , 160355 , 10172943 , 17755052 , 11234052 , 76098 , 11338033 , 3038522 , 153999 , 24779724 , 11485656 , 6918454 , 10427712 , 5005498 , 16722836 , 10296883]

1508|P06213(138) [11314340 , 11712649 , 3025986 , 10113978 , 15983966 , 151194 , 16722836 , 11364421 , 11442891 , 11338033 , 24871491 , 24779724 , 10275001 , 447966 , 448008 , 156414 , 76098 , 11667893 , 10127622 , 3033769 , 3973 , 447077 , 10296883 , 6419766 , 57379345 , 11608401 , 176167 , 2396 , 11656518 , 11213558 , 11427553 , 6918454 , 11485656 , 11234052 , 10427712 , 9549303 , 2856 , 10074640 , 17755052 , 11409972 , 24889392 , 3038522 , 160355 , 5005498 , 176870 , 16137271 , 208908 , 153999 , 16122633 , 11640390]

1509|O96017(110) [24779724 , 11751922 , 3038522 , 208908 , 2396 , 3078519 , 160355 , 156414 , 6918454 , 11427553 , 11485656 , 24889392 , 9549303 , 11213558 , 10427712 , 76098 , 176167 , 6419766 , 11712649 , 3542 , 11152667 , 11314340 , 11234052 , 10275001 , 11610113 , 11338033 , 447966 , 3540 , 15983966 , 153999 , 448008 , 3973 , 151194 , 3025986 , 447077 , 176870 , 10113978 , 16722836 , 11409972 , 2856 , 16122633 , 11364421 , 11656518 , 10074640 , 5005498 , 10172943 , 17755052 , 11608401 , 11667893 , 10127622]

1510|Q15759(8) [22049997 , 9871074 , 447966 , 3025986 , 9549303 , 153999 , 11712649 , 11409972 , 10341154 , 176870 , 11667893 , 11234052 , 2396 , 15983966 , 10427712 , 24779724 , 6918454 , 3542 , 10113978 , 448008 , 10074640 , 3540 , 17755052 , 10172943 , 6419766 , 3038522 , 11338033 , 11656518 , 208908 , 10267580 , 11213558 , 11427553 , 447077 , 3973 , 160355 , 2856 , 16722836 , 1694 , 11314340 , 24889392 , 11167602 , 11364421 , 5005498 , 11485656 , 10127622 , 156414 , 176167 , 151194 , 3078519 , 10409068]

1511|Q9UQB9(13) [10113978 , 24779724 , 10172943 , 17755052 , 160355 , 10074640 , 156414 , 176167 , 11364421 , 2396 , 15983966 , 11442891 , 11667893 , 11338033 , 16722836 , 208908 , 151194 , 6419766 , 9549303 , 153999 , 176870 , 24856041 , 11608401 , 3038522 , 11485656 , 447077 , 11234052 , 11314340 , 11656518 , 11712649 , 6918454 , 11213558 , 2856 , 5005498 , 447966 , 46885626 , 11427553 , 24995524 , 3025986 , 24889392 , 11409972 , 3078519 , 10127622 , 3540 , 448008 , 16122633 , 3542 , 10427712 , 76098 , 3973]

1512|P52333(77) [160355 , 6918454 , 16722836 , 11712649 , 15991573 , 5330286 , 151194 , 9926791 , 57379345 , 17755052 , 11213558 , 11409972 , 176167 , 10296883 , 16659841 , 10127622 , 3038522 , 153999 , 11485656 , 2396 , 448008 , 2856 , 11667893 , 11234052 , 176870 , 10427712 , 15983966 , 46866319 , 10113978 , 11656518 , 45375955 , 11314340 , 25062766 , 6419766 , 11364421 , 156414 , 447077 , 208908 , 10074640 , 447966 , 46216796 , 11977753 , 11338033 , 5005498 , 11427553 , 24779724 , 3973 , 24889392 , 3025986 , 9549303]

1513|P51151(5) [3455 , 1552036 , 17113 , 3114023 , 10219 , 2576 , 162834 , 3095276 , 2291046 , 16187479 , 1568843 , 3542 , 1878823 , 16490 , 3240818 , 3746037 , 3127493 , 2197 , 2768954 , 160355 , 5289501 , 1608140 , 327045 , 22430825 , 1599306 , 65758 , 2812 , 5405 , 42725 , 3503 , 41684 , 1580955 , 265580 , 19646 , 19910 , 24817194 , 1720828 , 1238 , 187 , 2161 , 1811924 , 680935 , 5074 , 70846 , 4380 , 3885 , 107985 , 1432578 , 16362 , 1694 , 4493]

1514|P48736(286) [15983966 , 25033539 , 24889392 , 9849735 , 6852165 , 25254071 , 176870 , 16654980 , 3025986 , 50905713 , 11485656 , 11234052 , 3973 , 11338033 , 16736978 , 11213558 , 11977753 , 447077 , 44516953 , 3038522 , 208908 , 11409972 , 176167 , 151171 , 10113978 , 10074640 , 153999 , 11647372 , 24779724 , 11667893 , 11427553 , 17755052 , 10427712 , 44137675 , 10296883 , 11314340 , 11625818 , 11712649 , 49784945 , 11364421 , 56649450 , 51001932 , 54575456 , 24989044 , 156414 , 151194 , 16722836 , 6918454 , 11656518 , 25167777 , 10127622]

1515|P11309(66) [151194 , 11364421 , 153999 , 11667893 , 24748573 , 448008 , 10172943 , 11338033 , 3038522 , 10427712 , 11234052 , 11712649 , 9549303 , 176167 , 3540 , 16722836 , 156414 , 76098 , 17755052 , 11656518 , 2396 , 10127622 , 208908 , 11608401 , 5005498 , 11314340 , 447966 , 6918454 , 24889392 , 57899889 , 160355 , 11213558 , 6419766 , 3025986 , 176870 , 11485656 , 24795070 , 2856 , 611002 , 16122633 , 3973 , 24779724 , 15983966 , 10074640 , 3542 , 10296883 , 11409972 , 447077 , 11427553 , 10113978 , 3078519]

1516|P35368(14) [3822 , 2170 , 115237 , 2435 , 5073 , 3157 , 10624 , 1615 , 33625 , 2159 , 4747 , 5265 , 60602 , 119828 , 5816 , 6082 , 2818 , 17747460 , 10836 , 1355 , 439260 , 164089 , 3677 , 208898 , 4184 , 5074 , 2092 , 60820 , 30487 , 3559 , 72106 , 119570 , 6041 , 129211 , 148842 , 4636 , 60809 , 5826 , 5268 , 5775 , 11597698 , 5401 , 5312125 , 13542 , 2368 , 4893 , 38521 , 3389 , 6077 , 216249 , 2913]

1517|P53350(99) [11338033 , 24889392 , 3973 , 3038522 , 2856 , 3542 , 176167 , 448008 , 17755052 , 11442891 , 176870 , 16722836 , 53357478 , 11213558 , 11409972 , 151194 , 11314340 , 2396 , 3078519 , 447966 , 10427712 , 208908 , 10127622 , 16058637 , 11552706 , 10461508 , 447077 , 160355 , 3540 , 10074640 , 156414 , 153999 , 11712649 , 11364421 , 15983966 , 24779724 , 10172943 , 10113978 , 11427553 , 6918736 , 11485656 , 9549303 , 3025986 , 11234052 , 131682 , 11667893 , 11656518 , 6419766 , 35595 , 6918454 , 5005498]

1518|P34969(18) [71360 , 2159 , 11430856 , 10531 , 3559 , 11954293 , 2913 , 2726 , 5074 , 4847 , 1615 , 4184 , 4585 , 197033 , 11961293 , 11292933 , 9966051 , 2818 , 213046 , 1150 , 119828 , 60809 , 3822 , 68848 , 9805719 , 2170 , 44623946 , 119570 , 23897 , 16362 , 4748 , 49381 , 1355 , 19241 , 27400 , 10212 , 3372 , 62865 , 60854 , 5265 , 28693 , 71768094 , 3389 , 8969 , 5073 , 5452 , 10624 , 115237 , 5736 , 4106 , 42601552 , 5358]

1519|P07949(222) [447077 , 11656518 , 15983966 , 11667893 , 3081361 , 57379345 , 25102847 , 24826799 , 11314340 , 11427553 , 3038522 , 10113978 , 6419766 , 17755052 , 10296883 , 11485656 , 208908 , 25031915 , 216239 , 11234052 , 160355 , 156414 , 176167 , 5329102 , 448008 , 11442891 , 3025986 , 11213558 , 11338033 , 10427712 , 11167602 , 447966 , 11364421 , 9549303 , 3973 , 2396 , 176870 , 24779724 , 11712649 , 11751922 , 153999 , 151194 , 5005498 , 11282283 , 24889392 , 10127622 , 11409972 , 24767976 , 10074640 , 16722836 , 2856 , 6918454]

1520|P17612(37) [3542 , 11667893 , 11712649 , 11656518 , 11409972 , 176167 , 6918454 , 24889392 , 11427553 , 16122634 , 160355 , 17755052 , 12412578 , 208908 , 448008 , 151194 , 3973 , 11234052 , 11314340 , 10113978 , 5005498 , 2396 , 10050566 , 3025986 , 11338033 , 10427712 , 9549303 , 11364421 , 10074640 , 10172943 , 3078519 , 24779724 , 11840906 , 16122635 , 11608401 , 10127622 , 16122633 , 3540 , 15983966 , 11213558 , 447966 , 447077 , 11175137 , 16722836 , 6419766 , 153999 , 156414 , 449241 , 3038522 , 176870 , 2856 , 11485656]

1521|P16234(175) [57379345 , 11167602 , 3973 , 448008 , 11608401 , 11409972 , 24826799 , 10074640 , 2396 , 447077 , 9809715 , 153999 , 76098 , 6918454 , 160355 , 151194 , 16722836 , 25031915 , 11667893 , 17755052 , 9933475 , 3025986 , 9549303 , 10366136 , 16122633 , 447966 , 2856 , 15983966 , 5005498 , 11338033 , 24779724 , 11751922 , 176870 , 176167 , 24889392 , 10427712 , 11213558 , 11485656 , 156414 , 11656518 , 10302451 , 10113978 , 208908 , 11364421 , 24767976 , 6419766 , 10127622 , 11234052 , 11427553 , 11314340 , 11282283 , 3038522 , 11712649]

1522|P35916(99) [176167 , 9809715 , 156414 , 11167602 , 3038522 , 11973736 , 176870 , 11409972 , 10296883 , 10127622 , 3973 , 2396 , 6419766 , 151194 , 5005498 , 24889392 , 11442891 , 11485656 , 10074640 , 11364421 , 10275001 , 448008 , 9823820 , 10113978 , 9911830 , 17755052 , 10427712 , 11234052 , 45142457 , 11712649 , 160355 , 153999 , 16722836 , 208908 , 447077 , 42642645 , 10302451 , 6918454 , 9549295 , 11656518 , 2856 , 3025986 , 447966 , 9933475 , 15983966 , 11314340 , 11667893 , 11338033 , 11427553 , 9549303 , 11213558 , 24779724 , 24767976]

1523|P04062(105) [3237705 , 3455 , 24144 , 61574 , 6603901 , 228526 , 3559 , 3731631 , 54675783 , 2426546 , 11293 , 2161 , 1123 , 2740698 , 2932047 , 2391 , 2330 , 16725204 , 3760 , 10382715 , 12454 , 5405 , 2132 , 244136 , 2540 , 1989 , 47472 , 10133 , 3926 , 10206 , 1548942 , 2179 , 10235 , 2092 , 3238739 , 5723 , 11790 , 2318 , 2396 , 2090 , 19646 , 24791741 , 3478 , 2216 , 9820526 , 3316 , 12028 , 1580955 , 2938038 , 16231 , 2562 , 8041 , 2247]

1524|Q13526(75) [646716 , 13791 , 2834684 , 3243710 , 24816636 , 3108 , 71157 , 3245402 , 6603842 , 3503 , 19646 , 114924 , 1082702 , 31475 , 16015629 , 10621 , 11852 , 1870753 , 3885 , 6603901 , 3435 , 6301 , 11954283 , 3698 , 5392 , 2090 , 2866904 , 4380 , 4814 , 32681 , 1369 , 2768975 , 11293 , 327045 , 5289501 , 4197 , 104838 , 327044 , 185915 , 197033 , 44142959 , 55918 , 3238739 , 5722 , 2490338 , 90206 , 10612 , 10168 , 1561922 , 104762 , 4343310 , 2927638 , 3238413 , 288875]

1525|O60674(214) [208908 , 24889392 , 10296883 , 11234052 , 11338033 , 46216796 , 3038522 , 160355 , 5005498 , 447966 , 11285002 , 11492186 , 3025986 , 16659841 , 448008 , 11213558 , 10427712 , 447077 , 156414 , 17755052 , 11409972 , 9549303 , 11656518 , 10127622 , 11427553 , 10074640 , 11485656 , 10113978 , 57379345 , 6918454 , 3973 , 24779724 , 25062766 , 6419766 , 176167 , 11314340 , 153999 , 46866319 , 11667893 , 11712649 , 46213929 , 2856 , 15983966 , 151194 , 24963047 , 57990770 , 2396 , 44205240 , 176870 , 177336 , 45375955 , 11364421 , 25126798 , 16722836]

1526|P28221(14) [77993 , 66004 , 1150 , 5074 , 28864 , 4636 , 60857 , 8969 , 16362 , 5002 , 71351 , 3822 , 31101 , 5736 , 4106 , 219050 , 1355 , 5073 , 2818 , 197706 , 123606 , 60795 , 2913 , 5265 , 182137 , 10257 , 119828 , 1615 , 115237 , 2159 , 60854 , 5358 , 4830 , 60809 , 77992 , 47811 , 54746 , 5078 , 11961293 , 6089 , 4585 , 3389 , 3559 , 28693 , 11292933 , 8223 , 119570 , 9805719 , 443951 , 21138 , 3396 , 10531 , 10624 , 4440 , 60149]

1527|P10721(263) [11667893 , 447966 , 10302451 , 24889392 , 3038522 , 5291 , 24826799 , 11427553 , 11485656 , 2396 , 3025986 , 2856 , 11338033 , 11234052 , 10127622 , 448008 , 9868037 , 10366136 , 153999 , 156414 , 24779724 , 17755052 , 11656518 , 10074640 , 11364421 , 16722836 , 644241 , 10427712 , 9933475 , 11409972 , 15983966 , 25102847 , 11282283 , 208908 , 5005498 , 11314340 , 176167 , 6419766 , 160355 , 42642645 , 10296883 , 176870 , 25031915 , 11213558 , 447077 , 5329102 , 11712649 , 10275001 , 10113978 , 11167602 , 9549303 , 57379345 , 151194 , 6918454 , 11442891 , 3973]

1528|Q6W5P4(27) [2082 , 3298363 , 3333 , 1392 , 2754 , 208820 , 3311 , 41684 , 3138330 , 1811924 , 16682071 , 361655 , 3117 , 3671 , 11683 , 3455 , 2170 , 1066 , 16362 , 1720828 , 1967 , 10382715 , 3503 , 5405 , 6603842 , 1234 , 2303880 , 4362 , 3221232 , 50248 , 719632 , 3108 , 5289501 , 2179 , 11954283 , 4493 , 3885 , 1547484 , 1568843 , 53708 , 647116 , 2740698 , 4122 , 824727 , 5074 , 5335 , 2812 , 1878823 , 936430 , 2832895 , 10219 , 2327 , 1893 , 3746037 , 11289 , 76915]

1529|P37840(72) [547914 , 3746037 , 11296583 , 3138370 , 361655 , 7329 , 344675 , 2812 , 16268999 , 1694 , 16745942 , 441383 , 3333 , 722121 , 11852 , 3138330 , 2799 , 9953 , 26695 , 12449 , 3760 , 2333 , 114924 , 19910 , 2090 , 3162006 , 6603842 , 4030278 , 16574 , 50248 , 1878823 , 16129778 , 3117 , 37175 , 1720828 , 11779629 , 3286 , 3138375 , 13752 , 249 , 3885 , 5917 , 2359 , 1568843 , 192197 , 3244425 , 3242068 , 10133 , 1392 , 310612 , 3138364 , 19646 , 6603901 , 11293 , 3503 , 3406]

1530|P11362(191) [10302451 , 2856 , 6918454 , 15983966 , 447966 , 208908 , 160355 , 1633 , 5005498 , 24779724 , 53235510 , 11234052 , 24889392 , 76098 , 151194 , 16722836 , 10113978 , 156414 , 9549303 , 11608401 , 3025986 , 11314340 , 10127622 , 5330286 , 11213558 , 11167602 , 153999 , 447077 , 11364421 , 11610113 , 51039095 , 9809715 , 11485656 , 24826799 , 17755052 , 11712649 , 11751922 , 3038522 , 11409972 , 6419766 , 176167 , 10275001 , 3973 , 11667893 , 448008 , 11442891 , 2396 , 10074640 , 11338033 , 176870 , 25031915 , 5289418 , 5329099 , 10427712 , 11656518 , 11427553 , 16122633]

1531|O14757(82) [3038522 , 11656518 , 16750408 , 3973 , 16722836 , 6918454 , 10172943 , 3078519 , 11442891 , 11667893 , 24889392 , 447966 , 739358 , 1694 , 11712649 , 11409972 , 10127622 , 3540 , 10113978 , 3542 , 11485656 , 17755052 , 10156987 , 176167 , 6419766 , 16058637 , 5005498 , 46937179 , 9549303 , 10275001 , 208908 , 11338033 , 160355 , 11427553 , 2856 , 11234052 , 76098 , 72271 , 3025986 , 153999 , 176870 , 447077 , 2396 , 4566 , 11364421 , 11608401 , 16122633 , 448008 , 11314340 , 10427712 , 15983966 , 151194 , 24779724 , 11152667 , 156414 , 11213558 , 10074640]

1532|P31749(379) [447966 , 156414 , 2396 , 151194 , 11667893 , 10074640 , 148177 , 24779724 , 3542 , 11234052 , 25033539 , 15983966 , 16058637 , 5005498 , 11656518 , 6918454 , 17755052 , 11608401 , 16725726 , 176167 , 11552706 , 1694 , 208908 , 11442891 , 3540 , 11213558 , 11338033 , 24889392 , 3078519 , 2856 , 11427553 , 10113978 , 448008 , 10427712 , 176870 , 10172943 , 10296883 , 6419766 , 3973 , 3038522 , 11485656 , 447077 , 16122633 , 160355 , 11364421 , 16722836 , 11314340 , 11712649 , 1392 , 24748573 , 153999 , 10127622 , 44516953 , 9549303 , 3025986 , 25227436 , 11409972 , 76098]

1533|P36888(79) [11234052 , 160355 , 76098 , 11213558 , 448008 , 16722836 , 5329102 , 11712649 , 10275001 , 42642645 , 10113978 , 17755052 , 11656518 , 447966 , 151194 , 24779724 , 10427712 , 15983966 , 10074640 , 11282283 , 3973 , 24748573 , 11349170 , 176167 , 3038522 , 5005498 , 2396 , 176870 , 11314340 , 447077 , 57379345 , 11485656 , 6420138 , 9549303 , 3025986 , 11608401 , 46216796 , 2856 , 10366136 , 10127622 , 6918454 , 11442891 , 11285002 , 5330286 , 11751922 , 156414 , 24889392 , 11427553 , 153999 , 11667893 , 208908 , 24826799 , 9933475 , 11409972 , 16122633 , 11364421 , 6419766 , 11338033]

1534|O14965(103) [2856 , 24995524 , 11656518 , 11338033 , 10127622 , 11610113 , 11409972 , 447077 , 447961 , 9549303 , 24779724 , 11442891 , 156414 , 3038522 , 11751922 , 24889392 , 176167 , 153999 , 11485656 , 24748204 , 11213558 , 16722836 , 151194 , 160355 , 208908 , 6419766 , 11667893 , 5329099 , 17755052 , 46885626 , 5494449 , 6918454 , 46207586 , 11364421 , 24893974 , 11496629 , 24771867 , 11427553 , 10113978 , 16122633 , 176870 , 3025986 , 11712649 , 11234052 , 3973 , 10074640 , 76098 , 448008 , 5005498 , 447966 , 2396 , 15983966 , 11497983 , 11608401 , 24856041 , 10427712 , 6420138 , 11314340]

1535|P14867(30) [3016 , 10237 , 33746 , 37632 , 3261 , 2441 , 4912 , 3608 , 2164 , 3033621 , 5281077 , 3369 , 2893 , 5361323 , 3448 , 4266 , 5556 , 6450813 , 3380 , 9908684 , 65914 , 4162 , 3032285 , 32051 , 2576 , 3003157 , 10531 , 6473 , 2294 , 4999 , 21777 , 104781 , 4737 , 5193 , 8275 , 10518 , 4116 , 4064 , 2118 , 5732 , 4506 , 10133 , 3373 , 4890 , 107926 , 4909 , 2170 , 216456 , 31304 , 2802 , 2479 , 2811 , 2481 , 9034 , 702 , 8271 , 4099 , 31640 , 2789]

1536|P08581(191) [448008 , 16122633 , 6419766 , 3038522 , 17755052 , 25102847 , 24779724 , 24864821 , 6918454 , 9549303 , 11656518 , 11626560 , 2396 , 15983966 , 24889392 , 11667893 , 5005498 , 24794418 , 160355 , 10074640 , 153999 , 11282283 , 24901704 , 25145656 , 11314340 , 25113181 , 16722836 , 25171648 , 49806720 , 11442891 , 2856 , 10113978 , 151194 , 11338033 , 11712649 , 42642645 , 3025986 , 17748441 , 11494412 , 156414 , 17754438 , 3973 , 447077 , 176167 , 10427712 , 11409972 , 447966 , 208908 , 11427553 , 45142457 , 10127622 , 11364421 , 176870 , 11485656 , 11234052 , 76098 , 11608401 , 57379345 , 11213558 , 10296883]

1537|Q96GD4(60) [447077 , 11712649 , 6918454 , 11314340 , 24856041 , 11485656 , 156414 , 3025986 , 11497983 , 17755052 , 10127622 , 3078519 , 10621 , 447966 , 6419766 , 16122633 , 5494449 , 2396 , 16722836 , 151194 , 9549303 , 24889392 , 11442891 , 11667893 , 24995524 , 3973 , 447961 , 3038522 , 15983966 , 160355 , 11427553 , 10172943 , 11608401 , 448008 , 76098 , 10302451 , 11409972 , 11364421 , 16124208 , 10074640 , 11496629 , 10427712 , 176167 , 3542 , 11656518 , 24748204 , 153999 , 5005498 , 6420138 , 11213558 , 208908 , 46885626 , 176870 , 5329099 , 10113978 , 2856 , 3540 , 46207586 , 11338033 , 11234052 , 24779724]

1538|P08183(360) [33746 , 107715 , 10258 , 123979 , 2794 , 6063342 , 16129681 , 2789 , 2997 , 2247 , 1001 , 11954293 , 182137 , 2540 , 2179 , 108000 , 5405 , 3333 , 2812 , 11683 , 123409 , 148201 , 25019940 , 65889 , 11147 , 10218498 , 11617 , 3033877 , 216454 , 2355 , 146396 , 119373 , 2725 , 175540 , 213039 , 3025986 , 10531 , 71144 , 2082 , 3005573 , 10219 , 3108 , 11765960 , 3559 , 114948 , 1234 , 124087 , 3033767 , 176870 , 154048 , 4064 , 16362 , 1715 , 2473 , 4506 , 3151 , 107807 , 4030 , 2893 , 21138 , 3404 , 104850]

1539|P10253(78) [22530 , 3503 , 3241177 , 2161 , 652757 , 3222852 , 5225473 , 228526 , 131411 , 2092 , 104762 , 4592 , 3244566 , 2247 , 2291046 , 28446 , 657977 , 2162118 , 36303 , 3240818 , 657677 , 60734 , 2369 , 5405 , 3239385 , 12132 , 2132 , 2894446 , 1547484 , 40146 , 2327 , 24144 , 3108 , 2132993 , 2201704 , 54675783 , 2743305 , 3242068 , 3092847 , 3236575 , 2990891 , 660989 , 187 , 3442589 , 14369 , 4673656 , 3138373 , 2057112 , 444254 , 1473386 , 16231 , 3094465 , 665530 , 948382 , 2426546 , 10212 , 2998488 , 54677971 , 750895 , 1548942 , 68872 , 3132640 , 2998359 , 3697226]

1540|P06746(44) [1719874 , 26695 , 3842920 , 1967 , 10621 , 650908 , 54690031 , 11683 , 3182 , 10168 , 5289501 , 21501 , 3163418 , 8144119 , 57469 , 4343310 , 1985 , 3151041 , 114924 , 1599306 , 2063649 , 2896475 , 539709 , 4487 , 660883 , 56948249 , 2768954 , 349503 , 1568843 , 4342 , 581148 , 28446 , 911675 , 3168 , 2327 , 4777942 , 4116037 , 1870615 , 719632 , 10633 , 265580 , 12555 , 261282 , 5335 , 2333 , 1780 , 327045 , 647884 , 20544 , 2768975 , 703905 , 5804 , 3503 , 54675783 , 660708 , 3245402 , 1973720 , 1094968 , 3245025 , 178144 , 894690 , 54680702 , 15139 , 13791]

1541|P08908(41) [4850 , 3055171 , 4828 , 3822 , 9805719 , 5074 , 4830 , 5265 , 54746 , 5268 , 4585 , 5073 , 163925 , 3033769 , 3559 , 5736 , 31101 , 2477 , 28864 , 27400 , 5761 , 9826744 , 47811 , 5002 , 6005 , 16362 , 60795 , 71351 , 55752 , 9966051 , 2818 , 62865 , 8969 , 2726 , 130918 , 198746 , 57347 , 56971 , 54562 , 219050 , 197706 , 208951 , 60809 , 5358 , 443951 , 11430856 , 60857 , 72036 , 37459 , 3372 , 71360 , 77993 , 60854 , 5452 , 6918314 , 60149 , 198757 , 91273 , 4440 , 77992 , 128919 , 5078 , 4106 , 3396 , 55191]

1542|P35348(70) [3559 , 28864 , 11597698 , 9860294 , 148842 , 2913 , 5265 , 4184 , 23897 , 208898 , 72106 , 4850 , 5816 , 5073 , 3389 , 5074 , 5401 , 443951 , 5312125 , 37632 , 6041 , 164089 , 60602 , 12454 , 115237 , 119828 , 10531 , 54746 , 4636 , 2435 , 219050 , 3157 , 3404 , 3168 , 3016 , 5268 , 60820 , 17747460 , 129211 , 3698 , 6077 , 13542 , 22297 , 6082 , 11954293 , 2170 , 1355 , 10624 , 439260 , 40589 , 119570 , 2818 , 3822 , 16362 , 38521 , 2216 , 2159 , 2368 , 5775 , 4893 , 1615 , 3677 , 4493 , 2092 , 216249 , 33625]

1543|P19838(383) [16362 , 175540 , 115157 , 16960 , 3151 , 160355 , 1045 , 5233 , 361655 , 11293 , 4278 , 2081 , 155774 , 3433 , 16739648 , 107782 , 3117 , 6603901 , 1967 , 3973 , 3885 , 4593 , 2717 , 2216 , 5593 , 101616 , 6603842 , 21138 , 2812 , 104926 , 3108 , 28803 , 123600 , 3559 , 19996 , 4578 , 10219 , 123895 , 25644 , 5289501 , 3478 , 1238 , 5074 , 10382715 , 2229 , 2576 , 1694 , 199 , 5335 , 3542 , 2170 , 3455 , 208820 , 2725 , 3540 , 133621 , 3404 , 4122 , 3333 , 3503 , 4493 , 53708 , 4592 , 36811 , 4342 , 1433 , 28688]

1544|P00734(267) [24963036 , 25220914 , 204102 , 6540268 , 5494440 , 9574101 , 25271577 , 447733 , 107706 , 17754066 , 1746 , 44141860 , 183797 , 11957380 , 25113127 , 44331389 , 446805 , 25011733 , 10095865 , 3156995 , 10182969 , 42601552 , 104625 , 46228924 , 46937056 , 9820034 , 25113614 , 25113126 , 445843 , 42615254 , 448955 , 10621 , 25113617 , 16129704 , 24754814 , 185915 , 448677 , 1507 , 6445226 , 10820951 , 4634038 , 11641515 , 24963035 , 447732 , 25113128 , 448953 , 122267 , 152951 , 24963037 , 216210 , 24800541 , 25113616 , 10343728 , 46937030 , 1792 , 25011732 , 448042 , 17758361 , 9914780 , 42615253 , 10324367 , 5494439 , 25021183 , 23629654 , 25113615 , 44346051 , 25134248]

1545|P12931(96) [5287463 , 5287461 , 11608401 , 16040294 , 57379345 , 24889392 , 447532 , 11712649 , 11364421 , 447077 , 10427712 , 11656518 , 46937131 , 11409972 , 176167 , 10401956 , 25174101 , 5287550 , 17755052 , 160355 , 3025986 , 153999 , 3038522 , 10127622 , 11338033 , 447527 , 16722836 , 2856 , 23635314 , 6419766 , 176870 , 11552706 , 11427553 , 4708 , 5289215 , 15983966 , 16122633 , 151194 , 5328940 , 5005498 , 9549303 , 76098 , 10302451 , 6918454 , 156414 , 11234052 , 24779724 , 3973 , 4030 , 4369496 , 11314340 , 11751922 , 5287488 , 10915062 , 10113978 , 2396 , 447966 , 448008 , 24826799 , 11667893 , 11213558 , 10074640 , 208908 , 11485656 , 5287465 , 3062316 , 447537 , 447534]

1546|O15296(25) [3973 , 16362 , 3138375 , 170344 , 160355 , 1238 , 123409 , 2247 , 151506 , 10219 , 37175 , 21138 , 2081 , 1568843 , 5480 , 3760 , 15139 , 68186 , 192197 , 2562 , 683816 , 2448 , 12555 , 193949 , 3239105 , 3713404 , 11293 , 10382715 , 3240818 , 2812 , 101616 , 2318 , 2090 , 2170 , 10831 , 2327 , 15443 , 7329 , 2466 , 3503 , 5074 , 2540 , 1547484 , 124087 , 133621 , 3455 , 10230 , 3151 , 65909 , 16363 , 2132 , 3559 , 441383 , 10168 , 3108 , 3244425 , 127404 , 115015 , 3404 , 6918508 , 3885 , 19675 , 1355 , 3117 , 10206 , 3926 , 2377 , 2913 , 114924]

1547|Q9NR56(17) [4337923 , 2179 , 4493 , 4360846 , 24239 , 2113270 , 11683 , 19646 , 3799111 , 2082 , 3240818 , 13789 , 54675783 , 71407 , 16191372 , 2265 , 1893 , 7475368 , 327045 , 2762 , 3240179 , 660989 , 2829782 , 2950 , 4622 , 2426546 , 3138370 , 41684 , 316274 , 3885 , 3503 , 824727 , 4343310 , 3746037 , 3435 , 3237465 , 89105 , 801418 , 752652 , 3698 , 2315667 , 3244425 , 265580 , 3245131 , 107782 , 3117 , 70846 , 7475448 , 1649 , 2435 , 5359646 , 19910 , 3781338 , 10104227 , 612424 , 3245402 , 21501 , 4760 , 1985 , 3217977 , 3108 , 3238649 , 16231 , 3033877 , 2844395 , 219269 , 14369 , 10168 , 3676681 , 53708]

1548|P42858(59) [156419 , 3117 , 3542 , 7329 , 3503 , 12492 , 1878823 , 3236874 , 1719873 , 72139 , 3610 , 4142675 , 2315667 , 151506 , 16231 , 327045 , 3094465 , 3156762 , 41684 , 68805 , 5187962 , 2950 , 1568843 , 3698 , 3686 , 2090 , 2259930 , 657534 , 2812 , 10219 , 20906 , 1552036 , 7347 , 6918508 , 1720828 , 10221470 , 351111 , 36303 , 10382715 , 36811 , 67686 , 893703 , 656641 , 3866131 , 13986 , 3455 , 2180707 , 5282060 , 3783853 , 3781338 , 11310 , 4030 , 2063649 , 19910 , 2453 , 1717864 , 3109 , 14868 , 824155 , 11296583 , 5405 , 31475 , 2562 , 1355 , 104762 , 16362 , 21453 , 2219849 , 4122 , 2247 , 2913]

1549|P10828(59) [19996 , 14899645 , 6764 , 6545 , 2562 , 12535 , 4342 , 10299876 , 5920 , 16362 , 2170 , 2123 , 8730 , 6 , 10082482 , 18303 , 8041 , 1355 , 5819 , 3503 , 4365905 , 7329 , 2391 , 3282 , 2735009 , 3885 , 5921 , 18635 , 31236 , 5282060 , 16574 , 3034285 , 2950 , 9862248 , 114924 , 104926 , 6540 , 115157 , 16231 , 3286 , 1989 , 4284 , 37175 , 520196 , 5803 , 11167 , 5289501 , 2450 , 160355 , 28803 , 10168 , 16734800 , 22571 , 25644 , 10913 , 2017 , 3108 , 448011 , 656641 , 32793 , 16181 , 9863447 , 1003 , 13 , 3698 , 71404 , 4493 , 5804 , 3639 , 1046 , 2717 , 2467]

1550|Q9UBT6(9) [5405 , 6982 , 5289501 , 24144 , 1973720 , 13791 , 11296583 , 54675783 , 6237 , 3503 , 265580 , 187 , 33630 , 5475 , 1694 , 25074470 , 10133 , 5917 , 2576 , 22571 , 31200 , 2090 , 1238 , 16129681 , 1050 , 35970 , 5510 , 163659 , 10168 , 78933 , 2799 , 46937134 , 6400 , 61574 , 14878 , 68876 , 1701 , 2484 , 204105 , 2016 , 10114 , 2315 , 1123 , 1433 , 1369 , 17141 , 5282176 , 121871 , 11967809 , 3455 , 16362 , 51040 , 5282060 , 54675757 , 6764 , 2017 , 3746037 , 54676538 , 11852 , 10036135 , 13081 , 327044 , 2540 , 10621 , 3333 , 122081 , 2725 , 3610 , 104741 , 41684 , 1057 , 1780 , 327045]

1551|P16050(84) [1017 , 1649 , 5074 , 5722 , 3243812 , 2750 , 2478 , 37175 , 4912 , 16590 , 10308106 , 1057 , 3969 , 3760 , 612424 , 1694 , 3237465 , 10168 , 2812 , 65889 , 1548942 , 3435 , 361655 , 3117 , 51040 , 10531 , 1066 , 6708773 , 3610 , 2540 , 15474019 , 2749 , 107715 , 1996 , 2159 , 2132 , 192197 , 11852 , 53708 , 3245131 , 104741 , 2419371 , 2366 , 10313 , 199 , 2435 , 3311 , 3830 , 151506 , 1967 , 3244566 , 170344 , 107883 , 60910 , 3542 , 8569 , 11286230 , 2950 , 24239 , 3973 , 158794 , 6603842 , 2090 , 2998 , 3244425 , 3559 , 16362 , 7329 , 13791 , 3503 , 3698 , 3246760 , 47472 , 3885 , 4592]

1552|Q16665(350) [54675783 , 3474 , 2466 , 11683 , 4030 , 10219 , 3503 , 19996 , 3830 , 3002119 , 1547484 , 2132 , 160355 , 3351 , 3108 , 3433 , 2391 , 1981 , 170344 , 2200 , 3151 , 3973 , 28803 , 5804 , 10868 , 16490 , 5405 , 6603842 , 3760 , 123600 , 108144 , 26596 , 2090 , 3885 , 3616 , 1238 , 3404 , 11954283 , 107782 , 361655 , 3698 , 4622 , 16363 , 2333 , 3730 , 3435 , 4380 , 2179 , 3168 , 11289 , 10237 , 19529 , 2794 , 16362 , 10168 , 7329 , 26258 , 2170 , 5420 , 11779629 , 2137 , 3295 , 31729 , 8569 , 4122 , 3333 , 36811 , 16574 , 16960 , 11852 , 227681 , 11293 , 1967 , 5722 , 155774 , 5074 , 47472 , 6914666 , 2812 , 3455]

1553|P42345(257) [16135625 , 6918454 , 6918289 , 151171 , 2396 , 51001932 , 447077 , 2856 , 10074640 , 6918508 , 3542 , 11409972 , 16736978 , 44224160 , 11485656 , 156414 , 10113978 , 1238 , 2170 , 3038522 , 11213558 , 208908 , 5469318 , 3973 , 11520894 , 15983966 , 176870 , 11712649 , 16362 , 9549303 , 45375953 , 10427712 , 25167777 , 24889392 , 11977753 , 24748573 , 5074 , 11364421 , 3151 , 11427553 , 10127622 , 11338033 , 11667893 , 176167 , 11656518 , 3885 , 23724530 , 5005498 , 10219 , 49784945 , 11314340 , 25033539 , 3559 , 44516953 , 5405 , 6419766 , 6603842 , 17755052 , 11234052 , 24779724 , 447966 , 5289501 , 25254071 , 151194 , 153999 , 2913 , 16722836 , 3117 , 25262965 , 3455 , 4122 , 59239165 , 2327 , 16231 , 3025986 , 3333 , 1967 , 25262792 , 3503 , 6442177]

1554|P54132(71) [2265 , 20544 , 5917 , 4814 , 3433 , 16362 , 1694 , 5289501 , 2435 , 3885 , 10219 , 11967800 , 175540 , 3973 , 2998 , 9363 , 21138 , 4362 , 119259 , 3311 , 3478 , 5405 , 5723 , 89105 , 11296583 , 1780 , 4342 , 3746037 , 3333 , 249 , 1967 , 2754 , 3277 , 2017 , 11967809 , 23897 , 4197 , 1973720 , 5722 , 2812 , 6603842 , 1392 , 1066 , 5282060 , 2277 , 1676 , 4593 , 104762 , 1123 , 1649 , 5074 , 4578 , 2123 , 3698 , 2179 , 10036135 , 3074827 , 2090 , 3503 , 4122 , 4843 , 114924 , 2750 , 2753 , 1893 , 2762 , 16231 , 3455 , 1355 , 6603901 , 5593 , 53708 , 6764 , 361655 , 2369 , 1993 , 31475 , 10168 , 3671 , 2216 , 3108 , 1989]

1555|P15428(64) [2197 , 2540 , 3708374 , 1984 , 1811924 , 5459671 , 1561922 , 3108 , 16231 , 3324 , 2082 , 660989 , 5723 , 32681 , 4842 , 4284 , 11289 , 10219 , 5722 , 2179 , 612424 , 2426546 , 24144 , 1694 , 3698 , 10651 , 107992 , 3244425 , 1893 , 3238649 , 11852 , 11790 , 683816 , 1780 , 227681 , 2333 , 4506 , 3503 , 3034012 , 2092 , 3277 , 4380 , 2932343 , 10036135 , 1057 , 5153171 , 76915 , 2828376 , 3828 , 2998 , 16590 , 4577033 , 3333 , 72139 , 3240818 , 8569 , 11293 , 2219849 , 53708 , 12028 , 16315 , 6918508 , 2950 , 1676 , 4593 , 10168 , 2435 , 3238154 , 1050 , 2122 , 2750 , 3474 , 7329 , 1719874 , 3237465 , 3117 , 10245972 , 115015 , 1322 , 3245624 , 4197 , 1967 , 2753]

1556|P35968(217) [6450551 , 9549303 , 176870 , 24812719 , 24779724 , 25116064 , 447077 , 156414 , 4030 , 9797919 , 6918454 , 5329099 , 11610113 , 10302451 , 208908 , 10275001 , 11234052 , 10113978 , 10427712 , 11314340 , 9911830 , 16122633 , 5289418 , 11349170 , 11973736 , 9811611 , 24889392 , 11608401 , 11667893 , 11409972 , 9808844 , 24826799 , 3081361 , 448008 , 17755052 , 11338033 , 46207586 , 3025986 , 160355 , 16722836 , 42642645 , 447966 , 151194 , 15991573 , 10074640 , 9549295 , 16662431 , 6420138 , 11712649 , 11656518 , 11427553 , 11364421 , 57379345 , 24767976 , 10296883 , 25102847 , 11552706 , 10458325 , 6419766 , 10127622 , 2396 , 9933475 , 15983966 , 2856 , 5005498 , 10138259 , 9823820 , 9868037 , 24901704 , 3973 , 3038522 , 51039095 , 16040289 , 176167 , 5329102 , 11213558 , 9809715 , 11485656 , 153999 , 11751922 , 76098 , 11167118 , 53235510 , 25031915 , 11442891 , 11167602]

1557|P24941(127) [5327686 , 10224714 , 176870 , 11285002 , 11427553 , 5287969 , 447656 , 10127622 , 11338033 , 16718576 , 5326739 , 151194 , 3078519 , 680935 , 6918834 , 17754027 , 5327096 , 9601217 , 5005498 , 16122633 , 176167 , 160355 , 16113377 , 6852201 , 10113978 , 118458 , 16739650 , 153999 , 11712649 , 17755052 , 5289419 , 11234052 , 6918852 , 3038522 , 87031 , 449087 , 4592 , 2608 , 156414 , 57379345 , 24901723 , 1369 , 10074640 , 447966 , 11213558 , 447961 , 208908 , 448991 , 11314340 , 449088 , 447960 , 6918454 , 11270500 , 16058637 , 11409972 , 447077 , 11608401 , 1707 , 76098 , 11656518 , 4566 , 11485656 , 24889392 , 447655 , 24864077 , 9994066 , 15983966 , 10427712 , 24864078 , 447766 , 11442891 , 16722836 , 11667893 , 9547890 , 5288016 , 1540 , 447649 , 11552706 , 4564 , 24963033 , 4565 , 5289411 , 445967 , 5327131 , 448008 , 3025986 , 17754054 , 46926350 , 11610113 , 11364421 , 24779724 , 9926933]

1558|Q99714(31) [51040 , 15286 , 3117 , 593113 , 11293 , 3245131 , 21307 , 2333 , 10660 , 2123 , 2327 , 16231 , 3333 , 107992 , 4842 , 3324 , 7916 , 547914 , 648831 , 2057112 , 10245972 , 21138 , 5593 , 3239879 , 2753 , 5227 , 4064 , 2913 , 2179 , 3244425 , 5335 , 10313 , 2540 , 3474 , 1548942 , 11289 , 4593 , 2277 , 665652 , 3503 , 1676 , 1815815 , 612424 , 3237465 , 2092 , 28688 , 3698 , 1694 , 155774 , 1561922 , 2216 , 10219 , 10168 , 3243850 , 2165979 , 2355 , 2304617 , 11852 , 6307 , 24144 , 1984 , 6604423 , 5074 , 4278 , 2435 , 4380 , 3238649 , 3240818 , 11790 , 1057 , 3108 , 660989 , 3542 , 193949 , 53708 , 19996 , 25670 , 115015 , 2750 , 3277 , 3973 , 4670 , 5289501 , 2315 , 361655 , 3118 , 11241 , 41684 , 1893 , 2950 , 2866904 , 6605027 , 10651 , 7329 , 12492 , 114924 , 36811 , 15474019]

1559|P49798(18) [4278 , 2396 , 4761 , 107992 , 10245190 , 1568843 , 1676 , 2750 , 1045 , 4593 , 44602029 , 10245972 , 53708 , 3542 , 2768954 , 50287 , 23897 , 3762 , 683816 , 12449 , 2743305 , 3983561 , 10212 , 3151 , 739358 , 5917 , 3298363 , 3092847 , 44112 , 3286 , 123600 , 115015 , 6603842 , 4197 , 3117 , 20544 , 1392 , 1273944 , 175540 , 3973 , 219081 , 361655 , 70464 , 3138330 , 3245131 , 2123 , 1694 , 208820 , 2812 , 5005498 , 1066 , 3455 , 2831167 , 127599 , 16351 , 1892 , 104762 , 2435 , 10531 , 1993 , 31729 , 1234 , 124663 , 2216 , 10168 , 4362 , 1811924 , 2090 , 107883 , 2234617 , 3108 , 547914 , 2913 , 2327 , 4380 , 3698 , 2562 , 4680274 , 107715 , 4342 , 16231 , 1967 , 13752 , 10133 , 2914644 , 3885 , 14899645 , 2229 , 68186 , 3244425 , 10569483 , 5074 , 348986 , 114924 , 3433 , 3503 , 3746037 , 3559]

1560|Q9NUW8(14) [11293 , 123600 , 130881 , 19910 , 2265 , 15158 , 3686 , 2576 , 5593 , 121871 , 1030 , 213013 , 115150 , 10168 , 106729 , 16189712 , 54675783 , 122081 , 107715 , 2396 , 11245 , 11241 , 1549789 , 65909 , 3503 , 2178 , 21138 , 3760 , 5510 , 3156917 , 5335 , 51040 , 121752 , 5074 , 16837 , 6708773 , 31957 , 10206 , 1989 , 2453 , 3969 , 3151041 , 6034 , 10133 , 7916 , 1967 , 2950 , 7191 , 22430815 , 2132 , 16362 , 2170 , 32681 , 2327 , 2750 , 10230 , 16231 , 9273 , 10621 , 1895388 , 3108 , 3455 , 2562 , 4487 , 2812 , 3610 , 47472 , 170344 , 4337923 , 68089 , 10245972 , 246831 , 35970 , 8041 , 4284 , 2264 , 192197 , 115015 , 61247 , 3229428 , 4592 , 1780 , 5233 , 18635 , 10531 , 14052 , 4456136 , 4174 , 4842 , 5326713 , 1355 , 6764 , 26323 , 3295 , 3885 , 175540 , 1781 , 1057]

1561|O75164(18) [21109 , 2016 , 3132640 , 54677971 , 3138330 , 1066 , 3455 , 660989 , 3746037 , 10172943 , 2855211 , 10621 , 7329 , 3003803 , 2478 , 3758 , 2366 , 24792593 , 2165605 , 3138375 , 1870615 , 2762 , 3926 , 3118 , 1349907 , 2090 , 2182 , 2179 , 67686 , 3138373 , 14052 , 2794 , 1967 , 10212 , 11293 , 3760 , 3151041 , 131411 , 3298512 , 6 , 547914 , 2812 , 54675783 , 14369 , 32681 , 2876323 , 68089 , 2866904 , 3286 , 1369 , 3139316 , 441383 , 5074 , 4119575 , 2743305 , 683816 , 5392 , 11852 , 2327 , 3125446 , 3406 , 1720828 , 12454 , 801418 , 24892221 , 16347 , 3240461 , 16190692 , 54676538 , 612424 , 31475 , 3126341 , 3515 , 19910 , 10168 , 219081 , 3138364 , 51040 , 28446 , 2998359 , 10036135 , 3120949 , 115163 , 4680274 , 1973720 , 5510 , 598513 , 4619 , 3117 , 13752 , 3229428 , 10206 , 2453 , 752652 , 16362 , 12449 , 685814 , 3244425 , 76915]

1562|Q9UIF8(0) [76915 , 3610 , 1017 , 2876323 , 24761713 , 10168 , 12454 , 5510 , 4961961 , 647499 , 3298512 , 1989 , 3132640 , 659036 , 18573525 , 2984762 , 3760 , 3236936 , 3242481 , 68089 , 5074 , 19910 , 2343 , 10219 , 1561922 , 2812 , 3229428 , 3377088 , 9550559 , 131411 , 3503 , 10235 , 72900 , 3108 , 1829960 , 21501 , 2122 , 10258 , 2247 , 2315667 , 31475 , 1967 , 2333 , 3151041 , 290012 , 3542 , 612424 , 648831 , 2327 , 2950 , 5359646 , 3731631 , 3932 , 54676538 , 2743305 , 3237465 , 15723 , 645503 , 3435 , 660989 , 911675 , 2229 , 3126341 , 1547484 , 332697 , 32681 , 5804 , 1973720 , 14369 , 3686 , 194595 , 661085 , 752652 , 4110197 , 3245728 , 41684 , 1780 , 16362 , 2754 , 567825 , 16129778 , 2366 , 8210 , 265580 , 3474 , 4760 , 1870615 , 10621 , 11852 , 70846 , 54675783 , 54677971 , 1066 , 2197 , 124087 , 36303 , 3746037 , 3118 , 3244425 , 2179 , 4119575 , 21109]

1563|Q08209(55) [71851 , 3334 , 5921 , 5585 , 3748 , 5541 , 21109 , 2883 , 1548942 , 4122 , 2754 , 4107 , 11643449 , 3639 , 441383 , 1981 , 4912 , 4197 , 5335 , 1349907 , 4843 , 35802 , 3333 , 14385 , 2264 , 2082 , 3686 , 1057 , 2197 , 14868 , 2361 , 2749 , 2732 , 10219 , 2812 , 3516 , 2123 , 4156 , 10531 , 14052 , 5405 , 54676038 , 4174 , 2435 , 44112 , 3055 , 13765 , 16231 , 2482 , 2725 , 2753 , 119259 , 2750 , 54677971 , 3435 , 2366 , 2577 , 1030 , 3561 , 17134 , 4855 , 2484 , 16129778 , 21138 , 50942 , 2758 , 10660 , 3117 , 3559 , 3406 , 5576 , 11683 , 39042 , 3168 , 36811 , 1046 , 3478 , 3698 , 1547484 , 12124 , 2333 , 2467 , 3151 , 4760 , 2893 , 6761 , 3182 , 107782 , 2170 , 4030 , 2265 , 5593 , 11286230 , 2913 , 3973 , 182137 , 3108 , 57469 , 2247 , 124087 , 2132 , 4506 , 5510 , 31072 , 11790 , 4753 , 123600 , 5198 , 2478 , 16574 , 131204 , 2391 , 3324 , 2176 , 175540 , 5722]

1564|P51679(50) [14385 , 2082 , 2170 , 1030 , 3561 , 17134 , 3055 , 16129778 , 2482 , 2725 , 4912 , 36811 , 3478 , 2577 , 2467 , 3151 , 21138 , 31072 , 3168 , 14052 , 2758 , 3117 , 54676038 , 5593 , 6761 , 44112 , 107782 , 4855 , 5576 , 2265 , 2176 , 123600 , 2264 , 175540 , 3686 , 2913 , 119259 , 2893 , 54677971 , 3435 , 3182 , 124087 , 50942 , 11790 , 4753 , 10660 , 3559 , 2750 , 4030 , 182137 , 3108 , 13765 , 2247 , 3324 , 5722 , 3334 , 12124 , 2333 , 4506 , 5510 , 4760 , 2883 , 5335 , 3333 , 1548942 , 11286230 , 2197 , 441383 , 2749 , 5921 , 5585 , 2732 , 3973 , 10219 , 4197 , 57469 , 4122 , 65015 , 4843 , 2478 , 4174 , 2435 , 131204 , 1057 , 3698 , 1981 , 2391 , 3406 , 2132 , 16231 , 71851 , 2361 , 3748 , 1349907 , 5541 , 21109 , 2812 , 35802 , 3516 , 2123 , 2366 , 10531 , 2754 , 4107 , 11643449 , 2484 , 3639 , 14868 , 1547484 , 5405 , 5198 , 16574 , 11683 , 39042 , 1046 , 4156 , 2753]

1565|P32241(49) [10219 , 3055 , 14052 , 39042 , 16129778 , 182137 , 5593 , 3406 , 36811 , 4855 , 71851 , 2725 , 2913 , 4030 , 31072 , 57469 , 2754 , 50942 , 2893 , 4506 , 3559 , 2478 , 2435 , 2170 , 2265 , 4843 , 131204 , 5585 , 35802 , 10531 , 124087 , 14868 , 3686 , 11643449 , 1030 , 17134 , 16231 , 4107 , 1057 , 2366 , 4912 , 2484 , 1548942 , 4156 , 6761 , 1046 , 5722 , 5405 , 3168 , 54677971 , 3435 , 3639 , 2132 , 2123 , 5510 , 3182 , 11286230 , 2176 , 3108 , 4760 , 10660 , 107782 , 3973 , 2361 , 2482 , 11790 , 2577 , 4753 , 123600 , 2391 , 2467 , 5198 , 4174 , 11683 , 5576 , 13765 , 3516 , 2750 , 2753 , 3333 , 44112 , 1981 , 1547484 , 119259 , 1349907 , 2082 , 3561 , 2883 , 44623946 , 2758 , 3324 , 2264 , 3334 , 54676038 , 16574 , 21138 , 2732 , 2749 , 2197 , 2247 , 5335 , 441383 , 2812 , 12124 , 3748 , 3117 , 4197 , 2333 , 5921 , 4122 , 3698 , 5541 , 21109 , 175540 , 3151 , 14385 , 3478]

1566|P49146(14) [4107 , 5585 , 11286230 , 4156 , 2482 , 2197 , 12124 , 2435 , 1030 , 3973 , 3333 , 1548942 , 5921 , 5541 , 21138 , 4753 , 2577 , 2265 , 44112 , 17134 , 4843 , 1981 , 2391 , 182137 , 11643449 , 2247 , 5576 , 10660 , 1057 , 119259 , 2753 , 2883 , 4506 , 3055 , 2484 , 5722 , 187 , 14052 , 10219 , 39042 , 2732 , 2170 , 1547484 , 123600 , 3334 , 14868 , 3748 , 3559 , 3698 , 2725 , 21109 , 2176 , 5510 , 71851 , 3516 , 3435 , 3324 , 131204 , 3478 , 3108 , 4760 , 31072 , 2264 , 11790 , 2478 , 3182 , 2132 , 50942 , 13765 , 4197 , 3151 , 3406 , 441383 , 2893 , 2333 , 4174 , 2361 , 16129778 , 175540 , 36811 , 2749 , 2366 , 10531 , 2750 , 2754 , 3561 , 2758 , 3639 , 4912 , 35802 , 2467 , 2913 , 16231 , 1046 , 2812 , 3168 , 14385 , 3686 , 124087 , 5198 , 6761 , 16574 , 2123 , 3117 , 54676038 , 4122 , 5335 , 4855 , 4030 , 5593 , 107782 , 2082 , 5405 , 54677971 , 11683 , 1349907 , 57469]

1567|P08575(136) [11683 , 3973 , 2361 , 2435 , 2265 , 2732 , 2170 , 131204 , 21109 , 3334 , 2176 , 4912 , 50942 , 2577 , 10660 , 11790 , 4753 , 3561 , 2913 , 3639 , 5510 , 1030 , 5576 , 124087 , 5593 , 12124 , 54676038 , 1548942 , 13765 , 3055 , 2812 , 57469 , 4760 , 54677971 , 3108 , 10219 , 2123 , 4855 , 2750 , 16574 , 9547959 , 31072 , 16231 , 182137 , 5541 , 1981 , 175540 , 2758 , 2391 , 4843 , 11286230 , 3516 , 17134 , 3748 , 4156 , 123600 , 39042 , 3151 , 11643449 , 5405 , 1547484 , 35802 , 2883 , 4122 , 2754 , 2893 , 4030 , 3324 , 3182 , 2333 , 14385 , 3478 , 3435 , 3117 , 1349907 , 5585 , 2082 , 3698 , 2753 , 2482 , 2197 , 3168 , 441383 , 5722 , 5335 , 36811 , 10531 , 2725 , 3686 , 21138 , 2264 , 4506 , 1046 , 6761 , 16129778 , 4197 , 14052 , 2366 , 5921 , 1057 , 2132 , 3559 , 2478 , 2484 , 107782 , 5198 , 4107 , 2467 , 2749 , 3406 , 4174 , 44112 , 3333 , 14868 , 71851 , 119259 , 2247]

1568|P30988(44) [2170 , 6761 , 5335 , 2750 , 3168 , 4122 , 2758 , 2264 , 16231 , 5541 , 11643449 , 16574 , 1548942 , 70691388 , 2391 , 3748 , 123600 , 2732 , 3516 , 12124 , 10531 , 5405 , 2333 , 13765 , 21109 , 3333 , 1547484 , 11683 , 2176 , 4197 , 71851 , 14052 , 21138 , 4912 , 2725 , 11286230 , 3055 , 2754 , 2812 , 4030 , 1046 , 54677971 , 3435 , 2749 , 5921 , 36811 , 2753 , 441383 , 2913 , 4174 , 57469 , 3639 , 3973 , 2478 , 3151 , 3324 , 3478 , 2893 , 3334 , 131204 , 44112 , 4843 , 175540 , 5593 , 14868 , 5585 , 2883 , 14385 , 124087 , 2482 , 2197 , 2577 , 3698 , 4156 , 10219 , 4506 , 16129778 , 2132 , 2484 , 2247 , 50942 , 3108 , 2366 , 1349907 , 2082 , 54676038 , 4760 , 5722 , 10660 , 182137 , 3559 , 5510 , 2123 , 3182 , 2265 , 3686 , 107782 , 1981 , 35802 , 3561 , 1057 , 2361 , 3406 , 4855 , 2435 , 5576 , 11790 , 4753 , 39042 , 4107 , 1030 , 5198 , 17134 , 31072 , 119259 , 3117 , 2467]

1569|P25929(16) [4122 , 4855 , 3117 , 3182 , 5921 , 3108 , 10660 , 14052 , 1057 , 119259 , 182137 , 11683 , 124087 , 1547484 , 2197 , 57469 , 3686 , 2132 , 1548942 , 21109 , 13765 , 2812 , 31072 , 10531 , 17134 , 11643449 , 4843 , 3561 , 3324 , 2754 , 2366 , 2577 , 1349907 , 5335 , 3698 , 12124 , 50942 , 5198 , 3748 , 175540 , 2247 , 54676038 , 44112 , 16129778 , 11286230 , 5722 , 3435 , 11790 , 39042 , 4753 , 2082 , 1046 , 10219 , 2883 , 107782 , 4506 , 2264 , 2176 , 6761 , 2913 , 14868 , 187 , 3333 , 2361 , 4760 , 7504 , 5541 , 2753 , 2391 , 3334 , 5510 , 2750 , 2482 , 2333 , 2467 , 4156 , 4030 , 2732 , 2725 , 2435 , 4107 , 123600 , 3639 , 14385 , 131204 , 4174 , 71851 , 2749 , 35802 , 1981 , 2265 , 2123 , 3055 , 3478 , 3516 , 5405 , 2758 , 3559 , 2170 , 16574 , 3151 , 1030 , 4197 , 3406 , 4912 , 2484 , 21138 , 441383 , 16231 , 2478 , 5593 , 3168 , 5585 , 54677971 , 2893 , 5576 , 3973 , 36811]

1570|P25024(78) [2264 , 2082 , 2366 , 10219 , 3478 , 2197 , 2812 , 1349907 , 3324 , 5510 , 4174 , 2484 , 2265 , 2893 , 1981 , 10531 , 3973 , 123600 , 3516 , 131204 , 3748 , 3698 , 9865554 , 1057 , 35802 , 2176 , 4753 , 3182 , 2435 , 16231 , 2478 , 182137 , 2361 , 57469 , 2750 , 2758 , 54676038 , 4197 , 3406 , 1547484 , 54677971 , 5722 , 11643449 , 3055 , 2123 , 4030 , 2732 , 4855 , 124087 , 10660 , 12124 , 3561 , 9838712 , 4156 , 4912 , 4506 , 16574 , 11683 , 2467 , 2577 , 5198 , 11286230 , 2170 , 175540 , 2247 , 5593 , 3334 , 11790 , 1548942 , 3559 , 1046 , 2132 , 5335 , 5541 , 5585 , 2391 , 16007088 , 3151 , 44112 , 13765 , 2754 , 3108 , 2753 , 441383 , 5576 , 6761 , 1030 , 4107 , 3168 , 36811 , 2913 , 4760 , 3686 , 119259 , 3639 , 4122 , 3117 , 14052 , 4843 , 2749 , 39042 , 3435 , 31072 , 3333 , 17134 , 21109 , 107782 , 71851 , 14868 , 21138 , 2333 , 5405 , 2725 , 14385 , 2883 , 5921 , 16129778 , 2482 , 50942]

1571|P33032(4) [44112 , 2754 , 3055 , 10219 , 16129664 , 3108 , 107782 , 4506 , 1057 , 2753 , 5593 , 2132 , 6761 , 11683 , 2812 , 182137 , 1046 , 3334 , 3324 , 5541 , 2883 , 2750 , 16197727 , 21109 , 4030 , 54677971 , 12124 , 5335 , 2366 , 50942 , 3516 , 21138 , 175540 , 4912 , 2265 , 3698 , 5585 , 3748 , 5576 , 54676038 , 123600 , 2170 , 16129778 , 16574 , 2247 , 2913 , 57469 , 2176 , 10660 , 39042 , 4753 , 119259 , 2082 , 2264 , 4197 , 36811 , 17134 , 14868 , 5198 , 4760 , 11643449 , 3333 , 13765 , 4843 , 10531 , 2482 , 5510 , 2577 , 4122 , 3561 , 2732 , 11328898 , 2435 , 2333 , 1349907 , 2467 , 3639 , 31072 , 71851 , 3686 , 3182 , 3117 , 2123 , 124087 , 2197 , 1981 , 14385 , 11286230 , 14052 , 5405 , 131204 , 3478 , 1548942 , 4174 , 3151 , 1547484 , 1030 , 2749 , 35802 , 11790 , 4107 , 2484 , 2758 , 3559 , 3406 , 441383 , 5722 , 2478 , 2361 , 3973 , 2391 , 3168 , 2893 , 16231 , 5921 , 2725 , 3435 , 4855 , 4156]

1572|Q16236(220) [1568843 , 3377088 , 13791 , 8178 , 19996 , 16316 , 660989 , 3082 , 2478 , 2836838 , 3503 , 18573528 , 213013 , 84098 , 67686 , 3034285 , 1614257 , 6540 , 2750 , 8124 , 2170 , 16188984 , 4404908 , 197033 , 5233 , 651913 , 1893 , 151506 , 15439 , 16960 , 160355 , 3114023 , 133621 , 6307 , 657497 , 2913 , 31475 , 7347 , 1392 , 3242535 , 312183 , 3244566 , 9820526 , 5392 , 54676538 , 54675783 , 1720828 , 1123 , 104741 , 4342 , 8872 , 11742 , 6603901 , 35758 , 4961961 , 2179 , 101744 , 11006 , 2166261 , 31200 , 1548942 , 10850 , 3114024 , 27648 , 114924 , 62485 , 12589 , 39040 , 3559 , 12968 , 4156 , 13113 , 6301 , 16231 , 658365 , 2247 , 115157 , 10958 , 1694 , 11604 , 14369 , 9298 , 5289501 , 348986 , 3138330 , 3156709 , 3885 , 16653 , 3781338 , 1895388 , 104926 , 68363 , 1057 , 225371 , 8467 , 2137779 , 2365 , 5459650 , 344675 , 4572075 , 2355 , 3326 , 16637 , 9551522 , 76915 , 10245972 , 10660 , 7475414 , 8907 , 2082 , 7395618 , 3672772 , 22571 , 657677 , 650127 , 44142959 , 273053 , 65768 , 2450 , 7475368]

1573|P41968(13) [5405 , 57469 , 5335 , 21138 , 3698 , 2913 , 441383 , 119259 , 2732 , 10219 , 2361 , 16129778 , 2265 , 44112 , 1981 , 5198 , 5585 , 2333 , 71851 , 4197 , 17134 , 2754 , 11643449 , 3055 , 2170 , 2749 , 50942 , 2197 , 54677971 , 175540 , 3559 , 4843 , 5510 , 1057 , 16231 , 4156 , 182137 , 2482 , 3117 , 3108 , 6761 , 36811 , 3478 , 3334 , 10660 , 5722 , 16129664 , 5921 , 3151 , 3973 , 2132 , 4030 , 3516 , 4912 , 1030 , 4174 , 2435 , 4753 , 11790 , 5576 , 3406 , 11683 , 2758 , 5541 , 14385 , 5593 , 14052 , 35802 , 4506 , 3639 , 1046 , 131204 , 31072 , 16574 , 10531 , 21109 , 3168 , 2247 , 14868 , 2725 , 3748 , 107782 , 1547484 , 2753 , 2264 , 4107 , 2467 , 6323491 , 54676038 , 11286230 , 2484 , 2750 , 12124 , 2812 , 39042 , 3561 , 2082 , 3686 , 2478 , 124087 , 1349907 , 2577 , 4122 , 2366 , 11328898 , 2883 , 3182 , 2391 , 16197727 , 2893 , 2176 , 3324 , 1548942 , 4855 , 2123 , 4760 , 3333 , 3435 , 123600 , 13765]

1574|P08311(36) [50942 , 3686 , 124087 , 57469 , 131204 , 35802 , 2732 , 2812 , 13765 , 21138 , 2132 , 2754 , 107782 , 1046 , 3748 , 2467 , 1057 , 3108 , 4855 , 441383 , 1030 , 2577 , 1349907 , 182137 , 2333 , 10219 , 14052 , 3516 , 24800541 , 2176 , 2366 , 3151 , 4122 , 5405 , 5921 , 5541 , 10660 , 3117 , 123600 , 4030 , 11790 , 2893 , 2484 , 2482 , 2197 , 5335 , 4760 , 2391 , 3559 , 31072 , 1548942 , 2082 , 2264 , 3561 , 2749 , 16574 , 2247 , 36811 , 4174 , 11286230 , 6761 , 2265 , 17134 , 4506 , 2753 , 10531 , 2435 , 4912 , 3333 , 5593 , 16129778 , 1981 , 2361 , 3334 , 39042 , 5576 , 107706 , 3406 , 4197 , 3639 , 2750 , 14385 , 119259 , 4753 , 2913 , 3698 , 5510 , 42601552 , 2170 , 4156 , 16231 , 2725 , 54676038 , 71851 , 3182 , 14868 , 3324 , 446833 , 21109 , 5722 , 44112 , 3973 , 11643449 , 2478 , 175540 , 12124 , 2758 , 3435 , 11683 , 3055 , 5198 , 4107 , 2123 , 3478 , 5585 , 54677971 , 1547484 , 3168 , 4843 , 2883]

1575|P30411(40) [2732 , 119259 , 175540 , 2577 , 5198 , 10660 , 16231 , 11790 , 2913 , 1046 , 21138 , 17134 , 5541 , 2758 , 11643449 , 4753 , 4843 , 10531 , 4912 , 2366 , 2893 , 4107 , 16129778 , 1349907 , 107782 , 4030 , 3561 , 11286230 , 2197 , 2176 , 4156 , 57469 , 54677971 , 14052 , 441383 , 3151 , 3117 , 3055 , 5405 , 2123 , 1548942 , 3748 , 5576 , 2484 , 2264 , 131204 , 31072 , 50942 , 5510 , 2132 , 16574 , 2478 , 5585 , 1057 , 3559 , 3108 , 14868 , 2247 , 4760 , 2265 , 3686 , 21109 , 3698 , 3324 , 9831652 , 439201 , 11498853 , 124087 , 71851 , 2435 , 1981 , 2750 , 39042 , 3333 , 36811 , 5593 , 4122 , 3973 , 2361 , 44112 , 4197 , 3182 , 13765 , 3168 , 2391 , 2482 , 182137 , 5722 , 71364 , 4855 , 6761 , 44623946 , 5335 , 14385 , 3478 , 5921 , 3435 , 1030 , 11683 , 3516 , 123600 , 2725 , 2082 , 2467 , 35802 , 1547484 , 2812 , 12124 , 54676038 , 3406 , 2883 , 2170 , 3334 , 4506 , 2749 , 2754 , 3639 , 2753 , 4174 , 2333 , 10219]

1576|Q99720(24) [36811 , 2123 , 2264 , 2913 , 5585 , 11286230 , 3478 , 6761 , 4030 , 3324 , 5405 , 3151 , 3108 , 39042 , 2361 , 3406 , 2391 , 2333 , 2132 , 13765 , 4855 , 4107 , 57469 , 16574 , 3748 , 5198 , 2754 , 50942 , 175540 , 4156 , 10531 , 44112 , 4506 , 14385 , 16231 , 71851 , 54677971 , 53389 , 2893 , 3435 , 31072 , 16129778 , 2197 , 3686 , 2883 , 14868 , 14052 , 5593 , 2170 , 2482 , 2812 , 35802 , 1547484 , 4197 , 2366 , 11790 , 1030 , 4753 , 10660 , 2082 , 1057 , 3168 , 54676038 , 4912 , 123600 , 11683 , 2577 , 119259 , 17134 , 3516 , 53359 , 4760 , 2478 , 2467 , 3639 , 4843 , 2732 , 2435 , 3055 , 3117 , 182137 , 4122 , 5541 , 4174 , 2753 , 1548942 , 5510 , 12124 , 5722 , 2176 , 21138 , 2750 , 3698 , 10219 , 11430856 , 2758 , 3333 , 3008 , 3559 , 2725 , 21109 , 131204 , 2749 , 3182 , 1046 , 124087 , 107782 , 5576 , 441278 , 3973 , 1981 , 2484 , 2247 , 441383 , 1349907 , 3561 , 5335 , 5360696 , 5921 , 3334 , 2265 , 11643449]

1577|P32245(43) [6323491 , 5593 , 124087 , 3406 , 14385 , 71851 , 2725 , 3055 , 39042 , 182137 , 31072 , 36811 , 16129664 , 57469 , 2754 , 50942 , 4030 , 1349907 , 2478 , 2758 , 2170 , 3639 , 2265 , 4843 , 2082 , 3559 , 2435 , 2893 , 3686 , 131204 , 2482 , 1030 , 17134 , 5585 , 4912 , 2484 , 35802 , 10531 , 14868 , 4156 , 1046 , 5405 , 54677971 , 16231 , 3435 , 4107 , 2366 , 2132 , 1548942 , 2123 , 6761 , 71768094 , 11286230 , 2176 , 16129778 , 3168 , 10660 , 3973 , 4855 , 5510 , 4760 , 2391 , 107782 , 2361 , 5198 , 11790 , 44112 , 2577 , 4753 , 123600 , 175540 , 5722 , 3516 , 3108 , 2753 , 1981 , 2467 , 119259 , 11683 , 5576 , 16197727 , 11328898 , 44623946 , 13765 , 2750 , 2913 , 3324 , 3333 , 1547484 , 3334 , 11643449 , 16574 , 3561 , 2883 , 2197 , 4506 , 2264 , 54676038 , 21138 , 2732 , 2749 , 5335 , 441383 , 2812 , 4174 , 12124 , 1057 , 3748 , 2247 , 3182 , 5921 , 3117 , 3151 , 4197 , 2333 , 4122 , 3478 , 3698 , 5541 , 10219 , 21109 , 14052]

1578|P21452(15) [4122 , 11790 , 3324 , 17134 , 2123 , 50942 , 21109 , 10660 , 3168 , 3748 , 6761 , 182137 , 44112 , 5541 , 4107 , 11643449 , 104974 , 5198 , 5510 , 3151 , 3639 , 16574 , 2732 , 10219 , 4156 , 2361 , 2913 , 3698 , 1046 , 3406 , 36811 , 2247 , 14385 , 2435 , 3686 , 3055 , 1547484 , 44623946 , 2333 , 4506 , 4753 , 123600 , 2478 , 131204 , 107782 , 4855 , 2812 , 2265 , 2391 , 2366 , 5722 , 71851 , 2758 , 5405 , 11527495 , 5921 , 219077 , 1349907 , 2484 , 3973 , 14052 , 11683 , 3333 , 39042 , 10328936 , 3117 , 5576 , 119259 , 14868 , 54677971 , 441383 , 2753 , 1030 , 3561 , 2750 , 2482 , 16129778 , 3435 , 1981 , 3182 , 3334 , 3559 , 4174 , 5311424 , 3108 , 57469 , 2883 , 5335 , 35802 , 4030 , 2082 , 1548942 , 2132 , 12124 , 13765 , 31072 , 2754 , 16231 , 3478 , 2893 , 2197 , 2725 , 2749 , 4912 , 2467 , 4843 , 4760 , 5593 , 3516 , 11286230 , 4197 , 2577 , 10531 , 5585 , 21138 , 2176 , 175540 , 54676038 , 2264 , 2170 , 1057 , 124087]

1579|P11509(69) [2725 , 4174 , 36811 , 2754 , 3334 , 2082 , 2264 , 2478 , 2170 , 39042 , 5335 , 12124 , 3748 , 31072 , 441383 , 2361 , 1548942 , 1547484 , 3698 , 2123 , 16574 , 2750 , 2391 , 5405 , 2812 , 2197 , 44112 , 2482 , 2913 , 2753 , 1057 , 11643449 , 3686 , 2366 , 14385 , 4506 , 16231 , 1349907 , 54677971 , 131204 , 2355 , 3324 , 2577 , 175540 , 2758 , 4912 , 182137 , 35802 , 123600 , 3516 , 5722 , 3639 , 10531 , 3117 , 3333 , 1981 , 4760 , 124087 , 3168 , 2176 , 4122 , 4843 , 11790 , 21109 , 4753 , 3108 , 2893 , 2883 , 1046 , 2467 , 5585 , 2732 , 2132 , 3435 , 2484 , 2265 , 3561 , 10660 , 11229234 , 16129778 , 11683 , 3182 , 1030 , 4107 , 5510 , 148201 , 11332763 , 14052 , 4030 , 4156 , 21138 , 4197 , 2435 , 51049968 , 54676038 , 5198 , 4855 , 5541 , 5576 , 50942 , 14868 , 107782 , 3559 , 3478 , 2247 , 5593 , 2333 , 1048 , 2749 , 119259 , 11286230 , 3055 , 57469 , 10219 , 3406 , 5921 , 17134 , 6761 , 3151 , 71851 , 3973 , 13765]

1580|P32238(17) [54676038 , 16231 , 1981 , 1046 , 2484 , 107782 , 5198 , 3406 , 12124 , 2725 , 3478 , 2478 , 2435 , 1547484 , 4030 , 2391 , 6761 , 14052 , 17134 , 1057 , 3333 , 5921 , 39042 , 3561 , 119259 , 4912 , 5405 , 11286230 , 4156 , 5541 , 2812 , 3973 , 60182 , 3324 , 3334 , 10660 , 4753 , 2247 , 5722 , 65937 , 13765 , 1548942 , 124087 , 10531 , 3698 , 50942 , 16129675 , 3639 , 2482 , 31072 , 2754 , 2749 , 2577 , 14868 , 21109 , 54677971 , 2758 , 2197 , 2893 , 71851 , 5576 , 3182 , 5510 , 2467 , 2082 , 4174 , 10343641 , 21138 , 4506 , 122077 , 5593 , 123600 , 2264 , 2123 , 4122 , 16129778 , 1030 , 4197 , 175540 , 2265 , 11643449 , 3516 , 16574 , 4760 , 2176 , 2361 , 2366 , 3748 , 11790 , 2732 , 3108 , 51353551 , 3168 , 5335 , 57469 , 3559 , 2132 , 2883 , 3151 , 3686 , 11683 , 2913 , 182137 , 4855 , 36811 , 5585 , 35802 , 3117 , 2753 , 1349907 , 44112 , 2333 , 131204 , 3435 , 4107 , 14385 , 2750 , 3055 , 4843 , 10219 , 441383 , 44623946 , 2170]

1581|P25105(36) [2753 , 2247 , 3698 , 54676038 , 3478 , 4753 , 2264 , 3973 , 3406 , 5541 , 3686 , 2478 , 3748 , 2913 , 2451 , 119259 , 4506 , 14052 , 4107 , 4156 , 2577 , 2482 , 36811 , 2082 , 3055 , 5585 , 1057 , 2170 , 3435 , 108156 , 10531 , 175540 , 44112 , 13765 , 21109 , 3168 , 17134 , 182137 , 4122 , 12124 , 4030 , 2123 , 5198 , 16129778 , 2265 , 3117 , 3333 , 2435 , 2883 , 35802 , 1981 , 5593 , 2499 , 3151 , 119175 , 16231 , 441383 , 3559 , 1030 , 11683 , 2725 , 2366 , 21138 , 65923 , 3516 , 3108 , 123600 , 2484 , 1046 , 4912 , 2732 , 5335 , 65889 , 2467 , 2750 , 16574 , 119368 , 71851 , 54677971 , 4197 , 5921 , 11790 , 2812 , 1349907 , 133017 , 10219 , 2361 , 1547484 , 2197 , 4843 , 11643449 , 5405 , 3639 , 50942 , 10660 , 5722 , 57469 , 4174 , 2758 , 4760 , 2749 , 107782 , 2754 , 3182 , 2176 , 31072 , 5576 , 2132 , 124087 , 39042 , 11286230 , 131204 , 14868 , 3334 , 2893 , 3324 , 5510 , 1548942 , 3561 , 6761 , 44370579 , 4855 , 14385 , 2391 , 2333]

1582|P05181(174) [2725 , 4753 , 2333 , 5576 , 11790 , 3182 , 2754 , 12124 , 4197 , 3168 , 16129778 , 2577 , 1349907 , 5198 , 2753 , 4506 , 11643449 , 3334 , 4855 , 1981 , 3478 , 2247 , 14052 , 3117 , 2750 , 2482 , 119259 , 4843 , 3151 , 16574 , 182137 , 3435 , 54677971 , 10660 , 4122 , 5510 , 3406 , 4156 , 51049968 , 31072 , 16960 , 2197 , 5585 , 5593 , 2467 , 441383 , 36811 , 11683 , 71851 , 124087 , 39042 , 54676038 , 1030 , 2812 , 4912 , 21109 , 2176 , 3324 , 2758 , 175540 , 3559 , 2361 , 1548942 , 4107 , 6761 , 10219 , 10783 , 2749 , 2366 , 2391 , 57469 , 2732 , 5722 , 17134 , 3516 , 14868 , 5541 , 2883 , 2170 , 3333 , 4174 , 4760 , 4030 , 2435 , 21138 , 148201 , 11332763 , 14385 , 35802 , 3973 , 3561 , 5405 , 2082 , 2264 , 3108 , 11286230 , 1547484 , 3748 , 1046 , 2265 , 2132 , 16590 , 44112 , 2950 , 19987169 , 1057 , 131204 , 5921 , 2913 , 5335 , 3698 , 2484 , 3686 , 11229234 , 3639 , 2123 , 50942 , 107782 , 16231 , 2893 , 3055 , 2478 , 10531 , 13765 , 123600]

1583|Q92830(13) [4843 , 3032361 , 115244 , 2170 , 3117 , 4855 , 2540 , 911675 , 4097 , 3163418 , 3814414 , 3607 , 1861634 , 6237 , 3828 , 3561 , 3748 , 54676538 , 294256 , 3245563 , 10767 , 3474 , 2963649 , 122077 , 3109 , 1052 , 2950 , 1829960 , 24761713 , 4119575 , 4506 , 11046239 , 3236575 , 2426546 , 1050 , 3245493 , 11296583 , 39299 , 32681 , 3108 , 2913 , 160355 , 539709 , 2450 , 2724 , 3758 , 6466196 , 2113270 , 3406 , 3730 , 3245728 , 54676537 , 107715 , 22571 , 11852 , 2799 , 2176 , 5392 , 114924 , 1967 , 3503 , 16315 , 6307 , 124663 , 20906 , 2017 , 446816 , 12454 , 67686 , 5153171 , 10798271 , 5576 , 7347 , 7699 , 18573528 , 41684 , 229296 , 2743305 , 121871 , 14868 , 2132993 , 194699 , 1017 , 35455 , 6603901 , 2123 , 16129778 , 2750 , 5074 , 76915 , 11061 , 5917 , 4842 , 683816 , 6603842 , 439201 , 4342 , 115015 , 10404 , 10036135 , 31475 , 10219 , 104762 , 51040 , 104741 , 2466 , 53708 , 1649 , 11967809 , 4487 , 3243567 , 2333 , 1780 , 8210 , 115237 , 16685 , 54675783 , 4456136 , 2264 , 104850 , 1694 , 12620 , 108143 , 25066642 , 3239105 , 3239295]

1584|Q9Y271(37) [2750 , 5405 , 5541 , 4855 , 14385 , 16231 , 2123 , 2247 , 2333 , 16574 , 2478 , 182137 , 6436135 , 123600 , 3055 , 107782 , 5722 , 3406 , 2170 , 4887 , 13765 , 175540 , 11643449 , 2758 , 50942 , 3333 , 17134 , 5198 , 441383 , 119259 , 4912 , 11286230 , 21109 , 39042 , 3117 , 5921 , 2893 , 124087 , 71851 , 2176 , 2913 , 3334 , 3168 , 2732 , 5585 , 11683 , 54677971 , 2132 , 5281040 , 2484 , 1030 , 44112 , 3108 , 21138 , 4107 , 12124 , 14868 , 4156 , 2197 , 10660 , 54676038 , 2812 , 60842 , 2264 , 6913104 , 3686 , 2577 , 1349907 , 2435 , 2391 , 2366 , 5576 , 2753 , 4506 , 6761 , 1057 , 10531 , 5593 , 3559 , 2749 , 35802 , 3516 , 4760 , 10219 , 5510 , 4174 , 4197 , 3151 , 36811 , 11790 , 3478 , 6436123 , 4753 , 2082 , 1981 , 2725 , 2883 , 57469 , 2161 , 3182 , 11508736 , 2467 , 4122 , 3639 , 3324 , 3973 , 1547484 , 2361 , 2754 , 3561 , 16129778 , 1046 , 14052 , 2482 , 6509849 , 3698 , 31072 , 2265 , 4843 , 3748 , 1548942 , 5335 , 131204 , 3435 , 5717 , 4030]

1585|P08246(111) [54686804 , 54677971 , 21109 , 5921 , 2749 , 36811 , 2812 , 1547484 , 2482 , 54678950 , 14052 , 71851 , 3698 , 2132 , 1046 , 3973 , 3151 , 3108 , 2478 , 5198 , 5585 , 2577 , 5593 , 3478 , 3334 , 4843 , 131204 , 2753 , 4156 , 441383 , 2883 , 5510 , 39042 , 54676038 , 3182 , 2484 , 5335 , 2913 , 16129778 , 11286230 , 2893 , 14868 , 175540 , 3324 , 46228924 , 1981 , 35802 , 14385 , 31072 , 124087 , 6761 , 2082 , 4760 , 4197 , 17134 , 3117 , 2758 , 11643449 , 2265 , 2750 , 107706 , 2435 , 5576 , 3686 , 107782 , 44112 , 50942 , 4855 , 5541 , 47499 , 3559 , 24800541 , 1057 , 3406 , 4107 , 11790 , 4753 , 3168 , 13765 , 4122 , 5722 , 10660 , 2732 , 3748 , 16574 , 119259 , 3516 , 4174 , 10324367 , 54735926 , 123600 , 2361 , 2725 , 10531 , 42601552 , 3561 , 21138 , 4912 , 2366 , 4506 , 11683 , 4030 , 3055 , 1030 , 3333 , 2247 , 2391 , 2467 , 2264 , 12124 , 182137 , 1349907 , 5405 , 70683024 , 2754 , 16231 , 2333 , 57469 , 2123 , 65837 , 3435 , 2170 , 2176 , 2197 , 1548942 , 10219 , 3639]

1586|B2RXH2(0) [1694 , 36811 , 665652 , 54675783 , 5722 , 63306 , 2391 , 16188943 , 4380 , 824727 , 2426546 , 3240818 , 11622909 , 2478 , 10168 , 2753 , 4059 , 3092847 , 5153171 , 2310 , 1050 , 1548942 , 3236874 , 3126341 , 11790 , 3237465 , 4027541 , 1322 , 660989 , 3055 , 2855211 , 1878823 , 8395 , 612424 , 3168 , 119259 , 2768954 , 1713166 , 666418 , 4107 , 24144 , 911675 , 3138364 , 245369 , 104838 , 2743305 , 549445 , 327045 , 3333 , 4110197 , 10783 , 2453 , 2122 , 2768974 , 4842 , 3474 , 3746037 , 3865676 , 3781338 , 16190692 , 12555 , 2762 , 3114023 , 1967 , 3503 , 157922 , 10531 , 1649 , 2092 , 3138370 , 2562 , 11289 , 19910 , 68872 , 2540 , 3114022 , 3295 , 3698 , 14052 , 228526 , 193949 , 4197 , 10219 , 4021578 , 2466 , 3648616 , 5585 , 3973 , 3324 , 107992 , 2170 , 8144119 , 104762 , 2739563 , 12492 , 68089 , 2315 , 10235 , 2179 , 3885 , 3277 , 2161 , 175540 , 2307977 , 1066 , 3616 , 16362 , 10651 , 3108 , 32681 , 3647 , 3114024 , 2327 , 2178 , 2876323 , 10206 , 1780 , 2435 , 2355 , 4592 , 126569 , 16231 , 6466196 , 2343 , 54677971 , 36303 , 115015 , 5289501]

1587|P25101(138) [9912992 , 14385 , 4912 , 5510 , 3973 , 2333 , 3406 , 2247 , 3698 , 6918493 , 119259 , 4753 , 2264 , 178103 , 4156 , 4843 , 2482 , 5541 , 3748 , 2913 , 1057 , 2170 , 14052 , 4107 , 216235 , 2577 , 36811 , 44623946 , 17134 , 12124 , 2123 , 44112 , 107782 , 13765 , 4855 , 5585 , 3168 , 16231 , 2361 , 54676038 , 182137 , 4122 , 3559 , 1981 , 104865 , 16129778 , 5593 , 2478 , 3117 , 2366 , 159594 , 3151 , 2883 , 35802 , 10531 , 2176 , 1030 , 2725 , 5198 , 3516 , 2484 , 3108 , 1046 , 3055 , 11683 , 5335 , 16574 , 21138 , 2132 , 123600 , 71851 , 54677971 , 5921 , 2812 , 2732 , 10219 , 2467 , 2750 , 11643449 , 4197 , 5405 , 11790 , 1349907 , 16004692 , 3639 , 50942 , 4506 , 10257882 , 2265 , 1547484 , 443289 , 21109 , 5576 , 10660 , 57469 , 441383 , 2754 , 175540 , 31072 , 5722 , 2758 , 4760 , 124087 , 11286230 , 2749 , 3182 , 9910224 , 3334 , 3324 , 2082 , 2435 , 39042 , 4030 , 3435 , 3333 , 131204 , 14868 , 2893 , 2391 , 1548942 , 2753 , 3561 , 4174 , 6761 , 3686 , 3478 , 177236 , 2197]

1588|O76074(50) [175540 , 17134 , 3151 , 4156 , 2467 , 11465695 , 11286230 , 14868 , 119259 , 5198 , 6761 , 4680 , 50942 , 5318980 , 2197 , 4506 , 4760 , 2132 , 3559 , 3406 , 3639 , 4855 , 1046 , 16574 , 3108 , 10219 , 5335 , 1548942 , 3334 , 5921 , 2754 , 1057 , 1030 , 2753 , 3055 , 2170 , 5593 , 2812 , 3182 , 2478 , 3561 , 3973 , 3686 , 44112 , 2123 , 11683 , 2893 , 2435 , 9892860 , 4174 , 3478 , 2758 , 2577 , 21109 , 54676038 , 447108 , 5585 , 1547484 , 3516 , 3758 , 4107 , 107782 , 3748 , 10660 , 110635 , 2749 , 131204 , 5541 , 2264 , 16960 , 3333 , 19987169 , 123600 , 16129778 , 4912 , 1981 , 11643449 , 3435 , 4843 , 31072 , 182137 , 5212 , 14052 , 36811 , 2391 , 3698 , 4753 , 4030 , 5576 , 2333 , 2725 , 35802 , 10531 , 2883 , 151170 , 124087 , 6918523 , 441383 , 54677971 , 2732 , 11790 , 12124 , 57469 , 21138 , 2176 , 2482 , 13765 , 2366 , 2484 , 2361 , 14385 , 2750 , 3324 , 3671 , 110634 , 4122 , 2265 , 3168 , 2247 , 4197 , 2082 , 5405 , 1349907 , 3117 , 5510 , 16231 , 5722 , 39042 , 2913 , 71851]

1589|P29466(148) [31072 , 16231 , 44112 , 2391 , 2435 , 3108 , 24800541 , 5335 , 2753 , 2197 , 4156 , 5921 , 2333 , 5198 , 124087 , 175540 , 182137 , 3334 , 4843 , 3324 , 3698 , 71851 , 2577 , 1349907 , 5585 , 3333 , 3168 , 2799 , 3516 , 57469 , 3406 , 5510 , 2482 , 2176 , 39042 , 50942 , 12000240 , 2732 , 2758 , 3435 , 11683 , 2247 , 2366 , 4855 , 3055 , 2170 , 3182 , 54677971 , 2361 , 2812 , 4197 , 3561 , 10168 , 2178 , 4912 , 1057 , 16574 , 1547484 , 2893 , 12124 , 10219 , 21109 , 441383 , 2883 , 4506 , 5722 , 42601552 , 54675783 , 2265 , 5593 , 5541 , 2484 , 2132 , 1030 , 2749 , 6761 , 2866904 , 2725 , 4030 , 648831 , 11643449 , 3503 , 4122 , 4760 , 11286230 , 14868 , 1548942 , 119259 , 14052 , 153270 , 4174 , 17134 , 10660 , 36811 , 2750 , 2264 , 13765 , 123600 , 3748 , 3559 , 1046 , 4107 , 16129778 , 2467 , 3117 , 3478 , 2913 , 2123 , 35802 , 24144 , 5576 , 3151 , 3973 , 107782 , 21138 , 131204 , 5405 , 3639 , 5288581 , 1981 , 54676038 , 3686 , 2754 , 2179 , 4753 , 10531 , 11790 , 14385 , 2478 , 2082]

1590|P04035(49) [2725 , 2732 , 3973 , 5312662 , 5510 , 3748 , 50942 , 2170 , 36811 , 3117 , 64715 , 2913 , 107782 , 21138 , 54677971 , 2265 , 14309406 , 2754 , 131204 , 3324 , 446156 , 9867642 , 3639 , 35802 , 1057 , 54687 , 1981 , 12124 , 71851 , 4506 , 1547484 , 446157 , 123600 , 3435 , 1046 , 21109 , 5335 , 2812 , 16574 , 54454 , 53232 , 44112 , 5722 , 2478 , 2577 , 2753 , 4030 , 2082 , 2484 , 2749 , 16231 , 441383 , 5198 , 119259 , 5921 , 2244 , 3698 , 4843 , 124087 , 5585 , 68167 , 4174 , 14868 , 1349907 , 2264 , 3406 , 2758 , 3516 , 10660 , 16129778 , 2750 , 11683 , 5593 , 39042 , 2893 , 14052 , 2482 , 60823 , 13765 , 4122 , 2123 , 2435 , 5405 , 3478 , 2361 , 54684141 , 3151 , 2467 , 3055 , 5576 , 3182 , 4855 , 4197 , 5541 , 3561 , 3108 , 31072 , 2132 , 14385 , 1030 , 2197 , 1662 , 54676038 , 4912 , 3686 , 6761 , 4156 , 10219 , 3334 , 2333 , 4753 , 10531 , 11790 , 11643449 , 175540 , 2883 , 182137 , 2391 , 11286230 , 1548942 , 3168 , 57469 , 4760 , 3333 , 5282452 , 3559 , 4107 , 2176 , 17134 , 2366 , 2247]

1591|P0DMS8(24) [2132 , 3686 , 2197 , 3516 , 5510 , 9953065 , 31072 , 11683 , 2478 , 4156 , 2893 , 10133 , 5722 , 2732 , 182137 , 4855 , 5921 , 2366 , 2753 , 16574 , 2361 , 2391 , 3334 , 124087 , 5198 , 1349907 , 2577 , 35802 , 175540 , 7 , 36811 , 21874557 , 2754 , 5576 , 2333 , 5585 , 3639 , 3324 , 4843 , 2123 , 1057 , 14385 , 1981 , 3478 , 5405 , 5335 , 16231 , 3151 , 14868 , 4753 , 3455 , 3559 , 3748 , 3698 , 3117 , 54676038 , 3973 , 44112 , 131204 , 16129778 , 4107 , 4030 , 2435 , 107782 , 2264 , 2082 , 219024 , 11790 , 123600 , 13765 , 1676 , 2913 , 4760 , 10036135 , 4174 , 10660 , 2170 , 2749 , 5541 , 5593 , 10117987 , 119259 , 2750 , 2883 , 2484 , 14052 , 3108 , 2758 , 3435 , 1548942 , 17134 , 1046 , 4912 , 2812 , 1547484 , 11643449 , 2467 , 4506 , 71851 , 10219 , 10531 , 2265 , 2247 , 21109 , 2176 , 11270783 , 12124 , 16071896 , 1030 , 3561 , 9860294 , 39042 , 21138 , 9896267 , 50942 , 44623946 , 3168 , 3055 , 158795 , 3406 , 441383 , 4122 , 54677971 , 11286230 , 3333 , 57469 , 6761 , 3182 , 2482 , 2725 , 4197]

1592|P13945(94) [4753 , 3779 , 3324 , 39147 , 5405 , 16231 , 107782 , 2913 , 1548942 , 3559 , 5585 , 39468 , 39042 , 124087 , 4156 , 2391 , 3748 , 36811 , 2812 , 2264 , 4843 , 3561 , 2482 , 9865528 , 11286230 , 3698 , 44112 , 31072 , 131204 , 119259 , 3973 , 14385 , 4174 , 1547484 , 2247 , 3435 , 4760 , 2577 , 3117 , 439260 , 4122 , 14052 , 3151 , 175540 , 36920 , 4030 , 3677 , 57469 , 71739 , 3516 , 2749 , 11790 , 5576 , 448991 , 123600 , 5722 , 10531 , 2732 , 2883 , 5541 , 2754 , 2333 , 6761 , 4912 , 2361 , 16129778 , 2123 , 14868 , 3182 , 2176 , 4197 , 50942 , 10219 , 3639 , 838 , 2366 , 2893 , 2478 , 21109 , 2484 , 6439232 , 2467 , 5335 , 54676038 , 71851 , 5510 , 2758 , 1057 , 441383 , 54677971 , 2197 , 3686 , 5593 , 1046 , 10660 , 5921 , 21138 , 2475 , 4946 , 155774 , 1981 , 35802 , 3406 , 4855 , 11683 , 2132 , 3478 , 2082 , 12124 , 4506 , 2435 , 2265 , 3168 , 3108 , 182137 , 16574 , 2585 , 1349907 , 2750 , 11643449 , 1030 , 3035442 , 17134 , 13765 , 2170 , 5198 , 3334 , 2159 , 2753 , 3333 , 2725 , 4107 , 3055]

1593|P25103(56) [1030 , 6451149 , 2170 , 104974 , 2725 , 5198 , 3108 , 1548942 , 3055 , 3516 , 9852175 , 5335 , 21138 , 1046 , 11683 , 10531 , 2132 , 123600 , 71851 , 16574 , 2366 , 2812 , 10219 , 5921 , 2467 , 54677971 , 188927 , 2732 , 2750 , 5585 , 11790 , 3639 , 50942 , 4506 , 108167 , 4197 , 1547484 , 5405 , 1349907 , 21109 , 10660 , 2361 , 57469 , 9917021 , 175540 , 5722 , 4174 , 2754 , 124087 , 219090 , 2749 , 2758 , 4760 , 3334 , 11286230 , 3324 , 2082 , 3182 , 39042 , 3333 , 5576 , 131204 , 2484 , 4030 , 2391 , 3561 , 2893 , 3478 , 2753 , 6761 , 4912 , 219077 , 6918365 , 2333 , 3406 , 2247 , 3698 , 14385 , 3086681 , 3151 , 5510 , 2478 , 3973 , 26757 , 119259 , 4753 , 54676038 , 3686 , 4156 , 4843 , 2482 , 2264 , 2913 , 1057 , 3748 , 5541 , 3435 , 14052 , 4107 , 2577 , 36811 , 107782 , 10328936 , 14868 , 17134 , 2265 , 115237 , 12124 , 31072 , 2123 , 44112 , 6450815 , 16231 , 4855 , 182137 , 6918331 , 177287 , 3168 , 1981 , 4122 , 13765 , 3559 , 16129778 , 10311306 , 5593 , 2197 , 35802 , 11643449 , 2435 , 2176 , 3117 , 2883 , 441383 , 9832383]

1594|Q99700(73) [1234 , 3240461 , 265436 , 3238124 , 361655 , 647884 , 310612 , 265580 , 2725 , 107715 , 6469502 , 10172943 , 6603842 , 6 , 3333 , 2247 , 21749 , 3932 , 16362 , 11293 , 1967 , 12454 , 246835 , 44201498 , 1286501 , 4070375 , 1608140 , 3117 , 19646 , 65758 , 10651 , 10206 , 2799 , 3237705 , 2113270 , 3119467 , 2090 , 3455 , 28688 , 5005498 , 23009 , 3926 , 4116926 , 3151 , 2732927 , 160355 , 327044 , 2927638 , 5187962 , 4777942 , 3168 , 101616 , 6063342 , 1238 , 2562 , 1369 , 2016 , 2179 , 3094465 , 19910 , 2265 , 68363 , 1719874 , 3245163 , 76915 , 1694 , 4342 , 4487 , 3559 , 12449 , 1811924 , 22430825 , 225371 , 2166261 , 26695 , 108144 , 2740698 , 5074 , 21138 , 10104227 , 4454112 , 5405 , 16190984 , 1568843 , 4593 , 13791 , 3503 , 255945 , 24792601 , 3435 , 2938038 , 2794 , 36811 , 24792593 , 2747 , 4122 , 3244425 , 11289 , 3108 , 1392 , 115368 , 16960 , 2229 , 2812 , 3542 , 4031 , 648075 , 2717 , 394347 , 54675783 , 4795607 , 2307977 , 3246767 , 2170 , 10219 , 441383 , 2913 , 1878823 , 4961961 , 3885 , 31593 , 3746037 , 4396341 , 3090880 , 2333 , 16269005 , 28803 , 1720828 , 2466 , 19675 , 44142959 , 45480035 , 3760 , 2165605 , 3404 , 3762 , 4014178]

1595|P14780(502) [2435 , 2132 , 1030 , 131204 , 2482 , 441383 , 2913 , 4912 , 10219 , 44112 , 3108 , 2753 , 6761 , 13765 , 2333 , 2082 , 54677971 , 2754 , 3435 , 5722 , 10660 , 3342298 , 2247 , 44302165 , 119031 , 3561 , 10531 , 4174 , 3559 , 15485452 , 2577 , 3639 , 5405 , 3333 , 2123 , 448002 , 5576 , 175540 , 3334 , 2749 , 36811 , 1046 , 2467 , 21138 , 4122 , 11790 , 2732 , 24751752 , 4753 , 1057 , 2478 , 16231 , 123600 , 1547484 , 5335 , 94413 , 4030 , 39042 , 3324 , 2176 , 4107 , 4506 , 5921 , 14052 , 54676038 , 42601552 , 124087 , 73761 , 3168 , 5541 , 11643449 , 107782 , 2361 , 3182 , 114829 , 5593 , 3698 , 57469 , 50942 , 2197 , 54675783 , 3748 , 128564 , 53317936 , 1981 , 35802 , 151506 , 21109 , 3406 , 16129778 , 2893 , 71851 , 1269845 , 1349907 , 69521 , 5585 , 14385 , 12473 , 12124 , 11683 , 44270901 , 3151 , 4760 , 14868 , 2366 , 4855 , 3478 , 2484 , 2391 , 2883 , 31072 , 2725 , 182137 , 3117 , 3055 , 3686 , 10039403 , 16574 , 2812 , 2758 , 2264 , 3516 , 17134 , 4197 , 119259 , 2265 , 5510 , 4843 , 11286230 , 1066 , 1548942 , 2170 , 5198 , 2750 , 16108938 , 9933197 , 3973 , 4156]

1596|P57059(16) [1548942 , 16129778 , 1981 , 2391 , 3117 , 3698 , 182137 , 2247 , 2123 , 2216 , 54676038 , 115237 , 4122 , 2159 , 3389 , 5510 , 3435 , 1057 , 3748 , 1615 , 2482 , 107782 , 208898 , 3677 , 2478 , 2753 , 39042 , 3561 , 5585 , 14385 , 3334 , 3182 , 3686 , 2170 , 13765 , 5541 , 3973 , 44112 , 6761 , 4174 , 4912 , 175540 , 2913 , 2893 , 2749 , 3478 , 14868 , 4753 , 4843 , 208822 , 36811 , 11643449 , 2577 , 2750 , 2132 , 37632 , 3324 , 131204 , 4030 , 10660 , 5405 , 4156 , 4506 , 50942 , 31072 , 2197 , 2264 , 3055 , 11286230 , 10219 , 3333 , 119570 , 2333 , 21138 , 10531 , 4760 , 2366 , 16574 , 5335 , 3639 , 35802 , 2725 , 2754 , 11954293 , 23897 , 2758 , 40589 , 2082 , 5576 , 2812 , 54677971 , 11683 , 1349907 , 11790 , 57469 , 2883 , 124087 , 3168 , 1547484 , 5722 , 16362 , 4197 , 8966 , 2732 , 12124 , 5593 , 2435 , 1030 , 12454 , 123600 , 2265 , 441383 , 21109 , 2176 , 3559 , 2361 , 71851 , 119828 , 17134 , 4855 , 133621 , 3516 , 197033 , 3108 , 2467 , 2368 , 5921 , 119259 , 16231 , 4107 , 216249 , 1355 , 1046 , 3406 , 2484 , 3151 , 14052 , 38521 , 5198 , 10624]

1597|P25021(16) [42601552 , 4843 , 2247 , 16231 , 11697697 , 54677971 , 182137 , 2913 , 2361 , 4760 , 11643449 , 2750 , 2123 , 2484 , 24745335 , 3435 , 2725 , 2265 , 115237 , 4855 , 14052 , 17134 , 3001055 , 44112 , 1547484 , 124087 , 5722 , 71851 , 10660 , 3033637 , 5335 , 39042 , 2732 , 10624 , 5921 , 107782 , 4156 , 4912 , 2333 , 2264 , 50942 , 2883 , 2159 , 2753 , 2197 , 2170 , 50287 , 175540 , 4174 , 11683 , 2366 , 9976892 , 1981 , 3151 , 4122 , 3389 , 1548942 , 3516 , 3559 , 5510 , 10531 , 1030 , 2749 , 1057 , 2176 , 119570 , 131204 , 17747460 , 2478 , 5541 , 6761 , 2754 , 4107 , 35802 , 4753 , 36811 , 3406 , 54676038 , 3117 , 31072 , 41376 , 4197 , 7741 , 11954293 , 3333 , 21109 , 71768094 , 3055 , 3108 , 3334 , 5593 , 5702160 , 14868 , 21138 , 2482 , 14385 , 119828 , 10219 , 5405 , 3478 , 4097 , 3561 , 5198 , 11286230 , 3324 , 12124 , 2082 , 16129778 , 16574 , 4506 , 3973 , 1046 , 2467 , 1615 , 1349907 , 2577 , 2391 , 2132 , 4030 , 2756 , 2893 , 11790 , 123600 , 44623946 , 3168 , 2812 , 1548992 , 441383 , 5576 , 5585 , 3748 , 25070031 , 3698 , 13765 , 2758 , 65895 , 3686 , 119259 , 57469 , 3639 , 3182 , 2435]

1598|Q92731(186) [4197 , 179337 , 5284643 , 11683 , 11286230 , 10660 , 5722 , 3182 , 5510 , 2247 , 2082 , 9912639 , 5991 , 656952 , 656936 , 1349907 , 12124 , 4506 , 104741 , 3561 , 5576 , 2484 , 14868 , 10219 , 1547484 , 2754 , 3686 , 21109 , 11643449 , 13765 , 2482 , 4122 , 4760 , 192197 , 1548942 , 175540 , 2366 , 2577 , 16734800 , 14052 , 5756 , 119259 , 2478 , 6102690 , 2732 , 1030 , 50942 , 5870 , 2913 , 656953 , 2361 , 441383 , 5035 , 5280961 , 3435 , 3055 , 124087 , 4912 , 2733526 , 2327 , 131204 , 2725 , 14385 , 11368987 , 2264 , 2758 , 3151 , 3478 , 10286462 , 3168 , 123600 , 107782 , 6761 , 39042 , 2123 , 44112 , 31072 , 4107 , 2333 , 3639 , 1046 , 3406 , 1057 , 1981 , 4855 , 2753 , 3698 , 5921 , 35802 , 2812 , 4030 , 4843 , 2197 , 2265 , 5198 , 21138 , 182137 , 3559 , 154257 , 5757 , 2893 , 567825 , 71851 , 683816 , 2176 , 54676038 , 3108 , 4174 , 3973 , 5335 , 3333 , 5405 , 2749 , 57469 , 2132 , 16574 , 36811 , 16750040 , 16129778 , 4156 , 54677971 , 2750 , 2467 , 3117 , 448537 , 5585 , 10531 , 3748 , 216416 , 4753 , 17134 , 11790 , 3334 , 5541 , 3516 , 2883 , 11149479 , 5593 , 16231 , 3324 , 2170 , 2435 , 2391 , 6102691]

1599|P41145(25) [2159 , 3435 , 57469 , 50942 , 3559 , 2197 , 5488548 , 4156 , 128563 , 13765 , 4197 , 5585 , 1547484 , 2913 , 2725 , 14868 , 1057 , 3108 , 5510 , 2361 , 441383 , 16574 , 4855 , 2082 , 175540 , 5284570 , 11286230 , 2132 , 3698 , 11790 , 2482 , 4058 , 21138 , 44129648 , 3151 , 179340 , 3406 , 3686 , 6761 , 2123 , 5593 , 5462508 , 131204 , 3748 , 3561 , 1349907 , 54676038 , 2758 , 44112 , 119259 , 21109 , 2478 , 5576 , 5335 , 11683 , 66553195 , 2749 , 4107 , 2247 , 441278 , 3333 , 3334 , 17134 , 5405 , 5284594 , 2750 , 1046 , 60768 , 5360515 , 2333 , 5311304 , 2754 , 3182 , 115237 , 1548942 , 10100 , 16129778 , 5198 , 4174 , 3478 , 3055 , 4030 , 182137 , 119828 , 115208 , 4843 , 2435 , 2264 , 1030 , 10101 , 36811 , 10219 , 5361918 , 10660 , 2176 , 6445230 , 2893 , 107782 , 2577 , 4912 , 2467 , 16231 , 4122 , 5288826 , 123600 , 2391 , 3345 , 2265 , 31072 , 3973 , 3516 , 3168 , 44623946 , 5722 , 4753 , 2366 , 2484 , 35802 , 1981 , 14385 , 3117 , 5284569 , 14052 , 124087 , 5361092 , 39042 , 5284596 , 2812 , 13493 , 5284595 , 2753 , 22267 , 644073 , 5541 , 54677971 , 71851 , 3324 , 3639 , 11643449 , 2883 , 2732 , 5359421 , 4506 , 12124 , 5921 , 2170 , 10531 , 4760]

1600|P41143(16) [4843 , 3345 , 2577 , 10101 , 4174 , 10668 , 2435 , 2264 , 3055 , 182137 , 36811 , 5405 , 2176 , 31072 , 5361918 , 3973 , 4912 , 2893 , 5288826 , 4030 , 11790 , 2265 , 16362 , 10531 , 24762741 , 5510 , 2484 , 16231 , 3168 , 123600 , 5284569 , 1981 , 2391 , 2366 , 124087 , 2750 , 5361092 , 3455 , 4122 , 5722 , 11683 , 3516 , 4753 , 3955 , 14052 , 35802 , 3117 , 54677971 , 44112 , 13493 , 39042 , 5359421 , 10219 , 5284596 , 22267 , 71851 , 3324 , 3639 , 11643449 , 5284595 , 2753 , 5541 , 2883 , 13505 , 5462508 , 2732 , 2159 , 5921 , 3435 , 57469 , 5576 , 6761 , 2467 , 4506 , 50942 , 2197 , 5488548 , 4156 , 10624 , 4760 , 5198 , 14868 , 3559 , 441383 , 16574 , 14385 , 4855 , 1057 , 13765 , 4197 , 11286230 , 12124 , 2361 , 5585 , 2913 , 2132 , 2812 , 2482 , 2725 , 4058 , 175540 , 44129648 , 2082 , 5284603 , 2123 , 2170 , 3406 , 5284570 , 1547484 , 3698 , 5593 , 21138 , 3108 , 3151 , 5284371 , 119259 , 3748 , 1349907 , 54676038 , 2478 , 2758 , 5335 , 131204 , 441278 , 3561 , 17134 , 5284594 , 1046 , 5311304 , 3334 , 2754 , 4107 , 107782 , 10660 , 10100 , 66553195 , 2749 , 119570 , 21109 , 2247 , 5360515 , 3333 , 3686 , 3182 , 3478 , 1548942 , 1030 , 16129778 , 2333]

1601|P25100(13) [2123 , 148842 , 4855 , 16231 , 182137 , 3559 , 5593 , 216249 , 11286230 , 3117 , 1547484 , 3334 , 164089 , 4184 , 3151 , 5576 , 39042 , 3677 , 4030 , 2176 , 38521 , 131204 , 2818 , 2391 , 2753 , 1030 , 3561 , 2159 , 2725 , 3168 , 5722 , 5510 , 3033538 , 2265 , 164739 , 2333 , 2484 , 2247 , 3108 , 11683 , 5335 , 3686 , 2478 , 16574 , 439260 , 60602 , 21138 , 119259 , 4753 , 2132 , 123600 , 54677971 , 2368 , 72106 , 4843 , 2732 , 5541 , 10219 , 2750 , 2913 , 11643449 , 4636 , 3435 , 14052 , 5405 , 1349907 , 1057 , 2577 , 5585 , 21109 , 10660 , 2812 , 17134 , 12124 , 5312125 , 44112 , 107782 , 4760 , 2754 , 5265 , 175540 , 13765 , 31072 , 4174 , 2758 , 124087 , 33625 , 4122 , 2749 , 1981 , 16129778 , 3182 , 3324 , 2082 , 4893 , 3822 , 3333 , 2361 , 2883 , 35802 , 14868 , 2893 , 1548942 , 6761 , 441383 , 4197 , 5074 , 3478 , 3157 , 6082 , 14385 , 4912 , 5073 , 3406 , 3973 , 5198 , 3698 , 3516 , 5816 , 3869 , 208898 , 1046 , 3055 , 10531 , 2435 , 2264 , 5775 , 2366 , 4156 , 2092 , 2482 , 13542 , 71851 , 5401 , 3748 , 54676038 , 119570 , 5921 , 2170 , 5268 , 4107 , 2467 , 2197 , 129211 , 36811 , 11790 , 3639 , 57469 , 50942 , 4506]

1602|P10636(83) [11286230 , 6624620 , 1829960 , 24239 , 101744 , 6063342 , 11790 , 2758 , 15474019 , 107715 , 648831 , 2732 , 4912 , 3151004 , 1780 , 2913 , 68186 , 1967 , 4592 , 16362 , 1810986 , 21501 , 50287 , 2435 , 2179 , 5282060 , 2247 , 3116068 , 54675783 , 2950 , 5593 , 115015 , 10168 , 19910 , 11061 , 2855211 , 547914 , 3245131 , 1322 , 114924 , 6472251 , 2392140 , 36303 , 24867796 , 3034012 , 78933 , 1896320 , 3244194 , 2265 , 219269 , 115150 , 4362 , 10235 , 89105 , 3237465 , 2169358 , 911675 , 10114 , 3686 , 1548942 , 2147557 , 3885 , 3610 , 2812 , 3108 , 6301 , 3117 , 100016 , 1811924 , 16490 , 4122 , 3478 , 3082 , 10230 , 3277 , 2315667 , 265580 , 3455 , 3435 , 11289 , 2197 , 5917 , 3244425 , 5185709 , 107782 , 44112 , 10206 , 6603842 , 26937 , 14878 , 1993 , 2333 , 5722 , 2743305 , 100095 , 4760 , 104826 , 361939 , 3316 , 2894446 , 1066 , 3138364 , 5480 , 12492 , 3503 , 296597 , 5153171 , 141870 , 50248 , 1649 , 2768975 , 2750 , 10382715 , 14868 , 1694 , 3698 , 72139 , 2799 , 4380 , 10219 , 76915 , 4278 , 1568843 , 949760 , 8269 , 4343310 , 5074 , 1878823 , 123409 , 13791 , 5405 , 3168 , 36811 , 122623 , 5510 , 8609 , 2396 , 446816 , 3138370 , 3138330 , 612424 , 2327 , 3242381 , 3138375 , 361655 , 5475 , 41684 , 10245972 , 6603901 , 10531]

1603|Q01959(74) [54677971 , 57469 , 4156 , 5722 , 119828 , 182137 , 2482 , 2391 , 2247 , 3182 , 2801 , 101616 , 17134 , 2159 , 2913 , 4107 , 4528 , 10531 , 11790 , 4197 , 10624 , 2577 , 11643449 , 65650 , 4912 , 11597698 , 2758 , 2361 , 2750 , 444 , 2435 , 2753 , 3639 , 3561 , 2366 , 2264 , 124087 , 3748 , 1981 , 9860294 , 119570 , 14868 , 54676038 , 2812 , 25070031 , 4174 , 14052 , 54841 , 2467 , 11430856 , 2754 , 2749 , 16574 , 2265 , 115237 , 2170 , 1057 , 2484 , 3516 , 5585 , 5584 , 3435 , 1547484 , 3151 , 5921 , 4506 , 11622909 , 3406 , 2176 , 5541 , 1030 , 6917779 , 4753 , 3168 , 1349907 , 2893 , 16129778 , 3686 , 5335 , 2197 , 11286230 , 3455 , 13765 , 3675 , 107782 , 6761 , 4760 , 4855 , 4158 , 3389 , 3404 , 40589 , 4020 , 21138 , 5210 , 4843 , 16231 , 125017 , 1614 , 10660 , 1046 , 175540 , 2732 , 5198 , 10219 , 3973 , 71851 , 119259 , 154101 , 5593 , 39042 , 36811 , 3117 , 5826 , 1615 , 2333 , 11683 , 2137 , 2478 , 5510 , 50942 , 3334 , 131204 , 5576 , 3324 , 3698 , 2082 , 21109 , 2725 , 14584 , 2883 , 3108 , 123600 , 3559 , 3055 , 35802 , 441383 , 2132 , 31072 , 4122 , 13542 , 44112 , 12124 , 3333 , 3478 , 14385 , 1548942 , 2123 , 4030 , 10048368 , 44623946 , 5405]

1604|P22303(94) [2131 , 119259 , 6761 , 2264 , 2082 , 3559 , 3561 , 4156 , 3324 , 3334 , 10660 , 2132 , 2265 , 4843 , 17754078 , 2176 , 8179 , 1046 , 76915 , 3748 , 175540 , 3108 , 3686 , 2577 , 1548942 , 1935 , 12124 , 39042 , 4991 , 16739244 , 5335 , 5405 , 2812 , 5198 , 5576 , 5593 , 13005 , 5722 , 31072 , 2482 , 2123 , 11286230 , 14052 , 57469 , 3698 , 4760 , 182137 , 3333 , 107782 , 3973 , 2361 , 42601552 , 3202 , 2435 , 2170 , 11790 , 4506 , 4753 , 36811 , 3182 , 4855 , 123600 , 10902085 , 2732 , 124087 , 2467 , 14868 , 2197 , 5282338 , 35802 , 138508 , 44112 , 4456 , 2366 , 2484 , 11643449 , 2893 , 2883 , 1547484 , 2753 , 4912 , 3168 , 50942 , 3117 , 54676038 , 4122 , 3152 , 26257 , 2478 , 5510 , 10879668 , 21138 , 3639 , 62539 , 131204 , 237515 , 104850 , 3516 , 1030 , 4107 , 78057 , 441383 , 10531 , 10219 , 2750 , 3055 , 1057 , 2333 , 2725 , 11683 , 54677971 , 4197 , 3435 , 187 , 5921 , 4030 , 5936 , 13765 , 77991 , 10198924 , 9651 , 11747 , 1349907 , 5541 , 3151 , 21109 , 4004 , 3321360 , 39793 , 14385 , 2758 , 3478 , 16129778 , 16574 , 79690 , 656986 , 16231 , 1981 , 2391 , 2754 , 3406 , 71851 , 2913 , 2749 , 5585 , 5420 , 5983 , 198752 , 4174 , 17134 , 446506 , 2247 , 132228]

1605|P41597(168) [2366 , 2132 , 2123 , 4855 , 2435 , 3168 , 3108 , 3117 , 1547484 , 3639 , 5585 , 12124 , 2082 , 2484 , 11790 , 4122 , 2758 , 4760 , 2467 , 11151929 , 53320968 , 3334 , 41214 , 6761 , 2176 , 35802 , 53317028 , 2883 , 11651747 , 119259 , 5510 , 4174 , 3698 , 441383 , 5541 , 10219 , 36811 , 5593 , 53326225 , 5921 , 2732 , 11286230 , 39042 , 3324 , 53320973 , 16129778 , 53323664 , 53317041 , 2361 , 175540 , 11643449 , 2478 , 2482 , 1349907 , 2749 , 2577 , 4197 , 4156 , 16007088 , 4030 , 46208367 , 3435 , 2391 , 3973 , 14052 , 3333 , 21138 , 2170 , 3151 , 53323652 , 123600 , 13765 , 2812 , 11151928 , 2146 , 3516 , 4843 , 3748 , 11370332 , 53326226 , 53322299 , 3406 , 11254190 , 3686 , 131204 , 16231 , 2197 , 53323651 , 2265 , 5405 , 53320970 , 4506 , 21109 , 5335 , 11173161 , 2893 , 3182 , 124087 , 5576 , 71851 , 2725 , 3055 , 1030 , 11322108 , 5198 , 4912 , 31072 , 2333 , 2247 , 53326901 , 3561 , 53323665 , 53318313 , 1046 , 2264 , 11390777 , 11218678 , 54677971 , 50942 , 11683 , 53322298 , 14385 , 53317027 , 11609693 , 11735999 , 182137 , 4753 , 1981 , 1548942 , 44112 , 3478 , 53318314 , 16574 , 53320967 , 3559 , 53324941 , 53320349 , 2913 , 57469 , 54676038 , 2750 , 107782 , 1057 , 17134 , 10531 , 11437477 , 11544064 , 14868 , 53320974 , 5722 , 53326242 , 2753 , 10660 , 2754 , 4107]

1606|P08588(65) [6761 , 3973 , 115237 , 4631 , 11504295 , 11286230 , 208898 , 131204 , 50942 , 4156 , 1355 , 44623946 , 39042 , 3677 , 5405 , 2265 , 39468 , 1057 , 2482 , 146294 , 1615 , 5253 , 3748 , 16231 , 3324 , 1548942 , 2750 , 9860294 , 3182 , 14385 , 3478 , 5585 , 2247 , 14052 , 4828 , 10660 , 2159 , 3869 , 5198 , 2170 , 3686 , 17134 , 3334 , 2119 , 59768 , 2467 , 6918554 , 2369 , 119259 , 1349907 , 11683 , 182137 , 3389 , 4932 , 16129778 , 2361 , 2082 , 1978 , 2132 , 2249 , 2435 , 2391 , 1981 , 2732 , 4197 , 3559 , 3108 , 3516 , 441383 , 5722 , 4107 , 155774 , 123600 , 46937143 , 1030 , 2475 , 3151 , 11790 , 2725 , 12124 , 3406 , 14868 , 60657 , 2405 , 21138 , 57469 , 9865528 , 54677971 , 5593 , 3055 , 5311064 , 71301 , 2758 , 10219 , 71851 , 2176 , 5335 , 16574 , 2366 , 3561 , 36811 , 16739244 , 1547484 , 3168 , 119570 , 838 , 2883 , 2484 , 10531 , 11954293 , 2754 , 3762 , 2123 , 3639 , 4506 , 2333 , 11643449 , 5541 , 5576 , 124087 , 4843 , 4171 , 175540 , 21109 , 5921 , 35802 , 2812 , 4912 , 3779 , 4883 , 2753 , 3083544 , 2577 , 4760 , 31477 , 107782 , 1046 , 71768094 , 2478 , 39147 , 4753 , 4030 , 2749 , 2893 , 42396 , 2197 , 4855 , 31072 , 5510 , 4174 , 44112 , 4122 , 2585 , 54676038 , 13765 , 3333 , 2913 , 3435 , 3698 , 3117 , 2264]

1607|P06241(38) [16722836 , 5005498 , 4174 , 124087 , 10296883 , 11608401 , 2265 , 2170 , 2132 , 14052 , 16122633 , 6761 , 4912 , 2247 , 2750 , 21138 , 24779724 , 1349907 , 5576 , 36811 , 2366 , 57469 , 3478 , 2812 , 123600 , 2123 , 3151 , 15983966 , 4855 , 107782 , 2577 , 448008 , 2482 , 5405 , 2758 , 11485656 , 10427712 , 3038522 , 3639 , 156414 , 4107 , 76098 , 3686 , 8189 , 208908 , 11712649 , 1057 , 10113978 , 11427553 , 1030 , 131204 , 176870 , 5541 , 31072 , 2264 , 3062316 , 14385 , 11338033 , 39042 , 11667893 , 3748 , 24889392 , 5921 , 10531 , 10127622 , 3055 , 5198 , 11314340 , 4156 , 2725 , 3516 , 2749 , 35802 , 6918454 , 2484 , 4122 , 16574 , 2732 , 3561 , 153999 , 5585 , 176167 , 3324 , 16231 , 71851 , 2435 , 3406 , 2391 , 5593 , 1046 , 4506 , 2361 , 11643449 , 2478 , 21109 , 3333 , 13765 , 4760 , 4197 , 14868 , 11234052 , 2754 , 10660 , 447077 , 10074640 , 5722 , 54677971 , 1981 , 182137 , 2197 , 151194 , 9549303 , 16129778 , 447966 , 2396 , 441383 , 1548942 , 11409972 , 6419766 , 4030 , 5330286 , 3182 , 3973 , 12124 , 5335 , 11683 , 3698 , 2333 , 3168 , 3435 , 2883 , 11364421 , 4843 , 2176 , 1547484 , 44112 , 11656518 , 4753 , 160355 , 50942 , 3334 , 5510 , 3025986 , 10219 , 2082 , 2856 , 11790 , 17755052 , 54676038 , 11213558 , 2467 , 2893 , 119259 , 3117 , 2913 , 17134 , 3559 , 2753 , 3108 , 175540 , 11286230]

1608|P31645(151) [3182 , 54841 , 124087 , 2753 , 115237 , 1030 , 3386 , 35802 , 13765 , 2170 , 3435 , 3455 , 9966051 , 6917779 , 441383 , 21109 , 3108 , 4156 , 2159 , 2883 , 3639 , 3559 , 10624 , 5585 , 34869 , 6761 , 2132 , 54677971 , 5736 , 2577 , 3117 , 16129778 , 11683 , 1349907 , 14385 , 40589 , 2801 , 1981 , 4122 , 4976 , 3478 , 4174 , 5576 , 5324346 , 13542 , 14868 , 9860294 , 2754 , 2749 , 4030 , 2467 , 2123 , 3404 , 1615 , 14052 , 3151 , 6918314 , 182137 , 54676038 , 11790 , 2484 , 4753 , 2082 , 5405 , 2750 , 16574 , 4197 , 1547484 , 36811 , 2725 , 2435 , 2333 , 119570 , 2478 , 5584 , 3516 , 119259 , 3168 , 11286230 , 5541 , 1548942 , 4107 , 2160 , 2771 , 68617 , 5921 , 2482 , 11643449 , 50942 , 2391 , 2265 , 4912 , 23573 , 3324 , 125017 , 2361 , 5198 , 2366 , 10660 , 119828 , 2197 , 5335 , 57469 , 3698 , 5210 , 10219 , 4843 , 12124 , 3675 , 71768094 , 2247 , 5593 , 60854 , 5722 , 2893 , 3561 , 44112 , 3055 , 9884876 , 16362 , 3333 , 43815 , 4760 , 3389 , 3696 , 71851 , 1355 , 101616 , 5510 , 3686 , 4855 , 65650 , 21138 , 2812 , 10531 , 175540 , 3406 , 2732 , 2758 , 16231 , 146570 , 123600 , 1614 , 4506 , 1057 , 4449 , 3334 , 60835 , 1046 , 131204 , 5656 , 107782 , 17134 , 2913 , 3748 , 4543 , 39042 , 2264 , 3947 , 2995 , 31072 , 44623946 , 2176 , 11622909 , 3973]

1609|P08173(2) [49381 , 2159 , 4934 , 36811 , 208824 , 1273944 , 2478 , 4912 , 187 , 2370 , 3108 , 5335 , 4843 , 2753 , 131204 , 2361 , 44112 , 5585 , 10531 , 2381 , 123600 , 442021 , 3406 , 24199 , 71851 , 2913 , 4506 , 2883 , 11790 , 2366 , 15376 , 107782 , 2170 , 37632 , 3478 , 2197 , 16960 , 10219 , 3494 , 9860294 , 44259 , 50942 , 1349907 , 2082 , 10090005 , 124087 , 4855 , 2160 , 1615 , 2732 , 2758 , 3324 , 2230 , 2812 , 5405 , 11643449 , 4629 , 6761 , 1981 , 5921 , 55752 , 3168 , 4122 , 2754 , 9577995 , 182137 , 5593 , 2123 , 5198 , 17134 , 3182 , 115237 , 3334 , 2176 , 2435 , 2448 , 2577 , 1046 , 9571002 , 50905989 , 2342 , 21138 , 40589 , 44419370 , 2247 , 5510 , 3698 , 11286230 , 21109 , 50906191 , 4848 , 54676038 , 4030 , 3151 , 2484 , 1548942 , 4753 , 39042 , 4197 , 1547484 , 10938 , 2391 , 5541 , 5576 , 14052 , 2725 , 16065403 , 2551 , 16231 , 4107 , 13765 , 5910 , 3516 , 2265 , 16574 , 4156 , 31072 , 3973 , 3055 , 2333 , 119259 , 2749 , 5722 , 57469 , 107867 , 54677971 , 3561 , 4174 , 3686 , 2893 , 3748 , 71203 , 1057 , 2482 , 3559 , 10660 , 3117 , 2750 , 2229 , 441071 , 1993 , 16129778 , 3435 , 14868 , 444031 , 4760 , 2264 , 1030 , 441383 , 175540 , 11683 , 3639 , 12124 , 14385 , 50906192 , 174174 , 3389 , 3333 , 35802 , 119570 , 71183 , 5440 , 2132 , 60809 , 2467]

1610|P35354(484) [4037 , 4760 , 4197 , 2082 , 3698 , 2264 , 4753 , 3825 , 2913 , 3973 , 3033 , 2478 , 2366 , 134780 , 12124 , 3406 , 54676038 , 10219 , 2758 , 60542 , 5722 , 3435 , 2170 , 14052 , 123600 , 2176 , 208925 , 3151 , 16574 , 5405 , 4044 , 4506 , 3059 , 123619 , 3826 , 3324 , 21138 , 1983 , 1548942 , 5090 , 131204 , 35802 , 50942 , 159271 , 11683 , 151166 , 175540 , 3561 , 4912 , 2132 , 2197 , 2883 , 208910 , 54677470 , 2750 , 2732 , 3108 , 3055 , 2333 , 68723 , 4843 , 119828 , 3333 , 3182 , 3686 , 3394 , 14868 , 17134 , 4614 , 124087 , 3117 , 2361 , 107782 , 57469 , 2753 , 5936 , 2467 , 39042 , 2662 , 1057 , 10660 , 2247 , 3559 , 5468 , 1046 , 4855 , 78363 , 2482 , 16682734 , 4107 , 182137 , 2581 , 16231 , 2342 , 1030 , 1396 , 5585 , 119607 , 3168 , 3308 , 19910 , 2391 , 11286230 , 2724 , 213053 , 3516 , 441383 , 5198 , 54677971 , 11790 , 2265 , 68752 , 2244 , 5509 , 21109 , 16741227 , 156391 , 2484 , 5576 , 5335 , 3748 , 151075 , 2749 , 36811 , 1547484 , 4781 , 31072 , 4174 , 4488 , 2754 , 5593 , 1349907 , 2123 , 2577 , 71851 , 3478 , 11643449 , 1981 , 3334 , 5359 , 4030 , 4409 , 6761 , 2812 , 9832687 , 5510 , 2435 , 10531 , 4495 , 4122 , 3639 , 13765 , 119259 , 54676228 , 14385 , 11508736 , 5541 , 33675 , 4156 , 3672 , 16129778 , 2893 , 2725 , 5921 , 4493 , 44112]

1611|P05177(120) [1057 , 3433 , 2883 , 35802 , 52919 , 2264 , 2082 , 2366 , 2749 , 1548942 , 1030 , 3561 , 3748 , 2484 , 11286230 , 14868 , 2893 , 158781 , 643477 , 3973 , 441383 , 3108 , 21138 , 46883536 , 10660 , 3117 , 104850 , 4197 , 4174 , 6761 , 5510 , 2132 , 10368812 , 14052 , 2176 , 17754438 , 5722 , 11552706 , 2753 , 4760 , 3406 , 71851 , 15506 , 5541 , 21109 , 119259 , 156419 , 2750 , 11790 , 11508736 , 17134 , 3516 , 11234052 , 4912 , 11488320 , 124087 , 4030 , 16574 , 36811 , 11634973 , 11643449 , 31072 , 11683 , 50942 , 16960 , 39042 , 16220188 , 11406590 , 2758 , 2812 , 24800541 , 4855 , 4506 , 10114 , 3474 , 11154925 , 3324 , 4843 , 56846693 , 16362 , 3333 , 2333 , 3334 , 2170 , 12124 , 16071896 , 3686 , 5335 , 44578433 , 2197 , 51049968 , 12813 , 59823 , 5921 , 3404 , 5585 , 54677971 , 3435 , 2123 , 10531 , 3168 , 2247 , 1046 , 3478 , 5405 , 4107 , 2179 , 19987169 , 4156 , 11285588 , 3151 , 16231 , 175540 , 107782 , 2361 , 16739650 , 3055 , 4493 , 10253143 , 71768094 , 11526696 , 3698 , 2482 , 2725 , 2391 , 46216796 , 3182 , 16363 , 14385 , 54676537 , 2913 , 16129778 , 11292933 , 4122 , 2182 , 5198 , 2577 , 182137 , 10219 , 5593 , 2754 , 44112 , 154104 , 4753 , 3639 , 123600 , 3559 , 56950369 , 2435 , 54897 , 1547484 , 46885626 , 131204 , 5576 , 2467 , 47318 , 57469 , 1981 , 20629114 , 13765 , 54676038 , 2478 , 148201 , 12756 , 2732 , 1349907 , 2265]

1612|Q573B4(0) [10296883 , 11286230 , 2725 , 11552706 , 3151 , 71851 , 3559 , 11364421 , 2247 , 44112 , 76098 , 2082 , 16574 , 11656518 , 15991573 , 4197 , 6761 , 10113978 , 4506 , 11314340 , 10427712 , 3698 , 3324 , 2482 , 3334 , 2750 , 5326739 , 11234052 , 2577 , 2170 , 5921 , 1547484 , 153999 , 21109 , 448008 , 54677971 , 5722 , 1030 , 2396 , 2123 , 10267580 , 16231 , 3748 , 3025986 , 3686 , 2754 , 2391 , 24826799 , 5405 , 2812 , 11643449 , 2856 , 24779724 , 14385 , 2265 , 11790 , 4753 , 31072 , 6419766 , 2893 , 2753 , 1057 , 2333 , 4855 , 11608401 , 175540 , 2467 , 4912 , 182137 , 3117 , 5576 , 57379345 , 4122 , 208908 , 3182 , 14052 , 2366 , 11213558 , 2484 , 2197 , 3333 , 10660 , 2732 , 10074640 , 4760 , 151194 , 3168 , 2758 , 2435 , 4843 , 10302451 , 156414 , 123600 , 1981 , 39042 , 11683 , 2883 , 1046 , 124087 , 15983966 , 6918454 , 2176 , 16722836 , 5585 , 35802 , 10531 , 3435 , 16129778 , 176870 , 3516 , 11712649 , 2749 , 9549303 , 24889392 , 3639 , 11442891 , 131204 , 11667893 , 11427553 , 54676038 , 119259 , 13765 , 4107 , 1694 , 5541 , 57469 , 5510 , 16122633 , 4174 , 107782 , 1349907 , 5198 , 3973 , 2913 , 3561 , 11338033 , 3108 , 5335 , 4156 , 11485656 , 176167 , 2264 , 2478 , 3055 , 2132 , 447966 , 5005498 , 4030 , 3406 , 3038522 , 2361 , 21138 , 17755052 , 10127622 , 50942 , 10219 , 14868 , 17134 , 1548942 , 11409972 , 3478 , 36811 , 12124 , 160355 , 5593 , 441383 , 447077]

1613|P17948(177) [1030 , 2856 , 3334 , 11338033 , 3561 , 17755052 , 5541 , 25031915 , 10127622 , 160355 , 182137 , 151194 , 3038522 , 11790 , 4753 , 4156 , 10275001 , 3055 , 5005498 , 11234052 , 2396 , 4122 , 3516 , 24889392 , 5405 , 10427712 , 3151 , 3182 , 5576 , 11485656 , 11683 , 2750 , 2123 , 448008 , 2758 , 39042 , 3478 , 1349907 , 14868 , 2361 , 5921 , 2482 , 13765 , 11667893 , 153999 , 208908 , 447077 , 4843 , 11973736 , 14052 , 4912 , 10660 , 176870 , 35802 , 3406 , 16129778 , 1046 , 2264 , 2478 , 6918454 , 4030 , 10302451 , 11286230 , 3435 , 9911830 , 2435 , 16231 , 119259 , 124087 , 3698 , 2082 , 16722836 , 9549295 , 4174 , 3025986 , 2753 , 5198 , 6419766 , 11409972 , 2484 , 5585 , 2754 , 15983966 , 3639 , 9868037 , 5593 , 24767976 , 2265 , 24779724 , 16574 , 14385 , 3168 , 2749 , 2812 , 21138 , 107782 , 2467 , 44112 , 176167 , 5335 , 2725 , 12124 , 9933475 , 5722 , 10074640 , 3748 , 71851 , 1981 , 2893 , 3117 , 1057 , 5510 , 3324 , 21109 , 2366 , 36811 , 1547484 , 11213558 , 4506 , 441383 , 123600 , 2170 , 156414 , 9809715 , 2247 , 10113978 , 50942 , 3973 , 131204 , 3559 , 11714580 , 11364421 , 17134 , 2913 , 42642645 , 2732 , 175540 , 11314340 , 4855 , 216239 , 11427553 , 57469 , 11643449 , 54677971 , 10219 , 11167602 , 11751922 , 4107 , 2577 , 31072 , 10531 , 4197 , 2197 , 6761 , 2132 , 4760 , 11656518 , 54676038 , 2176 , 3333 , 3108 , 2391 , 2333 , 11712649 , 1548942 , 447966 , 9549303 , 3686 , 2883]

1614|P35372(93) [3334 , 16574 , 10531 , 124087 , 3455 , 2435 , 3748 , 131204 , 57469 , 3333 , 5284595 , 35802 , 39042 , 1057 , 2361 , 2750 , 1349907 , 21138 , 4197 , 5359272 , 1981 , 4855 , 10660 , 4753 , 5361092 , 5284594 , 14385 , 5510 , 11643449 , 41693 , 2333 , 3698 , 3406 , 10219 , 6761 , 2159 , 10517 , 5284371 , 123600 , 2366 , 4122 , 5405 , 2893 , 10100 , 14868 , 3516 , 44112 , 2725 , 441278 , 5921 , 115237 , 2265 , 4095 , 10668 , 2082 , 13493 , 2264 , 2482 , 3324 , 4760 , 2749 , 2758 , 5593 , 36811 , 4058 , 5284596 , 5585 , 11683 , 31072 , 4107 , 2484 , 4174 , 3168 , 119570 , 3973 , 2883 , 175540 , 3435 , 2170 , 10101 , 5198 , 2247 , 66553195 , 60815 , 3117 , 13505 , 3955 , 4843 , 3478 , 21109 , 51263 , 4506 , 50942 , 3686 , 13765 , 3055 , 5576 , 2732 , 107782 , 5488548 , 17134 , 2467 , 3345 , 4912 , 5361918 , 2812 , 22267 , 16129778 , 24737629 , 2753 , 44129648 , 2577 , 71851 , 2132 , 10624 , 1046 , 2754 , 131534 , 182137 , 4030 , 14052 , 16231 , 33741 , 3108 , 5284604 , 15130 , 2913 , 68938 , 3182 , 1030 , 11250029 , 5288826 , 8944 , 5541 , 4156 , 441383 , 119828 , 56959087 , 9838022 , 3639 , 10064061 , 119259 , 11790 , 5722 , 2176 , 3151 , 5311304 , 5359371 , 644073 , 3559 , 1548942 , 16362 , 1547484 , 3561 , 2391 , 54676038 , 5360515 , 5335 , 5284569 , 5284570 , 2123 , 2478 , 5462508 , 12124 , 2197 , 54677971 , 11286230 , 44623946 , 5359421]

1615|P50406(6) [3108 , 4156 , 4855 , 5358 , 11643449 , 2749 , 2132 , 1547484 , 2893 , 10219 , 5541 , 3478 , 44112 , 49381 , 50942 , 119828 , 2484 , 3698 , 3324 , 5585 , 16574 , 2123 , 2725 , 12124 , 3406 , 3055 , 2264 , 115237 , 4506 , 5452 , 54676038 , 4174 , 2812 , 3435 , 4107 , 2883 , 35802 , 5198 , 124087 , 3559 , 2265 , 2753 , 60809 , 2170 , 1057 , 13765 , 2758 , 16106 , 2732 , 9805719 , 14052 , 4184 , 3561 , 4843 , 68848 , 10624 , 4753 , 3372 , 36811 , 3748 , 21109 , 11683 , 31072 , 10531 , 11430856 , 1981 , 42601552 , 16071605 , 16129778 , 119570 , 11292933 , 2361 , 107782 , 4760 , 1548942 , 3334 , 10660 , 2750 , 123600 , 5722 , 9860294 , 2391 , 39042 , 2176 , 1355 , 1349907 , 16231 , 4122 , 1030 , 197706 , 3151 , 3964 , 28693 , 5405 , 17134 , 1238 , 31101 , 5335 , 1046 , 2366 , 5921 , 2333 , 3168 , 2754 , 3389 , 3973 , 5074 , 3686 , 131204 , 14868 , 16362 , 2159 , 54677971 , 11286230 , 6761 , 6089 , 2082 , 4106 , 197033 , 2913 , 71851 , 2160 , 2435 , 2467 , 4748 , 3333 , 2818 , 2478 , 5576 , 175540 , 57469 , 5073 , 28864 , 5761 , 12454 , 4585 , 10090005 , 11961293 , 71768094 , 4197 , 2577 , 2247 , 182137 , 2197 , 21138 , 5736 , 3516 , 8223 , 23897 , 14385 , 5593 , 119259 , 4030 , 9966051 , 11256720 , 60835 , 3182 , 44623946 , 3639 , 11790 , 2482 , 3005573 , 5510 , 71360 , 441383 , 55752 , 1615 , 2726 , 11954293 , 3117 , 4912]

1616|P23975(57) [5722 , 25070031 , 5002 , 11430856 , 2754 , 4158 , 4174 , 65856 , 2478 , 5576 , 2749 , 16574 , 2893 , 1614 , 3151 , 3404 , 449193 , 3698 , 65650 , 2732 , 11643449 , 1547484 , 5736 , 2750 , 2435 , 36811 , 2176 , 2995 , 2771 , 11683 , 1349907 , 119259 , 3168 , 6917779 , 119828 , 2812 , 4543 , 14868 , 1355 , 2467 , 2577 , 5541 , 101616 , 4976 , 6761 , 2265 , 2913 , 1057 , 5510 , 4528 , 50942 , 3334 , 11790 , 125017 , 21138 , 5585 , 3435 , 4197 , 2883 , 10531 , 16231 , 3055 , 35802 , 21109 , 3333 , 2368 , 2361 , 1046 , 3639 , 10660 , 34869 , 3696 , 444 , 2484 , 12124 , 54677971 , 5584 , 2753 , 23573 , 1981 , 5593 , 4912 , 9860294 , 39042 , 182137 , 3455 , 5335 , 54841 , 3947 , 54676038 , 31072 , 2082 , 16129778 , 175540 , 2391 , 3108 , 123600 , 441383 , 4855 , 3516 , 17134 , 2159 , 4843 , 5656 , 3973 , 2160 , 4500 , 5210 , 38521 , 2801 , 3478 , 5921 , 2366 , 5198 , 14385 , 4506 , 2123 , 10219 , 57469 , 4753 , 3686 , 4156 , 44623946 , 4760 , 2482 , 3561 , 2333 , 11286230 , 13765 , 4020 , 3675 , 9838022 , 119570 , 2197 , 107782 , 2132 , 131204 , 2170 , 3182 , 3748 , 3324 , 2725 , 71768094 , 3117 , 4107 , 3389 , 2247 , 44112 , 3559 , 40589 , 2758 , 4011 , 10624 , 5405 , 1030 , 4122 , 42601552 , 13542 , 4449 , 4030 , 2264 , 71851 , 124087 , 11622909 , 60835 , 1548942 , 3406 , 14052 , 5826 , 1615 , 115237]

1617|P21917(31) [1349907 , 2812 , 2577 , 4122 , 54676038 , 2467 , 2478 , 16363 , 2366 , 16574 , 688272 , 71851 , 2818 , 3686 , 124087 , 2391 , 10775317 , 4452 , 16231 , 21109 , 10039198 , 36811 , 2197 , 31101 , 14385 , 14868 , 2750 , 10705550 , 3341 , 2484 , 2758 , 219050 , 54746 , 3639 , 123600 , 5593 , 10531 , 2753 , 5330286 , 1238 , 443951 , 3117 , 115237 , 2435 , 4850 , 47811 , 3334 , 60149 , 37459 , 681 , 49381 , 44112 , 4174 , 3333 , 12124 , 10667966 , 13765 , 1030 , 5265 , 2749 , 6761 , 2482 , 4107 , 21138 , 107782 , 2159 , 2893 , 50942 , 3964 , 10219 , 2726 , 6005 , 5566 , 4420454 , 3406 , 59227 , 441383 , 2448 , 184841 , 182137 , 2732 , 4506 , 119828 , 3561 , 2754 , 9860294 , 2333 , 5440 , 11286230 , 10824155 , 10420539 , 54677971 , 16 , 2725 , 5541 , 11954293 , 3168 , 2247 , 4912 , 1981 , 2176 , 10660 , 4030 , 28864 , 4855 , 39042 , 3478 , 119259 , 5198 , 2913 , 3151 , 1547484 , 3516 , 10116877 , 17134 , 3055 , 131204 , 11643449 , 4917 , 5722 , 16362 , 3748 , 2265 , 5585 , 5576 , 16106 , 1046 , 133079 , 119570 , 2170 , 5510 , 31072 , 11683 , 6918248 , 2361 , 3973 , 16129778 , 57469 , 5921 , 5736 , 2264 , 3324 , 3389 , 4753 , 11790 , 68950 , 4156 , 3435 , 2123 , 3698 , 10624 , 4197 , 2883 , 2082 , 1615 , 5405 , 14052 , 5335 , 1548942 , 3108 , 4760 , 57267 , 5074 , 3182 , 4843 , 115368 , 35802 , 2132 , 54562 , 1057 , 175540 , 3559 , 23897]

1618|P11473(346) [1811924 , 3245163 , 11061 , 161562 , 5288670 , 288875 , 612424 , 3559 , 124087 , 6918508 , 16315 , 17931 , 31957 , 10212 , 2540 , 3117 , 3080557 , 2333 , 3698 , 37175 , 122077 , 16269005 , 3420746 , 27812 , 115368 , 3762 , 13789 , 25644 , 5281107 , 101616 , 3746037 , 20055510 , 14899645 , 1057 , 2170 , 3237439 , 25102723 , 5283734 , 1967 , 2950 , 11350 , 65790 , 115150 , 3503 , 2812 , 549445 , 1322 , 32681 , 28803 , 3082 , 2090 , 240112 , 24776445 , 7352 , 2396 , 2161 , 10382715 , 6603901 , 2450 , 2194 , 18573632 , 3237465 , 3286 , 6194 , 13986 , 12713 , 121871 , 2743305 , 71905 , 11967809 , 3109 , 7916 , 115015 , 6764 , 2453 , 16347 , 5074 , 3120949 , 10868 , 5392 , 5480 , 2330 , 20906 , 24144 , 6063342 , 1046 , 12449 , 31200 , 18104 , 4278 , 104850 , 3885 , 24361 , 16362 , 3760 , 6 , 3138375 , 649156 , 16837 , 10672195 , 219081 , 108143 , 2179 , 6915835 , 911675 , 11088 , 19996 , 3032279 , 14868 , 115157 , 5288783 , 7347 , 20055424 , 6237 , 41684 , 15474019 , 2017 , 31475 , 11683 , 17434 , 18573525 , 3610 , 1694 , 14190 , 6982 , 10168 , 1052 , 10404 , 1781 , 542364 , 3002119 , 114924 , 8189 , 3686 , 16590 , 2327 , 11852 , 194699 , 122623 , 2799 , 10114 , 19910 , 72139 , 5282060 , 3244425 , 15286 , 13521 , 5281104 , 10172943 , 53708 , 13113 , 1238 , 2794 , 76915 , 2435 , 132862 , 4680274 , 446313 , 3415217 , 36303 , 3969 , 3277 , 11006 , 10245972 , 35455 , 39299 , 5289549 , 10041070 , 16490 , 47472 , 1369 , 3561 , 2893 , 5280453]

1619|Q504U8(0) [2754 , 4506 , 1046 , 11488320 , 3973 , 3025986 , 17134 , 71851 , 11620908 , 5585 , 35802 , 3038522 , 11442891 , 11667893 , 11511120 , 2333 , 14385 , 31072 , 11338033 , 2396 , 2758 , 151194 , 16722836 , 5198 , 119259 , 14052 , 10297043 , 9549303 , 5335 , 447966 , 6419766 , 176870 , 2467 , 3505109 , 5921 , 10127622 , 4912 , 156414 , 10296883 , 1981 , 176167 , 3478 , 2366 , 3698 , 3516 , 10401956 , 441383 , 3108 , 1547484 , 44112 , 5329106 , 2856 , 17755052 , 15983966 , 21138 , 11712649 , 2132 , 3686 , 12124 , 2264 , 3559 , 5722 , 3324 , 6761 , 123600 , 39042 , 11213558 , 36811 , 5510 , 2753 , 10302451 , 24794418 , 2883 , 2123 , 54677971 , 54676038 , 2577 , 2478 , 11640390 , 10660 , 3055 , 107782 , 10427712 , 2484 , 50942 , 2749 , 16574 , 6918454 , 131204 , 14868 , 3639 , 2750 , 2170 , 2247 , 57469 , 4174 , 2435 , 11643449 , 11552706 , 10531 , 5005498 , 21109 , 447077 , 153999 , 448008 , 5593 , 16129778 , 2361 , 2913 , 24779724 , 2265 , 3151 , 2732 , 4107 , 5405 , 5541 , 5576 , 1548942 , 1030 , 4030 , 3406 , 3435 , 11790 , 4753 , 208908 , 11409972 , 4122 , 24889392 , 11485656 , 11683 , 4760 , 13765 , 175540 , 3117 , 3333 , 10219 , 2391 , 11608401 , 76098 , 2197 , 3334 , 2176 , 22024915 , 11286230 , 2482 , 2893 , 2725 , 3182 , 10113978 , 2812 , 46937083 , 1349907 , 4843 , 182137 , 11656518 , 4708 , 1057 , 3748 , 124087 , 2082 , 160355 , 11314340 , 11427553 , 11364421 , 16122633 , 4855 , 3168 , 4156 , 16231 , 11234052 , 3561 , 10074640 , 57379345 , 4197]

1620|P41595(22) [5011 , 3406 , 5198 , 131204 , 16231 , 62065 , 5074 , 11954293 , 5405 , 31072 , 2893 , 21109 , 5576 , 5335 , 1548942 , 2176 , 124087 , 2725 , 4199 , 71851 , 1030 , 3055 , 4912 , 1046 , 2264 , 2333 , 2247 , 54677971 , 71202 , 8226 , 1614 , 107992 , 11683 , 28693 , 4843 , 14385 , 68848 , 11292933 , 11961293 , 50942 , 31101 , 16574 , 4753 , 3478 , 60809 , 12124 , 3559 , 5510 , 57469 , 1355 , 54676038 , 2750 , 5362436 , 5533 , 896 , 107782 , 1547484 , 2913 , 68186 , 17134 , 10531 , 3333 , 3561 , 177336 , 14868 , 1057 , 2753 , 115237 , 119828 , 219050 , 2754 , 4107 , 3639 , 2197 , 5722 , 2366 , 2123 , 10660 , 4855 , 2435 , 2132 , 54746 , 3168 , 21138 , 27400 , 11430856 , 82148 , 8969 , 2265 , 4184 , 3108 , 3117 , 3973 , 2484 , 5585 , 4122 , 4760 , 2818 , 4850 , 1615 , 2082 , 47811 , 54562 , 3822 , 2467 , 6761 , 44623946 , 2732 , 2883 , 3334 , 441383 , 35802 , 114709 , 2482 , 11790 , 5265 , 36811 , 5593 , 4174 , 3698 , 28864 , 11658860 , 5921 , 6005 , 5541 , 10219 , 11604525 , 4828 , 11286230 , 9805719 , 443951 , 119259 , 119570 , 16129778 , 4506 , 8223 , 2361 , 39042 , 448400 , 3324 , 1349907 , 10624 , 2577 , 182137 , 4197 , 175540 , 2758 , 11643449 , 4106 , 2478 , 2749 , 4030 , 3435 , 4156 , 2159 , 14052 , 5160 , 5568 , 71781 , 2170 , 3151 , 2391 , 1150 , 13765 , 3516 , 5311271 , 3748 , 123600 , 5268 , 2812 , 3386 , 44112 , 3182 , 1981 , 3686]

1621|P07550(200) [146294 , 2577 , 11790 , 3410 , 2749 , 3442589 , 1547484 , 119259 , 4753 , 4030 , 1057 , 2482 , 14385 , 9860294 , 10531 , 124087 , 2265 , 13765 , 155774 , 39147 , 16129778 , 71739 , 9892481 , 17134 , 6761 , 107782 , 4156 , 2753 , 5253 , 36811 , 9865528 , 39042 , 31072 , 39468 , 4946 , 5510 , 35330 , 6918554 , 2197 , 2725 , 4107 , 31729 , 11954293 , 1234 , 2893 , 115237 , 4843 , 3117 , 12124 , 2170 , 2783 , 2361 , 44112 , 3055 , 11683 , 3108 , 5405 , 2159 , 3561 , 5585 , 3435 , 2083 , 19910 , 3168 , 5722 , 2369 , 35802 , 2176 , 2132 , 50942 , 5403 , 4828 , 3516 , 1355 , 16231 , 1030 , 16574 , 5198 , 1981 , 16739244 , 119570 , 4506 , 54676038 , 1046 , 2249 , 3973 , 3748 , 32051 , 2475 , 2264 , 4171 , 3389 , 175540 , 3151 , 3559 , 2758 , 1349907 , 9294 , 5335 , 14052 , 5541 , 2366 , 2883 , 4845 , 4932 , 2750 , 2391 , 4760 , 33624 , 2467 , 5593 , 3639 , 2333 , 2435 , 11643449 , 2754 , 3677 , 4916 , 10184665 , 3406 , 44623946 , 3343 , 11505444 , 5152 , 10660 , 3779 , 57469 , 11504295 , 2913 , 131204 , 4086 , 6917655 , 1615 , 54677971 , 4122 , 10219 , 11286230 , 4197 , 123600 , 14868 , 441383 , 2585 , 31477 , 3698 , 54766 , 63952 , 3333 , 5921 , 2082 , 37990 , 2247 , 182137 , 21138 , 4174 , 1548942 , 3478 , 5816 , 3762 , 2478 , 5576 , 21109 , 2123 , 4912 , 3334 , 3869 , 3182 , 71851 , 4855 , 2119 , 3083544 , 2484 , 3324 , 2812 , 71768094 , 3686 , 2732]

1622|P20309(71) [10938 , 5576 , 2883 , 11519070 , 2448 , 4848 , 119570 , 4197 , 11683 , 2732 , 50942 , 4634 , 4107 , 443879 , 36811 , 3561 , 174174 , 441383 , 2342 , 14868 , 9577995 , 2484 , 2753 , 1030 , 2750 , 10090005 , 2749 , 2551 , 2265 , 44419370 , 3748 , 3334 , 2725 , 5510 , 107867 , 16231 , 16362 , 2247 , 3333 , 2435 , 21109 , 12124 , 5440 , 4174 , 2176 , 5593 , 3494 , 44112 , 5541 , 187 , 115237 , 442021 , 3435 , 6126 , 154059 , 4753 , 31072 , 182137 , 5198 , 16129778 , 54676038 , 9571002 , 2467 , 2754 , 11434515 , 5314 , 2082 , 2913 , 2577 , 2160 , 444031 , 5585 , 16960 , 441071 , 5910 , 50906192 , 2361 , 55752 , 4629 , 2132 , 119259 , 14052 , 2478 , 10660 , 39042 , 5921 , 71203 , 2812 , 2229 , 6761 , 3108 , 21867154 , 3686 , 3324 , 11643449 , 1547484 , 3389 , 1349907 , 1057 , 2159 , 2366 , 3478 , 11790 , 5722 , 49381 , 107782 , 3168 , 5335 , 2170 , 3973 , 35802 , 5405 , 37632 , 3182 , 3042 , 17134 , 15376 , 2230 , 71851 , 124087 , 131204 , 5487427 , 54677971 , 11286230 , 3559 , 2758 , 3117 , 10219 , 1046 , 4760 , 1981 , 4122 , 10531 , 16065403 , 23897 , 21138 , 16574 , 2893 , 14385 , 175540 , 1548942 , 2391 , 57469 , 3055 , 24199 , 2370 , 4855 , 4506 , 2197 , 50906191 , 3406 , 13765 , 40589 , 3698 , 9860294 , 4843 , 60809 , 50905989 , 1993 , 4156 , 4912 , 71183 , 3639 , 4934 , 4030 , 2123 , 3151 , 44623946 , 3516 , 2784 , 2381 , 123600 , 1615 , 2482 , 2264 , 2333]

1623|P43220(51) [2795 , 3241177 , 16363 , 16157882 , 45480035 , 19675 , 3236724 , 327045 , 17113 , 38531 , 2081 , 131411 , 16188984 , 187 , 50248 , 2799 , 12454 , 11289 , 11310 , 2220273 , 2466 , 3442589 , 2132 , 16574 , 3245025 , 166553 , 10172943 , 801418 , 1580955 , 11104 , 22530 , 2294842 , 68943 , 3828 , 3758 , 16231 , 3127904 , 3125446 , 65800 , 3760 , 13505 , 192197 , 11954283 , 5918 , 16187479 , 2762 , 588415 , 16347 , 3286 , 1719873 , 68634 , 2315 , 13450 , 3002820 , 31729 , 441383 , 5359646 , 3671 , 288875 , 204105 , 37175 , 446816 , 67686 , 3334 , 119570 , 108144 , 310612 , 3245385 , 2812 , 26695 , 5335 , 265580 , 2229 , 249 , 123606 , 115368 , 44278361 , 193949 , 3734228 , 6301 , 76915 , 1552036 , 6470206 , 3003803 , 2361 , 547914 , 11643449 , 2179 , 614669 , 2998359 , 4197 , 24792593 , 2016 , 5510 , 660989 , 1878823 , 98514 , 3333 , 24792601 , 24466 , 1599306 , 16191546 , 1937568 , 55483 , 16362 , 2137779 , 68684 , 16639 , 13789 , 5074 , 660708 , 3245402 , 1608140 , 268472 , 4655877 , 3351 , 1432578 , 175540 , 1811924 , 661085 , 12124 , 68363 , 21138 , 1234 , 10206 , 680935 , 5585 , 2082 , 2983151 , 10651 , 45480040 , 808378 , 3094465 , 1720828 , 19646 , 72462 , 101744 , 7329 , 6063342 , 9551522 , 5389584 , 3168 , 2453 , 327044 , 1568843 , 1993 , 10212 , 2359114 , 151506 , 42725 , 2247 , 2132993 , 4619 , 10517 , 18573524 , 19910 , 394347 , 5723 , 581148 , 6 , 53708 , 12555 , 3940466 , 16190941 , 776717 , 7475368 , 178144 , 3542 , 3746037 , 68089 , 2315667 , 5005498 , 12449 , 3559 , 2090 , 19003 , 8395]

1624|O94782(15) [14868 , 3433 , 3610 , 68089 , 31072 , 3316 , 23897 , 6603842 , 3326 , 2092 , 14878 , 2333 , 2396 , 19646 , 3117 , 11289 , 7572 , 21102 , 5381 , 1392 , 3936 , 35375 , 3005573 , 193949 , 6603901 , 6708773 , 2750 , 156419 , 114811 , 10206 , 10651 , 115244 , 1967 , 1892 , 27448 , 159977 , 16362 , 11046239 , 5531 , 61574 , 175540 , 2883 , 5392 , 14759 , 68363 , 5063962 , 3926 , 13791 , 2318 , 2893 , 68186 , 248271 , 15443 , 1046 , 107751 , 4890 , 17931 , 2446 , 19910 , 20906 , 11967809 , 10718 , 3759 , 243274 , 50259 , 5282060 , 361655 , 10235 , 4380 , 108143 , 71645 , 104850 , 10219 , 3969 , 2366 , 192197 , 47472 , 22571 , 7329 , 2724 , 1548942 , 3828 , 3435 , 2377 , 5074 , 3312 , 2794 , 11286230 , 3607 , 31475 , 11412540 , 3515 , 31060 , 2090 , 27812 , 3474 , 2170 , 104741 , 4670 , 2017 , 107867 , 11683 , 26533 , 2161 , 3333 , 2200 , 67686 , 3503 , 4487 , 4122 , 1549789 , 21138 , 4031 , 2540 , 122081 , 3277 , 11104 , 41109 , 71874 , 3885 , 2179 , 43231 , 2194 , 3108 , 3736 , 24107 , 4006 , 14899645 , 8798 , 3741 , 50248 , 122077 , 8138 , 9363 , 32170 , 10831 , 51040 , 10212 , 196122 , 3698 , 2812 , 13986 , 54676038 , 11296583 , 16837 , 10046567 , 16351 , 114924 , 6764 , 6237 , 68909 , 2576 , 21414 , 1238 , 5405 , 2330 , 2799 , 115015 , 194699 , 10531 , 2484 , 7191 , 1547484 , 2315 , 2247 , 5289501 , 666418 , 107715 , 18104 , 65909 , 1694 , 13505 , 94280 , 16490 , 3760 , 5510 , 13916 , 8609 , 656641 , 56463 , 13765 , 10114 , 3455]

1625|P04150(213) [4506 , 3503 , 17134 , 5289501 , 3333 , 2082 , 2366 , 5921 , 21109 , 2812 , 16923 , 153909 , 2391 , 11006 , 14052 , 3324 , 44112 , 10219 , 1057 , 182137 , 12589 , 2735009 , 3182 , 3559 , 3435 , 2132 , 247839 , 444036 , 5281004 , 3698 , 1981 , 31072 , 10133 , 6741 , 16129778 , 4107 , 16490 , 2247 , 2123 , 6 , 8196 , 28803 , 3973 , 2577 , 3108 , 9865442 , 2333 , 9576789 , 5198 , 2749 , 4843 , 2725 , 54677971 , 13765 , 2893 , 4912 , 31307 , 4156 , 3516 , 41684 , 11660 , 5865 , 14868 , 2197 , 123600 , 2478 , 28594 , 443936 , 4753 , 7903 , 15433 , 107782 , 4760 , 5311505 , 57469 , 4174 , 1548942 , 11683 , 5335 , 10168 , 2264 , 1547484 , 3117 , 9642 , 16574 , 5754 , 32798 , 1046 , 3478 , 2758 , 6545 , 3406 , 2176 , 16734800 , 4122 , 2435 , 5755 , 124087 , 12532 , 2170 , 6761 , 61186 , 3686 , 131204 , 25644 , 2482 , 5405 , 11286230 , 5576 , 7347 , 3748 , 7329 , 11852 , 6505 , 5743 , 6215 , 16231 , 71851 , 3034285 , 6436 , 3639 , 6918155 , 9782 , 2750 , 2484 , 5510 , 55245 , 3082 , 111332 , 4030 , 11643449 , 3168 , 36811 , 441383 , 21138 , 5585 , 71414 , 50942 , 21805 , 2883 , 10531 , 2754 , 1030 , 31253 , 31236 , 3334 , 119259 , 10660 , 5311067 , 115157 , 2467 , 2732 , 3151 , 11293 , 8907 , 441335 , 84098 , 9878 , 3055 , 35802 , 175540 , 2753 , 39042 , 12302 , 1349907 , 54676038 , 82153 , 2265 , 121752 , 443958 , 14385 , 5593 , 2913 , 2361 , 5541 , 3118 , 3561 , 4855 , 4197 , 114924 , 19996 , 12329 , 444025 , 12124 , 11790 , 5722]

1626|P21728(41) [17676 , 4506 , 14385 , 5311360 , 1981 , 11286230 , 4843 , 16 , 2265 , 2264 , 4197 , 31101 , 4107 , 5335 , 4912 , 123600 , 3404 , 3973 , 54746 , 2725 , 1057 , 2818 , 3686 , 10783 , 2749 , 3406 , 31729 , 175540 , 107715 , 52919 , 3435 , 50942 , 5593 , 1649 , 441383 , 6603842 , 2170 , 1030 , 53708 , 4760 , 5198 , 5452 , 11954293 , 1614 , 1238 , 1548942 , 4174 , 2758 , 9860294 , 11643449 , 4814 , 3639 , 28864 , 5921 , 3341 , 2732 , 71851 , 2750 , 361655 , 5281881 , 3561 , 6077 , 11683 , 182137 , 10624 , 5722 , 119828 , 14052 , 35802 , 3389 , 3117 , 16129778 , 4917 , 3748 , 60820 , 8226 , 2484 , 5265 , 2478 , 3333 , 44623946 , 10219 , 6005 , 59227 , 14899645 , 131204 , 2435 , 2159 , 107930 , 1349907 , 2883 , 681 , 5585 , 124087 , 4855 , 5510 , 3334 , 115237 , 2726 , 107782 , 2123 , 5541 , 16231 , 42601552 , 2176 , 5405 , 1547484 , 39042 , 16574 , 13765 , 3559 , 3108 , 1355 , 3932 , 57469 , 3372 , 2081 , 3516 , 2482 , 2812 , 2893 , 36811 , 31072 , 4753 , 3055 , 10660 , 2132 , 11790 , 2333 , 133621 , 12124 , 4156 , 10531 , 14868 , 2467 , 3885 , 2753 , 3698 , 3478 , 5074 , 21138 , 28688 , 4592 , 3151 , 4030 , 208820 , 1615 , 2082 , 18104 , 16739244 , 3822 , 49381 , 4122 , 23897 , 2247 , 16362 , 54676038 , 5440 , 2366 , 119570 , 2754 , 54677971 , 21109 , 6761 , 3324 , 16960 , 11154555 , 119259 , 1046 , 17134 , 37459 , 2197 , 2577 , 5576 , 68950 , 44112 , 12454 , 2913 , 3182 , 6603901 , 47811 , 3503 , 2391 , 4593 , 3168 , 2361]

1627|P16473(93) [15394 , 3239339 , 8124 , 4814 , 28803 , 666418 , 2478 , 2137 , 208820 , 26695 , 3671 , 2725 , 3655 , 3455 , 3236383 , 3559 , 123600 , 41684 , 25644 , 2159 , 1066 , 954161 , 2812 , 3245385 , 2133505 , 175540 , 81530 , 10423 , 101616 , 3973 , 6605037 , 3245025 , 2355 , 2754 , 160355 , 2753 , 2576 , 2053712 , 5921 , 4843 , 7915 , 1649 , 10651 , 3333 , 28688 , 2717 , 26596 , 3760 , 655287 , 2562 , 53708 , 4031 , 228244 , 3245163 , 3672772 , 16316 , 4197 , 3245451 , 3830 , 36811 , 3758 , 11790 , 19266 , 47472 , 665087 , 1989 , 4380 , 5335 , 10382715 , 2216 , 104762 , 144457 , 11293 , 6598 , 3034012 , 22530 , 1993 , 21138 , 2170 , 1238 , 647884 , 2365 , 2090 , 6605027 , 16362 , 170344 , 8041 , 2391 , 660883 , 133621 , 2762 , 3117 , 6605039 , 3244425 , 2930983 , 249 , 3326 , 31253 , 2333 , 2836838 , 2419371 , 5593 , 4278 , 1861634 , 361655 , 1815815 , 3244430 , 3610 , 12968 , 3245931 , 3969 , 2950 , 3238134 , 2758 , 4493 , 10114 , 6077 , 68872 , 2315 , 2750 , 8872 , 7819 , 54676038 , 25914 , 26533 , 2179 , 107715 , 7352 , 61247 , 2482 , 2277 , 61410 , 3243710 , 5723 , 2200 , 3478 , 2166261 , 25134246 , 2913 , 652355 , 6605024 , 2396 , 647201 , 55918 , 4122 , 1046 , 31475 , 4578 , 665652 , 19996 , 10168 , 648831 , 2132 , 1701 , 547914 , 3237649 , 11 , 3542 , 11245 , 3002119 , 2377 , 10036135 , 4156 , 28594 , 16231 , 3108 , 37175 , 20686 , 5074 , 10237 , 441383 , 16637 , 1234 , 18510 , 14242 , 1967 , 2343 , 3242198 , 8095 , 3239387 , 115244 , 4761 , 11289 , 101744 , 645503 , 6540 , 3125057 , 25670]

1628|P28335(38) [3964 , 5736 , 60809 , 2467 , 1355 , 2732 , 8226 , 2082 , 2482 , 124087 , 1349907 , 2264 , 115237 , 3334 , 3168 , 3389 , 3324 , 4585 , 107992 , 5566 , 8223 , 448400 , 3973 , 2132 , 28864 , 1615 , 5335 , 2577 , 16574 , 2197 , 54562 , 60795 , 3406 , 441383 , 10219 , 3516 , 11292933 , 82148 , 2726 , 119570 , 23897 , 4636 , 1238 , 5533 , 2812 , 5160 , 5073 , 5011 , 4184 , 31072 , 3108 , 2435 , 39042 , 5265 , 28693 , 14052 , 71768094 , 4855 , 71851 , 9860294 , 4122 , 44112 , 107782 , 55752 , 2750 , 5576 , 5722 , 2247 , 16362 , 2754 , 11954293 , 3435 , 5452 , 4030 , 1548942 , 17134 , 10660 , 71781 , 219050 , 4760 , 60854 , 3822 , 10624 , 4912 , 68848 , 21138 , 14868 , 12124 , 33630 , 4156 , 11604525 , 2758 , 5198 , 2883 , 5593 , 11683 , 4106 , 2366 , 2893 , 2484 , 1046 , 1150 , 54746 , 2333 , 4506 , 2159 , 68186 , 2391 , 57469 , 4174 , 10531 , 5074 , 2170 , 2749 , 131204 , 60835 , 54677971 , 3478 , 123600 , 4843 , 71202 , 5311271 , 119259 , 6761 , 1030 , 4107 , 3748 , 16129778 , 443951 , 54676038 , 62065 , 2361 , 1057 , 2913 , 35802 , 2753 , 4753 , 13765 , 11790 , 11643449 , 11430856 , 4449 , 4748 , 4197 , 175540 , 36811 , 5541 , 60149 , 16231 , 3686 , 4205 , 2123 , 1547484 , 21109 , 9805719 , 5585 , 2478 , 3182 , 197033 , 47811 , 182137 , 5405 , 3117 , 5761 , 3639 , 3698 , 14385 , 5921 , 2818 , 9966051 , 2176 , 11286230 , 11961293 , 3561 , 3333 , 3151 , 50942 , 6005 , 5440 , 62865 , 3559 , 3055 , 11658860 , 27400 , 2725 , 2265 , 5510 , 11683556 , 31101 , 1981]

1629|Q96QE3(10) [3781338 , 1720828 , 16187479 , 24978701 , 1238 , 4380 , 2286863 , 3108 , 3117 , 2888937 , 3973 , 2914644 , 660708 , 2724 , 2768975 , 4097 , 16362 , 24791741 , 1701 , 954161 , 1599306 , 3420746 , 2265 , 539709 , 647038 , 4163388 , 44602029 , 4961961 , 3455 , 680935 , 542364 , 162834 , 2831167 , 107715 , 8041 , 76915 , 54677971 , 4843 , 3334 , 65758 , 3708374 , 44144252 , 208820 , 3503 , 2179 , 4487 , 1046 , 886096 , 16574 , 3542 , 228526 , 10219 , 55918 , 4777950 , 1967 , 31729 , 11683 , 70846 , 4578 , 2913 , 647201 , 2090 , 3156709 , 10258 , 3813687 , 24817214 , 3326 , 2812 , 4564 , 53708 , 911675 , 10832 , 4493 , 10767 , 2291046 , 3151 , 44142242 , 10531 , 2827330 , 14369 , 1614257 , 2435 , 3237465 , 3168 , 348986 , 28803 , 3240818 , 1432578 , 4983363 , 6 , 1937568 , 2197 , 1392 , 133621 , 68363 , 14604 , 5585 , 4122 , 100472 , 265580 , 1863658 , 18573632 , 268472 , 4278 , 288875 , 6603842 , 1811924 , 4593 , 1694 , 19646 , 17113 , 3236874 , 1568843 , 1608140 , 820311 , 756673 , 5405 , 100095 , 5074 , 2291103 , 21109 , 3926 , 2082 , 7699 , 1878823 , 5917 , 4103738 , 3842920 , 18573526 , 4343310 , 160355 , 549445 , 5459650 , 5804 , 24817194 , 16269005 , 2914308 , 44201975 , 2234553 , 327045 , 24792593 , 3377088 , 3433 , 3698 , 4366092 , 3238134 , 2359114 , 5510 , 3139316 , 1985 , 7475448 , 3559 , 16129778 , 3236724 , 4404908 , 3246760 , 1552036 , 194595 , 1580955 , 3237655 , 2277 , 16306185 , 3748 , 2999850 , 750895 , 3351 , 3230434 , 42725 , 703905 , 2753 , 16190945 , 3940466 , 660989 , 3138330 , 4261 , 3095276 , 361655 , 1745499 , 3746037 , 3238726 , 4622 , 12454 , 3094465 , 104741 , 2855211 , 16059888 , 3474 , 3333 , 5289501 , 655083 , 3295 , 2482 , 2997734 , 3730 , 824727 , 19910 , 3885]

1630|Q16539(188) [3324 , 2391 , 17755052 , 5405 , 11314340 , 3151 , 46937120 , 16722836 , 2082 , 2176 , 2435 , 9871074 , 5171 , 2333 , 4912 , 10297982 , 2366 , 4174 , 11364421 , 160355 , 16129778 , 5326869 , 4506 , 11427553 , 17134 , 54676038 , 3698 , 57469 , 16574 , 11338033 , 5287728 , 3406 , 2478 , 10531 , 2484 , 5326871 , 3435 , 2893 , 2132 , 21138 , 2749 , 208908 , 25174101 , 3561 , 11790 , 12124 , 24963046 , 176167 , 6419766 , 10275001 , 3108 , 5541 , 3973 , 2725 , 11667893 , 11608401 , 10341154 , 441383 , 4369443 , 2812 , 35802 , 15991573 , 131204 , 16220188 , 31072 , 446816 , 50942 , 76098 , 16231 , 2170 , 5005498 , 2577 , 2264 , 5326868 , 3334 , 1349907 , 4122 , 1057 , 1030 , 5327066 , 2396 , 4760 , 5510 , 42647299 , 447721 , 447077 , 1694 , 3078519 , 10113978 , 2361 , 2467 , 9865587 , 2123 , 448008 , 1547484 , 3168 , 119259 , 3542 , 1046 , 10074640 , 3478 , 10409068 , 156414 , 107782 , 3540 , 3686 , 11234052 , 2265 , 6761 , 3516 , 11643449 , 11485656 , 10296883 , 10172943 , 22049997 , 24779724 , 4156 , 176870 , 14385 , 12106168 , 5921 , 5172 , 36811 , 5164 , 182137 , 3038522 , 11683 , 6918454 , 4030 , 10427712 , 3025986 , 5576 , 15983966 , 11656518 , 39042 , 24889392 , 2352168 , 44112 , 5198 , 5289514 , 5593 , 2754 , 1981 , 153999 , 3639 , 54677971 , 4843 , 11712649 , 175540 , 11406590 , 4855 , 3333 , 2883 , 10127622 , 3008319 , 3055 , 11442891 , 21109 , 3748 , 1548942 , 24941253 , 9549303 , 3182 , 447966 , 124087 , 71851 , 5722 , 46883775 , 2753 , 5326866 , 11373432 , 151194 , 2482 , 2247 , 11409972 , 4107 , 4753 , 10219 , 3117 , 10660 , 2856 , 14052 , 16122633 , 2197 , 5282440 , 129236 , 11213558 , 5326870 , 4197 , 5335 , 2750 , 2913 , 5585 , 11286230 , 13765 , 11552706 , 2758 , 2732 , 3559 , 123600 , 11714580 , 14868]

1631|P35367(24) [39042 , 2482 , 2444 , 131204 , 2726 , 2267 , 14868 , 1057 , 23897 , 2732 , 4506 , 3241 , 10531 , 2818 , 3686 , 3055 , 1349907 , 9860294 , 3964 , 2812 , 33036 , 3103 , 164522 , 65895 , 14677 , 10219 , 37632 , 16739244 , 55482 , 50287 , 2391 , 3396 , 101616 , 3957 , 2176 , 175540 , 4030 , 10660 , 5510 , 11683 , 11954293 , 3478 , 3698 , 71768094 , 71851 , 3973 , 2082 , 2350 , 5585 , 24745335 , 5440 , 5593 , 2170 , 2564 , 31072 , 4761 , 5576 , 40589 , 3516 , 2484 , 3324 , 2750 , 4753 , 3333 , 2361 , 3182 , 4107 , 5736 , 44112 , 441281 , 14052 , 10624 , 1238 , 119828 , 2197 , 2435 , 11790 , 65820 , 107782 , 21855 , 14385 , 941651 , 4174 , 4156 , 10237 , 54385 , 3658 , 2749 , 119570 , 2132 , 3117 , 4760 , 3406 , 2123 , 5541 , 4992 , 13765 , 2366 , 5284514 , 5566 , 5587 , 2342 , 26035 , 441383 , 2478 , 65906 , 4843 , 6834 , 4066 , 124087 , 1547484 , 3561 , 36811 , 2333 , 9976892 , 2467 , 16574 , 11643449 , 2577 , 2795 , 2247 , 26987 , 44623946 , 2758 , 5921 , 5574 , 3168 , 19371515 , 3639 , 21138 , 17747460 , 5198 , 12454 , 1355 , 3151 , 2754 , 4615 , 60854 , 4912 , 57697 , 1981 , 2265 , 3827 , 16129778 , 6729 , 119259 , 3219 , 11286230 , 54677971 , 2725 , 11697697 , 2200 , 11291 , 3372 , 1046 , 4855 , 16362 , 123600 , 3748 , 5282230 , 1549000 , 2753 , 115237 , 15723 , 2160 , 5281071 , 6726 , 1615 , 6918314 , 17134 , 2913 , 6761 , 4197 , 3334 , 3389 , 2883 , 774 , 42601552 , 5452 , 1548942 , 60795 , 4830 , 19861 , 5282443 , 3108 , 2264 , 197033 , 170336 , 4940 , 5073 , 5002 , 5405 , 182137 , 133017 , 16960 , 3559 , 5722 , 57469 , 12124 , 50942 , 60149 , 4927 , 3348 , 25070031 , 1030 , 41376 , 2159 , 4122 , 3100 , 35802 , 54676038 , 3162 , 16231 , 5335 , 21109 , 4748 , 4585 , 2678 , 3435 , 2893]

1632|P28223(101) [4830 , 5533 , 13765 , 23897 , 60809 , 11683 , 2812 , 3151 , 60835 , 62065 , 3168 , 16106 , 47811 , 8226 , 3686 , 941651 , 82148 , 54562 , 54746 , 5452 , 2082 , 4174 , 2123 , 12124 , 1547484 , 1548942 , 11961293 , 9805719 , 6005 , 8223 , 16739244 , 2725 , 27400 , 4760 , 71768094 , 35802 , 1981 , 5335 , 25293 , 3559 , 4585 , 3389 , 36811 , 3561 , 4843 , 71202 , 10071196 , 4449 , 5510 , 28693 , 10257 , 2750 , 5576 , 2478 , 54676038 , 1046 , 448400 , 1001 , 11292933 , 107782 , 40589 , 1349907 , 10219 , 5160 , 11286230 , 2482 , 119259 , 3334 , 3748 , 1030 , 4122 , 16129778 , 5311271 , 14385 , 5198 , 11697676 , 119570 , 14868 , 11683556 , 50942 , 10531 , 55752 , 16574 , 2749 , 443951 , 21138 , 3478 , 3396 , 3372 , 3516 , 44112 , 5593 , 10624 , 123600 , 5761 , 4753 , 9821951 , 60149 , 2754 , 4506 , 3108 , 37632 , 60785 , 3333 , 131204 , 2435 , 16007088 , 71851 , 16231 , 16362 , 5440 , 11954293 , 3055 , 2132 , 1150 , 4107 , 5074 , 10660 , 5722 , 54677971 , 4156 , 3964 , 2265 , 6761 , 37459 , 3182 , 2170 , 60854 , 28864 , 5585 , 60262 , 68867 , 11430856 , 130918 , 3973 , 2333 , 2247 , 5002 , 3698 , 3822 , 4912 , 2758 , 31101 , 4078 , 4184 , 5921 , 3404 , 71351 , 49381 , 125564 , 5541 , 62865 , 3406 , 4030 , 2893 , 31072 , 2484 , 4748 , 21109 , 11643449 , 2732 , 11604525 , 39042 , 11658860 , 2467 , 1355 , 5405 , 2726 , 5736 , 4106 , 5073 , 2883 , 68186 , 2818 , 6918248 , 3435 , 2366 , 71360 , 11790 , 197706 , 60795 , 175540 , 219050 , 4197 , 2176 , 124087 , 1614 , 107992 , 2264 , 12454 , 5265 , 5011 , 2361 , 2913 , 119828 , 441383 , 14052 , 2159 , 17134 , 9966051 , 182137 , 4205 , 71781 , 68848 , 2577 , 1615 , 115237 , 4828 , 197033 , 3117 , 6077 , 2197 , 3324 , 3639 , 4855 , 1057 , 5566 , 57469 , 2753 , 2391 , 9860294]

1633|Q5JWF2(180) [228244 , 327044 , 2179 , 2754 , 4163388 , 2894446 , 651353 , 3091626 , 28803 , 650908 , 3261980 , 2938038 , 6063342 , 50248 , 4295316 , 770040 , 2291413 , 4118928 , 3241521 , 593113 , 12449 , 351111 , 657534 , 2865112 , 3442589 , 2735646 , 2165979 , 1369 , 246835 , 5389584 , 808378 , 11289 , 24793507 , 3127493 , 25134246 , 2898508 , 22430860 , 31475 , 13752 , 657977 , 294256 , 736069 , 3156690 , 5541 , 3236575 , 4337923 , 647038 , 3237705 , 3245285 , 651913 , 2304617 , 3238739 , 3708374 , 2819985 , 5240507 , 1870615 , 4103738 , 14604 , 24793326 , 3244583 , 16410213 , 3126762 , 19646 , 234387 , 16060802 , 3239387 , 1870753 , 801418 , 2827330 , 24792050 , 23009 , 1473386 , 3239879 , 2174167 , 3799111 , 4843 , 893703 , 3236588 , 3237465 , 2984762 , 3136028 , 3235671 , 316274 , 10133 , 824155 , 3145395 , 267368 , 332697 , 3236383 , 894690 , 3245402 , 3095236 , 44144252 , 2802499 , 35758 , 2834684 , 16190984 , 602681 , 2836838 , 24761713 , 3243710 , 41109 , 2737716 , 1286501 , 2846481 , 1815811 , 6472026 , 2016 , 246441 , 843822 , 3245025 , 16191563 , 3156762 , 8395 , 4396341 , 658099 , 22430904 , 24466 , 10104227 , 1973720 , 581148 , 3969 , 265436 , 2806901 , 607728 , 7329 , 3639 , 238499 , 4343310 , 3237649 , 3842920 , 5187962 , 645503 , 683816 , 3235986 , 3236583 , 223613 , 24816706 , 1811924 , 2426546 , 2763709 , 68634 , 722193 , 6470206 , 4624023 , 2162118 , 16190941 , 976292 , 3239200 , 3244430 , 660051 , 31236 , 2361 , 1815812 , 3836519 , 265341 , 806859 , 6624620 , 3236874 , 3240006 , 2180707 , 22530 , 3239339 , 3764070 , 3240442 , 3648616 , 2133505 , 3240403 , 5074 , 3108051 , 2090 , 13789 , 903966 , 2812 , 3243609 , 3090880 , 3865676 , 265580 , 19003 , 4343274 , 24856310 , 361939 , 3236724 , 2166261 , 1530100 , 3136361 , 4030278 , 10219 , 16306185 , 26695 , 100095 , 4659978 , 3603333 , 1810986 , 3236681 , 44201975 , 549445 , 3731631 , 535796 , 3245411 , 2844395 , 3551080 , 657677 , 660883 , 1082702 , 3242288 , 3243850 , 660688 , 788502 , 240112 , 1745499 , 24856270 , 3244292 , 3236558 , 2355 , 2795 , 2904782 , 71209 , 652720 , 3246767 , 573613 , 3244425 , 296597 , 3420746 , 3242481 , 2763377 , 2768974 , 21749]

1634|P14416(110) [2448 , 3108 , 3334 , 57469 , 21138 , 115237 , 124087 , 125564 , 6918314 , 2482 , 4926 , 10824155 , 9826744 , 2883 , 3748 , 57242 , 5405 , 4850 , 1355 , 14868 , 681 , 4855 , 5541 , 4843 , 3516 , 123600 , 3055 , 3973 , 2123 , 131204 , 2818 , 16739244 , 107782 , 2754 , 14052 , 2893 , 6005 , 10219 , 1046 , 3372 , 42601552 , 4753 , 2197 , 2812 , 16106 , 2247 , 2333 , 10420539 , 10660 , 3151 , 1615 , 3388 , 5736 , 6761 , 5074 , 115368 , 3559 , 31765 , 36811 , 12124 , 208951 , 4168 , 4030 , 37459 , 4585 , 5576 , 2753 , 5335 , 3478 , 197033 , 4122 , 5921 , 11790 , 3182 , 2366 , 62867 , 10705550 , 10116877 , 16363 , 3324 , 2342 , 2577 , 47811 , 219050 , 11430856 , 1981 , 17134 , 5355 , 68950 , 4107 , 119570 , 21109 , 4748 , 2725 , 55645 , 54677971 , 9818479 , 18104 , 11154555 , 119259 , 6918525 , 71351 , 175540 , 16 , 44112 , 688272 , 54477 , 60820 , 667467 , 23897 , 54746 , 3964 , 114840 , 5440 , 4912 , 52919 , 4506 , 4078 , 60795 , 5510 , 2170 , 5198 , 27400 , 5826 , 2361 , 2478 , 2391 , 11978813 , 3033769 , 1238 , 37632 , 2265 , 12454 , 10836 , 14385 , 2467 , 60149 , 5002 , 2749 , 2913 , 5073 , 4174 , 2264 , 2580 , 1614 , 11286230 , 3389 , 10531 , 2732 , 40589 , 2082 , 2484 , 54676038 , 2159 , 10667966 , 5593 , 11683 , 182137 , 441383 , 3698 , 1057 , 5095 , 1030 , 68634 , 2132 , 3686 , 11292933 , 10624 , 4528 , 1547484 , 3406 , 4197 , 2758 , 4917 , 444254 , 28864 , 3396 , 50942 , 4760 , 3333 , 3168 , 71360 , 5281881 , 5330286 , 35802 , 3117 , 60854 , 11697676 , 10039198 , 11954293 , 1349907 , 9860294 , 2176 , 119828 , 10775317 , 4420454 , 60809 , 11643449 , 54562 , 3639 , 16231 , 17676 , 2726 , 3561 , 6077 , 15443 , 71851 , 5722 , 31101 , 5566 , 2435 , 44623946 , 443951 , 16129778 , 31072 , 1548942 , 59227 , 39042 , 4156 , 57267 , 5585 , 2750 , 3435 , 13765 , 5311507 , 16362 , 13542 , 187 , 16574]

1635|Q5TCI8(0) [2754 , 1349907 , 3516 , 2123 , 2577 , 1548942 , 7347 , 5459650 , 157922 , 4030 , 6855 , 11683 , 6604423 , 2132 , 192197 , 41684 , 3639 , 31343 , 4030278 , 1057 , 35375 , 2435 , 10831 , 5480 , 10783 , 598513 , 1967 , 3108 , 3236874 , 11088 , 123606 , 3002820 , 2883 , 17113 , 3969 , 115368 , 2576 , 3433 , 3724 , 3503 , 1488408 , 2762 , 3005573 , 14052 , 10770 , 3333 , 104850 , 26533 , 4760 , 4487 , 2913 , 3885 , 16363 , 3182 , 16015629 , 3686 , 2794 , 14868 , 4578 , 3760 , 124087 , 2562 , 55918 , 3677 , 10036135 , 3926 , 3973 , 3117 , 2365 , 4770 , 152951 , 196122 , 3074827 , 8569 , 5722 , 10850 , 2176 , 2377 , 3781338 , 3542 , 51040 , 4506 , 660989 , 1234 , 6237 , 1985 , 16231 , 2758 , 2998359 , 1694 , 16190945 , 3245285 , 2178 , 6127 , 37175 , 33925 , 15459 , 21138 , 10382715 , 70464 , 10237 , 7191 , 361655 , 107985 , 31264 , 19910 , 26937 , 119182 , 1989 , 1123 , 170336 , 10114 , 2159 , 101616 , 175540 , 54675783 , 4097 , 6077 , 21414 , 441383 , 2265 , 3236724 , 155774 , 10235 , 2997693 , 3936 , 16362 , 4622 , 2017 , 4572075 , 3109 , 8138 , 54677972 , 1892 , 3515 , 4362 , 3540 , 652629 , 3759 , 6982 , 2333 , 176870 , 2090 , 3241177 , 19003 , 119259 , 666418 , 2315 , 1547484 , 67686 , 3404 , 208820 , 1815815 , 3736 , 2717 , 16188984 , 15723 , 31072 , 6307 , 36811 , 27648 , 182137 , 5531 , 3245131 , 3236065 , 3316 , 8609 , 660708 , 3865676 , 4621782 , 1811924 , 5593 , 2707 , 3334 , 2893 , 2467 , 11289 , 2200 , 12492 , 23897 , 57469 , 1552036 , 24239 , 31729 , 107715 , 12454 , 10660 , 4165 , 2092 , 2310 , 115163 , 16960 , 5510 , 4064 , 5723 , 2315667 , 35455 , 1052 , 5541 , 2482 , 4122 , 2950 , 2277 , 3055 , 1561922 , 1050 , 6603842 , 3311 , 4855 , 6603901 , 4107 , 26596 , 5233 , 3326 , 3783853 , 2247 , 4031 , 2391 , 1392 , 3351 , 2450 , 3156710 , 27400 , 72157 , 16187418 , 4842 , 32681 , 3168 , 2081 , 10133 , 36303 , 10221470 , 3599497 , 3698 , 10168 , 2327 , 9873 , 8907 , 114811 , 4197 , 54677971 , 15443 , 5074 , 2478 , 104838 , 17100 , 1549789 , 3406 , 10219 , 3119467 , 11310 , 13791 , 22571 , 16190984 , 3455 , 2366 , 10476437 , 2812 , 54676038 , 5335 , 1568843 , 2082 , 2484 , 3435 , 2170 , 3647 , 47472 , 16351 , 5576 , 2229 , 3080557 , 4753 , 2113270 , 160355 , 4174 , 3151 , 4602 , 16574 , 5405 , 1870615 , 2179 , 4493 , 2369 , 2468 , 3478 , 5289501 , 660883]

1636|Q9UNA4(14) [2063649 , 225371 , 3244776 , 949760 , 21501 , 1993 , 15945601 , 6466196 , 14604 , 3092847 , 4142675 , 10219 , 2896475 , 5074 , 2732927 , 163659 , 16191546 , 166553 , 263177 , 4680274 , 4655877 , 1756352 , 19266 , 24792601 , 133621 , 2735646 , 652629 , 2490338 , 255948 , 3751717 , 612424 , 1369 , 22430877 , 394347 , 421697 , 4456136 , 824155 , 3698 , 11852 , 54675783 , 3126762 , 680935 , 6469502 , 21453 , 3261980 , 30717 , 31236 , 3114023 , 2997734 , 2724 , 1599306 , 3238124 , 602681 , 843822 , 1967 , 5459650 , 12028 , 2815581 , 16362 , 3156995 , 273053 , 2435 , 15987950 , 3127284 , 3241177 , 5405 , 3237949 , 1719874 , 2057115 , 3034186 , 1090900 , 101744 , 1870615 , 10621 , 2762 , 16187418 , 660989 , 598513 , 2775706 , 3114022 , 2576 , 3151 , 3126341 , 10168 , 40146 , 8569 , 1676 , 5359646 , 19910 , 50942 , 4021578 , 2384580 , 3705369 , 4777950 , 246831 , 3218215 , 3478 , 21109 , 3435 , 219081 , 5934127 , 24761713 , 646716 , 33925 , 54676538 , 911675 , 655601 , 13752 , 4343526 , 4396341 , 824727 , 1713166 , 2990745 , 11296583 , 327044 , 16467159 , 2178 , 2932343 , 3847167 , 261282 , 3243347 , 1937568 , 739358 , 1878823 , 15953533 , 4404908 , 16347 , 3238739 , 16187479 , 4619 , 16739648 , 3117 , 3156743 , 54677972 , 2090 , 1973720 , 2291046 , 3094465 , 7329 , 2997693 , 170344 , 2277 , 1896320 , 4150224 , 4770 , 240112 , 2735009 , 3090866 , 3158622 , 24361 , 2022387 , 2294842 , 244136 , 2213986 , 6472251 , 3731631 , 361655 , 2998 , 3799111 , 3746037 , 136654 , 2426546 , 2234617 , 588415 , 2717 , 24792050 , 2220273 , 23009 , 2113270 , 123435 , 3218771 , 44201498 , 3239925 , 3238154 , 2053712 , 3243567 , 3503 , 6301 , 25102564 , 3239339 , 3326 , 4027541 , 2016 , 647116 , 750895 , 2998359 , 327045 , 2448 , 24816636 , 235434 , 1548942 , 16745942 , 3885 , 31475 , 2768975 , 1811924 , 22571 , 54680702 , 3794836 , 1694 , 175540 , 659036 , 16269005 , 3406 , 6473420 , 119570 , 3244425 , 3246760 , 3842920 , 2348004 , 22430777 , 4278 , 1892 , 3151041 , 2375956 , 3150575 , 3245025 , 1985 , 16270080 , 2315667 , 3242888 , 4077789 , 2482 , 72462 , 24793507 , 2931883 , 16187348 , 614669 , 3132640 , 4777942 , 1088 , 647884 , 1701 , 265436 , 2327 , 1066 , 98514 , 114811 , 54690031 , 2739563 , 3156710 , 5135482 , 2369 , 5480 , 3003803 , 2179 , 1432578 , 2305017 , 4343310 , 5289501 , 255945 , 16188943 , 719651 , 4154053 , 2844395 , 10767 , 605693 , 25102556 , 3237705 , 19646 , 6471716 , 3138364 , 251792 , 1810986 , 246835 , 19675 , 22530 , 101616 , 1561922 , 1608140 , 348986 , 2942924 , 1949 , 16189712 , 669633 , 65630 , 26695 , 2265 , 28213 , 3163418 , 2421810 , 3239584 , 6 , 4572075 , 4506 , 2384672 , 104762 , 22430904 , 5335 , 2234553 , 18573525 , 288875 , 798678 , 6603901 , 25102723 , 19529 , 249321 , 2132993 , 4014178 , 16268999 , 69594 , 25102671 , 3114024 , 2743305]

1637|Q96KQ7(13) [1608140 , 1967 , 1547484 , 10245972 , 65768 , 5153171 , 3108 , 219081 , 199 , 3125446 , 249 , 2170 , 3478 , 3885 , 123435 , 13113 , 6918508 , 1052 , 1973720 , 1050 , 2194 , 22571 , 6469502 , 2421810 , 10206 , 2717 , 3813687 , 18104 , 5405 , 2831207 , 14878 , 194699 , 3515 , 5289501 , 35758 , 4493 , 2179 , 5722 , 13765 , 65790 , 4593 , 4365905 , 327045 , 24761772 , 361655 , 208820 , 2831167 , 1066 , 3542 , 2081 , 24978701 , 2229 , 3074827 , 4760 , 5074 , 11350 , 68942 , 4680274 , 3116068 , 10104227 , 104741 , 3647 , 24107 , 1234 , 288875 , 70846 , 10237 , 55918 , 2990797 , 1780 , 332697 , 5459668 , 660688 , 2384672 , 2216 , 3760 , 10258 , 3333 , 22530 , 3455 , 234310 , 2466 , 68089 , 104826 , 2743305 , 6194 , 16362 , 54690031 , 11683 , 1861634 , 2369 , 16269005 , 3298512 , 2998 , 2366 , 2435 , 1552036 , 3253930 , 15250 , 28446 , 4578 , 3138373 , 1045 , 3969 , 19910 , 67686 , 265580 , 2327 , 3236588 , 3132640 , 16351 , 1082702 , 547914 , 10036135 , 36303 , 4843 , 3109 , 14369 , 10168 , 752652 , 10782 , 1355 , 5381 , 5392 , 4278 , 2950 , 1349907 , 3762 , 3406 , 3245728 , 16960 , 65909 , 12454 , 5480 , 68363 , 1600802 , 3639 , 3973 , 2913 , 5335 , 646716 , 2762 , 4110197 , 2484 , 10718 , 660989 , 3117 , 3759 , 2396 , 2178 , 3686 , 178144 , 2017 , 10252 , 158781 , 3298363 , 5918 , 3239584 , 1694 , 133621 , 2161 , 3275 , 44112 , 76915 , 3503 , 31475 , 3139316 , 2265 , 1870615 , 3005573 , 4122 , 24792593 , 3559 , 175540 , 3136361 , 23897 , 54677971 , 2450 , 2219849 , 104762 , 60871 , 107715 , 756673 , 2213986 , 156419 , 2082 , 53708 , 2739563 , 2754 , 2794 , 685814 , 3126341 , 3932 , 4362 , 1549789 , 2562 , 1993 , 2079895 , 683816 , 1720828 , 10230 , 107782 , 7347 , 6603842 , 11088 , 3138330 , 122623 , 1752606 , 10404 , 1989 , 3805738 , 6603901 , 162834 , 6473420 , 4238274 , 9363 , 3676681 , 1051 , 16231 , 4119575 , 1568843 , 2123 , 2132993 , 3138370 , 2333 , 16129778 , 72139 , 2247 , 3151041 , 13986 , 3698 , 3245131 , 3120949 , 124087 , 5510 , 648831 , 3055 , 2182 , 3433 , 10275 , 3138364 , 10651 , 28688 , 3156995 , 2159 , 65758 , 21138 , 3334 , 10212 , 5917 , 194595 , 122081 , 3095271 , 5593 , 612424 , 108189 , 123600 , 13505 , 32681 , 3277 , 2090 , 649959 , 10783 , 22297 , 2063649 , 657677 , 160355 , 24792590 , 5185709 , 2846481 , 4619 , 54675783 , 3151 , 5233 , 104838 , 3713404 , 3936 , 1046 , 104850 , 4343310 , 65620 , 2092 , 141870 , 4655877 , 16837 , 71905 , 652720 , 89105 , 10219 , 441383 , 13916 , 10767 , 2753 , 115150 , 50248 , 14677 , 2197 , 20544 , 36811 , 1561922 , 176870 , 131411 , 5420 , 1599306 , 14899645 , 2318 , 3865676 , 2812 , 1392 , 132862 , 750895 , 3237465 , 3540 , 54677972 , 41684 , 10382715 , 7191 , 3002119 , 1238 , 35455 , 110635 , 3242068 , 3731631 , 6604423 , 2467 , 54676538 , 2576 , 3092847 , 94280 , 2277 , 4380 , 10612 , 2162118 , 1811924 , 3311 , 101616 , 14052 , 2315667 , 24817194 , 21307 , 115237 , 1649 , 11967809 , 1057 , 4342 , 3244341]

1638|O75496(20) [3603333 , 2057112 , 16269005 , 2369 , 6237 , 2758 , 16060802 , 2167047 , 31729 , 19910 , 3118 , 3236575 , 57469 , 19675 , 68089 , 6473420 , 1234 , 3139316 , 4197 , 1893 , 15945601 , 3561 , 4342 , 2113270 , 3515 , 2562 , 19529 , 2831167 , 6855 , 33309 , 194699 , 1676 , 25102671 , 1985 , 2913 , 2831207 , 5005498 , 2799 , 14604 , 10036135 , 18573631 , 101616 , 11289 , 5282060 , 20544 , 3783853 , 3503 , 2391 , 65681 , 108189 , 13916 , 2482 , 27448 , 2997 , 13986 , 68805 , 18573632 , 3746037 , 6918508 , 28061 , 2081 , 3082 , 16270080 , 55918 , 5510 , 54677971 , 2866904 , 1967 , 2827330 , 10832 , 17134 , 4622 , 2732927 , 67686 , 3244583 , 6603842 , 3953130 , 5541 , 3117 , 72136 , 5459650 , 3182 , 2063649 , 1719874 , 4030 , 68872 , 18104 , 2450 , 11954283 , 16190984 , 65620 , 3478 , 182137 , 26596 , 1719873 , 12124 , 10206 , 5381 , 5722 , 1778877 , 8210 , 24761772 , 16187479 , 5420 , 10782 , 3138330 , 10237 , 4122 , 26695 , 6603901 , 24792593 , 3421253 , 273053 , 25102723 , 18573524 , 3032279 , 911675 , 6063342 , 24789385 , 2245988 , 3156995 , 13113 , 2164757 , 736069 , 62485 , 170344 , 3108 , 151506 , 4027541 , 10770 , 89105 , 28803 , 3932 , 3333 , 131411 , 15723 , 4770 , 74046 , 131204 , 2846481 , 8041 , 31072 , 1694 , 19646 , 31475 , 1720828 , 15443 , 5918 , 441383 , 4097 , 11310 , 6764 , 15583 , 16191372 , 22430825 , 50259 , 2836838 , 3236588 , 16347 , 16653 , 5074 , 9551522 , 4284 , 16188984 , 4163388 , 3240006 , 1088 , 16467159 , 16410213 , 3244194 , 10114 , 40146 , 2883 , 24792601 , 3334 , 4278 , 13791 , 21109 , 3312 , 2082 , 3698 , 2906039 , 6077 , 3244262 , 4673656 , 5187962 , 647499 , 1432578 , 3351 , 119259 , 2932047 , 7699 , 2576 , 2289748 , 3091626 , 2090 , 25102672 , 3973 , 2478 , 3156727 , 6466196 , 1780 , 1561922 , 192197 , 3926 , 1878823 , 4154053 , 10172943 , 11954034 , 12492 , 4760 , 4564 , 16362 , 248271 , 7352 , 2997734 , 10168 , 219081 , 36811 , 65651 , 394347 , 327045 , 20906 , 4843 , 68186 , 14677 , 3244566 , 3969 , 17676 , 10219 , 3005573 , 19920 , 327044 , 12329 , 2732 , 16745942 , 5480 , 2794 , 157922 , 2923668 , 2735009 , 249 , 18573525 , 65768 , 2775706 , 6271 , 175540 , 72157 , 107715 , 8569 , 3474 , 2182 , 107782 , 1369 , 4165 , 3616 , 10767 , 3034590 , 101744 , 16231 , 24239 , 3455 , 22297 , 7329 , 3298363 , 3245411 , 68684 , 3542 , 3885 , 10651 , 12454 , 119182 , 104850 , 21867154 , 2318 , 115368 , 21138 , 2453 , 176870 , 3404 , 5723 , 24761713 , 42725 , 72900 , 31276 , 238499 , 3236558 , 6301 , 3671 , 647201 , 4237 , 3127493 , 44825776 , 16725155 , 35455 , 10850 , 5185709 , 13765 , 10221470 , 3781338 , 446816 , 6 , 3242 , 3218215 , 1050 , 3277 , 41684 , 649156 , 1392 , 4506 , 1547484 , 12449 , 361655 , 16490 , 3002119 , 1811924 , 9550559 , 10133 , 11967800 , 5475 , 10046567 , 5921 , 12132 , 10517 , 4031 , 13505 , 160355 , 2200 , 22571 , 16189712 , 54676538 , 10235 , 156419 , 5392 , 72139 , 11350 , 2963649 , 3730 , 3435 , 65758 , 3647 , 2132993 , 11683 , 100472 , 265580 , 1701 , 169682 , 4614751 , 5233 , 1552036 , 3610 , 36303 , 3125446 , 3127904 , 51040 , 16059888 , 3758 , 4578 , 2327 , 1892 , 656641 , 3830 , 16574 , 33925 , 3677 , 5531 , 4014178 , 114924 , 2739563 , 8144119 , 2819985 , 3559 , 3218771 , 31593 , 1568843 , 11684 , 4511 , 5240507 , 243274 , 24791741 , 14052 , 3151 , 4362 , 3551080 , 5405 , 208820 , 2812 , 1355 , 54676038 , 10382715 , 3760 , 210320 , 3516 , 421697 , 3245385 , 1815815 , 56463 , 2942924 , 2484 , 2016 , 4396341 , 5289501 , 2170 , 547914 , 76915 , 11293 , 2247 , 54690031 , 2265 , 65909 , 2366 , 45480035 , 2997693 , 121396 , 2754 , 3237015 , 161562 , 2179 , 10831 , 32681 , 4343274 , 3241429 , 50248 , 2467 , 94280 , 2229 , 70464 , 121098 , 3639 , 1989 , 21307 , 11852 , 2333 , 4795607 , 5062924 , 7347 , 14868 , 3109 , 11779629 , 122077 , 13752 , 2750 , 8026]

## **Drug targets mapped to human proteome (D+ dataset)**

The D+ dataset contains proteins from the D dataset plus the below human proteins that share high sequence similarity (≥ 90%) to known drug targets from other organisms.

The list of is in the following format.
*<Sequential index>*|<*Uniprot ID>*(<*number of disease annotation>*)

1|O00506(4)

2|O00555(192)

3|O00764(7)

4|O14594(15)

5|O15144(4)

6|O60391(5)

7|O60939(6)

8|O75874(129)

9|O94768(12)

10|O95461(57)

11|O95819(19)

12|P00156(75)

13|P00352(84)

14|P00403(311)

15|P00491(63)

16|P00748(74)

17|P01215(67)

18|P04083(119)

19|P04406(140)

20|P04792(142)

21|P07339(103)

22|P07998(26)

23|P08319(24)

24|P08758(129)

25|P09211(364)

26|P09919(213)

27|P0DMN0(3)

28|P0DMS9 (0)

29|P11021(126)

30|P11310(27)

31|P13196(9)

32|P13674(8)

33|P14618(58)

34|P16109(393)

35|P16389(21)

36|P17707(23)

37|P19224(34)

38|P19367(114)

39|P19525(81)

40|P22001(133)

41|P22102(16)

42|P22301(759)

43|P22309(143)

44|P22459(5)

45|P22736(73)

46|P23528(39)

47|P24385(353)

48|P24557(32)

49|P24666(118)

50|P26358(177)

51|P28062(71)

52|P28482(326)

53|P29320(70)

54|P31941(26)

55|P32246(78)

56|P33176(18)

57|P34897(12)

58|P37058(23)

59|P39748(41)

60|P40189(89)

61|P40261(41)

62|P41279(75)

63|P42229(121)

64|P43003(38)

65|P43351(29)

66|P46734(22)

67|P48051(38)

68|P48547(6)

69|P48549(16)

70|P48637(30)

71|P49441(6)

72|P49674(21)

73|P51570(10)

74|P51793(16)

75|P51956(3)

76|P53355(80)

77|P54289(15)

78|P54756(9)

79|P61088(28)

80|P63252(32)

81|P83916(12)

82|Q00536(21)

83|Q01453(206)

84|Q02108(13)

85|Q02153(2)

86|Q03721(9)

87|Q04760(57)

88|Q05329(70)

89|Q05397(74)

90|Q06710(64)

91|Q07002(2)

92|Q09470(27)

93|Q13123(6)

94|Q13255(53)

95|Q13393(60)

96|Q13509(49)

97|Q14721(13)

98|Q14749(26)

99|Q15067(28)

100|Q15274(6)

101|Q15878(8)

102|Q16280(1)

103|Q16288(65)

104|Q16678(131)

105|Q2Y0W8 (0)

106|Q68DU8(1)

107|Q6U841(10)

108|Q6ZWB6 (0)

109|Q96BR1(9)

110|Q96CX2(6)

111|Q96D96(7)

112|Q96GD3 (0)

113|Q96L42(30)

114|Q96PR1(2)

115|Q9BY07(4)

116|Q9GZT9(39)

117|Q9HBA0(51)

118|Q9NVS9(14)

119|Q9NZV8(8)

120|Q9P0L2(14)

121|Q9UBN4(11)

122|Q9UNX9(1)

123|Q9Y6M7(11)

124|Q9Y6R1(19)

## **Non-drug target dataset (N dataset)**

The list of proteins in the non-drug target dataset in the following format
Sequential index of the record|UniprotID(*number of disease annotation*)

1|Q7Z398(0)

2|Q9H0E7(9)

3|O00628(44)

4|P40227(4)

5|Q9NQT6(1)

6|Q49A88(0)

7|Q86SJ2(2)

8|Q9HC77(10)

9|Q8N5F7(2)

10|Q6ZNW5(0)

11|Q9C0H9(7)

12|Q69YN2(10)

13|Q9BZQ8(7)

14|Q96J65(4)

15|Q9UBM7(82)

16|Q8IYK4(0)

17|Q9BW85(1)

18|Q13835(23)

19|Q9UPI3(10)

20|P02545(514)

21|P02549(12)

22|Q7Z3T8(0)

23|Q01804(26)

24|A6H8Z2(0)

25|Q96KX0(0)

26|Q8TDF5(0)

27|P49788(18)

28|Q9HB75(4)

29|P78347(57)

30|Q9UQ07(88)

31|Q5T7W7(0)

32|P52272(4)

33|A0AVT1(0)

34|O76027(1)

35|P01709(0)

36|Q8N8Z8(0)

37|P35244(4)

38|Q6ZTB9(0)

39|P35249(7)

40|Q96RV3(0)

41|Q86UQ5(0)

42|A8MZG2(0)

43|Q9P215(2)

44|Q8NBI5(2)

45|Q9UKL2(0)

46|P00540(27)

47|Q9BXW7(0)

48|Q9H3M7(66)

49|O15320(10)

50|Q15573(0)

51|O75387(5)

52|Q8TEZ7(0)

53|P78562(40)

54|Q8TE96(1)

55|B3EWG5(0)

56|Q6NX45(0)

57|Q8WYB5(50)

58|Q86VW0(0)

59|Q6YHK3(12)

60|Q14587(4)

61|Q9UHF7(39)

62|O94907(119)

63|Q14588(0)

64|Q8N999(0)

65|Q92526(1)

66|Q92521(0)

67|O43790(7)

68|Q92529(17)

69|Q8N3C0(6)

70|Q9ULB4(1)

71|Q5T9C2(2)

72|F5H2V8(0)

73|Q96Q89(10)

74|Q96HA4(0)

75|P86397(0)

76|Q9H210(0)

77|Q99460(1)

78|O60383(9)

79|Q99466(49)

80|Q8TCT6(9)

81|Q9H3Q3(1)

82|Q6P2I3(0)

83|Q8TCT8(3)

84|Q5R3K3(1)

85|Q6UW02(4)

86|Q99990(4)

87|Q8WUF8(2)

88|Q8N1E2(0)

89|O15417(0)

90|Q13349(0)

91|Q8NDA8(0)

92|Q9BU40(0)

93|Q96MG8(1)

94|Q9BSW7(0)

95|P62495(5)

96|Q8IZM9(5)

97|Q86UF1(9)

98|Q96DR5(2)

99|Q8NH41(0)

100|A6NC97(0)

101|Q96A83(1)

102|Q9NWS9(0)

103|P16333(8)

104|Q8TAP6(0)

105|Q9BQ50(3)

106|P49771(41)

107|Q8N1W2(0)

108|O15528(76)

109|O95251(7)

110|Q96M20(0)

111|Q86SU0(5)

112|Q9BZ29(3)

113|Q16670(0)

114|A0MZ66(0)

115|Q9BS34(0)

116|Q9H0B3(0)

117|Q6YN16(5)

118|O15069(0)

119|Q9H6R4(0)

120|O15062(3)

121|Q9H6R6(0)

122|Q9H6R0(0)

123|P60891(35)

124|O95741(0)

125|P29083(2)

126|Q9NW64(0)

127|Q6UXX5(0)

128|Q96D71(2)

129|P05062(30)

130|Q8NAB2(0)

131|Q7Z4H3(1)

132|Q2TBF2(1)

133|Q6UXN2(3)

134|Q9BR01(18)

135|Q8NCJ5(0)

136|F8WAN1(0)

137|A6NHT5(0)

138|P49959(55)

139|Q9HB19(1)

140|P13747(50)

141|Q5FYB1(1)

142|Q5FYB0(0)

143|E9PFP8(0)

144|B0I1T2(1)

145|P56545(12)

146|Q9NWW9(0)

147|Q15935(1)

148|Q00341(9)

149|P78352(19)

150|P10265(0)

151|Q5T2R2(11)

152|Q8IYB7(1)

153|Q969T3(0)

154|O15084(1)

155|O15083(0)

156|P07476(17)

157|Q9Y613(1)

158|P35251(51)

159|Q08170(4)

160|Q8IY18(3)

161|P32926(27)

162|Q00765(24)

163|A4D2P6(0)

164|O00507(9)

165|O00505(2)

166|Q8TCI5(1)

167|Q4V328(0)

168|Q9Y6D5(15)

169|Q53GA4(12)

170|Q9Y6D6(6)

171|A0A286YF58(0)

172|Q8N9M5(0)

173|O75398(9)

174|Q8IYD9(3)

175|O75558(9)

176|Q8TE68(0)

177|Q96P67(0)

178|Q96P63(0)

179|P04280(15)

180|Q9BSJ8(1)

181|Q96R69(0)

182|Q9BSJ2(8)

183|Q9H992(5)

184|P27694(26)

185|Q9BTC8(11)

186|Q7Z5J1(3)

187|Q08378(0)

188|Q8WVD3(1)

189|P98077(4)

190|Q8WXH5(4)

191|P68543(2)

192|Q32M78(0)

193|Q06787(105)

194|P58872(0)

195|Q96RD0(0)

196|P54886(40)

197|Q9H9K5(0)

198|Q9H930(1)

199|Q9UGC7(0)

200|P52738(1)

201|P52736(0)

202|Q8WVV4(2)

203|A6NMK8(0)

204|O95405(15)

205|Q9HCQ5(1)

206|Q6P4A8(1)

207|Q9HAE3(1)

208|Q9H222(26)

209|Q6NUI2(3)

210|O75175(5)

211|A6NFR6(0)

212|Q6P4A7(3)

213|Q7Z6Z6(0)

214|P11215(131)

215|A6NCV1(0)

216|Q8N145(0)

217|Q8TCU3(4)

218|Q9UF12(1)

219|A6NI47(0)

220|Q6ZNG1(0)

221|Q8N5V2(0)

222|Q8WUA8(0)

223|Q5VY43(5)

224|Q9NY74(1)

225|Q8WUA2(0)

226|Q8WUA7(0)

227|P31943(6)

228|P49407(28)

229|Q9UHK0(1)

230|O15400(0)

231|Q9Y5P8(2)

232|Q9UG22(0)

233|Q9NYT6(0)

234|Q14916(7)

235|Q96DS6(0)

236|Q9UJU2(59)

237|Q8NDV1(0)

238|Q12778(125)

239|Q9ULI1(0)

240|P08048(8)

241|Q5FWF6(0)

242|Q9H6A9(1)

243|Q12840(21)

244|Q00978(32)

245|Q6P6B1(0)

246|Q8NHW5(0)

247|Q8NA66(1)

248|Q9BX59(0)

249|Q8WZA2(1)

250|Q9HC56(2)

251|Q9HD20(0)

252|Q9NQZ5(2)

253|Q9H6S1(7)

254|Q9UN73(0)

255|Q9NQZ2(0)

256|Q01954(7)

257|Q13585(2)

258|Q9NZJ9(0)

259|Q3MIW9(3)

260|Q9NZJ4(23)

261|Q6UXY8(2)

262|Q6XYQ8(2)

263|P30566(19)

264|A6NFQ2(1)

265|Q96ID5(1)

266|Q8NCE0(4)

267|P38159(11)

268|Q10589(29)

269|Q10588(7)

270|A6NNW6(0)

271|Q99729(3)

272|O00267(4)

273|Q86X51(4)

274|Q8WWQ2(28)

275|Q9Y2E6(2)

276|Q9Y2E4(5)

277|Q6ZTY9(0)

278|P55289(3)

279|Q7Z449(4)

280|O76042(0)

281|P62955(0)

282|P78363(95)

283|P23025(97)

284|Q8TAU3(0)

285|Q9BVJ6(0)

286|Q9BVJ7(3)

287|Q96SZ6(1)

288|O00232(10)

289|A8MWY0(0)

290|Q5T2S8(9)

291|E9PJK4(0)

292|Q15345(0)

293|Q53G59(1)

294|Q9P0M6(4)

295|P28336(8)

296|Q8NGC3(0)

297|A8MWK0(0)

298|Q8N9X3(0)

299|P20916(26)

300|Q9C0D3(0)

301|O95985(1)

302|Q8NGA1(0)

303|Q9UKJ1(5)

304|Q8TCJ0(2)

305|Q8NGA4(0)

306|Q9BZE9(9)

307|P02747(6)

308|P01033(250)

309|Q8TAB3(11)

310|Q92611(4)

311|Q9UKX3(1)

312|Q96QS6(0)

313|Q9BXC9(37)

314|Q92618(2)

315|Q53GL7(0)

316|Q53GL0(1)

317|Q96P71(2)

318|Q96P70(1)

319|Q8TE73(13)

320|Q9Y6E2(0)

321|Q9H7Y0(1)

322|Q8N335(13)

323|Q8TAP4(14)

324|P42785(23)

325|P0DPB3(0)

326|Q9NPH3(23)

327|Q92508(16)

328|Q9NRD8(41)

329|Q92503(1)

330|Q92502(6)

331|Q13107(12)

332|P61966(16)

333|A0A0A0MTC6(0)

334|Q13480(34)

335|Q8N539(0)

336|P04746(5)

337|A0A087WX45(0)

338|Q6UWR7(0)

339|Q68CR7(0)

340|P49326(4)

341|Q9HAD4(0)

342|Q68CR1(0)

343|O75185(5)

344|Q0JRZ9(0)

345|Q99680(4)

346|Q99689(12)

347|Q3L8U1(0)

348|P50120(21)

349|A0A578(0)

350|Q96BR1(9)

351|Q9UDR5(13)

352|Q96BR9(0)

353|Q15742(13)

354|Q01851(27)

355|Q6ZSJ8(1)

356|O14926(22)

357|P23760(62)

358|Q13360(1)

359|Q13363(50)

360|O60235(21)

361|Q6MZM0(0)

362|Q9UJT0(2)

363|Q96MS3(0)

364|Q9UHX3(6)

365|Q2M3W8(0)

366|Q9NS66(0)

367|Q96HU1(129)

368|Q9UPP5(0)

369|C9K0E4(0)

370|I3L273(0)

371|Q9NS82(6)

372|Q9BPY3(0)

373|Q8NA72(0)

374|Q8NI51(26)

375|P13378(5)

376|Q6UY01(0)

377|P13804(34)

378|Q6B0B8(0)

379|Q9NZK5(47)

380|P01854(0)

381|Q9BY12(2)

382|Q9Y6T7(3)

383|O60499(0)

384|Q86V40(0)

385|A0A0G2JPR9(0)

386|Q538Z0(1)

387|Q86V48(0)

388|Q8WWP7(0)

389|Q5HYR2(0)

390|Q5JTH9(1)

391|Q9NQ40(12)

392|Q9BYL1(0)

393|Q9P2P5(2)

394|Q7L099(4)

395|Q07666(69)

396|Q9NRY2(0)

397|Q658N2(2)

398|O60832(58)

399|O95352(44)

400|P18462(0)

401|Q9Y388(1)

402|Q96CB8(2)

403|P01733(0)

404|Q8NBX0(0)

405|Q5XKL5(0)

406|Q9NVA4(4)

407|Q86XN8(1)

408|Q04844(23)

409|P57721(0)

410|P28329(37)

411|Q01167(4)

412|Q9UIC8(2)

413|Q9BXR3(0)

414|Q96CT2(0)

415|A0A1B0GUC4(0)

416|P17677(24)

417|P51825(9)

418|Q6UXB3(0)

419|O15355(1)

420|Q5SW96(23)

421|Q9GZN1(0)

422|Q8NGL2(0)

423|Q8NGL4(0)

424|P25089(0)

425|O75578(1)

426|Q86VH2(0)

427|Q6P1L5(0)

428|P60370(0)

429|P32942(17)

430|Q86U17(1)

431|Q86U10(21)

432|Q92754(13)

433|Q8WVF1(24)

434|Q13117(3)

435|Q99942(15)

436|Q9NYD6(8)

437|Q92570(74)

438|Q9HAU4(22)

439|Q7L8L6(0)

440|A1KZ92(2)

441|Q8N831(0)

442|Q14249(22)

443|O95429(4)

444|Q96HD9(3)

445|Q8TD31(26)

446|P48200(21)

447|Q8IVL0(14)

448|P35410(3)

449|A6NI28(1)

450|Q8IVH4(2)

451|Q8TCW7(2)

452|Q8NGZ0(0)

453|Q9BWX1(0)

454|Q68E01(6)

455|A6NEE1(0)

456|P54198(25)

457|P19086(1)

458|Q16798(0)

459|O43347(39)

460|Q16401(0)

461|Q9Y5R4(0)

462|Q9UHI5(8)

463|Q9UHI7(27)

464|Q99547(6)

465|Q53EV4(0)

466|Q5T0Z8(0)

467|Q8IZF7(0)

468|Q12799(0)

469|Q15029(14)

470|O94777(8)

471|Q8IWJ2(0)

472|O15514(2)

473|Q9BUT1(6)

474|Q96G97(59)

475|Q9UPW5(5)

476|Q96EK2(2)

477|Q9NQX6(20)

478|A6NJW4(0)

479|Q17RS7(9)

480|Q8TBB1(4)

481|Q03405(74)

482|A6NJW9(0)

483|Q9BV94(0)

484|J3KSS5(0)

485|P30498(0)

486|P30493(0)

487|O95873(4)

488|Q13761(98)

489|P61571(0)

490|P15260(82)

491|P0CI00(0)

492|Q13569(19)

493|Q9Y2M2(0)

494|Q9BUJ2(5)

495|Q96BA8(13)

496|Q9BY07(2)

497|P50479(17)

498|Q9UJW8(0)

499|Q9Y6W6(11)

500|A6NHY2(0)

501|P37287(47)

502|O00204(9)

503|Q9Y2G2(30)

504|Q8WTS6(17)

505|Q6UX04(0)

506|Q6UX07(0)

507|Q9Y2G9(8)

508|O00192(31)

509|P17014(0)

510|Q9BPZ7(29)

511|Q03395(26)

512|A0A2R8Y712(0)

513|Q12893(1)

514|Q9BTT6(0)

515|O00330(39)

516|O60826(21)

517|Q96AQ6(3)

518|Q9BV79(2)

519|Q9NVF7(2)

520|Q969Y0(0)

521|Q9H5N1(9)

522|Q92185(15)

523|P0DMW3(0)

524|P78414(11)

525|P04180(36)

526|P78411(23)

527|Q8N228(0)

528|Q96QC0(4)

529|Q15035(0)

530|Q86V97(0)

531|Q9Y247(3)

532|A6NGE4(0)

533|Q8TE57(6)

534|Q5VT52(0)

535|O95948(5)

536|Q9UKV5(15)

537|Q6P087(0)

538|Q9Y4K0(37)

539|Q00005(27)

540|Q96AG3(6)

541|Q92966(0)

542|Q92968(48)

543|P16860(78)

544|Q9NPJ8(0)

545|Q99956(10)

546|Q13166(0)

547|P87889(0)

548|Q92569(27)

549|Q8NGE3(0)

550|P57082(15)

551|P57081(0)

552|Q7RTY7(0)

553|P57088(2)

554|Q7Z6K1(2)

555|Q8WU76(0)

556|Q8NES3(7)

557|P0CL85(0)

558|Q8N365(0)

559|Q8TD23(1)

560|Q04721(109)

561|P49184(0)

562|Q04726(6)

563|Q2VIQ3(0)

564|Q7Z7M9(1)

565|Q8IVI9(3)

566|Q6N075(0)

567|A0A1W2PPV3(0)

568|Q9BQC3(1)

569|Q86X55(23)

570|Q86X59(0)

571|P07204(114)

572|O60216(35)

573|Q96RQ3(2)

574|Q96RQ1(7)

575|O94761(84)

576|P10696(7)

577|A6NKB5(5)

578|Q9H9P8(7)

579|Q9Y5E7(0)

580|A6NIJ5(0)

581|Q13416(1)

582|E9PKP7(0)

583|Q8IU60(0)

584|P04406(127)

585|Q7Z6M2(0)

586|A4QPH2(0)

587|Q1T7F1(0)

588|Q9UPV0(7)

589|Q9NSD4(0)

590|Q8NI77(8)

591|P10600(87)

592|P55039(0)

593|Q6NSW5(0)

594|P55036(24)

595|Q9NZI6(2)

596|Q13772(22)

597|Q9NZI8(32)

598|P62072(1)

599|Q15784(3)

600|Q13574(8)

601|Q9BUI4(0)

602|Q6TFL3(0)

603|Q9H5H4(0)

604|Q8N7C7(0)

605|P30550(29)

606|Q52LD8(0)

607|Q9NP58(126)

608|Q03701(13)

609|Q6UXR8(0)

610|P17025(0)

611|O15198(10)

612|P17028(6)

613|P17029(0)

614|P61218(2)

615|V9GZ46(0)

616|Q9Y2F9(1)

617|P52824(2)

618|Q8NDY4(3)

619|Q5GLZ8(0)

620|Q1L6U9(1)

621|Q5H9K5(0)

622|Q5VWI1(5)

623|P40424(54)

624|Q96AP4(0)

625|P31513(27)

626|C9JK28(0)

627|Q49SQ1(0)

628|A8MUZ8(0)

629|P21796(29)

630|Q13950(112)

631|Q6XE24(78)

632|Q4KMX7(0)

633|A6NHQ4(0)

634|Q9BT67(4)

635|Q9H6X2(12)

636|Q687X5(13)

637|Q96NL0(2)

638|Q5BKX5(0)

639|Q96CV9(53)

640|P78406(3)

641|Q07283(2)

642|Q9UKI9(22)

643|P51809(15)

644|A2RRD8(0)

645|Q9BZX4(1)

646|Q8N2N9(22)

647|Q9Y6X2(25)

648|Q15021(1)

649|Q86V85(2)

650|Q3SXM5(0)

651|Q3SXM0(0)

652|Q6P9F5(2)

653|P78539(7)

654|Q8N766(1)

655|Q8TAW3(2)

656|Q9H1V8(1)

657|Q9P1Z0(4)

658|Q96R28(0)

659|Q5JWR5(2)

660|Q86TJ2(0)

661|P21579(126)

662|Q8NEB5(8)

663|Q86U37(0)

664|Q99961(8)

665|Q92556(15)

666|Q92551(5)

667|Q92552(0)

668|Q8N3D4(0)

669|Q14444(3)

670|O75200(0)

671|Q9Y3B3(103)

672|Q96PE6(0)

673|P49903(3)

674|Q6PJG6(3)

675|Q96SC8(0)

676|Q99819(3)

677|Q8NEB7(6)

678|Q8N587(0)

679|Q9UPG8(10)

680|Q9H267(20)

681|P0CJ78(0)

682|P0CJ79(0)

683|Q8WU67(0)

684|O94844(5)

685|P14621(8)

686|P32189(24)

687|Q99490(6)

688|Q68CQ4(2)

689|Q6ZWK6(0)

690|O75333(2)

691|Q8N108(4)

692|Q9H1X1(3)

693|Q9H1X3(0)

694|Q6ZUS6(0)

695|Q8TCQ1(13)

696|Q02086(3)

697|Q8NFA0(0)

698|Q5TID7(1)

699|O43567(3)

700|P07237(64)

701|Q96DM3(39)

702|Q9H9A7(10)

703|P58304(10)

704|Q9Y5T5(2)

705|Q9UNW9(5)

706|Q6DHV5(0)

707|Q96PP9(0)

708|Q8NB90(3)

709|Q9Y5F8(1)

710|Q8IZD0(2)

711|Q8IZD2(10)

712|Q8IZD4(5)

713|O14807(9)

714|Q8NEU8(9)

715|P12036(31)

716|P11161(43)

717|Q8TBE0(2)

718|Q86WK6(0)

719|Q9BX10(0)

720|Q06413(43)

721|Q969H0(96)

722|Q14CN4(1)

723|P0CI25(0)

724|Q6AZZ1(5)

725|O95714(33)

726|Q494R4(0)

727|Q9UJY5(1)

728|Q9NR63(13)

729|Q3B7T1(0)

730|Q9HB65(2)

731|Q9NQ66(19)

732|P33908(3)

733|Q8TDL5(12)

734|P84098(10)

735|Q6ZS11(0)

736|Q6ZRQ5(2)

737|Q8IVT5(17)

738|Q6V9R5(0)

739|Q9BVV6(17)

740|Q07617(12)

741|Q03591(23)

742|Q5T2W1(26)

743|P55265(51)

744|P10412(2)

745|Q5JWF8(0)

746|Q9NUJ3(1)

747|P55795(3)

748|A3QJZ6(0)

749|Q86UW9(1)

750|Q9Y467(11)

751|Q9Y466(14)

752|Q9UKF2(0)

753|Q9UM63(42)

754|Q9BZY9(4)

755|O15389(1)

756|Q96S53(0)

757|O60449(13)

758|O43566(6)

759|P78329(17)

760|Q9HBE1(7)

761|O14490(4)

762|Q9HBE5(40)

763|A8K8P3(4)

764|O75529(3)

765|Q9UKT4(5)

766|Q8IX04(2)

767|P0C869(0)

768|Q8TAT5(3)

769|Q8NFW8(13)

770|P08247(54)

771|Q6ZMJ2(7)

772|P00414(57)

773|Q03828(3)

774|Q9NRR5(1)

775|Q9H720(0)

776|O43824(0)

777|P02675(96)

778|O95817(57)

779|Q92945(40)

780|A0A087WV07(0)

781|O95810(13)

782|P43631(62)

783|O43776(4)

784|Q92785(0)

785|Q5T7M4(1)

786|Q92782(0)

787|P46736(10)

788|Q5T7M9(1)

789|Q9Y3C5(6)

790|A8MYP8(1)

791|Q6PCE3(0)

792|P17213(32)

793|Q6P9G9(0)

794|Q86XW9(2)

795|Q8N594(0)

796|Q96JG6(0)

797|Q96JG9(0)

798|Q96LW1(0)

799|Q8WV83(0)

800|Q13098(1)

801|O75475(59)

802|Q9BQS8(0)

803|A6NL46(0)

804|Q5JPI3(0)

805|Q2M2H8(0)

806|O95544(1)

807|Q00G26(0)

808|A1L4K1(1)

809|Q96LA5(3)

810|Q96DN2(5)

811|Q9GZW5(0)

812|Q00839(8)

813|Q96MA6(0)

814|Q5J8X5(0)

815|Q96MA1(0)

816|Q8IUY3(0)

817|O60279(0)

818|Q8IWU2(3)

819|Q70YC5(11)

820|Q9Y5G1(0)

821|Q9Y5G6(0)

822|Q9Y5G5(0)

823|Q96PQ7(0)

824|Q86Y46(0)

825|Q8WXX7(14)

826|Q15651(0)

827|Q13435(0)

828|Q8NC56(1)

829|Q6DD87(0)

830|P81274(12)

831|P11117(6)

832|P06576(9)

833|A8MYZ6(0)

834|P15291(15)

835|Q9NWY4(0)

836|Q9UJX4(1)

837|P50336(18)

838|Q9UQB8(2)

839|Q6UXP9(0)

840|Q6ZS27(0)

841|P33240(5)

842|O00142(33)

843|Q9BW11(5)

844|P08910(3)

845|Q07444(0)

846|P48643(13)

847|O14639(7)

848|Q9NQ88(13)

849|Q6IEG0(0)

850|Q8NA31(0)

851|Q6ZRP7(1)

852|Q5VWK0(0)

853|Q9NV72(0)

854|Q5VWK5(105)

855|Q9BVW5(6)

856|Q5T7P8(1)

857|G3V3H7(0)

858|Q15828(39)

859|Q9UL03(19)

860|Q8N8S7(11)

861|P30047(18)

862|Q9BV20(2)

863|Q9Y672(11)

864|Q9UBB5(75)

865|Q9NP79(17)

866|P49862(26)

867|Q9C0C7(4)

868|Q15003(0)

869|Q86WT6(27)

870|P18887(210)

871|Q2UY09(0)

872|Q8TAG9(0)

873|Q9H7F4(0)

874|A0A087WYX8(0)

875|Q5R372(9)

876|P0C7P3(1)

877|Q9H1H9(1)

878|O60240(25)

879|Q6P5X5(0)

880|Q8WYK0(3)

881|Q8TAU0(11)

882|Q6ZMK1(0)

883|P27986(86)

884|O15034(1)

885|P28698(21)

886|Q8NBF6(1)

887|Q56NI9(6)

888|P78423(85)

889|Q9P2H3(12)

890|P08651(19)

891|Q7Z5B4(2)

892|P54826(25)

893|Q96AJ1(4)

894|Q05193(16)

895|Q6ZMY3(0)

896|P52797(9)

897|P46091(1)

898|Q8IYN0(0)

899|Q2Q1W2(2)

900|Q8N0Z9(0)

901|P07814(10)

902|Q9UN37(7)

903|Q8IW19(1)

904|Q32ZL2(2)

905|P98161(75)

906|A6NMS3(0)

907|Q8WV99(2)

908|Q5VW32(0)

909|Q96LP6(1)

910|Q5XUX0(6)

911|Q8IVL5(9)

912|Q8N126(1)

913|Q6S9Z5(0)

914|Q5TF58(0)

915|A0A1B0GX95(0)

916|Q8TCS8(9)

917|P05546(26)

918|Q7L8C5(1)

919|Q8WZ19(2)

920|O94991(1)

921|O94993(2)

922|Q93099(22)

923|Q86X29(10)

924|Q13336(17)

925|Q8N1L9(6)

926|Q9GZV1(4)

927|Q8IV20(7)

928|P0DN78(19)

929|O43490(157)

930|P54793(19)

931|Q6ZMW2(0)

932|Q96HH6(0)

933|O60260(128)

934|O60264(12)

935|Q9Y618(35)

936|Q9Y5H8(1)

937|Q8NDH6(0)

938|O15553(139)

939|Q8IWF2(0)

940|P98187(1)

941|Q9NU19(0)

942|Q7Z2Z1(0)

943|P07360(0)

944|O60313(69)

945|Q8NCY6(0)

946|Q6ZNI0(3)

947|Q14849(8)

948|Q9UBS4(2)

949|Q9UHB7(23)

950|A8MVW5(0)

951|A6NEQ2(0)

952|Q96KN7(12)

953|Q16777(4)

954|Q7Z628(15)

955|P12035(5)

956|Q6ZSA7(0)

957|Q9NR46(3)

958|Q8NF50(25)

959|O43903(4)

960|Q9UJM3(31)

961|Q6IA69(7)

962|Q15269(0)

963|Q9P2U7(6)

964|Q8TDN1(1)

965|Q8TDN6(0)

966|P51784(7)

967|Q9BS86(0)

968|Q86UR5(11)

969|Q6ZTQ4(1)

970|P03923(62)

971|P31271(25)

972|Q86WG3(9)

973|Q9Y661(0)

974|Q9BRT8(0)

975|Q9C009(19)

976|Q9NZR4(24)

977|P13671(12)

978|Q9P055(0)

979|Q86UY8(3)

980|Q8IYB3(0)

981|P0C5K7(0)

982|Q15070(22)

983|Q03933(7)

984|P17600(21)

985|Q86WU2(0)

986|O60469(9)

987|O95235(9)

988|O95232(5)

989|Q6IA86(4)

990|Q6L8Q7(3)

991|Q5TDP6(2)

992|P32519(11)

993|P82933(0)

994|Q5GAN4(1)

995|O15232(12)

996|Q9Y330(0)

997|P02750(16)

998|Q6DJT9(42)

999|Q8WVM7(6)

1000|Q86VQ1(4)

1001|P20702(68)

1002|Q9NTN3(7)

1003|Q14185(18)

1004|P08138(80)

1005|P26045(20)

1006|Q8NEE6(0)

1007|Q9Y3A5(28)

1008|Q6UWT4(0)

1009|Q9BXI3(14)

1010|O95907(4)

1011|Q92858(21)

1012|Q14439(0)

1013|Q8IW00(0)

1014|Q96LQ0(0)

1015|O75674(2)

1016|A0A1B0GW10(0)

1017|Q8N131(0)

1018|O94983(1)

1019|Q8N135(6)

1020|Q9NR11(0)

1021|Q8N9V6(0)

1022|P26373(7)

1023|Q765P7(0)

1024|P00748(36)

1025|O60784(3)

1026|Q96DH6(11)

1027|Q14722(4)

1028|Q14728(0)

1029|Q9Y5W5(70)

1030|Q7Z7D3(39)

1031|Q99590(24)

1032|Q8IV32(0)

1033|Q8IZU1(0)

1034|O94874(5)

1035|O15374(26)

1036|Q96MM6(8)

1037|Q9NYS7(5)

1038|Q9UHR6(2)

1039|Q9Y5I0(0)

1040|Q9NYS0(3)

1041|F5H4B4(0)

1042|Q9HCH0(0)

1043|Q6NSI3(0)

1044|Q5JQC4(2)

1045|Q16586(18)

1046|P03950(78)

1047|Q16589(20)

1048|Q8NEY8(1)

1049|Q96RP9(20)

1050|Q8NAX2(0)

1051|Q9UBR4(14)

1052|Q8ND25(1)

1053|Q96M89(0)

1054|F8WCT9(0)

1055|A4GXA9(0)

1056|Q13536(6)

1057|Q15111(14)

1058|Q4G148(0)

1059|Q06265(1)

1060|Q9UJZ1(15)

1061|Q96A00(11)

1062|Q8IXK0(2)

1063|Q5JTV8(7)

1064|Q96KA5(36)

1065|Q96BH1(0)

1066|Q6UXV0(0)

1067|O00168(3)

1068|Q9NWU1(0)

1069|O00160(2)

1070|Q8N8C0(0)

1071|Q9BYV6(1)

1072|Q9NV56(3)

1073|Q9BYV8(2)

1074|Q1EHB4(0)

1075|Q5T5N4(0)

1076|Q5TCQ9(8)

1077|Q9C030(0)

1078|Q7Z4P5(1)

1079|P52951(4)

1080|Q2L4Q9(0)

1081|Q9NSE4(19)

1082|P22492(2)

1083|Q9NP94(9)

1084|Q9NP91(4)

1085|Q9NP92(16)

1086|Q9Y4X4(5)

1087|Q96QF7(3)

1088|Q9UDX4(6)

1089|Q96ER9(0)

1090|Q6PJ21(0)

1091|Q75QN2(2)

1092|Q96S65(2)

1093|O00587(0)

1094|B9A6J9(0)

1095|Q86WR7(0)

1096|Q9Y236(0)

1097|Q93086(33)

1098|O95222(0)

1099|P09417(21)

1100|O15130(3)

1101|Q9Y6N1(4)

1102|Q5T8A7(0)

1103|Q9Y546(2)

1104|Q9H1J1(1)

1105|Q9NRQ2(6)

1106|Q9Y4B4(0)

1107|Q6NUN9(0)

1108|Q92917(0)

1109|O95822(25)

1110|P05556(96)

1111|Q5M775(10)

1112|P0CAP2(0)

1113|Q15113(4)

1114|Q86W74(1)

1115|Q9P2J3(3)

1116|Q9Y305(2)

1117|Q5VWQ0(6)

1118|Q9P2J8(0)

1119|P14678(17)

1120|Q9BVC3(3)

1121|Q9H845(21)

1122|Q5TZA2(2)

1123|Q7Z5L7(2)

1124|A8MXJ8(0)

1125|Q96Q77(0)

1126|P15923(45)

1127|A6NCL1(0)

1128|Q3KQV3(0)

1129|O60667(3)

1130|P20396(51)

1131|P0C7V0(0)

1132|Q9UKS6(0)

1133|Q8NGF7(0)

1134|P0C7V9(0)

1135|Q6UWY0(1)

1136|Q92466(50)

1137|O43638(0)

1138|Q15937(4)

1139|Q643R3(2)

1140|Q8TD94(0)

1141|A0A0A0MRP1(0)

1142|Q6NUQ1(7)

1143|Q92667(1)

1144|Q92664(10)

1145|Q8N0V3(0)

1146|Q8TEA1(0)

1147|P26368(5)

1148|Q67FW5(5)

1149|Q8NGT5(0)

1150|P0CG08(0)

1151|Q9P1P4(0)

1152|O43707(44)

1153|Q9NXD2(0)

1154|O15482(3)

1155|O15488(1)

1156|Q9H336(0)

1157|P42285(1)

1158|D6RF30(0)

1159|P07902(31)

1160|Q8IV01(0)

1161|Q8IV04(0)

1162|Q8IWV7(5)

1163|Q9BYP9(0)

1164|Q6URK8(2)

1165|Q8IWV1(1)

1166|P32019(1)

1167|Q96RJ6(0)

1168|P16109(124)

1169|P19338(50)

1170|P41002(4)

1171|Q9UPY8(5)

1172|A6NFX1(0)

1173|O60443(11)

1174|Q9UPY6(23)

1175|Q8NEZ2(3)

1176|O60337(1)

1177|P33121(15)

1178|Q16572(7)

1179|A6NE02(0)

1180|A8MV65(4)

1181|Q86SP6(0)

1182|Q53H76(12)

1183|P58170(0)

1184|A8MTY7(0)

1185|O14994(10)

1186|P51553(0)

1187|A6NEW6(0)

1188|Q8NG06(0)

1189|Q9NWZ5(1)

1190|Q96BI1(29)

1191|Q9H5Z6(2)

1192|P47883(0)

1193|Q13568(117)

1194|Q13613(3)

1195|Q96E52(3)

1196|Q16620(57)

1197|Q96T83(0)

1198|Q9NQ38(30)

1199|Q9BV10(1)

1200|O14529(3)

1201|P30307(42)

1202|Q6S8J7(0)

1203|Q5XKE5(0)

1204|Q9C029(0)

1205|P29350(84)

1206|Q9NXL6(1)

1207|Q12906(19)

1208|Q8WWI1(3)

1209|Q8N1Q8(2)

1210|Q5T6C5(0)

1211|Q86XE3(0)

1212|Q6DN14(0)

1213|Q96NG8(8)

1214|Q96H22(0)

1215|Q8NAE3(0)

1216|P10523(34)

1217|Q15054(19)

1218|Q63HQ0(1)

1219|Q96S95(0)

1220|Q8N2E2(0)

1221|Q7Z4G4(0)

1222|Q5SVZ6(0)

1223|Q9Y223(25)

1224|O15105(91)

1225|O00755(30)

1226|Q6UXE8(0)

1227|Q9HBI1(6)

1228|Q9UJA5(3)

1229|E7EVH7(0)

1230|Q9ULH7(3)

1231|Q5GH73(5)

1232|Q9P1A2(0)

1233|Q5JRC9(0)

1234|B4DWF2(0)

1235|P31323(7)

1236|Q92903(7)

1237|O43865(3)

1238|Q8IY85(1)

1239|Q96AY4(2)

1240|A8MXY4(0)

1241|Q9H0W7(0)

1242|O15213(3)

1243|Q70EK8(0)

1244|Q9P2K5(0)

1245|Q9Y6M5(13)

1246|Q9P2K3(2)

1247|Q86XP3(3)

1248|P0DPD5(0)

1249|B7ZC32(0)

1250|Q9NRL2(2)

1251|Q86VS3(1)

1252|P0DPD8(0)

1253|Q9UK53(41)

1254|Q5T5U3(5)

1255|Q7RTS7(8)

1256|Q7RTS6(0)

1257|O75473(44)

1258|Q9H582(2)

1259|Q8IYS2(0)

1260|Q6P2Q9(21)

1261|Q495Y7(0)

1262|Q53FD0(0)

1263|Q9BXK1(1)

1264|Q92696(1)

1265|Q92870(2)

1266|O43609(21)

1267|Q8N6Y1(4)

1268|Q6QNK2(1)

1269|P05534(0)

1270|Q8IYA6(1)

1271|P32314(0)

1272|B2RU33(0)

1273|Q14526(76)

1274|P0CG12(0)

1275|P35442(54)

1276|Q9Y5I4(0)

1277|O43731(0)

1278|Q70Z44(6)

1279|Q9BSY4(0)

1280|A6NIV6(0)

1281|Q9NPB9(7)

1282|P41252(24)

1283|Q496Y0(0)

1284|O96033(5)

1285|P53677(2)

1286|Q14410(0)

1287|A8MXE2(0)

1288|Q8N4E7(12)

1289|Q96RY5(0)

1290|Q7Z7K2(0)

1291|Q96MK3(12)

1292|Q9NYQ7(3)

1293|Q9NZ52(3)

1294|A0A087WZ40(0)

1295|Q8WXT5(0)

1296|P54296(48)

1297|O75121(3)

1298|Q13275(29)

1299|Q5TAA0(0)

1300|A0A1B0GTY4(0)

1301|Q9UQF2(7)

1302|Q9UBT7(6)

1303|Q96D96(7)

1304|P49643(1)

1305|P49641(1)

1306|A8MTZ7(0)

1307|O00299(18)

1308|Q9UFD9(0)

1309|Q8N9S9(0)

1310|Q96PU8(9)

1311|Q8NG11(1)

1312|P80217(7)

1313|Q6ICH7(0)

1314|Q9BY78(2)

1315|Q9Y2L9(6)

1316|Q8N7K0(1)

1317|P13798(10)

1318|Q9UJN7(0)

1319|O95674(2)

1320|Q8N8A8(0)

1321|Q4AC99(0)

1322|Q16630(1)

1323|Q8WXB4(0)

1324|P30460(0)

1325|P80108(9)

1326|Q8NBP0(0)

1327|Q9BVS5(0)

1328|A0A0A0MQZ8(0)

1329|P49821(28)

1330|Q9NQW7(13)

1331|Q7Z4V5(1)

1332|Q8N573(2)

1333|Q9HBX8(23)

1334|Q8WWH4(10)

1335|Q86SG2(0)

1336|Q9NQE7(5)

1337|Q9H8Q6(0)

1338|Q4KMP7(0)

1339|Q15049(21)

1340|Q9BZR6(14)

1341|Q15042(52)

1342|O15119(39)

1343|Q8WWV3(4)

1344|Q13867(12)

1345|P02533(53)

1346|Q8IZ40(0)

1347|Q6V0L0(2)

1348|P57103(2)

1349|Q8NFZ8(6)

1350|Q674R7(5)

1351|Q75VX8(1)

1352|Q86W56(8)

1353|P43246(193)

1354|P43243(33)

1355|Q86W50(0)

1356|C9JLR9(3)

1357|Q66PJ3(0)

1358|Q495X7(0)

1359|P10323(15)

1360|Q9NTI7(2)

1361|Q9BXT2(1)

1362|Q8IYR2(4)

1363|P27708(58)

1364|Q86XR8(20)

1365|Q92800(4)

1366|Q86XR7(106)

1367|P0C7T3(0)

1368|Q8N895(5)

1369|Q13286(30)

1370|Q8NGR2(0)

1371|Q12965(13)

1372|Q13049(27)

1373|P01871(0)

1374|Q13045(57)

1375|O75603(17)

1376|Q8TB37(1)

1377|P23490(24)

1378|Q9H488(3)

1379|Q86VR8(5)

1380|O43502(26)

1381|Q9H310(1)

1382|Q8N3L3(0)

1383|O96005(7)

1384|Q9Y5Z6(2)

1385|Q8N0W7(1)

1386|P36222(121)

1387|Q8WVZ9(1)

1388|O43615(4)

1389|Q9P258(8)

1390|Q92539(19)

1391|Q9H9Y6(0)

1392|Q8N3R3(1)

1393|Q9HCM4(3)

1394|P25800(15)

1395|Q6UWM5(0)

1396|O43189(10)

1397|Q5SXM8(10)

1398|Q16825(3)

1399|Q5TGJ6(0)

1400|Q8NCR9(0)

1401|P50749(34)

1402|A0A1B0GVH7(0)

1403|Q8WUE5(5)

1404|Q4G112(1)

1405|Q9BY89(0)

1406|Q96A58(9)

1407|Q9NT22(1)

1408|P56975(9)

1409|Q68EM7(0)

1410|Q9UJQ4(36)

1411|Q6L8G9(0)

1412|O00116(3)

1413|Q6L8G4(0)

1414|P49747(53)

1415|Q96MX3(0)

1416|P38646(44)

1417|Q8TBJ5(3)

1418|Q9NS39(5)

1419|Q5TAT6(18)

1420|Q9BRJ6(0)

1421|Q9HCC8(0)

1422|Q9HCC9(1)

1423|A6NDY2(0)

1424|Q6ZVF9(0)

1425|Q8TDD2(4)

1426|Q96EA4(0)

1427|Q03468(104)

1428|Q15554(51)

1429|Q96GM5(6)

1430|Q9BRX2(1)

1431|Q9NQV5(0)

1432|Q96NW4(0)

1433|Q9NZN8(1)

1434|Q01658(2)

1435|Q06546(152)

1436|P57775(18)

1437|P56192(17)

1438|Q9UBK8(129)

1439|Q8NAG6(2)

1440|P56199(22)

1441|Q9HBK9(16)

1442|Q9Y2Y4(6)

1443|Q93075(0)

1444|P28066(16)

1445|P52434(1)

1446|Q8WYN0(7)

1447|Q6ZV73(2)

1448|P00966(66)

1449|Q5JRA6(16)

1450|Q6PF18(0)

1451|Q9BT17(84)

1452|O43889(16)

1453|Q9BVN2(0)

1454|Q9UJ55(30)

1455|Q9H0U9(25)

1456|Q9H0U6(1)

1457|Q9Y376(4)

1458|Q9NTJ3(15)

1459|P32249(6)

1460|A0A0K0K1A3(0)

1461|Q96QZ7(7)

1462|Q9Y3E2(0)

1463|A6NDE4(0)

1464|Q68CJ6(0)

1465|Q8NGE7(0)

1466|Q9BXU1(7)

1467|Q969E2(0)

1468|Q969E3(26)

1469|P00367(24)

1470|Q92819(33)

1471|Q9Y572(47)

1472|Q8TCN5(0)

1473|Q7Z6M4(0)

1474|Q96LM6(0)

1475|Q92499(13)

1476|Q8N884(4)

1477|Q8NEK5(0)

1478|Q02817(0)

1479|Q658P3(15)

1480|Q9Y4A8(5)

1481|Q8IYG6(0)

1482|Q8TB24(1)

1483|Q14541(4)

1484|Q6ZUJ8(3)

1485|P60508(1)

1486|O60749(2)

1487|Q14549(2)

1488|Q9NRJ4(0)

1489|P51610(16)

1490|Q75N90(5)

1491|O96014(32)

1492|Q8IV76(5)

1493|P46937(73)

1494|A6NM11(1)

1495|Q9H307(10)

1496|Q99719(13)

1497|Q8IZQ8(25)

1498|Q8N660(0)

1499|Q3YBR2(7)

1500|Q14161(6)

1501|P41586(26)

1502|Q15637(12)

1503|Q5TGI0(0)

1504|Q969F8(36)

1505|A6NFU0(0)

1506|O75145(0)

1507|Q5VUJ6(0)

1508|Q6ZWT7(0)

1509|Q8TEJ3(0)

1510|Q0VG06(0)

1511|Q6P4R8(1)

1512|Q9UBV2(33)

1513|P48764(24)

1514|Q6P4Q7(18)

1515|Q5H8A3(7)

1516|Q96DF8(12)

1517|Q6BAA4(5)

1518|Q8IUQ4(18)

1519|Q7KYR7(6)

1520|Q68EN5(0)

1521|Q6ZQN7(2)

1522|Q86YW0(3)

1523|Q8NFG4(27)

1524|Q6ZS86(0)

1525|B9A070(0)

1526|Q15415(0)

1527|Q96KW9(0)

1528|Q9ULL1(1)

1529|Q9NWQ4(0)

1530|Q8NA92(0)

1531|O14893(25)

1532|Q9BYR4(0)

1533|Q5FWE3(0)

1534|Q16610(34)

1535|Q9H0D6(4)

1536|Q7Z417(8)

1537|Q7Z410(0)

1538|Q96NT3(2)

1539|Q7Z4T8(2)

1540|Q7Z4T9(1)

1541|Q15561(6)

1542|Q9NSY0(1)

1543|P16402(2)

1544|P08949(10)

1545|Q6E0U4(7)

1546|Q8NHY6(0)

1547|Q8NHY0(8)

1548|Q9HBZ2(31)

1549|Q8WWF6(1)

1550|P55895(85)

1551|P15813(67)

1552|P15812(4)

1553|Q969V3(0)

1554|Q15388(0)

1555|O00257(9)

1556|Q9HBL0(33)

1557|Q96H55(0)

1558|Q8WWT9(1)

1559|Q6UXH1(1)

1560|Q9Y2X0(1)

1561|P52848(16)

1562|Q8IZ20(2)

1563|Q15154(14)

1564|P17542(39)

1565|Q00325(46)

1566|C9J3V5(0)

1567|Q9P2N6(0)

1568|Q9P2N7(0)

1569|O60566(69)

1570|Q49AJ0(0)

1571|Q86W34(0)

1572|O95886(4)

1573|O75443(6)

1574|Q8IY33(2)

1575|Q8N4T0(8)

1576|B9ZVM9(0)

1577|Q86UP8(1)

1578|A8MX19(0)

1579|Q96SK2(2)

1580|Q8IVP9(0)

1581|F5H7D0(0)

1582|Q8IVP5(0)

1583|Q99871(0)

1584|Q05682(44)

1585|P0C7X1(0)

1586|Q92628(0)

1587|Q96P47(0)

1588|Q8TCY9(16)

1589|Q9H8K7(3)

1590|Q9H7D0(0)

1591|P0CG42(0)

1592|O95260(1)

1593|Q7Z5H4(1)

1594|P43686(4)

1595|Q13393(27)

1596|P27216(3)

1597|A8MPS7(2)

1598|Q8N988(0)

1599|Q86TV6(2)

1600|Q6ZMQ8(3)

1601|P60409(0)

1602|O43678(27)

1603|Q15629(20)

1604|O95461(47)

1605|Q15622(0)

1606|Q6PRD7(4)

1607|Q86Y38(9)

1608|P15170(13)

1609|Q9NZ01(3)

1610|Q9H209(45)

1611|Q16533(2)

1612|O75150(4)

1613|Q6NUK1(0)

1614|Q9GZX3(3)

1615|Q6ZNA1(0)

1616|P85298(1)

1617|Q6ZUA9(0)

1618|Q9NSV4(7)

1619|Q8N7X4(3)

1620|O43300(1)

1621|O43303(2)

1622|A0A1B0GTR0(0)

1623|Q6PIL6(11)

1624|Q9NWF9(12)

1625|A8MXQ1(0)

1626|Q96MH6(0)

1627|Q8NDB2(14)

1628|Q8NE28(0)

1629|P30501(0)

1630|Q9Y2I8(7)

1631|Q68EA5(0)

1632|Q9ULK4(16)

1633|Q8N201(8)

1634|J3QLG5(0)

1635|A6NH11(0)

1636|Q8IWN7(0)

1637|A0A286YFL2(0)

1638|Q9UPS8(6)
